# Photocatalysis as a mechanistic probe for the Staudinger β-lactam synthesis

**DOI:** 10.1016/j.checat.2025.101493

**Published:** 2025-11-20

**Authors:** Mihai V. Popescu, Nicholas A. Parker, Zhuqing Jia, Pearse Solon, Callan J. Maloney, Juan V. Alegre-Requena, Robert S. Paton, Martin D. Smith

**Affiliations:** 1 Chemistry Research Laboratory, University of Oxford, 12 Mansfield Road, Oxford OX1 3TA, UK; 2 Department of Chemistry, Colorado State University, 1301 Centre Avenue, Fort Collins, CO 80523-1872, USA; 3 Departamento de Química Inorgánica, Instituto de Síntesis Química y Catálisis Homogénea (ISQCH), CSIC-Universidad de Zaragoza, Calle Pedro Cerbuna 12, Zaragoza 50009, Spain; 4 These authors contributed equally; 5 Lead contact

## Abstract

In this study, we demonstrate that a visible-light-mediated energy transfer process can serve as a mechanistic probe in the Staudinger cyclization. Ikeda demonstrated that N-acyl enaminone substrates undergo diastereoselective cyclization to form **β**-lactams under UV irradiation. We reinvestigated this process, employing energy transfer catalysis and Hammett studies, which enabled us to conclude that these reactions proceed in the T_1_ state via the formation of a zwitterionic intermediate. This is in contrast to previous explanations, which suggest that the reaction proceeds via the S_1_ state and involves a ground-state open-shell biradical. In-depth computational investigations support the zwitterionic hypothesis, with the final ring-closing process being radical. The rate of intersystem crossing was found to be critical in controlling the geometry of the zwitterionic intermediate, being ultimately responsible for the observed diastereoselectivity. Finally, a crossover experiment confirms reversibility in formation of the zwitterionic intermediate, experimentally validating a long-standing hypothesis.

## In brief

The presence of high-energy zwitterionic intermediates is commonly postulated in many thermal transformations, with the Staudinger **β**-lactam synthesis being one of the most famous examples. Here, we demonstrate that, using triplet energy transfer catalysis, the same intermediates can be accessed indirectly, providing a powerful mechanistic probe for interrogating these species. Using a combination of experimental and computational methodologies, we were able to gain a deeper insight into the Staudinger **β**-lactam synthesis.

## Graphical Abstract

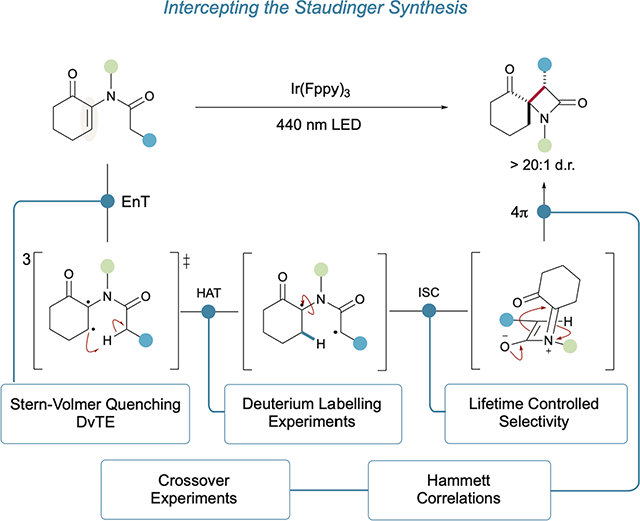

## INTRODUCTION

**Figure 1.**
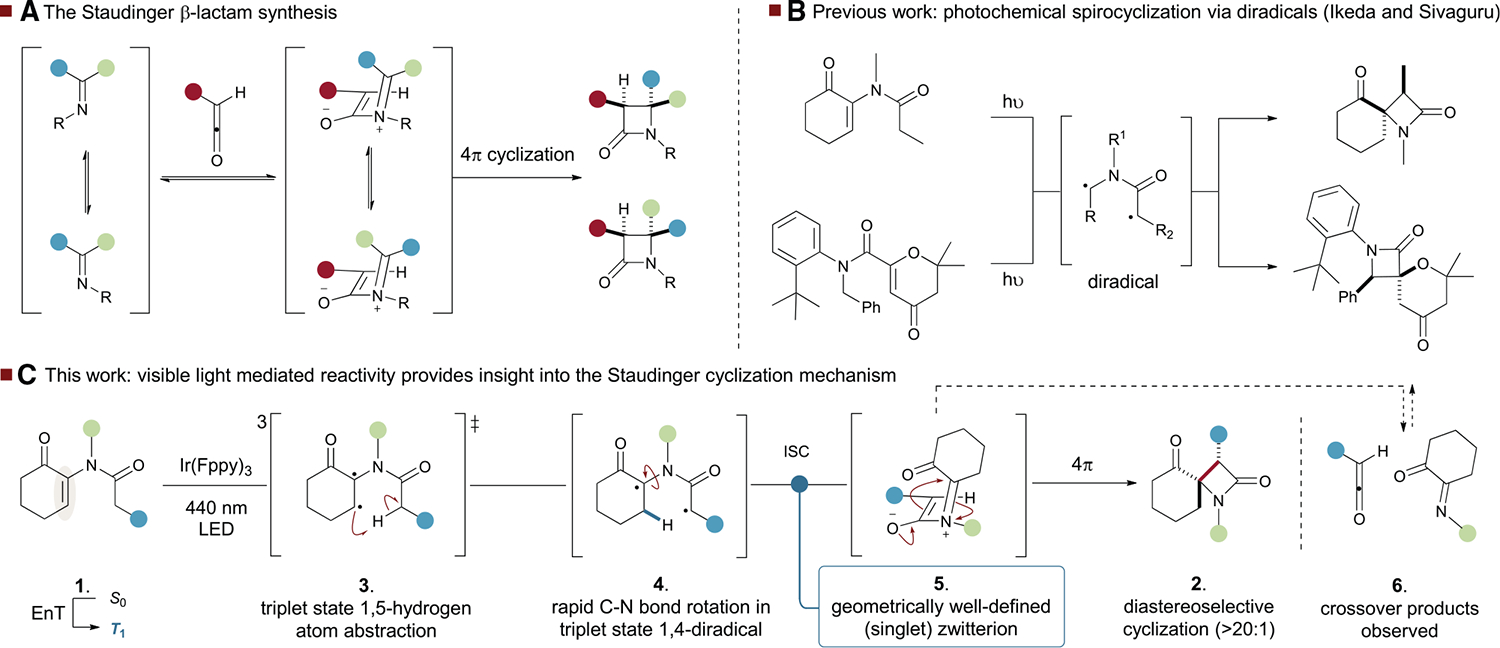
Overview of the Staudinger β-lactam synthesis (A) The Staudinger cyclization is proposed to proceed via a zwitterion and yields β-lactams through a conrotatory cyclization. (B) Previous photochemical spirocyclizations to generate β-lactams via a diradical recombination. (C) This work: a visible light photocyclization yields β-lactams via a geometrically well-defined zwitterionic intermediate and confirms crossover from zwitterionic intermediates. EnT, energy transfer; ISC, intersystem crossing.

The β-lactam is an important structural motif^1^ prevalent in essential medicinal compounds, as exemplified by penicillins and cephalosporins.^2,3^ The most common method for its synthesis is the classical Staudinger ketene-imine cyclization,^4^ which can be highly effective but is limited by challenges associated with the generation of substituted ketenes.^5^ This reaction has been intensively investigated since its discovery over 100 years ago.^6–12^ More recently, computational techniques in conjunction with physical organic chemistry methods have begun to elucidate the mechanistic details of this venerable process. The reaction is broadly accepted to proceed via the addition of an imine to a ketene to form an intermediate zwitterion, which can undergo cyclization via a conrotatory mechanism to generate the observed β-lactam products. The observed stereoselectivity is a consequence of a panoply of factors: the stereochemistry of the starting materials, the facial selectivity of the ketene-imine addition, reversibility in this step, bond rotation and interconversion of zwitterionic intermediates, and also the relative rates of cyclization of stereoisomeric zwitterions (Figure 1A). That all of these factors become more or less dominant depending on substitution patterns and reaction conditions characterizes a process that is complex and nuanced.^13–16^ An alternative strategy to generate the β-lactam nucleus is to first form the amide bond and subsequently form the carbon–carbon bond in an independent intramolecular step.^17,18^ This approach has been employed in the photo-mediated spirocyclization of enone derivatives, which is proposed to proceed via photoexcitation followed by 1,5-hydrogen atom transfer (HAT), ultimately leading to recombination of the resulting 1,4-diradical in the singlet state (Figure 1B).^19–23^ Ikeda^24,25^ and Piva^26^ demonstrated that this type of cyclization can be achieved by direct excitation with UV light, and Ikeda suggested that the reaction occurs in the S_1_ state, as the addition of benzophenone does not accelerate the reaction rate. Sivaguru has demonstrated that a triplet-mediated cyclization on an enantioenriched substrate can be sensitized using a thioxanthone photocatalyst and visible (420 nm) light.^27^ In this process, the diastereo- and enantiocontrol is a consequence of restricted rotation about a C–N bond. Elegant work from the Bach group has built on this observation to deliver spirocyclic azetidine-indolines in good to excellent diastereoselectivity via 1,4-radical recombination.^28^ The Petersen group has disclosed a related reaction that yields β-lactams; in this case, a radical recombination process is proposed to rationalize the lack of observed diastereoselectivity.^29^ The high levels of diastereoselectivity observed by Ikeda across a range of substrates led us to speculate that this could be a consequence of a cyclization from a geometrically well-defined zwitterion rather than a diradical.^30,31^ Resonance between open-shell singlet and closed-shell zwitterionic configurations has important implications, as the properties of diradicals^32^ are best described as the combination of multiple electronic states.^33–38^

We reasoned that a combined synthetic and computational approach to interrogating this photoreaction could provide a new avenue to intercept the postulated zwitterionic intermediate in the Staudinger cyclization. This is valuable, as the high reactivity of ketenes and imines can preclude examination of the elementary steps in zwitterion formation. Here, we show that visible-light sensitization of enones, such as **1**, ultimately led to complete diastereoselectivity in the formation of β-lactam **2**. We propose that this diastereoselective cyclization arises from 1,5-HAT from the triplet diradical **3** to yield 1,4-diradical **4** as an equilibrating mixture of isomers from rapid rotation about the C–N bond. Intersystem crossing (ISC) back to the singlet state from the most populated rotamer yields a well-defined zwitterion with diradical character **5** (Figure 1B). This intermediate is identical to the postulated zwitterion in the Staudinger cyclization. Cyclization from this zwitterion is completely diastereoselective, consistent with the Staudinger cyclization—and the presence of this species is inferred through the observation of crossover products derived from the reversible formation of ketene and imine intermediates **6**.

## RESULTS AND DISCUSSION

To test our hypothesis, we prepared substrate **1a** in three synthetic steps. Nucleophilic epoxidation of cyclohexenone with basic hydrogen peroxide, followed by treatment of the resulting epoxide with benzylamine in a 3:1 mixture of methanol:water at 80°C, and subsequent acylation with propionyl chloride gave the desired test substrate **1a**. Ikeda disclosed that irradiation of this substrate with UV light led to a 10:1 ratio of diastereoisomers,^25^ and hence, we investigated the influence of irradiation wavelength on reactivity with this substrate. Purple light (390 nm) was found to induce excited-state reactivity in the absence of a photocatalyst, enabling the formation of spirocyclic β-lactam **2a** in 55% yield as a single diastereoisomer, alongside γ-lactam **7a** as a side product (in a 10:1 regioisomeric ratio [r.r]). This is consistent with the observation from Ikeda that the reaction generates two products, but they are not diastereoisomers as suggested. Subsequently, we examined the reactivity of **1a** under irradiation with blue light-emitting diode (LED) light (440 nm) in the presence of commercial photocatalysts. Our previous work in this area^39^ has demonstrated that α-heteroenones are competent substrates for sensitization using visible light in combination with a suitable photocatalyst to allow triplet energy transfer (EnT) to take place (Table 1).^40,41^

**Figure 2.**
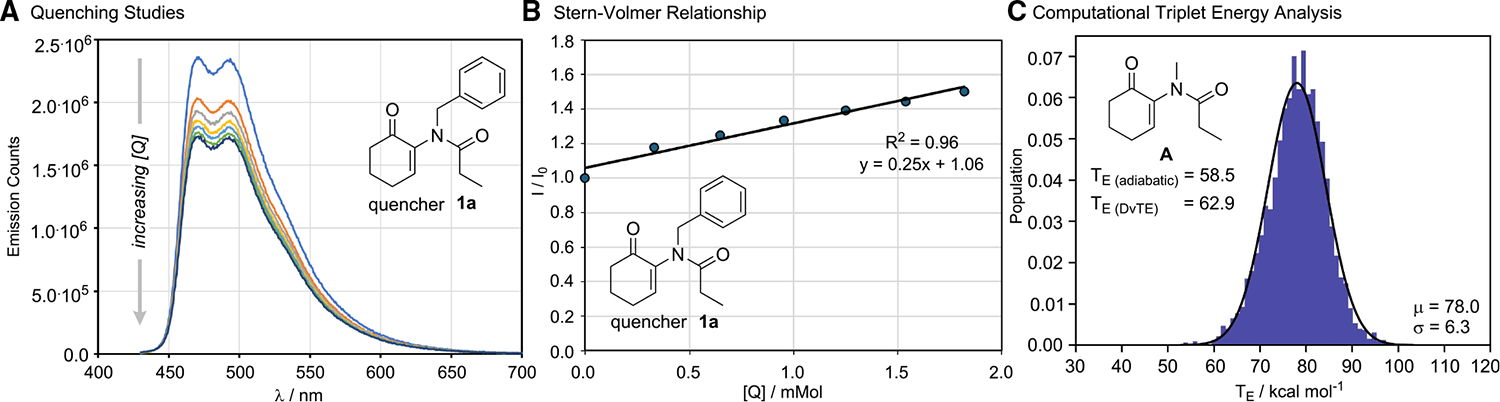
Triplet energy transfer photophysical studies (A) Quenching studies of Ir(dFppy)_3_ against quencher **1a** measured in EtOAc. (B) Stern-Volmer relationship using the 470-nm change in emission. (C) Computational analysis of triplet energy of system **1l** using both a static adiabatic picture (M06–2X/def2-TZVP//M06–2X/6–31+G(d,p)) and the DvTE protocol (M06–2X/6–31G(d)//M06–2X/MIDI!).

When changing the irradiation source to 440 nm, no conversion to β-lactam **1a** was observed, leading to complete recovery of the starting material. We subsequently employed 1 mol % Ir (dFppy)_3_ as the photocatalyst at 440 nm irradiation, which led to the desired product **2a** in a 69% isolated yield as a single diastereoisomer alongside γ-lactam figure **7a** (ratio 10:1), consistent with triplet sensitization. The reaction was found to be sensitive to the presence of oxygen but tolerant of moisture; no precautions were taken in drying the solvent or reagents before use. A series of metal-free sensitizers were also evaluated but were found to be less effective (see Table S1 and Figure S2). Quenching studies showed a typical linear Stern-Volmer relationship, indicating quenching of the excited photocatalyst by the substrate **1a** (Figure 2). The observed change in emission intensity at 470 nm with increasing concentrations of the substrate was found to correspond to a Stern-Volmer quenching constant of K_SV_ = 0.25 mM (for further details, see Figures S3 and S4). Using previous literature reports of an excited triplet state lifetime of τ = 1.6 μs,^42^ a bimolecular quenching constant between Ir (dFppy)_3_ and **1a** was calculated to be k_q_ = 1.56 × 10^8^ M^−1^s^−1^.

This quenching constant was found to be smaller than the expected half of the diffusion-limited kinetic quenching for a thermoneutral sensitization process according to the Sandros-Boltzmann relationship, suggesting that the substrate possesses a triplet energy higher than that of the photocatalyst (T_E_ = 61.3 kcal/mol; see Figures S25 and S26). Indeed, using our recently disclosed dynamic vertical triplet energy (DvTE)^43^ theoretical model on a truncated system **A**, a triplet energy of 62.9 kcal/mol was estimated, consistent with empirical observations. This is in contrast to the standard adiabatic model, which predicts a triplet energy of 58.4 kcal/mol.

**Figure 3.**
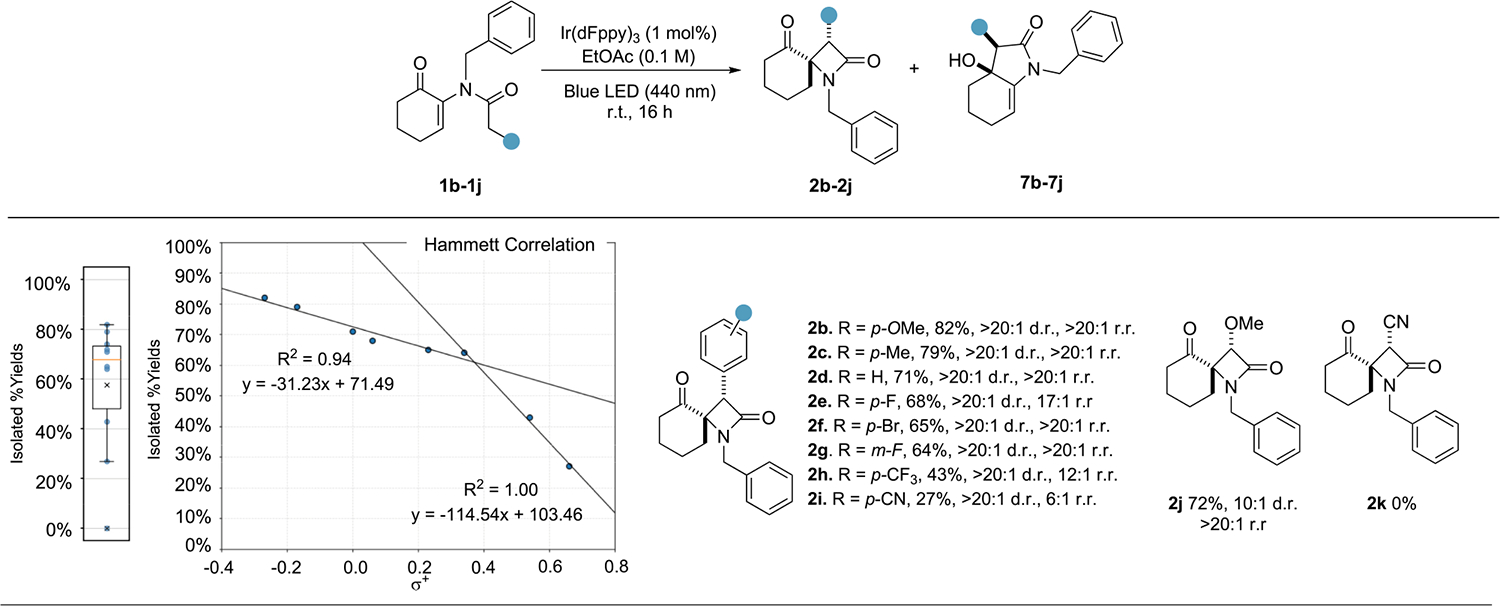
Scope of triplet energy transfer-mediated spirocyclization Reaction conditions: 0.3 mmol enone, 1 mol % Ir(dFppy)_3_, 34 W blue Kessil LED lamp, EtOAc ([enone] = 0.1 M); 16 h. Yields are for isolated material. Diastereoisomeric ratio (d.r.) and regioisomeric ratio (r.r.) determined by ^1^H-nuclear magnetic resonance (NMR) spectroscopy from crude reaction mixture.

To further gain insight into the mechanism of this transformation, we performed a series of substrate modifications. We hypothesized that the reaction proceeds via a classical Staudinger-like zwitterionic intermediate **5**, and consequently, it was expected that structural modifications on the β carbon of the amide would have significant contributions to the reaction outcome, in line with previous mechanistic studies (Figure 3).^44^ Investigation of the cyclization of a series of *p-* and *m*-substituted benzyl substrates (to yield **2b**–**2i**) revealed a strong dependency between reaction yields and electronics of the aryl substituent. Two regression lines with high coefficients of determination could be obtained between the σ+ Hammett parameters and the observed isolated yields, indicating a potential switch in the reaction rate-determining step within the electron-withdrawing σ+ region of 0.3–0.5. The highest yielding substrate was found to be the *p*-OMe-substituted aryl derivative **2b**, leading to an isolated yield of 82%, while strong electron-withdrawing groups were found to lead to lower isolated yields (such as 27% for the *p*-CN derivative **2i**). Intriguingly, high diastereoselectivity was maintained across the series, while the regioselectivity was found to decrease with increasing electron-with-drawing capabilities of the substituent, as exemplified by *p*-CF_3_ (**2h**) and *p*-CN (**2i**) derivatives, which led to 12:1 and 6:1 r.r., respectively (relative configuration was validated by single-crystal X-ray diffraction).^45^ To further test these observations, we investigated two additional substrates where the functional groups were placed directly on the β carbon of the amide carbonyl. Consistent with the aromatic series, a substrate bearing a methoxy group led to **2j** in a 72% isolated yield (10:1 diastereoisomeric ratio [d.r.] and >20:1 r.r.), while the substrate with a nitrile group in this position led to a complex mixture of products, and no desired product **2k** could be isolated. Additional investigations into the effect of structural modifications on the *N*-alkyl substituent and enone motif did not indicate any systematic deviation from the yield and selectivity observed above, suggesting that they do not play a significant mechanistic role (Figure S1).

**Figure 4.**
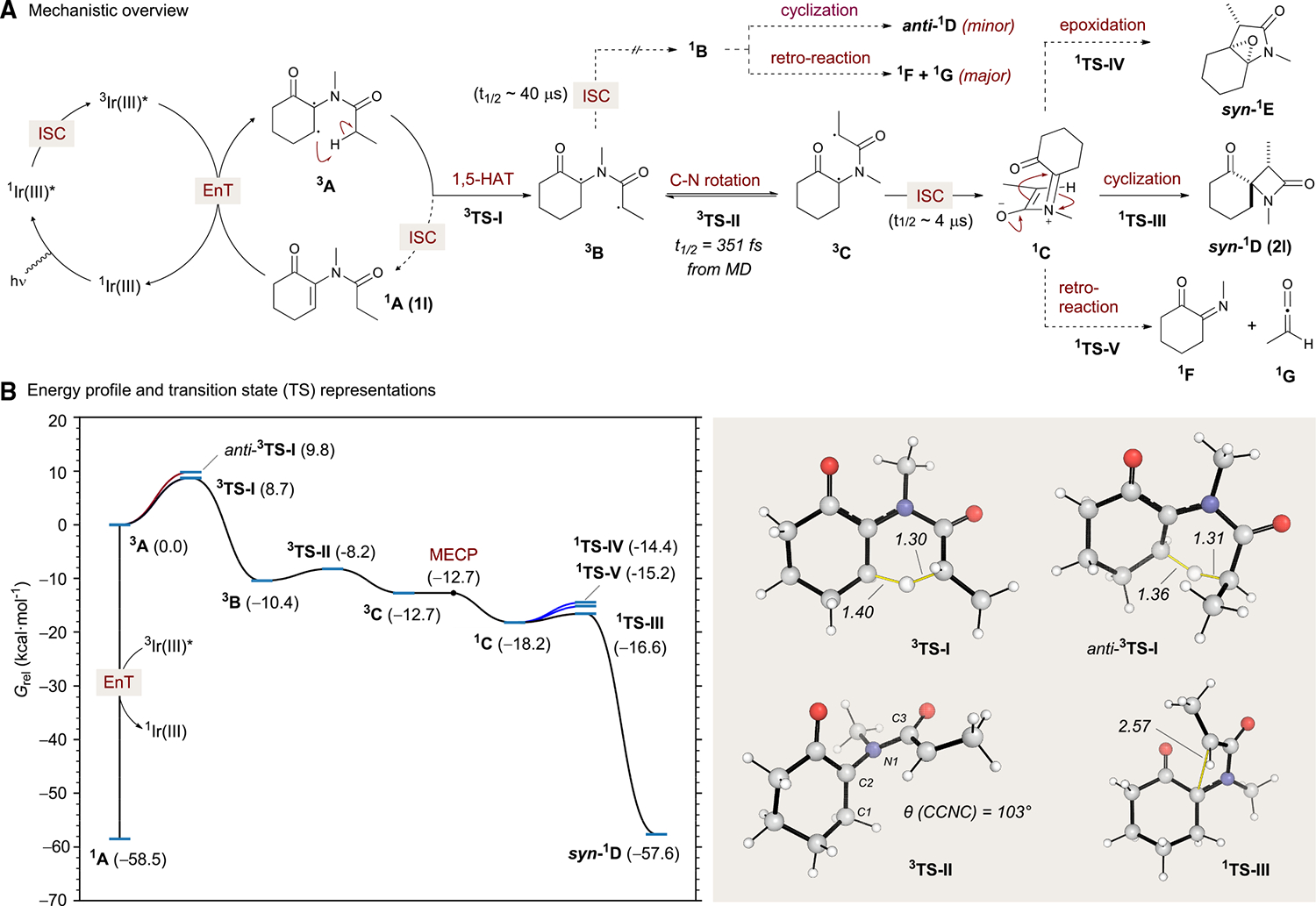
Density functional theory modeling of the main reaction pathways (A) Reaction mechanism after singlet-to-triplet excitation of the starting cyclohexenones. (B) (i) Boltzmann weighted Gibbs free energy profile obtained at the M06–2X/def2-TZVP//M06–2X/6–31+G(d,p) level. Calculations include the CPCM solvation model (solvent = EtOAc). Other levels of theory lead to similar profiles (see supplemental information for further details). (ii) Transition structures for key transformations (EnT, energy transfer; ISC, intersystem crossing; HAT, hydrogen atom transfer; MECP, minimum energy crossing point; MD, molecular dynamics.).

With additional insight from the substrate scope, we turned to quantum chemical calculations to determine a more complete mechanistic profile.^46^ After triplet EnT to substrate ^**1**^**A** (experimental substrate **1l**) by the catalyst to generate ^**3**^**A**, a 1,5-HAT (^**3**^**TS-I**, ΔG^‡^ = 8.7 kcal/mol) takes place via a transition state (TS) that places the methyl group *syn* to the carbonyl to give intermediate ^**3**^**B** (Figure 4). A competitive 1,5-HAT leading to the *anti*-conformer was also calculated; although this pathway is dis-favored (*anti-*^**3**^**TS-I**, ΔG^‡^ = 9.8 kcal/mol, Figure 3B), the resulting intermediate can rotate about a C–C bond to converge upon intermediate ^**3**^**B**. Subsequently, intermediate ^**3**^**B** can proceed to intersystem cross to its corresponding ground-state singlet ^**1**^**B** or, alternatively, rotate about the C–N bond (^**3**^**TS-II**) to facilitate the formation of intermediate ^**3**^**C** instead (for an extensive investigation of alternative reaction pathways and benchmarking results, see Figures S14–S19).

While the calculation of minimum energy crossing points (MECPs) for ^**3**^**B** and ^**3**^**C** suggests that ISC should be highly facile (<0.1 kcal⋅mol^−1^ energetic cost to reach the MECPs for both intermediates; see Table S2 and Figure S20), the rotation about the C–N bond from ^**3**^**B** to ^**3**^**C** via ^**3**^**TS-II** is also highly favorable, with a Gibbs activation barrier of only 2.2 kcal/mol. Further examination of the potential energy surface (PES) following the formation of ^**1**^**B** suggests that this reaction pathway favors fragmentation to a ketene (^**1**^**F**) and an imine (^**1**^**G**) (herein referred to as the “retro-reaction”) over the formation of *anti-*^**1**^**D** by a conrotatory electrocyclization. The formation of the anti-β-lactam product is not experimentally observed, suggesting that a pathway that proceeds via ISC to generate ^**1**^**B** does not occur primarily. To further resolve this mechanistic conundrum, we proceeded to estimate the rates of ISC using time-dependent density functional theory (TD-DFT; see the supplemental information for computational details). Evaluation of Fermi’s golden rule for intermediates ^**3**^**B** and ^**3**^**C** suggests that the lifetime of both intermediates toward ISC lies in the μs region (t_1/2_ ~40 and ~3 μs, respectively), which is consistent across a series of range-separated functionals (Table S5) and orders of magnitudes slower than the rate of rotation based on the activation barrier for ^**3**^**TS-II**. Quasi-classical molecular dynamics (MD; M06–2X/6–31+G(d,p)) was used to estimate the average time required to proceed from 1,5-HAT ^**3**^**TS-I** to rotation ^**3**^**TS-II** in 351 fs based on 100 trajectories initiated from the geometry of ^**3**^**TS-I**, with the accumulation of ^**3**^**C** after the TS is crossed (Figure S24; Table S6). This result is consistent with rapid equilibration between ^**3**^**B** and ^**3**^**C**, which occurs much more quickly than the calculated ISC rates. Consequently, equilibration ^**3**^**B** to (the more stable) ^**3**^**C** is enabled by a rotation about the C–N bond (^**3**^**TS-II**), which is faster than the corresponding ISCs to ^**1**^**B** and ^**1**^**C** (*vide infra*). ISC will occur from the most populated conformer at equilibrium (^**3**^**C**) to generate ^**1**^**C**. In addition to facile electrocyclization (barrier of 1.6 kcal/mol via ^**1**^**TS-III**), two additional competitive pathways, namely cyclization onto the carbonyl carbon (via **1TS-IV**) and fragmentation to the corresponding ketene and imine (via ^**1**^**TS-V**), were also found to be accessible, requiring activation energy barriers of 3.8 and 3.0 kcal/mol, respectively. Following the intrinsic reaction coordinates (IRCs) of ^**1**^**TS-IV**, epoxide *syn-*^**1**^**E** was ultimately identified as a (hypothetical) reaction by-product. The observed γ-lactam by-product **3a** is derived from this species via a ring-opening and elimination process. The overall reaction from ^**1**^**A** is slightly exergonic (ΔG = 0.9 kcal/mol) but irreversible, as the product β-lactam ^**1**^**D** is not photoreactive under the reaction conditions.

Diradical character in the Staudinger cyclization has been previously reported by Cossio.^47^ Our results are consistent with this and indicate that zwitterions and open-shell singlets may be found on the same potential energy surface of the Staudinger ketene-imine cyclization. Using the broken symmetry formalism, we were able to identify multiple conformers of intermediate ^**1**^**C**, some of which were closed-shell zwitterionic species and others that were open-shell diradical singlets (< S^2^ > 0.09 upon spin annihilation; Table S7). The open-shell structures were found to be 4.3–4.7 kcal/mol higher in energy than the corresponding closed-shell structures, suggesting that intermediate ^**1**^**C** is best described as a zwitterion.^48,49^ On the other hand, the open-shell electronic structures play a crucial role in the cyclization of ^**1**^**C**, as all closed-shell cyclization transition structures (^**1**^**TS-III**) were found to be in excess of 6 kcal/mol higher energy than their corresponding open-shell versions. As such, under an exclusive closed-shell cyclization model, the predicted reaction outcome would be the retro-reaction and epoxide formation (via ^**1**^**TS-IV** and ^**1**^**TS-V**).^50^ While this behavior is likely a limiting case that may not be universally applicable to all Staudinger cyclization processes, it should be considered in future studies.

**Figure 5.**
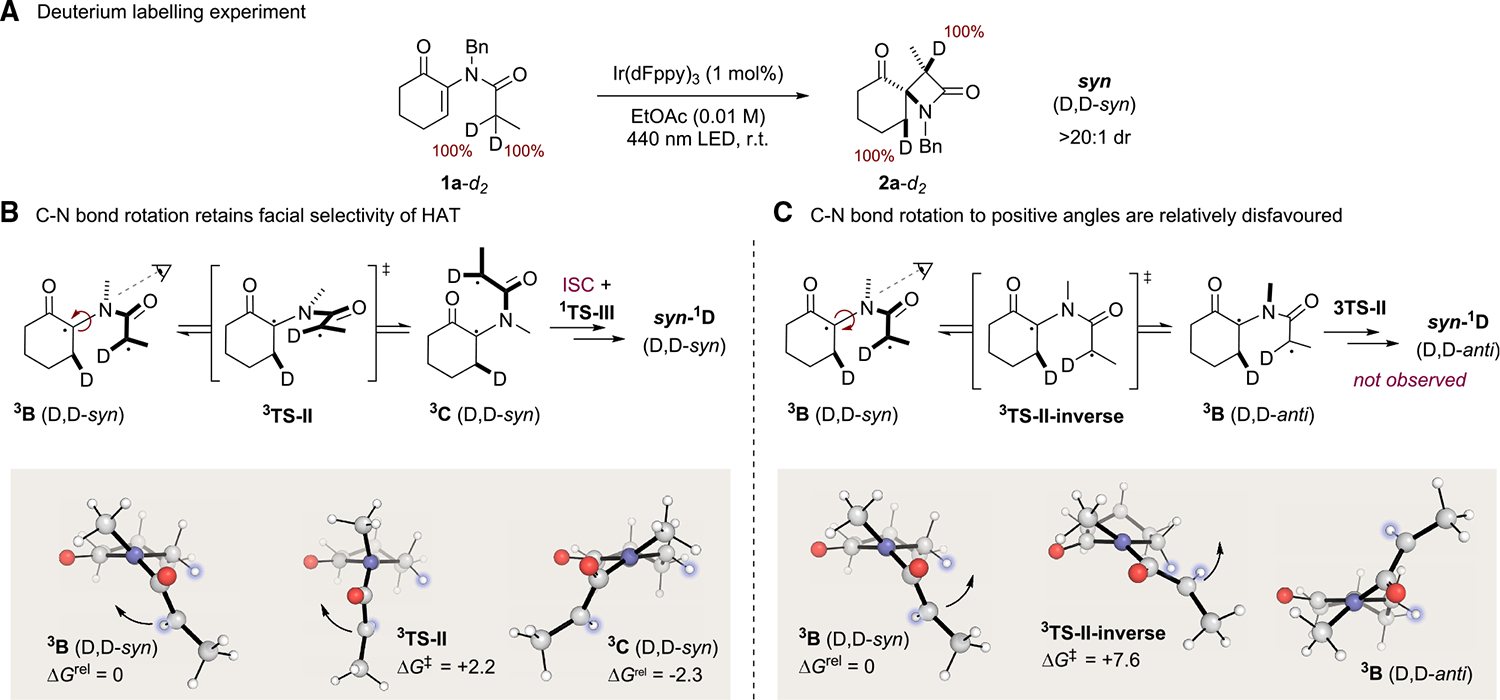
Deuterium labeling studies for hydrogen atom abstraction (A) Deuterium labeling experiment showing complete diastereoselectivity in both the 1,5-HAT and subsequent cyclization (conditions: 0.3 mmol enone, 1 mol % Ir (dFppy)_3_, 34 W blue Kessil LED lamp, EtOAc ([enone] = 0.1 M); reaction time 16 h). (B) Face selectivity in cyclization is determined by rotation onto the same face of the cyclohexanone as HAT occurred via ^**3**^**TS-II**. (C) Rotations onto the opposite face of the cyclohexanone are significantly disfavored.

In order to unequivocally confirm that the reaction proceeds via a 1,5-HAT, we prepared substrate **1a-d**_**2**_ bearing two deuterium atoms in the position α to the amide carbonyl. Subjecting this substrate to our optimized reaction conditions led to the formation of the doubly deuterated product **2a**-*d*_*2*_ with 100% deuterium incorporation as a >20:1 mixture of diastereoisomers (Figure 5A; for further details, see Figures S5–S7). This is consistent with the 1,5-HAT reaction as a key mechanistic step but also demonstrates that the hydrogen atom abstraction itself and the subsequent spirocyclization are both highly diastereoselective. In this process, the initial 1,5-HAT installs a deuterium atom β to the ketone (via ^**3**^**TS-I**), which also places the pendant amide on the same face of the cyclohexanone. Fast C–N rotation leads to the interconversion of ^**3**^**B** and ^**3**^**C** via ^**3**^**TS-II**, both of which lie on the same face of the cyclohexanone. This ultimately directs the facial selectivity (via ISC) to generate geometrically well-defined zwitterion ^**1**^**C**, which leads to carbon–carbon bond formation on the same face as the HAT (Figure 5B).

**Figure 6.**
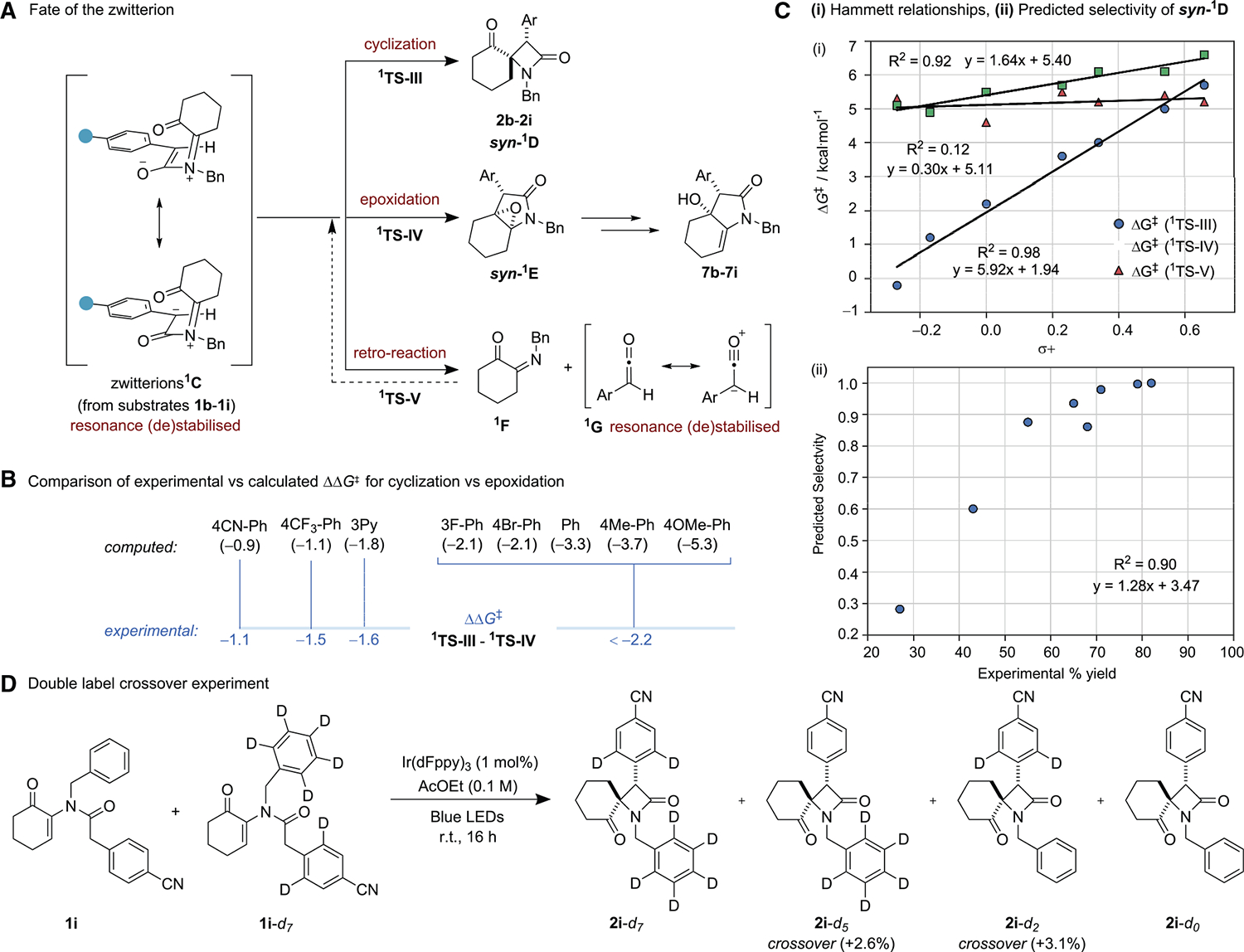
Explanation of stereoelectronic effects on reaction outcome (A) Calculation indicates that zwitterions ^1^C (from substrates **1b**–**1i** bearing different *para*-substituents on the α-aryl group) have three viable fates: cyclization to β-lactams *syn*-^1^D (**2b**–**2i**, via ^**1**^**TS-III**); cyclization onto the ketone to give epoxide *syn*-^**1**^**E** (via ^**1**^**TS-IV**), which ultimately yields lactams **7b**–**7i**; and retro-reaction to give imines ^**1**^**F** and ketenes ^**1**^**G** (via ^**1**^**TS-V**). (B) Experimental vs. calculated (in parentheses) ΔΔG^‡^ of the cyclization and epoxidation TSs for substrates **1b**–**1i** bearing different *para*-substituents on the α-aryl group in kcal/mol. (C) (i) Computed activation energy barriers for ^**1**^**TS-III**, ^**1**^**TS-IV**, and ^**1**^**TS-V** vs. Hammett σ+ constants for substrates **1b**–**1i**. (ii) Predicted selectivity for the formation of *syn*-^1^D based on computed relative rates (selectivity = k(^**1**^**TS-III**)/k(^**1**^**TS-III**) + k(^**1**^**TS-IV**) + k(^**1**^**TS-V**)). (D) Double-label crossover experiment with substrate **1i** (that bears a *para*-cyano group on the α-aryl group). Crossover product enrichment is expressed as (%) increase in total ion count vs. controls, normalized to most abundant parent ion by quantitative LC-MS analysis of purified product.

To interrogate broader trends in selectivity and reactivity, we examined the cyclization of aromatic amides **1b**–**1i** since they display a range of yields and product selectivity. Zwitterionic intermediate ^**1**^**C** can, in principle, follow three main pathways that lead to different products (Figure 6A). ^**1**^**TS-III** corresponds to a conrotatory cyclization leading to *syn*-β-lactam products **2b**–**2i**, whereas ^**1**^**TS-IV** embodies a pathway where the enolate carbon of the zwitterion ^**1**^**C** attacks the ketone group, leading to epoxide *syn-*^**1**^**E** (which ultimately generates the experimentally observed γ-lactam products **7h** and **7i**). ^**1**^**TS-V** is a retro-reaction that leads back to ketene and imine side products, which are starting substrates in the traditional Staudinger reaction. This retro-process has long been implicated in mechanistic studies of the classical Staudinger reaction but, to our knowledge, has never been experimentally validated.^44,51–53^ The partitioning of the zwitterionic intermediate between the cyclization and epoxidation pathways is observed in the r.r., which is dependent on the electronic nature of the 4-aryl substituent. A comparison between experimentally observed and calculated ΔΔG^‡^ for the cyclization and epoxidation TSs (for substrates **1h** and **1i**; Figure 6B) indicates that high selectivity is observed for ΔΔG^‡^calc > 2.2 kcal/mol, with correspondingly lower selectivity for smaller ΔΔG^‡^. We observed a strong correlation (Figure 6Ci, R^2^ = 0.98) between the Hammett σ+ constants and calculated ΔG^‡^ for the corresponding cyclizations to yield **2b**–**2i**.^43^ This strong linear free energy relationship (LFER) between the cyclization step (^**1**^**TS-III**) and the Hammett constant (with a slope of 5.92) is consistent with a significant reliance on charge stabilization, resulting in a notable variation in the cyclization rate.

This dependence is primarily observed due to the dissipation of negative charge from the carbon atom carrying the negative charge, a distinctive characteristic of the zwitterionic structure. Similarly, activation barriers for epoxide formation (which ultimately leads to the major observed by-product **7**) were also found to follow a Hammett behavior (Figure 6C, R^2^ = 0.92), albeit with a smaller dependency with a slope of 1.64. These results are consistent with our experimental observation that electron-poor arene substituents lead to lower yields of β-lactam product and increasing amounts of γ-lactam. Conversely, comparing the Gibbs free energy activation barrier for the retro-reaction (^**1**^**TS-V**) with the Hammett constants revealed a nearly horizontal dependency, with a slope of 0.30 (Figure 5Ci), illustrating that this pathway is practically indifferent to polar substituents. Overall, these results suggest that as the enolate becomes more electron rich, the 4π electrocyclization predominates over the competitive retro-reaction, with a change in the rate-determining step occurring at approximately a σ+ of 0.5, consistent with experimental Hammet-like correlation (Figure 3). This difference between reactivity trends of the 4π cyclization and retro-reaction as a function of substituent electronics may be rationalized in the context of the Hammond-Leffler postulate (Figure S23). The presence of electron-withdrawing groups stabilizes zwitterionic intermediate ^**1**^**C** relative to the spirocyclic electrocyclization product: the enolate negative charge can be delocalized onto the adjacent aryl substituent, whereas the aryl substituents are not conjugated in the resulting β-lactams **2b**–**2i**. This leads to less exergonic reactions and, consequently, later transition structures with increased reorganization energies. In contrast, ketene (^**1**^**G**) products generated from dissociation (via ^**1**^**TS-V**) possess conjugated π-systems capable of interacting with the aryl substituents. This means that delocalization onto adjacent aryl substituents bearing electron-withdrawing groups capable of stabilizing zwitterion ^**1**^**C** also stabilizes the ketene products, thus modulating any relative thermodynamic gain. Using the information, we were thus able to construct a selectivity prediction model by comparing the relative rates of the competitive pathways arising from intermediate ^**1**^**C**. The resulting predicted selectivity (Figure 6Cii) was found to possess a good Pearson correlation of R^2^ = 0.90 with the experimentally determined isolated yields.

An implication of this work is that the isolated yields of spirocyclic β-lactams for substrates bearing *para*-electron-withdrawing groups (such as **2i**) are lower due to a greater predominance of the competing retro-reaction (which may lead to other ketene-derived side products) and epoxidation-rearrangement pathways. In order to investigate this prediction experimentally, we designed a double-label crossover experiment with substrate **1i** and a *d*_*7*_ variant (Figure 6D). We reasoned that if this substrate was undergoing the retro-reaction as predicted, then we would generate *d*_*5*_ imine and d_*2*_ ketene, alongside unlabeled intermediates, which could recombine to afford *d*_*5*_- and *d*_*2*_-labeled crossover products (alongside the expected *d*_*7*_ and *d*_*0*_ products). A 1:1 mixture of substrate **1i** and **1i**-*d*_*7*_ was subjected to the standard reaction conditions to yield β-lactam products. Analysis of the β-lactam product mixture by liquid chromatography-mass spectrometry (LC-MS) showed a predominance of the expected **2i**-*d*_*7*_ and **2i**-*d*_*0*_ products alongside clear enrichment of the **2i**-*d*_*5*_ and **2i**-*d*_*2*_ crossover product peaks (circa 3% in each case vs. control experiments), which we attribute to reversible dissociation of the zwitterions to their component imine and ketene components and subsequent recombination (see Figures S8–S13 for a full analysis). This is consistent with the computational prediction of reversible dissociation of the zwitterion to ketene and imine and demonstrates that this could be a significant process in the Staudinger cyclization (for electronically biased substrates). We were unable to quantify the remaining ketene-derived by-products under our reaction conditions due to their inherent photochemical instability, as they are known to be highly photo-active and have low triplet energies.^54–56^

### Conclusion

We have demonstrated that an EnT-mediated synthesis of spirocyclic β-lactams proceeds with very high levels of diastereoselectivity via cyclization of a zwitterion rather than a diradical. This intermediate is directly analogous to the intermediate proposed for the classical Staudinger ketene-imine cyclization. Computational methods provide a rationale for the observed stereoselectivity and demonstrate that closed-shell zwitterionic and open-shell intermediates may be found on the same potential energy surface. These calculations also enable the prediction of a retro-reaction from the zwitterion intermediate back to imine and ketene substrates (which is also proposed in the Staudinger reaction); this is validated for the first time through a double-label crossover experiment. These findings indicate that visible light may offer an alternative route to generate and interrogate high-energy zwitterionic intermediates.

## METHODS

### General photochemical procedure

Ethyl acetate was sparged with argon for at least 30 min. The desired substrate **1** (1.0 equiv) and Ir(dFppy)_3_ (0.01 equiv) were dissolved in this degassed solvent (0.1 M substrate concentration) in a Schlenk tube and sealed while under an argon atmosphere. The resulting solution was then irradiated using a 36 W Kessil Blue LED lamp. After 16–40 h, the solvent was removed *in vacuo* and the product purified by flash column chromatography.

Further details regarding the methods can be found in the supplemental information.

## RESOURCE AVAILABILITY

### Lead contact

Requests for further information and resources should be directed to and will be fulfilled by the lead contact, Martin D. Smith (martin.smith@chem.ox.ac.uk).

### Materials availability

All other data supporting the findings of this study are available within the article and the supplemental information.

### Data and code availability

Generated computational data, including thermochemistry and cartesian coordinates, can be found in the supplemental information.

## Supplementary Material

supplementary information

NMR spectra

Supplemental information can be found online at https://doi.org/10.1016/j.checat.2025.101493.

## Figures and Tables

**Table 1.** Photoisomerization of substrate 1a

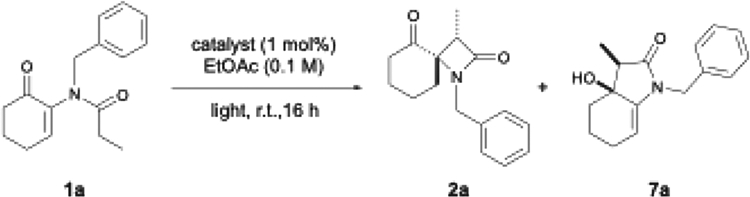				
Wavelength	Catalyst	Yield	2a (d.r.)	2a:7a (r.r.)
390 nm	-	55%	>20:1	10:1
440 nm	-	0%	N/A	N/A
440 nm	Ir(dFppy)_3_	69%	>20:1	10:1

## Highlights

- Intercepting the Staudinger **β**-lactam synthesis using triplet energy transfer
- Interplay between open-shell singlet biradicals and closed-shell zwitterions
- Diastereoselectivity dictated by T_1_ excited-state lifetimes
- First Staudinger **β**-lactam synthesis crossover experiment

## THE BIGGER PICTURE

The Staudinger ketene-imine cyclization is an effective method for the synthesis of **β**-lactams, but a complete understanding of its mechanism remains elusive. Here, we demonstrate that visible-light photocatalysis provides an alternative approach to accessing the postulated zwitterionic intermediate in the Staudinger cyclization without employing ketene and imine substrates. A combination of computational and experimental methods reveals a complex interplay between closed-shell zwitterions and open-shell biradical species, which has been previously overlooked. Confirmation of the presence of the zwitterion through crossover experiments validates a long-standing prediction of reversibility in its formation. This demonstrates that visible-light photocatalysis offers an alternative route to generate and interrogate high-energy zwitterionic intermediates.
