# Supplementary material for: Photocatalysis as a mechanistic probe for the Staudinger β-lactam synthesis
Source: Chem Catal. Author manuscript; Available in PMC 2026 May 15. (PMC13175146; doi:10.1016/j.checat.2025.101493)
Supplement: supplementary information [file NIHMS2173764-supplement-supplementary_information.pdf]

Chem Catalysis, Volume *5*

Supplemental information

Photocatalysis as a mechanistic probe for the Staudinger β-lactam synthesis

Mihai V. Popescu, Nicholas A. Parker, Zhuqing Jia, Pearse Solon, Callan J. Maloney, Juan V. Alegre-Requena, Robert S. Paton, and Martin D. Smith

Contents

General Information 2
General Procedures 4
Substrate Synthesis 7
Photocyclization 20
Crossover substrate synthesis 28
Mechanistic Investigations 34
Computational Studies 40
X-ray Crystallographic Data 61
References 64

General Information

Supplemental Methods:

All reagents and solvents were used as supplied commercially without further purification unless stated otherwise. Dry dichloromethane, tetrahydrofuran and diethyl ether were purified through activated alumina columns employing the method of Grubbs *et al*.[1] Water was purified using an Elix® UV-10 system. HPLC grade solvents for photochemical reactions were degassed by sparging the solvent with argon or nitrogen gas for at least 30 minutes. Petrol ether (PE) 40–60 refers to the fraction of petroleum ether which boils in the range 40–60 °C. Brine refers to a saturated aqueous solution of sodium chloride. Tris[2-(4,6-difluorophenyl)pyridinato-C^2,*N*]iridium (III) ($Ir(Fppy)_3$) was prepared according to literature procedure[2] or purchased from Sigma-Aldrich and used as received.

Photochemical reactions were performed using a Kessil H150 blue LED lamp. Optimization reactions were performed in sealed 7 mL screw top glass vials, whilst larger scale reactions were performed in sealed Schlenk tubes. In both cases the LED lamp was placed approximately 1 cm away from the edge of the reaction vessel. Cooling was provided by a stream of nitrogen, which kept the reaction at ambient temperature (approximately 25 °C).

Thin Layer Chromatography (TLC) was performed using pre-coated aluminium-backed Merck TLC Silica Gel 60 F_{254} plates and visualised using UV irradiation (λ = 254 nm) or staining with potassium permanganate solution. Flash column chromatography was performed with Merck Geduran® Si 60 Silicagel (40–63 µm particle size) or with Sigma-Aldrich Silica gel (technical grade, pore size 60 Å, 40–63 µm particle size). All solvents used for chromatographic purification were HPLC grade or equivalent and supplied by Sigma-Aldrich.

All Nuclear Magnetic Resonance (NMR) spectra were recorded on Bruker AVIIIHD 400 nanobay spectrometers at room temperature in solutions of $CDCl_3$ unless otherwise stated and the deuterated solvent acted as the deuterium lock. ^1H NMR spectra were recorded at 400 MHz, ^{13}C NMR spectra at 101 MHz with broadband proton decoupling and ^{19}F NMR spectra at 377 MHz. Residual protic solvent signal acted as an internal reference for ^1H NMR and the deuterated solvent carbon signal acted as an internal reference for ^{13}C NMR ($CDCl_3$: ^1H NMR = 7.26 ppm, ^{13}C NMR = 77.16 ppm). ^{19}F NMR spectra were reference externally to $CFCl_3$. Chemical shifts, δ, are given in parts per million (ppm) to the nearest 0.01 ppm for ^1H and ^{19}F and 0.1 ppm for ^{13}C NMR. The multiplicity of a signal is reported as such: s–singlet, d–doublet, t–triplet, q–quartet, quint–quintet, m–multiplet, br–broad, or combinations thereof. Coupling constants, J, are reported as observed in Hz. For inseparable mixtures of diastereomers, only peaks corresponding to the major diastereomer are reported. Additional 2D NMR experiments (COSY, HSQC, HMBC and NOESY) were used to assist in structural assignment.

Fourier-transform infrared (FT-IR) spectra were recorded from evaporated films on a Bruker Tensor 27 spectrometer equipped with a Pike Miracle Attenuated Total Reflectance (ATR) sampling accessory. Absorption maxima are quoted in wavenumbers, ν_{max}/cm^{-1}, for the range 3500–600 cm^{-1}.

High resolution mass spectrometry (HRMS) using electrospray ionization (ESI) was carried out on a Thermo Exactive orbitrap spectrometer equipped with a Waters Equity LC system. HRMS using chemical ionization (CI) or electron ionization (EI) was carried out on a Waters GCT system equipped with a Time of Flight (TOF) spectrometer. Low resolution mass spectrometry was carried out using ESI and was performed on a Micromass LCT Premier Spectrometer. In both cases the mass reported is that containing the most abundant isotopes.

Melting points were determined using a Reichert melting point apparatus or a Leica VMTG heated-stage microscope equipped with a Testo 720 thermometer and are reported uncorrected. All compounds were crystallised from chloroform ($CHCl_3$) unless stated otherwise.

Systematic names were generated using ChemDraw 16.0 in accordance with the guidelines specified by the International Union of Pure and Applied Chemistry (IUPAC).

General Procedures

Supplemental Methods:

General Procedure A: Epoxide opening with an amine

This process is based upon a modified literature procedure.[3] The specified amine (1.20 equiv.) and epoxide (1.00 equiv.) were added to a round-bottom flask fitted with a condenser. MeOH (1.80 mL per mmol epoxide) and H_2O (0.600 mL per mmol epoxide) were added as solvent and the resulting solution was heated to reflux (85 °C) for 4–22 h before being cooled to ambient temperature. The reaction mixture was poured into H_2O (10 mL per mmol epoxide) and extracted with Et_2O (3 × 10 mL per mmol epoxide). The combined organic extracts were washed with brine (5 mL per mmol epoxide), dried (Na_2SO_4), filtered and concentrated *in vacuo*. The crude product was then purified by flash column chromatography.

General Procedure B: *N*-Acylation of Enaminones (Acid chloride)

This process is based upon a modified literature procedure.[4] The specified enaminone (1.00 equiv.) was dissolved in CH_2Cl_2 (5 mL per mmol enaminone) and cooled to 0 °C. The desired acid chloride (1.10 equiv.) was then added dropwise, followed by Et_3N (1.10 equiv.). The mixture was then allowed to warm to ambient temperature and stirred for 12–23 h. The reaction mixture was then diluted with CH_2Cl_2 (5 mL per mmol enaminone) and then washed with H_2O (12.5 mL per mmol enaminone) and brine (12.5 mL per mmol enaminone). The organic phase was then dried (Na_2SO_4), filtered and concentrated *in vacuo*. The crude product was then purified by flash column chromatography.

General Procedure C: *N*-Acylation of Enaminones (Carboxylic acid)

To a solution of the specified carboxylic acid (1.10 equiv.) in dry CH_2Cl_2 (4.5 mL per mmol carboxylic acid) was added oxalyl chloride (2.20 equiv.) and DMF (10 µL) and the reaction stirred at ambient temperature for 1 h until effervescence stopped. The resulting solution was concentrated *in vacuo* and the crude residue dissolved in CH_2Cl_2 (2.5 mL per mmol carboxylic acid). This solution was added dropwise at 0 °C to a solution of the specified enaminone (1.00 equiv.) in CH_2Cl_2 (2.5 mL per mmol enaminone). Et_3N (1.10 equiv.) was then added dropwise and the reaction mixture warmed to ambient temperature and stirred for 16–21 h. The reaction mixture was diluted with CH_2Cl_2 (10 mL per mmol enaminone) and washed with H_2O (25 mL per mmol enaminone) and brine (25 mL per mmol enaminone). The organic phase was dried (Na_2SO_4), filtered and concentrated *in vacuo* and the crude product was purified by flash column chromatography.

General Procedure D: *N*-Acylation of Enaminones (Mukaiyama reagent coupling)

To a solution of the specified carboxylic acid (1.10 equiv.) in dry CH_2Cl_2 (2.5 mL per mmol carboxylic acid) under an argon atmosphere was added 2-chloro-1-methylpyridinium iodide (Mukaiyama's reagent; 2.20 equiv.). A solution of the specified enaminone (1.00 equiv.) in CH_2Cl_2 (2.5 mL per mmol enaminone) was then added to the reaction mixture, followed by the specified base (3.30 equiv.). The reaction mixture was then stirred in the dark for 19–22 h before being diluted with CH_2Cl_2 (10 mL per mmol enaminone) and washed with H_2O (20 mL per mmol enaminone) and brine (20 mL per mmol enaminone). The organic phase was then dried (Na_2SO_4), filtered and concentrated *in vacuo* and the crude product purified by flash column chromatography.

General Procedure E: *N*-Acylation of Enaminones (EDC coupling)

The specified enaminone (1.00 equiv.), the specified carboxylic acid (1.10 equiv.), Et_3N (2.00 equiv.), EDC (1.50 equiv.) and DMAP (0.100 equiv.) were dissolved in CH_2Cl_2 (10 mL per mmol enaminone) and the reaction mixture stirred at ambient temperature for 70 h. The reaction mixture was then diluted with H_2O (20 mL per mmol enaminone), the organic phase separated, and the aqueous phase

extracted with CH_2Cl_2 (2 × 10 mL per mmol enaminone). The combined organic extracts were then washed with brine (20 mL per mmol enaminone), dried (Na_2SO_4), filtered and concentrated *in vacuo* before being purified by flash column chromatography.

General Procedure F: Photochemical Spirocyclization

Ethyl acetate was sparged with argon for at least 30 minutes. The desired substrate (1.0 equiv.) and Ir(dFppy)$_3$ (0.01 equiv.) were dissolved in this degassed solvent (0.1 M substrate concentration) in a Schlenk tube and sealed whilst under an argon atmosphere. The resulting solution was then irradiated using a 36 W Kessil Blue LED lamp. After 16–40 h, the solvent was removed *in vacuo* and the product purified by flash column chromatography.

Expanded Substrate Investigations

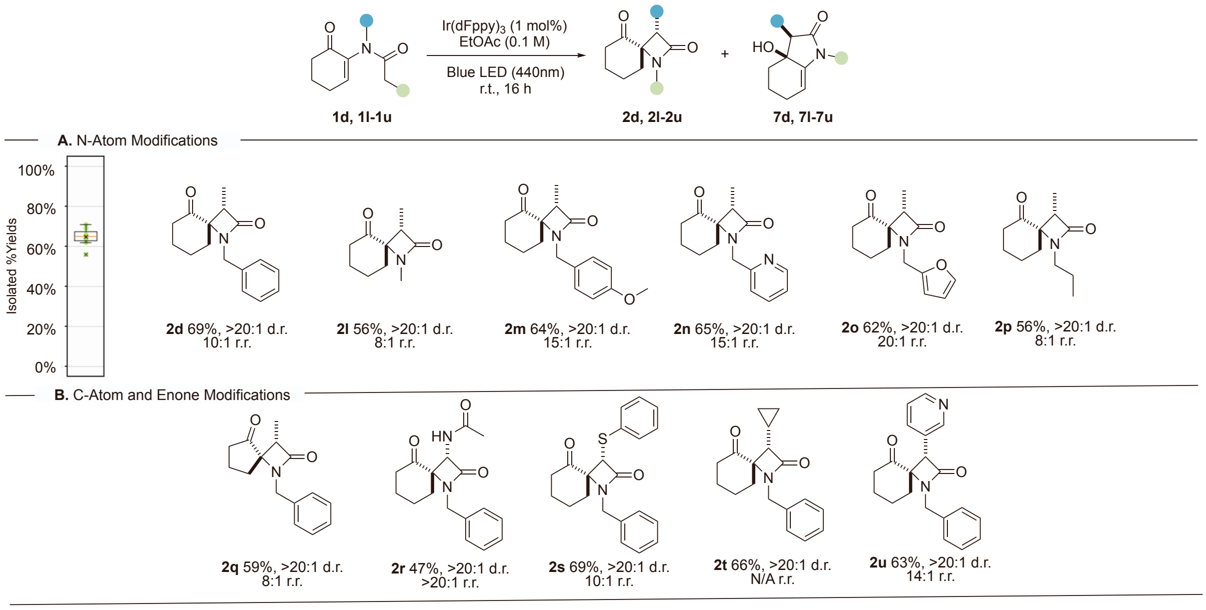

Figure S1: Expanded investigations into yields and selectivity changes as a function of (A) modifying the N-Atom substituent and (B) C-Atom and enone ring system, all of which show only, using General Procedure F. All reported yields are isolated.

Procedure F reaction optimization:

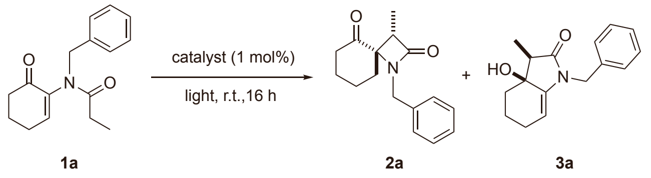

Entry	Wavelength	Catalyst	Solvent[(a)]	Concentration	Yield[(b)]	2a d.r.	2a : 3a r.r.
1	390 nm	-	EtOAc	0.1 M	55% (55%)[(c)]	>20:1	10:1
2	440 nm	-	EtOAc	0.1 M	0%	N/A	N/A
3	440 nm	Ir(dFppy)$_3$	EtOAc	0.1 M	72% (69%)[(c)]	>20:1	10:1
4	440 nm	Ir(dFppy)$_3$	EtOAc	0.05 M	71%	>20:1	10:1
5	440 nm	Ir(dFppy)$_3$	EtOAc	0.02 M	69%	>20:1	10:1
6	440 nm	Ir(dFppy)$_3$	EtOAc	0.01 M	71%	>20:1	10:1
7	440 nm	Ir(dFppy)$_3$	Toluene	0.1 M	70%	>20:1	10:1
8	440 nm	Ir(dFppy)$_3$	Acetone	0.1 M	59%	>20:1	10:1
9	440 nm	Ir(dFppy)$_3$	MeCN	0.1 M	42%	>20:1	10:1

Table S1. Reaction condition optimization; (a) NMR yields performed on 0.1 mmol scale using CH_2Br_2 as an internal standard; (b) Isolated yields performed on 0.3 mmol scale.

Screening of non-metal photosensitizers

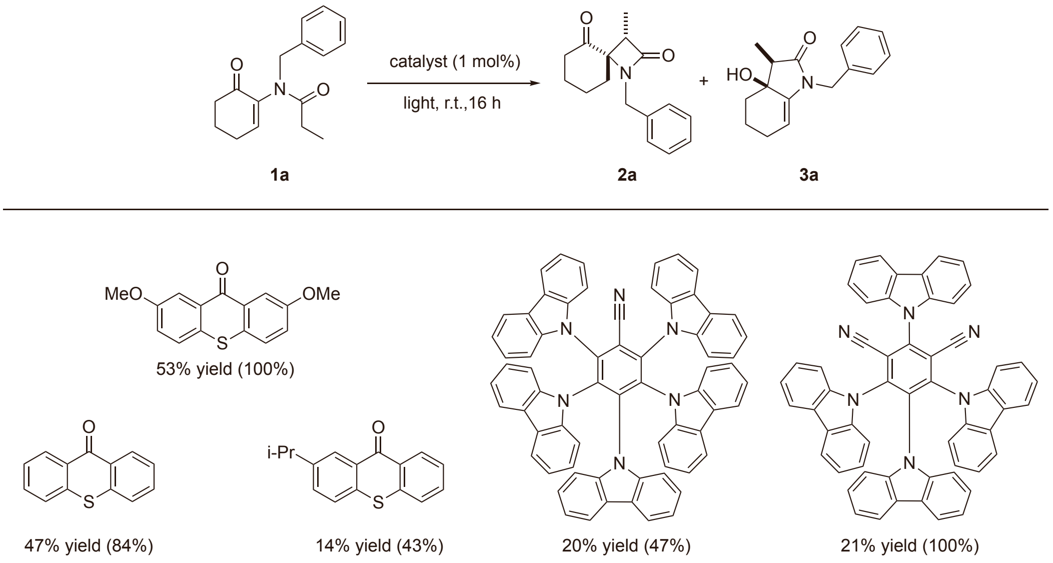

Figure S2. Reaction condition optimization; (a) isolated yields; parentheses indicate NMR conversions using CH_2Br_2 as an internal standard.

Substrate Synthesis

7-oxabicyclo[4.1.0]heptan-2-one (**8**)

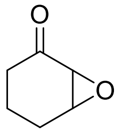

A solution of 2-cyclohexen-1-one (9.68 mL, 9.61 g, 100 mmol, 1.00 equiv.) in MeOH (100 mL, 1.00 M substrate concentration) was cooled to 0 °C and aqueous H_2O_2 (30%, 30.1 mL, 300 mmol, 3.00 equiv.) was added dropwise with stirring. Aqueous sodium hydroxide (3 M, 6.67 mL, 0.20 eq.) was then added dropwise and the reaction was stirred at 0 °C for 35 minutes until complete consumption of the starting material as monitored by TLC. The reaction was poured into a separating funnel containing ice (100 g) and brine (200 mL) and extracted with CH_2Cl_2 (3 × 100 mL). The combined organic extracts were dried (Na_2SO_4), filtered and concentrated *in vacuo*. The crude colourless oil of 7-oxabicyclo[4.1.0]heptan-2-one (9.18 g, 81.9 mmol, 82 % yield) was used directly without further purification in **General Procedure A**.

IR (neat) ν_{max}/cm^{-1} 2949, 1708, 1406, 1341, 1250, 970, 881, 817, 749.

^{1}H NMR ($CDCl_3$, 400 MHz) δ = 3.61–3.55 (m, 1H), 3.21 (*J* = 3.9 Hz, 1H), 2.61–2.46 (m, 1H), 2.29–2.23 (m, 1H), 2.16–2.00 (m, 1H), 2.00–1.84 (m, 2H), 1.73–1.62 (m, 1H).

^{13}C NMR ($CDCl_3$, 101 MHz) δ = 206.0, 55.9, 55.1, 36.4, 22.9, 17.0.

HRMS (CI) Found $[M+H]^+$ = 113.0600; $[C_6H_9O_2]^+$ requires 113.0603, Δ -2.91 ppm.

6-oxabicyclo[3.1.0]hexan-2-one (**9**)

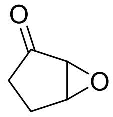

A solution of 2-cyclopenten-1-one (2.00 g, 24.4 mmol, 1.00 equiv.) in MeOH (25.0 mL, 1.00 M substrate concentration) was cooled to 0 °C and aqueous H_2O_2 (30%, 7.34 mL, 73.2 mmol, 3.00 equiv.) was added dropwise with stirring. Aqueous sodium hydroxide (3 M, 1.63 mL, 0.20 eq.) was then added dropwise and the reaction was stirred at 0 °C for 20 minutes until complete consumption of the starting material as monitored by TLC. The reaction was poured into a separating funnel containing ice (30 g) and brine (60 mL) and extracted with CH_2Cl_2 (3 × 30 mL). The combined organic extracts were dried (Na_2SO_4), filtered and concentrated *in vacuo*. The crude colourless oil of 6-oxabicyclo[3.1.0]hexan-2-one (1.48 g, 15.1 mmol, 62 % yield) was used directly without further purification in **General Procedure A**.

IR (film) ν_{max}/cm^{-1} 2942, 1743, 1448, 1410, 1371, 1176, 967, 840, 802.

^{1}H NMR ($CDCl_3$, 400 MHz) δ = 3.92 (m, 1H), 3.32 (d, *J* = 2.1 Hz, 1H), 2.45–2.23 (m, 2H), 2.14–2.01 (m, 2H).

^{13}C NMR ($CDCl_3$, 101 MHz) δ = 210.1, 57.9, 54.8, 30.5, 23.2.

HRMS (CI) Found $[M+NH_4]^+$ = 116.0705; $[C_5H_{10}NO_2]^+$ requires 116.0706, Δ 0.05 ppm.

2-(benzylamino)cyclohex-2-en-1-one (**10a**)

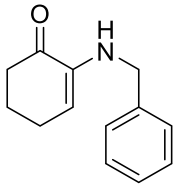

Prepared according to **General Procedure A** with 7-oxabicyclo[4.1.0]heptan-2-one () (1.12 g, 10.0 mmol, 1.00 equiv.) and benzylamine (1.31 mL, 1.29 g, 12.0 mmol, 1.20 equiv.) for 21 h. Purification by flash column chromatography (9:1→4:1 v/v Petrol ether 40–60:Et_2O) gave 2-(benzylamino)cyclohex-2-en-1-one (**10a**) as a yellow solid (1.42 g, 7.05 mmol, 71%).

m.p. = 56–58 °C.

IR (neat) ν_{max}/cm^{-1} = 3399, 3030, 2924, 2864, 2829, 1669, 1628, 1486, 1453, 1343, 1277, 1210, 1160, 1129, 1059, 1028, 970, 902, 868, 797, 740, 698.

^{1}H NMR ($CDCl_3$, 400 MHz) δ = 7.38–7.22 (m, 5H), 5.43 (t, J = 4.7 Hz, 1H), 4.61 (1H, br s), 4.09 (s, 2H), 2.49 (t, J = 6.6 Hz, 2H), 2.34 (q, J = 5.6 Hz, 2H), 1.96 (quint, J = 6.3 Hz, 2H).

^{13}C NMR ($CDCl_3$, 101 MHz) δ = 196.0, 140.5, 139.1, 128.6, 127.5, 127.2, 111.9, 47.7, 38.0, 24.6, 23.6.

HRMS (ESI^+) Found $[M+H]^+$ = 202.12259; $[C_{13}H_{16}ON]^+$ requires 202.12264, Δ -0.26 ppm.

2-(methylamino)cyclohex-2-en-1-one (**10l**)

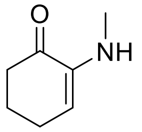

Prepared according to **General Procedure A** with 7-oxabicyclo[4.1.0]heptan-2-one (**8**) (1.12 g, 10.0 mmol, 1.00 equiv.) and 40 wt% methylamine aqueous solution (1.73 mL, 1.55 g, 20.0 mmol, 1.20 equiv.) for 19 h. Purification by flash column chromatography (19:1→4:1 v/v Petrol ether 40–60:Et_2O) gave 2-(methylamino)cyclohex-2-en-1-one (**10l**) as an orange liquid (457 mg, 3.65 mmol, 37%).

IR (neat) ν_{max}/cm^{-1} = 3406, 2922, 2827, 1668, 1627, 1489, 1441, 1425, 1339, 1280, 1203, 1176, 1151, 1128, 1074, 1039, 982, 886, 867, 795, 708.

^{1}H NMR ($CDCl_3$, 400 MHz) δ = 5.38 (t, J = 4.7 Hz, 1H), 4.14 (br s, 1H), 2.60 (s, 3H), 2.45 (t, J = 6.1 Hz, 2H), 2.37 (q, J = 5.6 Hz, 2H), 1.94 (quint, J = 6.2 Hz, 2H).

^{13}C NMR ($CDCl_3$, 101 MHz) δ = 196.1, 141.8, 110.9, 38.1, 29.9, 24.6, 23.7.

HRMS (ESI^+) Found $[M+H]^+$ = 126.09125; $[C_7H_{12}ON]^+$ requires 126.09134, Δ -0.71 ppm.

2-((4-methoxybenzyl)amino)cyclohex-2-en-1-one (**10m**)

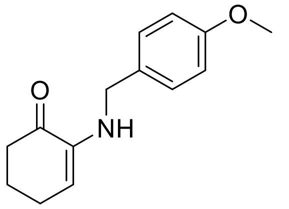

Prepared according to **General Procedure A** with 7-oxabicyclo[4.1.0]heptan-2-one (**8**) (673 mg, 6.00 mmol, 1.00 equiv.) and 4-methoxybenzylamine (0.941 mL, 988 mg, 7.20 mmol, 1.20 equiv.) for 22 h. Purification by flash column chromatography (9:1→4:1 v/v Petrol ether 40–60:Et_2O) gave 2-((4-methoxybenzyl)amino)cyclohex-2-en-1-one (**10m**) as an off-white solid (568 mg, 2.45 mmol, 41%).

m.p. = 56–58°C (*n*-hexane).

IR (neat) ν_{max}/cm^{-1} = 3403, 2917, 2832, 1662, 1623, 1612, 1585, 1509, 1495, 1467, 1453, 1433, 1416, 1356, 1341, 1283, 1246, 1213, 1179, 1168, 1159, 1126, 1108, 1087, 1068, 1032, 1011, 976, 928, 887, 869, 810, 797, 743, 721, 711, 698, 624.

^{1}H NMR ($CDCl_3$, 400 MHz) δ = 7.25–7.18 (m, 2H), 6.90–6.82 (m, 2H), 5.44 (t, J = 4.7 Hz, 1H), 4.51 (br s, 1H), 4.00 (d, J = 4.7 Hz, 2H), 3.80 (s, 3H), 2.52–2.44 (m, 2H), 2.39–2.30 (m, 2H), 2.00–1.89 (m, 2H).

^{13}C NMR ($CDCl_3$, 101 MHz) δ = 196.0, 158.9, 140.6, 131.2, 128.8, 114.1, 111.8, 55.4, 47.2, 38.1, 24.7, 23.6.

HRMS (ESI^+) Found $[M+H]^+$ = 232.13338; $[C_{14}H_{18}O_2N]^+$ requires 232.13321, Δ 0.74 ppm.

2-((Pyridin-2-ylmethyl)amino)cyclohex-2-en-1-one (**10n**)

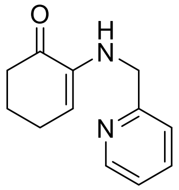

This reaction is based on a modified literature procedure.[4] To a solution of 1,2-cyclohexanedione (561 mg, 5.00 mmol, 1.00 equiv.) in toluene (50 mL) was added 2-picolylamine (0.567 mL, 595 mg, 5.50 mmol, 1.10 equiv.), and the resulting solution was heated to reflux in a Dean-Stark water separator for 5 h. After cooling to ambient temperature, the reaction mixture was washed with H_2O (50 mL), brine (50 mL), dried (Na_2SO_4), filtered and concentrated *In vacuo*. Purification by flash column chromatography (2:1→1:1 v/v Petrol ether 40–60:EtOAc) gave 2-((pyridin-2-ylmethyl)amino)cyclohex-2-en-1-one (**10n**) as an orange oil (406 mg, 2.01 mmol, 40%).

IR (neat) ν_{max}/cm^{-1} = 3051, 2938, 2865, 1669, 1627, 1591, 1571, 1474, 1456, 1435, 1398, 1341, 1277, 1197, 1162, 1130, 1099, 1048, 995, 914, 869, 755, 727, 642, 621.

^{1}H NMR ($CDCl_3$, 400 MHz) δ = 8.59–8.51 (m, 1H), 7.63 (td, *J* = 7.7, 1.8 Hz, 1H), 7.31–7.23 (m, 1H), 7.19–7.11 (m, 1H), 5.39 (t, *J* = 4.7 Hz, 1H), 5.01 (br s, 1H), 4.23 (s, 2H), 2.49 (t, *J* = 6.8 Hz, 2H), 2.31 (q, *J* = 5.5 Hz, 2H), 1.93 (quint, *J* = 6.3 Hz, 2H).

^{13}C NMR ($CDCl_3$, 101 MHz) δ = 195.9, 158.9, 149.4, 140.4, 136.7, 122.1, 121.3, 112.2, 49.2, 38.1, 24.6, 23.6.

HRMS (ESI$^+$) Found $[M+H]^+$ = 203.11801; $[C_{12}H_{15}ON_2]^+$ requires 201.11789, Δ 0.6 ppm.

2-((Furan-2-ylmethyl)amino)cyclohex-2-en-1-one (**10o**)

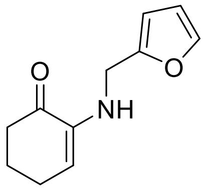

Prepared according to **General Procedure A** with 7-oxabicyclo[4.1.0]heptan-2-one (**8**) (336 mg, 3.00 mmol, 1.00 equiv.) and 2-(aminomethyl)furan (0.318 mL, 350 mg, 3.60 mmol, 1.20 equiv.) for 20 h. Purification by flash column chromatography (9:1→4:1 v/v Petrol ether 40–60:Et_2O) gave 2-((furan-2-ylmethyl)amino)cyclohex-2-en-1-one (**10o**) as a yellow oil (414 mg, 2.17 mmol, 72%).

IR (neat) ν_{max}/cm^{-1} = 3398, 2927, 1669, 1630, 1485, 1455, 1340, 1277, 1196, 1161, 1129, 1074, 1012, 919, 884, 869, 801, 736.

^{1}H NMR ($CDCl_3$, 400 MHz) δ = 7.37–7.32 (m, 1H), 6.34–6.28 (m, 1H), 6.21–6.15 (m, 1H), 5.54 (t, *J* = 4.7 Hz, 1H), 4.54 (br s, 1H), 4.09–4.03 (s, 2H), 2.48 (t, *J* = 6.5 Hz, 2H), 2.36 (q, *J* = 5.6 Hz, 2H), 1.95 (quint, *J* = 6.1 Hz, 2H).

^{13}C NMR ($CDCl_3$, 101 MHz) δ = 195.8, 152.5, 142.0, 140.3, 112.3, 110.4, 107.0, 40.8, 38.0, 24.6, 23.5.

HRMS (ESI$^+$) Found $[M+H]^+$ = 192.10188; $[C_{11}H_{14}O_2N]^+$ requires 192.10191, Δ -0.12 ppm.

2-(Propylamino)cyclohex-2-en-1-one (**10p**)

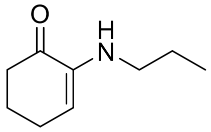

Prepared according to **General Procedure A** with 7-oxabicyclo[4.1.0]heptan-2-one (**8**) (561 mg, 5.00 mmol, 1.00 equiv.) and propylamine (0.617 mL, 443 mg, 7.50 mmol, 1.20 equiv.) for 4 h. Purification by flash column chromatography (19:1 v/v Petrol ether 40–60:EtOAc) gave 2-(propylamine)cyclohex-2-en-1-one (**10p**) as a yellow oil (285 mg, 1.86 mmol, 37%).

IR (neat) ν_{max}/cm^{-1} = 3399, 2931, 2871, 2829, 1672, 1627, 1489, 1456, 1416, 1383, 1340, 1275, 1245, 1201, 1167, 1127, 1078, 1046, 999, 984, 897, 867, 793, 757, 709.

^{1}H NMR ($CDCl_3$, 400 MHz) δ = 5.41 (t, J = 4.7 Hz, 1H), 4.10 (s, 1H), 2.78 (t, J = 7.1 Hz, 2H), 2.46 (t, J = 6.7 Hz, 2H), 2.36 (q, J = 5.6 Hz, 2H), 1.93 (quint, J = 6.3 Hz, 2H), 1.56 (quint, J = 7.3 Hz, 2H), 0.94 (t, J = 7.3 Hz, 3H).

^{13}C NMR ($CDCl_3$, 101 MHz) δ = 196.1, 140.8, 111.0, 45.2, 38.1, 24.7, 23.7, 22.3, 11.9.

HRMS (ESI^+) Found $[M+H]^+$ = 154.12260; $[C_9H_{16}ON]^+$ requires 154.12264, Δ -0.24 ppm.

2-(benzylamino)cyclopent-2-en-1-one (10q)

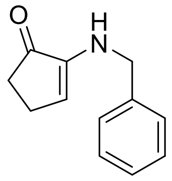

Prepared according to **General Procedure A** with 6-oxabicyclo[3.1.0]hexan-2-one (**9**) (245 mg, 2.50 mmol, 1.00 equiv.) and benzylamine (0.328 ml, 322 mg, 3.00 mmol, 1.20 equiv.) for 4 h. Purification by flash column chromatography (9:1 v/v Petrol ether 40–60:Et_2O) afforded 2-(benzylamino)cyclopent-2-en-1-one (**10q**) as a yellow solid (381 mg, 2.03 mmol, 81%).

m.p. = 85–87 °C.

IR (neat) ν_{max}/cm^{-1} = 3377, 3062, 2920, 1693, 1635, 1493, 1453, 1405, 1361, 1326, 1288, 1213, 1141, 1028, 992, 772, 735, 698.

^{1}H NMR ($CDCl_3$, 400 MHz) δ = 7.31–7.15 (m, 5H), 5.83 (t, J = 3.1 Hz, 1H), 4.20 (br s, 1H), 4.13 (s, 2H), 2.43–2.38 (m, 2H), 2.36–2.30 (m, 2H).

^{13}C NMR ($CDCl_3$, 101 MHz) δ = 204.8, 146.2, 138.7, 128.7, 127.4, 127.4, 122.3, 48.7, 33.7, 23.7.

HRMS (ESI^+) Found $[M+H]^+$ = 188.10714; $[C_{12}H_{14}ON]^+$ requires 188.10699, Δ 0.77 ppm.

***N*-benzyl-*N*-(6-oxocyclohex-1-en-1-yl)propionamide (1a)**

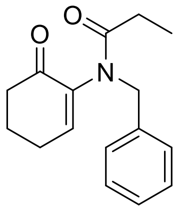

Prepared according to **General Procedure B** with 2-(benzylamino)cyclohex-2-en-1-one (**10a**) (805 mg, 4.00 mmol, 1.00 equiv.), propionyl chloride (0.384 mL, 407 mg, 4.40 mmol, 1.10 equiv.), Et_3N (0.613 mL, 445 mg, 4.40 mmol, 1.10 equiv.) and CH_2Cl_2 (20 mL) for 18 h. Purification by flash column chromatography (3:2→1:1 v/v Petrol ether 40–60:EtOAc) gave *N*-benzyl-*N*-(6-oxocyclohex-1-en-1-yl)propionamide (**1a**) as an off-white solid (964 mg, 3.74 mmol, 94%).

m.p. = 55–57 °C.

IR (neat) ν_{max}/cm^{-1} = 3030, 2976, 2938, 2875, 1683, 1658, 1630, 1585, 1495, 1455, 1397, 1362, 1345, 1263, 1227, 1205, 1189, 1153, 1124, 1076, 1029, 1006, 976, 915, 888, 812, 756, 722, 702, 662, 618.

^{1}H NMR ($CDCl_3$, 400 MHz) δ = 7.30–7.20 (m, 3H), 7.20–7.14 (m, 2H), 6.48 (t, J = 4.3 Hz, 1H), 5.34 (d, J = 14.5 Hz, 1H), 3.88 (d, J = 14.5 Hz, 1H), 2.57–2.43 (m, 2H), 2.41–2.26 (m, 2H), 2.23–2.09 (m, 1H), 2.06–1.89 (m, 3H), 1.08 (t, J = 7.4 Hz, 3H).

^{13}C NMR ($CDCl_3$, 101 MHz) δ = 195.2, 173.8, 149.6, 139.0, 137.8, 129.1, 128.4, 127.4, 50.7, 38.6, 27.2, 26.0, 22.5, 9.7.

HRMS (ESI^+) Found $[M+Na]^+$ = 280.13074; $[C_{16}H_{19}O_2NNa]^+$ requires 280.13080, Δ -0.22 ppm.

N-benzyl-N-(6-oxocyclohex-1-en-1-yl)propionamide-2,2-d_2 (1a-d_2)

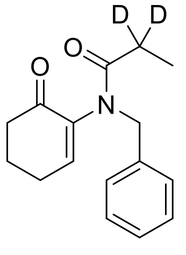

Prepared according to **General Procedure D** with 2-(benzylamino)cyclohex-2-en-1-one (**10a**) (302 mg, 1.50 mmol, 1.00 equiv.), propionic acid-d_2 (0.123 mL, 126 mg, 1.65 mmol, 1.10 equiv.), 2-chloro-1-methylpyridinium iodide (843 mg, 3.30 mmol, 2.20 equiv.), pyridine (0.400 mL, 392 mg, 4.95 mmol, 3.30 equiv.) and CH_2Cl_2 (7.5 mL) for 19 h. Purification by flash column chromatography (3:1→1:1 v/v Petrol ether 40–60:EtOAc) gave *N*-benzyl-*N*-(6-oxocyclohex-1-en-1-yl)propionamide-2,2-d_2 (**1a-d_2**) as a yellow oil (138 mg, 0.53 mmol, 35%).

IR (neat) ν_{max}/cm^{-1} = 2937, 1684, 1654, 1496, 1454, 1389, 1154, 1124, 1079, 1029, 977, 918, 854, 701.

^{1}H NMR ($CDCl_3$, 400 MHz) δ = 7.29–7.20 (m, 3H), 7.20–7.13 (m, 2H), 6.48 (t, J = 4.3 Hz, 1H), 5.33 (d, J = 14.5 Hz, 1H), 3.88 (d, J = 14.5 Hz, 1H), 2.56–2.40 (m, 2H), 2.40–2.22 (m, 2H), 2.06–1.87 (m, 2H), 1.06 (s, 3H).

^{13}C NMR ($CDCl_3$, 101 MHz) δ = 195.2, 173.8, 149.6, 139.0, 137.7, 129.0, 128.4, 127.4, 50.6, 38.6, 26.0, 25.9, 22.5, 9.5.

HRMS (ESI$^+$) Found $[M+H]^+$ = 260.16156; $[C_{16}H_{18}{}^2H_2O_2N]^+$ requires 260.16141, Δ 0.58 ppm.

N-benzyl-2-(4-methoxyphenyl)-N-(6-oxocyclohex-1-en-1-yl)acetamide (1b)

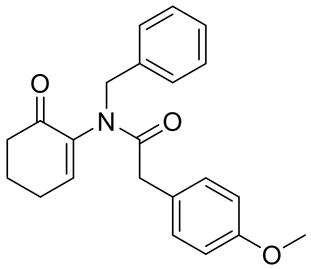

Prepared according to **General Procedure C** with 4-methoxyphenylacetic acid (274 mg, 1.65 mmol, 1.10 equiv.), oxalyl chloride (0.283 mL, 418 mg, 3.30 mmol, 2.20 equiv.), DMF (100 µL) and CH_2Cl_2 (5 mL) followed by 2-(benzylamino)cyclohex-2-en-1-one (**10a**) (302 mg, 1.50 mmol, 1.00 equiv.), Et_3N (0.230 mL, 167 mg, 1.65 mmol, 1.10 equiv.) and CH_2Cl_2 (7.5 mL) for 21 h. Purification by flash column chromatography (4:1→2:1 v/v Petrol ether 40–60:EtOAc) gave *N*-benzyl-2-(4-methoxyphenyl)-*N*-(6-oxocyclohex-1-en-1-yl)acetamide (**1b**) as an off-white solid (283 mg, 0.810 mmol, 54%).

m.p. = 55–57 °C.

IR (neat) ν_{max}/cm^{-1} = 2934, 1684, 1656, 1631, 1612, 1584, 1511, 1454, 1425, 1395, 1343, 1301, 1246, 1177, 1152, 1123, 1080, 1032, 1006, 977, 918, 814, 783, 758, 723, 702.

^{1}H NMR ($CDCl_3$, 400 MHz) δ = 7.30–7.20 (m, 3H), 7.20–7.13 (m, 2H), 7.10 (d, J = 8.1 Hz, 2H), 6.82 (d, J = 8.5 Hz, 2H), 6.25 (t, J = 4.3 Hz, 1H), 5.38 (d, J = 14.5 Hz, 1H), 3.89 (d, J = 14.5 Hz, 1H), 3.77 (s, 3H), 3.49 (d, J = 14.9 Hz, 1H), 3.36 (d, J = 15.0 Hz, 1H), 2.53–2.40 (m, 2H), 2.33–2.18 (m, 2H), 2.05–1.88 (m, 2H).

^{13}C NMR ($CDCl_3$, 101 MHz) δ = 195.1, 171.2, 158.5, 150.4, 138.5, 137.6, 130.0, 129.1, 128.5, 127.7, 127.5, 114.0, 55.4, 50.8, 40.7, 38.5, 26.0, 22.5.

HRMS (ESI$^+$) Found $[M+H]^+$ = 350.17514; $[C_{22}H_{24}O_3N]^+$ requires 350.17507, Δ 0.20 ppm.

N-benzyl--(6-oxocyclohex-1-en-1-yl)-2-(p-tolyl)acetamide (1c)

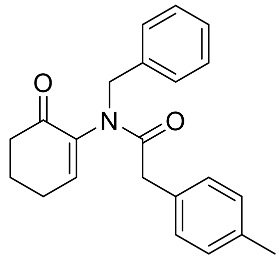

Prepared according to **General Procedure C** with *p*-tolylacetic acid (248 mg, 1.65 mmol, 1.10 equiv.), oxalyl chloride (0.283 mL, 418 mg, 3.30 mmol, 2.20 equiv.), DMF (100 µL) and CH_2Cl_2 (5 mL) followed by 2-(benzylamino)cyclohex-2-en-1-one (**10a**) (302 mg, 1.50 mmol, 1.00 equiv.), Et_3N (0.230 mL, 167 mg, 1.65 mmol, 1.10 equiv.) and CH_2Cl_2 (7.5 mL) for 17 h. Purification by flash column chromatography (4:1→2:1 v/v Petrol ether 40–60:EtOAc) gave *N*-benzyl-*N*-(6-oxocyclohex-1-en-1-yl)-2-(p-tolyl)acetamide (**1c**) as an off-white solid (203 mg, 0.610 mmol, 41%).

m.p. = 68–70 °C.

IR (neat) ν_{max}/cm^{-1} = 3029, 2924, 1685, 1656, 1631, 1515, 1496, 1454, 1416, 1394, 1361, 1343, 1263, 1234, 1191, 1152, 1123, 1080, 1028, 1006, 977, 919, 803, 778, 757, 722, 702.

^{1}H NMR ($CDCl_3$, 400 MHz) *δ* = 7.29–7.21 (m, 3H), 7.19–7.15 (m, 2H), 7.12–7.03 (m, 4H), 6.23 (t, *J* = 4.2 Hz, 1H), 5.39 (d, *J* = 14.4 Hz, 1H), 3.90 (d, *J* = 14.5 Hz, 1H), 3.52 (d, *J* = 14.9 Hz, 1H), 3.38 (d, *J* = 15.0 Hz, 1H), 2.47–2.41 (m, 2H), 2.30 (s, 3H), 2.27–2.20 (m, 2H), 2.01–1.90 (m, 2H).

^{13}C NMR ($CDCl_3$, 101 MHz) *δ* = 195.1, 171.1, 150.5, 138.5, 137.6, 136.3, 132.6, 129.3, 129.1, 128.8, 128.4, 127.4, 50.7, 41.3, 38.5, 26.0, 22.4, 21.2.

HRMS (ESI^+) Found $[M+H]^+$ = 334.18048; $[C_{22}H_{24}O_2N]^+$ requires 334.18016, Δ 0.97 ppm.

N-benzyl-N-(6-oxocyclohex-1-en-1-yl)-2-phenylacetamide (1d)

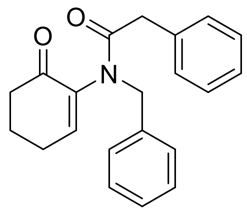

Prepared according to **General Procedure B** with 2-(benzylamino)cyclohex-2-en-1-one (**10a**) (403 mg, 2.00 mmol, 1.00 equiv.), phenylacetyl chloride (0.290 mL, 340 mg, 2.20 mmol, 1.10 equiv.), Et_3N (0.310 mL, 223 mg, 2.20 mmol, 1.10 equiv.) and CH_2Cl_2 (10 mL) for 12 h. Purification by flash column chromatography (1:1→1:2 v/v Petrol ether 40–60:Et_2O) gave *N*-benzyl-*N*-(6-oxocyclohex-1-en-1-yl)-2-phenylacetamide (**1d**) as an off–white solid (469 mg, 1.47 mmol, 73%).

m.p. = 109–111 °C.

IR (neat) ν_{max}/cm^{-1} = 3029, 2931, 1684, 1656, 1632, 1603, 1584, 1495, 1454, 1396, 1361, 1344, 1263, 1233, 1191, 1152, 1123, 1080, 1005, 977, 912, 846, 721, 701.

^{1}H NMR ($CDCl_3$, 400 MHz) *δ* = 7.32–7.06 (m, 10H), 6.15 (t, *J* = 4.3 Hz, 1H), 5.32 (d, *J* = 14.4 Hz, 1H), 3.83 (d, *J* = 14.5 Hz, 1H), 3.50 (d, *J* = 15.0 Hz, 1H), 3.36 (d, *J* = 15.0 Hz, 1H), 2.36 (t, *J* = 6.8 Hz, 2H), 2.23–2.12 (m, 2H), 1.97–1.78 (m, 2H).

^{13}C NMR ($CDCl_3$, 101 MHz) *δ* = 195.0, 170.8, 150.5, 138.4, 137.4, 135.6, 129.0, 128.9, 128.5, 128.4, 127.4, 126.7, 50.7, 41.6, 38.4, 25.9, 22.3.

HRMS (ESI^+) Found $[M+H]^+$ = 320.16437; $[C_{21}H_{22}O_2N]^+$ requires 320.16451, Δ -0.43 ppm.

N-benzyl-2-(4-fluorophenyl)-N-(6-oxocyclohex-1-en-1-yl)acetamide (**1e**)

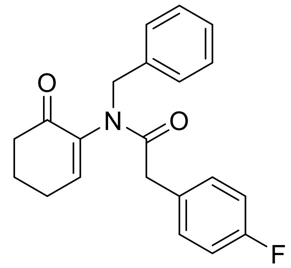

Prepared according to **General Procedure B** with 2-(benzylamino)cyclohex-2-en-1-one (**10a**) (302 mg, 1.50 mmol, 1.00 equiv.), 4-fluorophenylacetyl chloride (0.226 mL, 285 mg, 1.65 mmol, 1.10 equiv.), Et_3N (0.230 mL, 167 mg, 1.65 mmol, 1.10 equiv.) and CH_2Cl_2 (7.5 mL) for 18 h. Purification by flash column chromatography (5:1→2:1 v/v Petrol ether 40–60:EtOAc) gave *N*-benzyl-2-(4-fluorophenyl)-*N*-(6-oxocyclohex-1-en-1-yl)acetamide (**1e**) as an off-white solid (348 mg, 1.03 mmol, 69%).

m.p. = 96–98 °C.

IR (neat) ν_{max}/cm^{-1} = 2932, 1685, 1658, 1631, 1605, 1509, 1396, 1344, 1263, 1222, 1157, 1123, 1080, 1006, 817, 723, 702.

^{1}H NMR ($CDCl_3$, 400 MHz) δ = 7.28–7.23 (m, 3H), 7.19–7.12 (m, 4H), 6.97 (t, *J* = 8.6 Hz, 2H), 6.31 (t, *J* = 4.3 Hz, 1H), 5.36 (d, *J* = 14.4 Hz, 1H), 3.92 (d, *J* = 14.5 Hz, 1H), 3.49 (d, *J* = 15.1 Hz, 1H), 3.40 (d, *J* = 15.2 Hz, 1H), 2.51–2.42 (m, 2H), 2.33–2.24 (m, 2H), 2.01–1.92 (m, 2H).

^{13}C NMR ($CDCl_3$, 101 MHz) δ = 195.1, 170.8, 161.9 (d, *J* = 245.0 Hz), 150.3, 138.7, 137.5, 131.3 (d, *J* = 3.1 Hz), 130.7 (d, *J* = 7.9 Hz) 129.2, 128.5, 127.6, 115.4 (d, *J* = 21.5 Hz), 51.0, 40.6, 38.5, 26.1, 22.5.

^{19}F NMR ($CDCl_3$, 377 MHz) δ = -116.20 (tt, *J* = 8.9, 5.3 Hz).

HRMS (ESI$^+$) Found $[M+H]^+$ = 338.15512; $[C_{21}H_{21}O_2NF]^+$ requires 338.15508, Δ 0.11 ppm.

N-benzyl-2-(4-bromophenyl)-N-(6-oxocyclohex-1-en-1-yl)acetamide (**1f**)

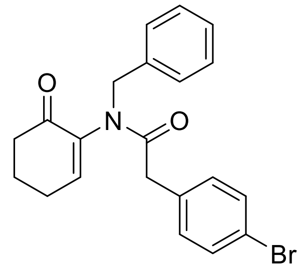

Prepared according to **General Procedure C** with 4-bromophenylacetic acid (237 mg, 1.10 mmol, 1.10 equiv.), oxalyl chloride (0.189 mL, 279 mg, 2.20 mmol, 2.20 equiv.), DMF (100 µL) and CH_2Cl_2 (5 mL) followed by 2-(benzylamino)cyclohex-2-en-1-one (**10a**) (201 mg, 1.00 mmol, 1.00 equiv.), Et_3N (0.153 mL, 111 mg, 1.10 mmol, 1.10 equiv.) and CH_2Cl_2 (7.5 mL) for 18 h. Purification by flash column chromatography (4:1→2:1 v/v Petrol ether 40–60:EtOAc) gave *N*-benzyl-2-(4-bromophenyl)-*N*-(6-oxocyclohex-1-en-1-yl)acetamide (**1f**) as an off-white solid (177 mg, 0.444 mmol, 44%).

m.p. = 94–96 °C.

IR (neat) ν_{max}/cm^{-1} = 3030, 2929, 1684, 1658, 1632, 1488, 1454, 1394, 1344, 1262, 1232, 1198, 1152, 1123, 1071, 1012, 977, 919, 801, 722, 702, 613.

^{1}H NMR ($CDCl_3$, 400 MHz) δ = 7.38 (d, *J* = 8.0 Hz, 2H), 7.26–7.21 (m, 3H), 7.16–7.12 (m, 2H), 7.04 (d, *J* = 8.0 Hz, 2H), 6.29 (t, *J* = 4.2 Hz, 1H), 5.32 (d, *J* = 14.4 Hz, 1H), 3.90 (d, *J* = 14.4 Hz, 1H), 3.44 (d, *J* = 15.2 Hz, 1H), 3.35 (d, *J* = 15.2 Hz, 1H), 2.47–2.41 (m, 2H), 2.30–2.23 (m, 2H), 1.98–1.89 (m, 2H).

^{13}C NMR ($CDCl_3$, 101 MHz) δ = 195.1, 170.4, 150.4, 138.6, 137.4, 134.6, 131.7, 130.9, 129.2, 128.5, 127.6, 120.8, 51.0, 40.8, 38.5, 26.0, 22.5.

HRMS (ESI$^+$) Found $[M+H]^+$ = 398.07513; $[C_{21}H_{21}O_2NBr]^+$ requires 398.07502, Δ 0.29 ppm.

_N_-benzyl-2-(3-fluorophenyl)-_N_-(6-oxocyclohex-1-en-1-yl)acetamide (**1g**)

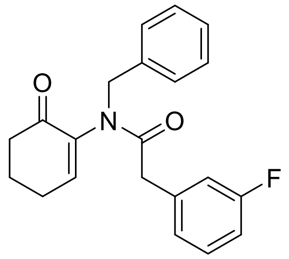

Prepared according to **General Procedure C** with 3-fluorophenylacetic acid (170 mg, 1.10 mmol, 1.10 equiv.), oxalyl chloride (0.189 mL, 280 mg, 2.20 mmol, 2.20 equiv.), DMF (100 µL) and CH_2Cl_2 (5 mL) followed by 2-(benzylamino)cyclohex-2-en-1-one (**10a**) (201 mg, 1.00 mmol, 1.00 equiv.), Et_3N (0.153 mL, 111 mg, 1.10 mmol, 1.10 equiv.) and CH_2Cl_2 (10 mL) for 18 h. Purification by flash column chromatography (4:1→2:1 v/v Petrol ether 40–60:EtOAc) gave *N*-benzyl-2-(3-fluorophenyl)-*N*-(6-oxocyclohex-1-en-1-yl)acetamide (**1g**) as an off-white solid (153 mg, 0.455 mmol, 45%).

m.p. = 86–88 °C.

IR (neat) ν_{max}/cm^{-1} = 3031, 2930, 1685, 1657, 1632, 1616, 1589, 1488, 1449, 1428, 1396, 1361, 1344, 1265, 1190, 1152, 1124, 1080, 1029, 1004, 977, 955, 912, 875, 838, 757, 722, 702, 685.

^{1}H NMR ($CDCl_3$, 400 MHz) δ = 7.37–7.29 (m, 4H), 7.28–7.22 (m, 2H), 7.09–6.94 (m, 3H), 6.38 (t, *J* = 4.3 Hz, 1H), 5.43 (d, *J* = 14.4 Hz, 1H), 4.02 (d, *J* = 14.4 Hz, 1H), 3.61 (d, *J* = 15.1 Hz, 1H), 3.50 (d, *J* = 15.2 Hz, 1H), 2.58–2.51 (m, 2H), 2.40–2.32 (m, 2H), 2.09–2.00 (m, 2H).

^{13}C NMR ($CDCl_3$, 101 MHz) δ = 195.0, 170.3, 162.9 (d, *J* = 245.6 Hz), 150.4, 138.6, 138.1 (d, *J* = 7.8 Hz), 137.4, 130.0 (d, *J* = 8.6 Hz), 129.2, 128.5, 127.6, 124.8 (d, *J* = 3.0 Hz), 116.2 (d, *J* = 21.6 Hz), 113.8 (d, *J* = 21.2 Hz), 51.0, 41.2, 38.5, 26.0, 22.5.

^{19}F NMR ($CDCl_3$, 377 MHz) δ = -113.22 (td, *J* = 9.4, 6.0 Hz).

HRMS (ESI$^+$) Found $[M+Na]^+$ = 360.13708; $[C_{21}H_{20}O_2NFNa]^+$ requires 360.13703, Δ 0.16 ppm.

_N_-benzyl-_N_-(6-oxocyclohex-1-en-1-yl)-2-(4-(trifluoromethyl)phenyl)acetamide (**1h**)

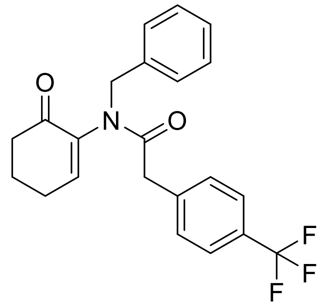

Prepared according to **General Procedure C** with 4-(trifluoromethyl)phenylacetic acid (337 mg, 1.65 mmol, 1.70 equiv.), oxalyl chloride (0.283 mL, 418 mg, 3.30 mmol, 3.40 equiv.), DMF (100 µL) and CH_2Cl_2 (5 mL) followed by 2-(benzylamino)cyclohex-2-en-1-one (**10a**) (192 mg, 0.950 mmol, 1.00 equiv.), Et_3N (0.230 mL, 167 mg, 1.65 mmol, 1.70 equiv.) and CH_2Cl_2 (7.5 mL) for 16 h. Purification by flash column chromatography (4:1→2:1 v/v Petrol ether 40–60:EtOAc) gave *N*-benzyl-*N*-(6-oxocyclohex-1-en-1-yl)-2-(4-(trifluoromethyl)phenyl)aceta-mide (**1h**) as an off-white solid (196 mg, 0.507 mmol, 53%).

m.p. = 89–91 °C.

IR (neat) ν_{max}/cm^{-1} = 2934, 1656, 1660, 1632, 1496, 1421, 1396, 1324, 1263, 1161, 1120, 1067, 1020, 977, 921, 818, 758, 702.

^{1}H NMR ($CDCl_3$, 400 MHz) δ = 7.48 (d, *J* = 7.9 Hz, 2H), 7.25 (d, *J* = 7.9 Hz, 2H), 7.22–7.15 (m, 3H), 7.11 (d, *J* = 7.0 Hz, 2H), 6.30 (t, *J* = 4.4 Hz, 1H), 5.27 (d, *J* = 14.4 Hz, 1H), 3.89 (d, *J* = 14.4 Hz, 1H), 3.49 (d, *J* = 15.3 Hz, 1H), 3.42 (d, *J* = 15.4 Hz, 1H), 2.48–2.37 (m, 2H), 2.32–2.21 (m, 2H), 1.98–1.86 (m, 2H).

^{13}C NMR ($CDCl_3$, 101 MHz) δ = 195.1, 170.2, 150.4, 139.7, 138.6, 137.3, 129.6, 129.2, 128.6, 127.7, 125.5 (q, *J* = 3.7 Hz), 123.0, 51.1, 41.0, 38.5, 26.0, 22.5.

^{19}F NMR ($CDCl_3$, 377 MHz) δ = -62.44 (s)

HRMS (ESI^+) Found $[M+H]^+$ = 388.15189; $[C_{22}H_{21}O_2NF_3]^+$ requires 388.15189, Δ -0.01 ppm.

***N*-benzyl-2-(4-cyanophenyl)-*N*-(6-oxocyclohex-1-en-1-yl)acetamide** (**1i**)

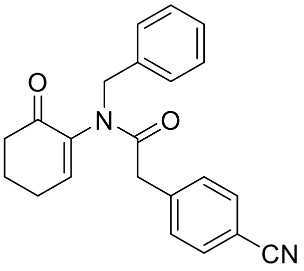

Prepared according to **General Procedure C** with (4-cyanophenyl)acetic acid (266 mg, 1.65 mmol, 1.10 equiv.), oxalyl chloride (0.279 mL, 418 mg, 3.30 mmol, 2.20 equiv.), DMF (100 µL) and CH_2Cl_2 (5 mL) followed by 2-(benzylamino)cyclohex-2-en-1-one (**10a**) (302 mg, 1.50 mmol, 1.00 equiv.), pyridine (0.130 mL, 133 mg, 1.65 mmol, 1.10 equiv.) and CH_2Cl_2 (7.5 mL) for 18 h. Purification by flash column chromatography (2:1→1:1 v/v Petrol ether 40–60:EtOAc) gave *N*-benzyl-2-(4-cyanophenyl)-*N*-(6-oxocyclohex-1-en-1-yl)acetamide (**1i**) as an off-white solid (370 mg, 1.07 mmol, 72%).

m.p. = 111–113 °C (EtOAc).

IR (neat) ν_{max}/cm^{-1} = 3032, 2946, 2227, 1683, 1656, 1631, 1607, 1496, 1454, 1417, 1395, 1361, 1344, 1263, 1232, 1197, 1153, 1123, 1080, 1023, 1005, 977, 919, 871, 815, 758, 722, 703, 666, 618.

^{1}H NMR ($CDCl_3$, 400 MHz) δ = 7.53 (d, *J* = 8.1 Hz, 2H), 7.28 (d, *J* = 8.1 Hz, 2H), 7.24–7.17 (m, 3H), 7.15–7.09 (m, 2H), 6.37 (t, *J* = 4.4 Hz, 1H), 5.25 (d, *J* = 14.4 Hz, 1H), 3.92 (d, *J* = 14.4 Hz, 1H), 3.49 (d, *J* = 15.3 Hz, 1H), 3.41 (d, *J* = 15.3 Hz, 1H), 2.46–2.40 (m, 2H), 2.32–2.25 (m, 2H), 1.96–1.88 (m, 2H).

^{13}C NMR ($CDCl_3$, 101 MHz) δ = 194.9, 169.7, 150.3, 141.1, 138.7, 137.1, 132.3, 130.2, 129.2, 128.6, 127.7, 118.9, 110.8, 51.2, 41.0, 38.4, 26.0, 22.4.

HRMS (ESI^+) Found $[M+H]^+$ = 345.15985; $[C_{22}H_{21}O_2N_2]^+$ requires 345.15975, Δ 0.28 ppm.

***N*-benzyl-2-methoxy-*N*-(6-oxocyclohex-1-en-1-yl)acetamide** (**1j**)

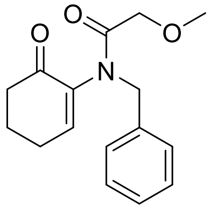

Prepared according to **General Procedure B** with 2-(benzylamino)cyclohex-2-en-1-one (**10a**) (201 mg, 1.00 mmol, 1.00 equiv.), methoxyacetyl chloride (0.101 mL, 119 mg, 1.10 mmol, 1.10 equiv.), Et_3N (0.153 mL, 111 mg, 1.10 mmol, 1.10 equiv.) and CH_2Cl_2 (5 mL) for 19 h. Purification by flash column chromatography (1:3 v/v Petrol ether 40–60:EtOAc) gave *N*-benzyl-2-methoxy-*N*-(6-oxocyclohex-1-en-1-yl)acetamide (**1j**) as a yellow oil (245 mg, 0.896 mmol, 90%).

IR (neat) ν_{max}/cm^{-1} = 2929, 1683, 1631, 1496, 1453, 1404, 1340, 1269, 1195, 1154, 1124, 1080, 1005, 976, 933, 839, 722, 702.

^{1}H NMR ($CDCl_3$, 400 MHz) δ = 7.33–7.16 (m, 5H), 6.46 (t, *J* = 4.3 Hz, 1H), 5.36 (d, *J* = 14.5 Hz, 1H), 3.95 (d, *J* = 13.9 Hz, 2H), 3.80 (d, *J* = 14.6 Hz, 1H), 3.34 (s, 3H), 2.58–2.41 (m, 2H), 2.41–2.22 (m, 2H), 2.06–1.90 (m, 2H).

^{13}C NMR ($CDCl_3$, 101 MHz) δ = 194.9, 169.3, 148.5, 137.7, 137.1, 129.1, 128.5, 127.6, 71.1, 59.1, 50.9, 38.4, 26.0, 22.4.

HRMS (ESI^+) Found $[M+H]^+$ = 274.14351; $[C_{16}H_{20}O_3N]^+$ requires 274.14377, Δ -0.94 ppm.

***N*-Benzyl-2-cyano-*N*-(6-oxocyclohex-1-en-1-yl)acetamide (1k)**

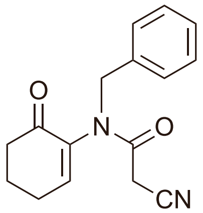

Prepared according to **General Procedure C** with cyanoacetic acid (140 mg, 1.65 mmol, 2.20 equiv.), oxalyl chloride (0.283 mL, 419 mg, 3.30 mmol), DMF (100 µL) and CH_2Cl_2 (5 mL) followed by 2-(benzylamino)cyclohex-2-en-1-one (**10a**) (151 mg, 0.750 mmol, 1.00 equiv.), pyridine (0.133 mL, 131 mg, 1.65 mmol, 2.20 equiv.) and CH_2Cl_2 (7.5 mL) for 17 h. Purification by flash column chromatography (3:2→1:1 v/v Petrol ether 40–60:EtOAc) gave *N*-benzyl-2-cyano-*N*-(6-oxocyclohex-1-en-1-yl)acetamide (**1k**) as a yellow oil (184 mg, 0.683 mmol, 46%).

IR (neat) ν_{max}/cm^{-1} = 2927, 2259, 1668, 1632, 1496, 1409, 1347, 1259, 1229, 1189, 1154, 1125, 1081, 1005, 977, 949, 916, 841, 757, 721, 702, 671, 619.

^{1}H NMR ($CDCl_3$, 400 MHz) δ = 7.34–7.20 (3H, m, C10H, C11H & C12H), 7.20–7.13 (2H, m, C9H & C13H), 6.69 (1H, t, *J* = 4.3 Hz, C4H), 5.17 (1H, d, *J* = 14.4 Hz, C7H_AH_B), 4.09 (1H, d, *J* = 14.4 Hz, C7H_AH_B), 3.32 (1H, d, *J* = 18.0 Hz, C15H_AH_B), 3.25 (1H, d, *J* = 18.0 Hz, C15H_AH_B), 2.59–2.44 (2H, m, C1H_2), 2.44–2.27 (2H, m, C3H_2), 2.07–1.90 (2H, m, C2H_2).

^{13}C NMR ($CDCl_3$, 101 MHz) δ = 194.8 (C6), 162.0 (C14), 151.9 (C4H), 137.6 (C5), 136.1 (C8), 129.2 (C9H & C13H), 128.7 (C10H & C12H), 128.0 (C11H), 114.4 (C16), 51.9 (C7H_2), 38.2 (C1H_2), 26.1 (C3H_2), 25.3 (C15H_2), 22.2 (C2H_2).

HRMS (ESI+) Found $[M+Na]^+$ = 291.1106; $[C_{16}H_{16}O_2N_2Na]^+$ requires 291.1104, Δ 0.65 ppm.

***N*-methyl-*N*-(6-oxocyclohex-1-en-1-yl)propionamide (1l)**

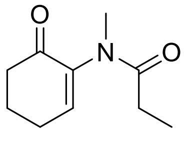

Prepared according to **General Procedure B** with 2-(methylamino)cyclohex-2-en-1-one (**10l**) (188 mg, 1.50 mmol, 1.00 equiv.), propionyl chloride (0.144 mL, 153 mg, 1.65 mmol, 1.10 equiv.), Et_3N (0.230 mL, 167 mg, 1.65 mmol, 1.10 equiv.) and CH_2Cl_2 (7.5 mL) for 19 h. Purification by flash column chromatography (1:1→0:1 v/v Petrol ether 40–60:EtOAc) gave *N*-methyl-*N*-(6-oxocyclohex-1-en-1-yl)propionamide (**1l**) as a yellow oil (207 mg, 1.14 mmol, 76%).

IR (neat) ν_{max}/cm^{-1} = 2922, 1682, 1656, 1631, 1462, 1422, 1383, 1353, 1311, 1277, 1224, 1162, 1130, 1112, 1072, 976, 914, 875, 814, 722.

^{1}H NMR ($CDCl_3$, 400 MHz) δ = 6.91 (t, *J* = 4.2 Hz, 1H), 2.96 (s, 3H), 2.53–2.48 (m, 4H), 2.10–2.01 (m, 3H), 2.00–1.88 (m, 1H), 1.02 (t, *J* = 7.5 Hz, 3H).

^{13}C NMR ($CDCl_3$, 101 MHz) δ = 195.1, 173.0, 147.7, 141.6, 38.5, 35.9, 26.8, 26.0, 22.6, 9.7.

HRMS (ESI$^+$) Found $[M+Na]^+$ = 204.09961; $[C_{10}H_{15}O_2NNa]^+$ requires 204.09950, Δ 0.54 ppm.

***N*-(4-methoxybenzyl)-*N*-(6-oxocyclohex-1-en-1-yl)propionamide (1m)**

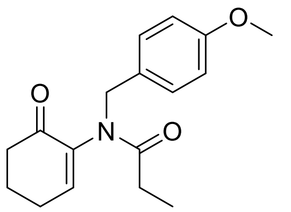

Prepared according to **General Procedure B** with 2-((4-methoxybenzyl)amino)cyclohex-2-en-1-one (**10m**) (463 mg, 2.00 mmol, 1.00 equiv.), propionyl chloride (0.192 mL, 204 mg, 2.20 mmol, 1.10 equiv.), Et_3N (0.307 mL, 223 mg, 2.20 mmol, 1.10 equiv.) and CH_2Cl_2 (10 mL) for 23 h. Purification by flash column chromatography (4:1→1:1 v/v Petrol ether 40–60:EtOAc) gave *N*-(4-methoxybenzyl)-*N*-(6-oxocyclohex-1-en-1-yl)propionamide (**1m**) as a yellow oil (546 mg, 1.90 mmol, 95%).

IR (neat) ν_{max}/cm^{-1} = 2937, 2836, 1683, 1656, 1629, 1612, 1585, 1511, 1461, 1399, 1363, 1343, 1301, 1246, 1176, 1153, 1123, 1111, 1088, 1031, 977, 911, 895, 849, 817, 758, 726, 661.

^{1}H NMR ($CDCl_3$, 400 MHz) δ = 7.13–7.04 (m, 2H), 6.81–6.73 (m, 2H), 6.44 (t, *J* = 4.2 Hz, 1H), 5.24 (d, *J* = 14.3 Hz, 1H), 3.82 (d, *J* = 14.3 Hz, 1H), 3.75 (s, 3H), 2.54–2.41 (m, 2H), 2.38–2.26 (m, 2H), 2.20–2.06 (m, 1H), 2.03–1.91 (m, 3H), 1.05 (t, *J* = 7.4 Hz, 3H).

^{13}C NMR ($CDCl_3$, 101 MHz) δ = 195.2, 173.6, 158.9, 149.6, 138.9, 130.4, 129.8, 113.7, 55.3, 50.0, 38.5, 27.1, 26.0, 22.5, 9.6.

HRMS (ESI^+) Found $[M+Na]^+$ = 310.14148; $[C_{17}H_{21}O_3NNa]^+$ requires 310.14136, Δ 0.37 ppm.

***N*-(6-oxocyclohex-1-en-1-yl)-*N*-(pyridin-2-ylmethyl)propionamide** (**1n**)

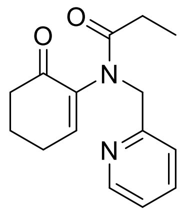

Prepared according to **General Procedure B** with 2-((pyridin-2-ylmethyl)amino)cyclohex-2-en-1-one (**10n**) (303 mg, 1.50 mmol, 1.00 equiv.), propionyl chloride (0.144 mL, 153 mg, 1.65 mmol, 1.10 equiv.), Et_3N (0.230 mL, 167 mg, 1.65 mmol, 1.10 equiv.) and CH_2Cl_2 (7.5 mL) for 20 h. Purification by flash column chromatography (97:3→94:6 v/v CH_2Cl_2:MeOH) gave *N*-(6-oxocyclohex-1-en-1-yl)-*N*-(pyridine-2-ylmethyl)propionamide (**1n**) as an orange oil (361 mg, 1.40 mmol, 93%).

IR (neat) ν_{max}/cm^{-1} = 2938, 2876, 1685, 1663, 1633, 1591, 1571, 1474, 1461, 1435, 1395, 1346, 1273, 1236, 1221, 1193, 1155, 1125, 1087, 1037, 1007, 995, 978, 906, 812, 757, 723, 667, 636, 615.

^{1}H NMR ($CDCl_3$, 400 MHz) δ = 8.46 (d, *J* = 4.8 Hz, 1H), 7.62 (td, *J* = 7.7, 1.8 Hz, 1H), 7.41 (d, *J* = 7.8 Hz, 1H), 7.14 (t, *J* = 7.8, 4.6 Hz, 1H), 6.84 (t, *J* = 4.3 Hz, 1H), 5.14 (d, *J* = 15.0 Hz, 1H), 4.34 (d, *J* = 15.0 Hz, 1H), 2.56–2.47 (m, 2H), 2.47–2.39 (m, 2H), 2.23–2.04 (m, 2H), 2.04–1.96 (m, 2H), 1.08 (t, *J* = 7.4 Hz, 3H).

^{13}C NMR ($CDCl_3$, 101 MHz) δ = 195.1, 174.1, 157.8, 149.1, 148.9, 140.1, 136.8, 123.5, 122.3, 53.6, 38.5, 27.0, 26.1, 22.5, 9.6.

HRMS (ESI^+) Found $[M+H]^+$ = 259.1446; $[C_{15}H_{19}N_2O_2]^+$ requires 259.1447, Δ -0.40 ppm.

***N*-(furan-2-ylmethyl)-*N*-(6-oxocyclohex-1-en-1-yl)propionamide** (**1o**)

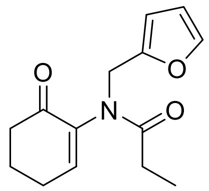

Prepared according to **General Procedure B** with 2-((furan-2-ylmethyl)amino)cyclohex-2-en-1-one (**10o**) (287 mg, 1.50 mmol, 1.00 equiv.), propionyl chloride (0.144 mL, 153 mg, 1.65 mmol, 1.10 equiv.), Et_3N (0.230 mL, 167 mg, 1.65 mmol, 1.10 equiv.) and CH_2Cl_2 (7.5 mL) for 19 h. Purification by flash column chromatography (3:2→1:1 v/v Petrol ether 40–60:EtOAc) gave *N*-(furan-2-ylmethyl)-*N*-(6-oxocyclohex-1-en-1-yl)propionamide (**1o**) as a yellow oil (357 mg, 1.45 mmol, 96%).

IR (neat) ν_{max}/cm^{-1} = 2939, 1683, 1661, 1633, 1504, 1399, 1257, 1214, 1178, 1150, 1124, 1074, 1013, 976, 923, 885, 811, 751.

^{1}H NMR ($CDCl_3$, 400 MHz) δ = 7.28 (d, J = 1.8 Hz, 1H), 6.63 (t, J = 4.3 Hz, 1H), 6.24 (dd, J = 3.2, 1.9 Hz, 1H), 6.14 (d, J = 3.2 Hz, 1H), 5.19 (d, J = 15.2 Hz, 1H), 4.02 (d, J = 15.2 Hz, 1H), 2.50–2.44 (m, 2H), 2.43–2.29 (m, 2H), 2.13 (dq, J = 15.0, 7.4 Hz, 1H), 2.03–1.89 (m, 3H), 1.03 (t, J = 7.4 Hz, 3H).

^{13}C NMR ($CDCl_3$, 101 MHz) δ = 195.1, 173.6, 151.1, 149.7, 142.2, 138.8, 110.4, 109.3, 43.3, 38.4, 27.1, 26.1, 22.4, 9.5.

HRMS (ESI^+) Found $[M+H]^+$ = 248.12796; $[C_{14}H_{18}O_3N]^+$ requires 248.12812, Δ -0.64 ppm.

***N*-(6-oxocyclohex-1-en-1-yl)-*N*-propylpropionamide (1p)**

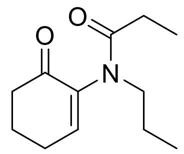

Prepared according to **General Procedure B** with 2-(propylamino)cyclohex-2-en-1-one (**10p**) (153 mg, 1.00 mmol, 1.00 equiv.), propionyl chloride (0.096 mL, 102 mg, 1.10 mmol, 1.10 equiv.), Et_3N (0.153 mL, 111 mg, 1.10 mmol, 1.10 equiv.) and CH_2Cl_2 (5 mL) for 15 h. Purification by flash column chromatography (2:1→1:1 v/v Petrol ether 40–60:EtOAc) gave *N*-(6-oxocyclohex-1-en-1-yl)-*N*-propylpropionamide (**1p**) as a yellow oil (140 mg, 0.667 mmol, 67%).

IR (neat) ν_{max}/cm^{-1} = 2962, 2936, 2874, 1684, 1657, 1629, 1461, 1402, 1375, 1345, 1264, 1237, 1210, 1134, 1120, 1081, 1020, 976, 942, 912, 889, 874, 813, 778, 723, 665.

^{1}H NMR ($CDCl_3$, 400 MHz) δ = 6.85 (t, J = 4.3 Hz, 1H), 3.63 (dt, J = 14.1, 7.4 Hz, 1H), 3.02 (dt, J = 14.3, 7.4 Hz, 1H), 2.54–2.49 (m, 4H), 2.09–2.01 (m, 3H), 1.90 (dq, J = 15.0, 7.3 Hz, 1H), 1.39 (q, J = 7.5 Hz, 2H), 1.00 (t, J = 7.4 Hz, 3H), 0.81 (t, J = 7.4 Hz, 3H).

^{13}C NMR ($CDCl_3$, 101 MHz) δ = 195.4, 173.6, 148.3, 140.1, 49.3, 38.6, 27.1, 26.1, 22.6, 21.3, 11.3, 9.6.

HRMS (ESI^+) Found $[M+H]^+$ = 210.14862; $[C_{12}H_{20}O_2N]^+$ requires 210.14886, Δ -1.12 ppm.

***N*-Benzyl-*N*-(5-oxocyclopent-1-en-1-yl)propionamide (1q)**

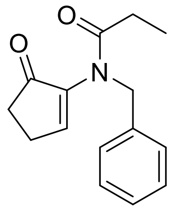

Prepared according to **General Procedure B** with 2-(benzylamino)cyclopent-2-en-1-one (**10q**) (375 mg, 2.00 mmol, 1.00 equiv.), propionyl chloride (0.192 mL, 204 mg, 2.20 mmol, 1.10 equiv.)), Et_3N (0.307 mL, 223 mg, 2.20 mmol, 1.10 equiv.) and CH_2Cl_2 (10 mL) for 19 h. Purification by flash column chromatography (1:1→1:2 v/v Petrol ether 40–60:EtOAc) gave *N*-benzyl-*N*-(5-oxocyclopent-1-en-1-yl)propionamide (**1q**) as a yellow oil (332 mg, 1.36 mmol, 68%).

IR (neat) ν_{max}/cm^{-1} = 3063, 3031, 2978, 2878, 1712, 1662, 1625, 1496, 1455, 1435, 1394, 1359, 1301, 1268, 1224, 1135, 1075, 1030, 1015, 1001, 973, 926, 882, 850, 811, 788, 736, 702, 669.

^{1}H NMR ($CDCl_3$, 400 MHz) δ = 7.31–7.13 (m, 6H), 4.74 (s, 2H), 2.60–2.52 (m, 2H), 2.45–2.38 (m, 2H), 2.21 (q, J = 7.3 Hz, 2H), 1.10 (t, J = 7.4 Hz, 3H).

^{13}C NMR ($CDCl_3$, 101 MHz) δ = 204.3, 173.5, 158.2, 144.8, 137.5, 128.5, 128.0, 127.4, 50.4, 33.5, 27.2, 24.6, 9.5.

HRMS (ESI^+) Found $[M+H]^+$ = 244.13324; $[C_{15}H_{18}O_2N]^+$ requires 244.13321, Δ 0.14 ppm.

2-acetamido-*N*-benzyl-*N*-(6-oxocyclohex-1-en-1-yl)acetamide (1r)

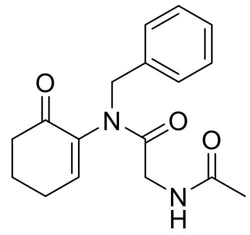

Prepared according to **General Procedure D** with 2-(benzylamino)cyclohex-2-en-1-one (**10a**) (201 mg, 1.00 mmol, 1.00 equiv.), *N*-acetylglycine (129 mg, 1.10 mmol, 1.10 equiv.), 2-chloro-1-methylpyridinium iodide (562 mg, 2.20 mmol, 2.20 equiv.), Et_3N (0.360 mL, 261 mg, 3.30 mmol, 3.30 equiv.) and CH_2Cl_2 (10 mL) for 22 h. Purification by flash column chromatography (100:0→85:15 v/v EtOAc:MeOH) gave 2-acetamido-*N*-benzyl-*N*-(6-oxocyclohex-1-en-1-yl)acetamide (**1r**) as a yellow oil (187 mg, 0.621 mmol, 62%).

IR (neat) ν_{max}/cm^{-1} = 3460, 2935, 2456, 1652, 1580, 1542, 1412, 1363, 1268, 1233, 1190, 1155, 1125, 1006, 977, 877, 840, 762, 721, 703.

^{1}H NMR ($CDCl_3$, 400 MHz) δ = 7.32–7.25 (m, 3H), 7.19–7.14 (m, 2H), 6.57 (t, *J* = 4.3 Hz, 1H), 6.51 (s, 1H), 5.36 (d, *J* = 14.4 Hz, 1H), 4.07 (dd, *J* = 17.6, 5.1 Hz, 1H), 3.91 (d, *J* = 14.5 Hz, 1H), 3.50 (dd, *J* = 17.6, 3.3 Hz, 1H), 2.59–2.41 (m, 3H), 2.36–2.24 (m, 1H), 2.12–2.03 (m, 1H), 2.02 (s, 3H), 1.99–1.88 (m, 1H).

^{13}C NMR ($CDCl_3$, 101 MHz) δ = 194.9, 170.1, 168.5, 151.5, 137.0, 136.6, 129.1, 128.7, 127.9, 51.2, 41.6, 38.4, 26.1, 23.2, 22.3.

HRMS (ESI$^+$) Found $[M+Na]^+$ = 323.13668; $[C_{17}H_{20}O_3N_2Na]^+$ requires 323.13661, Δ 0.22 ppm.

***N*-benzyl-*N*-(6-oxocyclohex-1-en-1-yl)-2-(phenylthiol)acetamide** (**1s**)

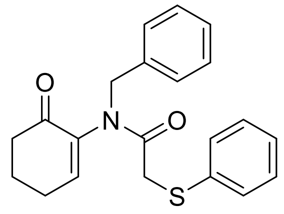

Prepared according to **General Procedure B** with 2-(benzylamino)cyclohex-2-en-1-one (**10a**) (201 mg, 1.00 mmol, 1.00 equiv.), (phenylthiol)acetyl chloride (0.163 mL, 205 mg, 1.10 mmol, 1.10 equiv.), Et_3N (0.153 mL, 111 mg, 1.10 mmol, 1.10 equiv.) and CH_2Cl_2 (10 mL) for 18 h. Purification by flash column chromatography (4:1→3:1 v/v Petrol ether 40–60:EtOAc) gave *N*-benzyl-*N*-(6-oxocyclohex-1-en-1-yl)-2-(phenylthiol)acetamide (**1s**) as an orange oil (269 mg, 0.764 mmol, 76%).

IR (neat) ν_{max}/cm^{-1} = 3030, 2926, 1682, 1656, 1629, 1583, 1495, 1481, 1454, 1438, 1391, 1360, 1342, 1292, 1217, 1188, 1152, 1123, 1079, 1026, 1004, 976, 907, 835, 740, 721, 692, 665, 616.

^{1}H NMR ($CDCl_3$, 400 MHz) δ = 7.41–7.34 (m, 2H), 7.28–7.11 (m, 8H), 6.34 (t, *J* = 4.3 Hz, 1H), 5.31 (d, *J* = 14.5 Hz, 1H), 3.90 (d, *J* = 14.5 Hz, 1H), 3.53 (d, *J* = 14.1 Hz, 1H), 3.47 (d, *J* = 14.2 Hz, 1H), 2.52–2.37 (m, 2H), 2.30–2.18 (m, 2H), 1.94–1.86 (m, 2H).

^{13}C NMR ($CDCl_3$, 101 MHz) δ = 195.0, 168.7, 150.4, 138.1, 137.0, 135.3, 130.4, 129.0, 128.4, 127.5, 127.0, 51.1, 38.4, 37.3, 25.9, 22.2.

HRMS (ESI$^+$) Found $[M+H]^+$ = 352.13658; $[C_{21}H_{22}O_2NS]^+$ requires 352.13658, Δ 0.01 ppm.

N-benzyl-2-cyclopropyl-N-(6-oxocyclohex-1-en-1-yl)acetamide (1t)

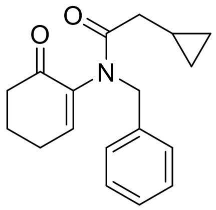

Prepared according to **General Procedure C** with cyclopropylacetic acid (110 mg, 1.10 mmol, 1.10 equiv.), oxalyl chloride (0.186 mL, 279 mg, 2.20 mmol, 2.20 equiv.), DMF (100 μL) and CH_2Cl_2 (5 mL) followed by 2-(benzylamino)cyclohex-2-en-1-one (**10a**) (201 mg, 1.00 mmol, 1.00 equiv.), Et_3N (0.153 mL, 111 mg, 1.10 mmol, 1.10 equiv.) and CH_2Cl_2 (10 mL) for 21 h. Purification by flash column chromatography (3:1→2:1 v/v Petrol ether 40–60:EtOAc) gave *N*-benzyl-2-cyclopropyl-*N*-(6-oxocyclohex-1-en-1-yl)acetamide (**1t**) as a yellow oil (121 mg, 0.425 mmol, 43%).

IR (neat) ν_{max}/cm^{-1} = 2930, 1685, 1659, 1630, 1495, 1400, 1342, 1260, 1224, 1189, 1154, 1122, 1079, 1018, 977, 840, 721, 702.

^{1}H NMR ($CDCl_3$, 400 MHz) δ = 7.32–7.16 (m, 5H), 6.48 (t, *J* = 4.4 Hz, 1H), 5.37 (d, *J* = 14.5 Hz, 1H), 3.90 (d, *J* = 14.5 Hz, 1H), 2.55–2.30 (m, 4H), 2.11–1.87 (m, 4H), 1.10–0.97 (m, 1H), 0.52 (d, *J* = 8.3 Hz, 2H), 0.17–0.00 (m, 2H).

^{13}C NMR ($CDCl_3$, 101 MHz) δ = 195.2, 172.7, 149.6, 139.0, 137.8, 129.2, 128.5, 127.5, 50.7, 39.1, 38.6, 26.0, 22.5, 7.6, 4.7, 4.5.

HRMS (ESI$^+$) Found $[M+Na]^+$ = 306.14642; $[C_{18}H_{21}O_2NNa]^+$ requires 306.14645, Δ -0.09 ppm.

N-benzyl-N-(6-oxocyclohex-1-en-1-yl)-2(pyridin-3-yl)acetamide (1u)

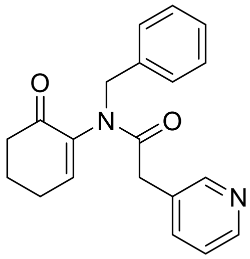

Prepared according to **General Procedure E** with 2-(benzylamino)cyclohex-2-en-1-one (**10a**) (201 mg, 1.00 mmol, 1.00equiv.), 3-pyridylacetic acid hydrochloride salt (191 mg, 1.10 mmol, 1.10 equiv.) Et_3N (0.220 mL, 158 mg, 1.10 mmol, 1.10 equiv.), EDC (288 mg, 1.50 mmol, 1.50 equiv.), DMAP (12.2 mg, 0.100 mmol, 0.100 equiv.) and CH_2Cl_2 (10 mL) for 70 h. Purification by flash column chromatography (100:0→95:5 v/v EtOAc:MeOH) gave *N*-benzyl-*N*-(6-oxocyclohex-1-en-1-yl)-2(pyridin-3-yl)acetamide (**1k**) as an off-white solid (121 mg, 0.378 mmol, 38%).

m.p. = 66–68 °C.

IR (neat) ν_{max}/cm^{-1} = 3030, 29833, 1684, 1658, 1632, 1577, 1495, 1480, 1454, 1426, 1397, 1361, 1345, 1266, 1218, 1190, 1153, 1124, 1080, 1029, 1006, 977, 916, 854, 795, 711, 627.

^{1}H NMR ($CDCl_3$, 400 MHz) δ = 8.51–8.44 (m, 1H), 8.34 (s, 1H), 7.70–7.62 (m, 1H), 7.29–7.23 (m, 4H), 7.19–7.13 (m, 2H), 6.39 (t, *J* = 4.3 Hz, 1H), 5.33 (d, *J* = 14.4 Hz, 1H), 3.95 (d, *J* = 14.4 Hz, 1H), 3.49 (d, *J* = 15.3 Hz, 1H), 3.44 (d, *J* = 15.3 Hz, 1H), 2.51–2.43 (m, 2H), 2.40–2.27 (m, 2H), 2.03–1.93 (m, 2H).

^{13}C NMR ($CDCl_3$, 101 MHz) δ = 195.0, 170.1, 150.5, 150.3, 148.4, 138.6, 137.2, 136.9, 131.3, 129.2, 128.6, 127.7, 123.6, 51.1, 38.5, 38.4, 26.0, 22.5.

HRMS (ESI$^+$) Found $[M+H]^+$ = 321.15973; $[C_{20}H_{21}O_2N_2]^+$ requires 321.15975, Δ -0.08 ppm.

Photocyclization

rac-(3*S*,4*S*)-1-Benzyl-3-methyl-1-azaspiro[3.5]nonane-2,5-dione (**2a**)

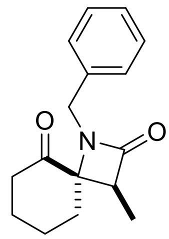

Prepared according to **General Procedure F** with *N*-benzyl-*N*-(6-oxocyclohex-1-en-1-yl)propionamide (**1a**) (77.2 mg, 0.300 mmol, 1.00 equiv.), Ir(dFppy)$_3$ (2.3 mg, 0.003 mmol, 0.01 equiv.), and EtOAc (30 mL) for 16 h. Purification by flash column chromatography (2:1→1:1 v/v Petrol ether 40–60:EtOAc) gave *rac*-(3*S*,4*S*)-1-benzyl-3-methyl-1-azaspiro[3.5]nonane-2,5-dione (**2a**) as a yellow oil (53 mg, 69%, >20:1 d.r., 10:1 r.r).

IR (neat) ν_{max}/cm^{-1} = 3030, 2935, 2862, 1747, 1710, 1602, 1496, 1454, 1398, 1350, 1156, 1107, 1077, 1029, 1013, 970, 913, 875, 856, 822, 782, 731.

^{1}H NMR ($CDCl_3$, 400 MHz) δ = 7.37–7.19 (m, 5H), 4.88 (d, *J* = 15.4 Hz, 1H), 4.10 (d, *J* = 15.4 Hz, 1H), 3.08 (q, *J* = 7.5 Hz, 1H), 2.64–2.53 (m, 1H), 2.22–2.11 (m, 1H), 2.04–1.95 (m, 1H), 1.80–1.70 (m, 2H), 1.60–1.47 (m, 3H), 1.20 (d, *J* = 7.5 Hz, 3H).

^{13}C NMR ($CDCl_3$, 101 MHz) δ = 208.2, 168.4, 137.3, 128.7, 128.5, 127.6, 71.1, 55.7, 44.8, 42.5, 37.8, 26.0, 23.4, 11.0.

HRMS (ESI$^+$) Found [M+Na]$^+$ = 280.13068; $[C_{16}H_{19}O_2Na]^+$ requires 280.13080, Δ -0.44 ppm.

Data for _rac_-(3*S*,3a*R*)-1-benzyl-3a-hydroxy-3-methyl-1,3,3a,4,5,6-hexahydro-2*H*-indol-2-one (7a)

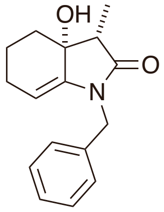

^{1}H NMR (C_6D_6, 400 MHz) δ 7.28 – 6.91 (m, 5H, H11, H12, H13), 4.75 (d, *J* = 15.4 Hz, 1H, H10), 4.47 (dd, *J* = 4.2, 3.1 Hz, 1H, H8), 4.27 (d, *J* = 15.4 Hz, 1H, H10'), 1.98 (q, *J* = 7.2 Hz, 1H, H2), 1.79 – 1.45 (m, 3H, H6', H7, H7'), 1.46 – 1.12 (m, 2H, H5, H5'), 1.29 (d, 3H, *J* = 7.2 Hz, H3) 1.12 – 0.83 (m, 1H).

^{13}C NMR (C_6D_6, 100MHz) δ 174.8 (C2), 142.2 (C11), 137.0 (C9), 128.4 (Ar, C11/12/13), 127.4 (Ar, C11/12/13), 127.1 (Ar, C11/12/13), 99.0 (C8), 70.6 (C4), 47.2 (C2), 43.1 (C10), 32.2 (C7), 23.0 (C6), 17.3 (C5), 6.9 (C3).

HRMS (ESI): Found[M+H]$^+$ = 258.1490; $[C_{16}H_{20}O_2N]^+$ requires 258.1489, Δ = 0.39 ppm.

rac-(3*S*,4*S*)-1-benzyl-3-(4-methoxyphenyl)-1-azaspiro[3.5]nonane-2,5-dione (**2b**)

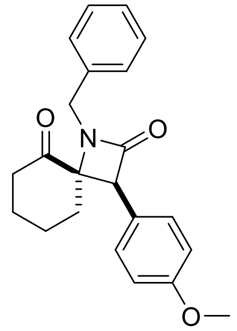

Prepared according to **General Procedure F** with *N*-benzyl-2-(4-methoxyphenyl)-*N*-(6-oxocyclohex-1-en-1-yl)acetamide (**1b**) (104.8 mg, 0.300 mmol, 1.00 equiv.), Ir(dFppy)$_3$ (2.3 mg, 0.003 mmol, 0.01 equiv.), and EtOAc (30 mL) for 16 h. Purification by flash column chromatography (4:1→2:1 v/v Petrol ether 40–60:EtOAc) gave *rac*-(3*S*,4*S*)-1-benzyl-3-(4-methoxyphenyl)-1-azaspiro[3.5]nonane-2,5-dione (**2b**) as an off-white solid (86 mg, 82%, >20:1 d.r., >20:1 r.r).

m.p. = 163–165 °C.

IR (neat) ν_{max}/cm^{-1} = 2935, 1751, 1710, 1612, 1515, 1454, 1396, 1297, 1251, 1181, 1134, 1111, 1056, 1030, 917, 837, 798, 728, 700.

^{1}H NMR ($CDCl_3$, 400 MHz) δ = 7.28–7.18 (m, 5H), 7.00 (d, *J* = 8.5 Hz, 2H), 6.77 (d, *J* = 8.5 Hz, 2H), 4.98 (d, *J* = 15.2 Hz, 1H), 4.18 (d, *J* = 15.2 Hz, 1H), 4.17 (s, 1H), 3.69 (s, 3H), 2.08–2.00 (m, 1H), 1.82–1.75 (m, 2H), 1.74–1.67 (m, 1H), 1.61–1.52 (m, 2H), 1.40–1.30 (m, 1H), 1.26–1.17 (m, 1H).

^{13}C NMR ($CDCl_3$, 101 MHz) δ = 208.0, 166.8, 159.6, 137.3, 130.3, 128.8, 128.6, 127.8, 124.3, 114.5, 74.0, 67.0, 55.4, 45.0, 42.0, 38.5, 26.2, 23.4.

HRMS (ESI^+) Found $[M+H]^+$ = 350.17520; $[C_{22}H_{24}O_3N]^+$ requires 350.17507, Δ 0.37 ppm.

rac-(3*S*,4*S*)-1-benzyl-3-(*p*-tolyl)-1-azaspiro[3.5]nonane-2,5-dione (2c)

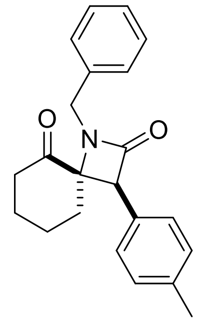

Prepared according to **General Procedure F** with *N*-benzyl-*N*-(6-oxocyclohex-1-en-1-yl)-2-(p-tolyl)acetamide (**1c**) (100.0 mg, 0.300 mmol, 1.00 equiv.), $Ir(dFppy)_3$ (2.3 mg, 0.003 mmol, 0.01 equiv.), and EtOAc (30 mL) for 16 h. Purification by flash column chromatography (5:1→4:1 v/v Petrol ether 40–60:EtOAc) gave *rac*-(3*S*,4*S*)-1-benzyl-3-(*p*-tolyl)-1-azaspiro[3.5]nonane-2,5-dione (**2c**) as an off-white solid (79.0 mg, 79%, >20:1 d.r., >20:1 r.r).

m.p. = 164–166 °C.

IR (neat) ν_{max}/cm^{-1} = 2931, 2861, 1750, 1710, 1517, 1496, 1453, 1395, 1350, 1160, 1133, 1109, 1078, 1056, 1003, 917, 829, 791, 730, 699, 646.

^{1}H NMR ($CDCl_3$, 400 MHz) δ = 7.37–7.25 (m, 5H), 7.12 (d, *J* = 7.7 Hz, 2H), 7.04 (d, *J* = 7.8 Hz, 2H), 5.07 (d, *J* = 15.3 Hz, 1H), 4.29 (d, *J* = 15.3 Hz, 1H), 4.25 (s, 1H), 2.30 (s, 3H), 2.14–2.06 (m, 1H), 1.90–1.76 (m, 3H), 1.70–1.56 (m, 2H), 1.48–1.36 (m, 1H), 1.33–1.23 (m, 1H).

^{13}C NMR ($CDCl_3$, 101 MHz) δ = 207.9, 166.6, 138.3, 137.3, 129.8, 129.3, 129.0, 128.7, 128.6, 127.7, 74.0, 67.3, 45.0, 41.9, 38.6, 26.2, 23.4, 21.3.

HRMS (ESI^+) Found $[M+H]^+$ = 334.18027; $[C_{22}H_{24}O_2N]^+$ requires 334.18016, Δ 0.33 ppm.

rac-(3*S*,4*S*)-1-benzyl-3-phenyl-1-azaspiro[3.5]nonane-2,5-dione (2d)

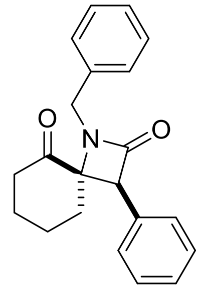

Prepared according to **General Procedure F** with *N*-benzyl-*N*-(6-oxocyclohex-1-en-1-yl)-2-phenylacetamide (**1d**) (95.8 mg, 0.300 mmol, 1.00 equiv.), $Ir(dFppy)_3$ (2.3 mg, 0.003 mmol, 0.01 equiv.), and EtOAc (30 mL) for 16 h. Purification by flash column chromatography (4:1→3:1 v/v Petrol ether 40–60:EtOAc) gave *rac*-(3*S*,4*S*)-1-benzyl-3-phenyl-1-azaspiro[3.5]nonane-2,5-dione (**2d**) as an off-white solid (68 mg, 71%, >20:1 d.r., >20:1 r.r).

m.p. = 133–135 °C from $CHCl_3$ (lit. 152–154 °C from Et_2O).

IR (neat) ν_{max}/cm^{-1} = 2935, 1749, 1710, 1497, 1454, 1395, 1349, 1159, 1133, 1108, 1077, 1056, 1029, 1001, 956, 914, 843, 807, 758, 731, 699, 646, 621.

^{1}H NMR ($CDCl_3$, 400 MHz) δ = 7.40–7.23 (m, 8H), 7.19–7.12 (m, 2H), 5.07 (d, *J* = 15.3 Hz, 1H), 4.29 (d, *J* = 15.1 Hz, 1H), 4.28 (s, 1H) 2.14–2.04 (m, 1H), 1.94–1.73 (m, 3H), 1.71–1.56 (m, 2H), 1.50–1.35 (m, 1H), 1.32–1.18 (m, 1H).

^{13}C NMR ($CDCl_3$, 101 MHz) δ = 207.7, 166.3, 137.3, 132.4, 129.1, 128.8, 128.6, 128.5, 127.8, 74.0, 67.6, 45.0, 41.8, 38.7, 26.2, 23.4.

HRMS (ESI$^+$) Found $[M+H]^+$ = 320.16449; $[C_{21}H_{22}O_2N]^+$ requires 320.16451, Δ -0.05 ppm.

***rac*-(3*S*,4*S*)-1-benzyl-3-(4-fluorophenyl)-1-azaspiro[3.5]nonane-2,5-dione (2e)**

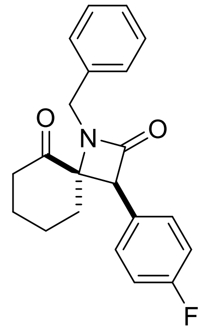

Prepared according to **General Procedure F** with *N*-benzyl-2-(4-fluorophenyl)-*N*-(6-oxocyclohex-1-en-1-yl)acetamide (**1e**) (101.2 mg, 0.300 mmol, 1.00 equiv.), $Ir(dFppy)_3$ (2.3 mg, 0.003 mmol, 0.01 equiv.), and EtOAc (30 mL) for 16 h. Purification by flash column chromatography (4:1→2:1 v/v Petrol ether 40–60:EtOAc) gave *rac*-(3*S*,4*S*)-1-benzyl-3-(4-fluorophenyl)-1-azaspiro[3.5]nonane-2,5-dione (**2e**) as a white solid (75 mg, 74%, >20:1 d.r., 17:1 r.r.).

m.p. = 140–142 °C.

IR (neat) ν_{max}/cm^{-1} = 2935, 1750, 1711, 1606, 1511, 1453, 1397, 1350, 1226, 1161, 1134, 1101, 1056, 1004, 917, 841, 802, 731, 699, 648.

^{1}H NMR ($CDCl_3$, 400 MHz) *δ* = 7.32–7.16 (m, 5H), 7.10–7.00 (m, 2H), 6.99–6.86 (m, 2H), 4.98 (d, *J* = 15.3 Hz, 1H), 4.30–4.08 (d, *J* = 15.3 Hz, 1H), 4.19 (s, 1H) 2.11–1.98 (m, 1H), 1.88–1.70 (m, 3H), 1.63–1.51 (m, 2H), 1.43–1.32 (m, 1H), 1.22–1.11 (m, 1H).

^{13}C NMR ($CDCl_3$, 101 MHz) *δ* = 207.6, 166.1, 162.7 (d, *J* = 248.0 Hz), 137.1, 130.93 (d, *J* = 8.5 Hz), 128.8, 128.6, 128.3 (d, *J* = 3.7 Hz), 127.8, 116.2 (d, *J* = 21.8 Hz), 73.9, 66.7, 45.1, 42.0, 38.5, 26.2, 23.4.

^{19}F NMR ($CDCl_3$, 377 MHz) *δ* = -112.92 (tt, *J* = 8.9, 5.3 Hz).

HRMS (ESI$^+$) Found $[M+H]^+$ = 338.15524; $[C_{21}H_{21}O_2NF]^+$ requires 338.15508, Δ 0.47 ppm.

***rac*-(3*S*,4*S*)-1-benzyl-3-(4-bromophenyl)-1-azaspiro[3.5]nonane-2,5-dione (2f)**

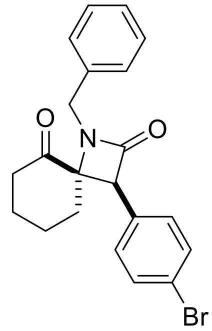

Prepared according to **General Procedure F** with *N*-benzyl-2-(4-bromophenyl)-*N*-(6-oxocyclohex-1-en-1-yl)acetamide (**1f**) (119.5 mg, 0.300 mmol, 1.00 equiv.), $Ir(dFppy)_3$ (2.3 mg, 0.003 mmol, 0.01 equiv.), and EtOAc (30 mL) for 16 h. Purification by flash column chromatography (4:1→3:1 v/v Petrol ether 40–60:EtOAc) gave *rac*-(3*S*,4*S*)-1-benzyl-3-(4-bromophenyl)-1-azaspiro[3.5]nonane-2,5-dione (**2f**) as an off-white solid (72 mg, 65%, >20:1 d.r., >20:1 r.r).

m.p. = 201–203 °C.

IR (neat) ν_{max}/cm^{-1} = 2935, 2861, 1752, 1710, 1592, 1489, 1454, 1397, 1350, 1234, 1172, 1133, 1106, 1074, 1055, 1012, 908, 866, 834, 791, 730, 702, 645.

^{1}H NMR ($CDCl_3$, 400 MHz) *δ* = 7.41 (d, *J* = 8.1 Hz, 2H), 7.31–7.21 (m, 5H), 6.99 (d, *J* = 8.0 Hz, 2H), 5.01 (d, *J* = 15.3 Hz, 1H), 4.23 (d, *J* = 15.3 Hz, 1H), 4.19 (s, 1H), 2.15–2.08 (m, 1H), 1.87–1.74 (m, 3H), 1.62–1.54 (m, 2H), 1.45–1.34 (m, 1H), 1.26–1.17 (1H, m, 1H).

^{13}C NMR ($CDCl_3$, 101 MHz) *δ* = 207.4, 165.7, 137.0, 132.3, 131.5, 130.8, 128.8, 128.6, 127.9, 122.7, 73.8, 66.8, 45.1, 42.0, 38.5, 26.1, 23.4.

HRMS (ESI$^+$) Found $[M+H]^+$ = 398.07513; $[C_{21}H_{21}O_2NBr]^+$ requires 398.07502, Δ 0.29 ppm.

rac-(3S,4S)-1-benzyl-3-(3-fluorophenyl)-1-azaspiro[3.5]nonane-2,5-dione (2g)

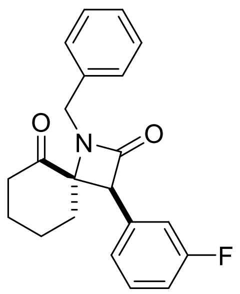

Prepared according to **General Procedure F** with *N*-benzyl-2-(3-fluorophenyl)-*N*-(6-oxocyclohex-1-en-1-yl)acetamide (**1g**) (101.2 mg, 0.300 mmol, 1.00 equiv.), Ir(dFppy)$_3$ (2.3 mg, 0.003 mmol, 0.01 equiv.), and EtOAc (30 mL) for 16 h. Purification by flash column chromatography (3:1→2:1 v/v Petrol ether 40–60:EtOAc) gave *rac*-(3*S*,4*S*)-1-benzyl-3-(3-fluorophenyl)-1-azaspiro[3.5]nonane-2,5-dione (**2g**) as an off-white solid (65 mg, 64%, >20:1 d.r., >20:1 r.r).

m.p. = 105–107 °C.

IR (neat) ν_{max}/cm^{-1} = 3031, 2938, 2862, 1751, 1711, 1615, 1589, 1489, 1451, 1397, 1351, 1310, 1271, 1241, 1224, 1182, 1150, 1133, 1107, 1077, 1055, 1029, 1003, 964, 914, 858, 782, 731, 699, 683, 647, 608.

^{1}H NMR (CDCl$_3$, 400 MHz) δ = 7.33–7.23 (m, 6H), 7.00–6.90 (m, 2H), 6.89–6.82 (m, 1H), 5.03 (d, *J* = 15.3 Hz, 1H), 4.25 (d, *J* = 15.2 Hz, 1H), 4.23 (s, 1H), 2.16–2.09 (m, 1H), 1.88–1.76 (m, 3H), 1.66–1.57 (m, 2H), 1.47–1.36 (m, 1H), 1.30–1.22 (m, 1H).s

^{13}C NMR (CDCl$_3$, 101 MHz) δ = 207.3, 165.6, 162.91 (d, *J* = 247.9 Hz), 137.1, 134.8 (d, *J* = 7.5 Hz), 130.8 (d, *J* = 8.6 Hz), 128.8, 128.6, 127.9, 124.9 (d, *J* = 2.9 Hz), 116.3 (d, *J* = 22.1 Hz), 115.6 (d, *J* = 20.8 Hz), 73.9, 66.9 (d, *J* = 1.9 Hz), 45.1, 41.9, 38.6, 26.2, 23.4.

^{19}F NMR (CDCl$_3$, 377 MHz) δ = -111.52 (td, *J* = 9.0, 5.9 Hz).

HRMS (ESI$^+$) Found [M+Na]$^+$ = 360.13718; $[C_{21}H_{20}O_2NFNa]^+$ requires 360.13703, Δ 0.41 ppm.

rac-(3S,4S)-1-benzyl-3-(4-trifluoromethyl)phenyl)-1-azaspiro[3.5]nonane-2,5-dione (2h)

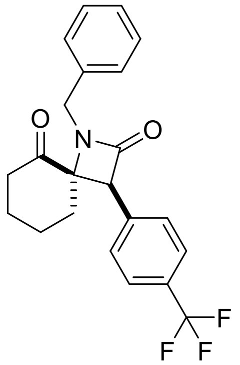

Prepared according to **General Procedure F** with *N*-benzyl-*N*-(6-oxocyclohex-1-en-1-yl)-2-(4-(trifluoromethyl)phenyl)acetamide (**1h**) (116.2 mg, 0.300 mmol, 1.00 equiv.), Ir(dFppy)$_3$ (2.3 mg, 0.003 mmol, 0.01 equiv.), and EtOAc (30 mL) for 16 h. Purification by flash column chromatography (4:1→3:1 v/v Petrol ether 40–60:EtOAc) gave *rac*-(3*S*,4*S*)-1-benzyl-3-(4-trifluoromethyl)phenyl)-1-azaspiro[3.5]nonane-2,5-dione (**2h**) as an off-white solid (50 mg, 43%, >20:1 d.r., 6:1 r.r.).

m.p. = 141–143 °C.

IR (neat) ν_{max}/cm^{-1} = 2939, 1755, 1713, 1620, 1496, 1453, 1421, 1398, 1324, 1260, 1166, 1124, 1113, 1070, 1019, 955, 918, 845, 798, 734, 701, 646.

^{1}H NMR (CDCl$_3$, 400 MHz) δ = 7.51 (d, *J* = 8.1 Hz, 2H), 7.28–7.17 (m, 7H), 4.99 (d, *J* = 15.3 Hz, 1H), 4.24 (s, 1H), 4.19 (d, *J* = 15.2 Hz, 1H), 2.11–2.04 (m, 1H), 1.86–1.73 (m, 3H), 1.59–1.52 (m, 2H), 1.42–1.31 (m, 1H), 1.17–1.07 (m, 1H).

^{13}C NMR (CDCl$_3$, 101 MHz) δ = 207.2, 165.4, 137.0, 136.6, 130.7 (d, *J* = 32.8 Hz), 129.6, 128.9, 128.7, 128.0, 126.2 (q, *J* = 4.0 Hz, 125.3, 73.9, 66.9, 45.2, 42.0, 38.6, 26.1, 23.5.

^{19}F NMR (CDCl$_3$, 377 MHz) δ = -62.76 (s)

HRMS (ESI$^+$) Found [M+H]$^+$ = 388.15207; $[C_{22}H_{21}O_2NF_3]^+$ requires 388.15189, Δ 0.46 ppm.

rac-4-((3S,4S)-1-benzyl-2,5-dioxo-1-azaspiro[3.5]nonan-3-yl)benzonitrile (2i)

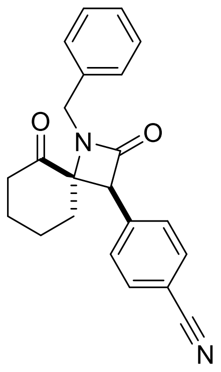

Prepared according to **General Procedure F** with *N*-benzyl-2-(4-cyanophenyl)-*N*-(6-oxocyclohex-1-en-1-yl)acetamide (**1i**) (103.3 mg, 0.300 mmol, 1.00 equiv.), Ir(dFppy)$_3$ (2.3 mg, 0.003 mmol, 0.01 equiv.), and EtOAc (30 mL) for 16 h. Purification by flash column chromatography (2:1→1:1 v/v Petrol ether 40–60:EtOAc) gave *rac*-(4-((3*S*,4*S*)-1-benzyl-2,5-dioxo-1-azaspiro[3.5]nonan-3-yl)benzonitrile (**2i**) as an off-white solid (28 mg, 27%, >20:1 d.r., 6:1 r.r

m.p. = 179–181 °C.

IR (neat) ν_{max}/cm^{-1} = 2940, 2229, 1752, 1711, 1608, 1496, 1452, 1398, 1351, 1134, 1110, 1055, 1004, 916, 844, 796, 729, 699, 648.

^{1}H NMR (CDCl$_3$, 400 MHz) δ = 7.58–7.52 (m, 2H), 7.27–7.19 (m, 7H), 4.97 (d, *J* = 15.2 Hz, 1H), 4.24 (s, 1H), 4.19 (d, *J* = 15.3 Hz, 1H), 2.13–2.06 (m, 1H), 1.88–1.75 (m, 3H), 1.61–1.48 (m, 2H), 1.44–1.33 (m, 1H), 1.15–1.04 (m, 1H).

^{13}C NMR (CDCl$_3$, 101 MHz) δ = 206.8, 164.9, 137.9, 136.8, 132.8, 130.0, 128.9, 128.6, 128.0, 118.3, 112.5, 73.9, 66.9, 45.2, 42.0, 38.5, 26.0, 23.5.

HRMS (ESI$^+$) Found [M+H]$^+$ = 345.1599; [$C_{22}H_{21}O_2N_2$]$^+$ requires 345.1598, Δ 0.46 ppm.

rac-(3S,4S)-1-Benzyl-3-methoxy-1-azaspiro[3.5]nonane-2,5-dione (2j)

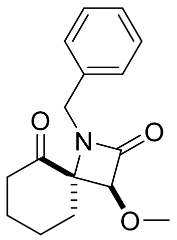

Prepared according to **General Procedure F** with *N*-benzyl-2-methoxy-*N*-(6-oxocyclohex-1-en-1-yl)acetamide (**1j**) (82.0 mg, 0.300 mmol, 1.00 equiv.), Ir(dFppy)$_3$ (2.3 mg, 0.003 mmol, 0.01 equiv.), and EtOAc (30 mL) for 16 h. Purification by flash column chromatography (3:1→2:1 v/v Petrol ether 40–60:EtOAc) gave *rac*-(3*S*,4*S*)-1-benzyl-3-methoxy-1-azaspiro[3.5]nonane-2,5-dione (**2j**) as a yellow oil (59 mg, 72%, 10:1 d.r., >20:1 r.r).

IR (neat) ν_{max}/cm^{-1} = 2937, 1753, 1716, 1496, 1452, 1394, 1350, 1215, 1167, 1145, 1108, 1077, 1050, 1004, 818, 700.

^{1}H NMR (CDCl$_3$, 400 MHz) δ = 7.34–7.20 (m, 5H), 4.93 (d, *J* = 15.3 Hz, 1H), 4.30 (s, 1H), 4.16 (d, *J* = 15.3 Hz, 1H), 3.57 (s, 3H), 2.57–2.47 (m, 1H), 2.47–2.34 (m, 1H), 2.07–1.94 (m, 1H), 1.84–1.73 (m, 1H), 1.72–1.57 (m, 2H), 1.57–1.40 (m, 2H).

^{13}C NMR (CDCl$_3$, 101 MHz) δ = 206.8, 165.4, 137.0, 128.7, 128.5, 127.8, 90.5, 72.8, 58.9, 44.6, 42.0, 36.2, 26.2, 23.4.

HRMS (ESI$^+$) Found [M+H]$^+$ = 274.14386; [$C_{16}H_{20}O_3N$]$^+$ requires 274.14377, Δ 0.33 ppm.

rac-(3S,4S)-1,3-dimethyl-1-azaspiro[3.5]nonane-2,5-dione (2l)

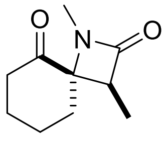

Prepared according to **General Procedure F** with *N*-methyl-*N*-(6-oxocyclohex-1-en-1-yl)propionamide (**1l**) (54.3 mg, 0.300 mmol, 1.00 equiv.), Ir(dFppy)$_3$ (2.3 mg, 0.003 mmol, 0.01 equiv.), and EtOAc (30 mL) for 16 h. Purification by flash column chromatography (2:1→0:1 v/v Petrol ether 40–60:EtOAc) gave *rac*-(3*S*,4*S*)-1,3-dimethyl-1-azaspiro[3.5]nonane-2,5-dione (**2l**) as a yellow oil (30 mg, 56%, >20:1 d.r., 8:1 r.r.).

IR (neat) ν_{max}/cm^{-1} = 2936, 2864, 1744, 1709, 1451, 1424, 1392, 1319, 1260, 1113, 1071, 1048, 991, 955, 894, 857, 780, 740.

^{1}H NMR ($CDCl_3$, 400 MHz) *δ* = 3.03 (qd, *J* = 7.6, 0.8 Hz, 1H), 2.79 (d, *J* = 0.7 Hz, 3H), 2.63–2.55 (m, 1H), 2.25–2.12 (m, 1H), 2.12–2.03 (m, 2H), 2.03–1.93 (m, 2H), 1.70–1.60 (m, 2H), 1.17 (d, *J* = 7.5 Hz, 3H).

^{13}C NMR ($CDCl_3$, 101 MHz) *δ* = 208.0, 168.4, 69.9, 55.4, 42.5, 35.6, 26.2, 25.4, 23.3, 10.9.

HRMS (ESI$^+$) Found $[M+Na]^+$ = 204.09969; $[C_{10}H_{15}O_2NNa]^+$ requires 204.09950, Δ 0.91 ppm.

***rac*-(3*S*,4*S*)-1-(4-methoxybenzyl)-3-methyl-1-azaspiro[3.5]nonane-2,5-dione** (**2m**)

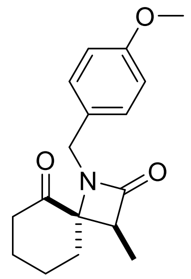

Prepared according to **General Procedure F** with *N*-(4-methoxybenzyl)-*N*-(6-oxocyclohex-1-en-1-yl)propionamide (**1m**) (86.2 mg, 0.300 mmol, 1.00 equiv.), $Ir(dFppy)_3$ (2.3 mg, 0.003 mmol, 0.01 equiv.), and EtOAc (30 mL) for 16 h. Purification by flash column chromatography (2:1→1:2 v/v Petrol ether 40–60:EtOAc) gave *rac*-(3*S*,4*S*)-1-(4-methoxybenzyl)-3-methyl-1-azaspiro[3.5]nonane-2,5-dione (**2m**) as a yellow oil (55 mg, 64%, >20:1 d.r., 15:1 r.r).

IR (neat) ν_{max}/cm^{-1} = 2935, 2862, 1745, 1709, 1613, 1586, 1513, 1453, 1421, 1398, 1348, 1304, 1245, 1177, 1102, 1080, 1064, 1032, 970, 914, 879, 847, 822, 766, 682.

^{1}H NMR ($CDCl_3$, 400 MHz) *δ* = 7.19 (d, *J* = 8.5 Hz, 2H), 6.82 (d, *J* = 8.5 Hz, 2H), 4.83 (d, *J* = 15.2 Hz, 1H), 4.06 (d, *J* = 15.2 Hz, 1H), 3.78 (s, 3H), 3.06 (q, *J* = 7.5 Hz, 1H), 2.64–2.54 (m, 1H), 2.21–2.11 (m, 1H), 2.03–1.95 (m, 1H), 1.80–1.68 (m, 2H), 1.56–1.44 (m, 3H), 1.20 (d, *J* = 7.5 Hz, 3H).

^{13}C NMR ($CDCl_3$, 101 MHz) *δ* = 208.3, 168.4, 159.1, 129.9, 129.3, 114.0, 71.0, 55.6, 55.4, 44.2, 42.6, 37.9, 26.0, 23.4, 11.1.

HRMS (ESI$^+$) Found $[M+H]^+$ = 288.15942; $[C_{17}H_{22}O_3N]^+$ requires 288.15942, Δ 0.01 ppm.

***rac*-(3*S*,4*S*)-3-methyl-1-(pyridine-2-ylmethyl)-1-azaspiro[3.5]nonane-2,5-dione** (**2n**)

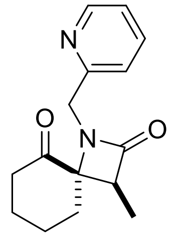

Prepared according to **General Procedure F** with *N*-(6-oxocyclohex-1-en-1-yl)-*N*-(pyridin-2-ylmethyl)propionamide (**1n**) (77.5 mg, 0.300 mmol, 1.00 equiv.), $Ir(dFppy)_3$ (2.3 mg, 0.003 mmol, 0.01 equiv.), and EtOAc (30 mL) for 16 h. Purification by flash column chromatography (98:2→95:5 v/v CH_2Cl_2:MeOH) gave *rac*-(3*S*,4*S*)-3-methyl-1-(pyridine-2-ylmethyl)-1-azaspiro[3.5]nonane-2,5-dione (**2n**) as a yellow oil (50 mg, 65%, >20:1 d.r., 15:1 r.r).

IR (neat) ν_{max}/cm^{-1} = 2937, 2863, 1749, 1711, 1592, 1571, 1475, 1436, 1397, 1375, 1339, 1312, 1270, 1154, 1132, 1110, 1092, 1080, 1064, 1049, 1013, 995, 953, 912, 878, 856, 825, 753, 729, 683, 646, 632.

^{1}H NMR ($CDCl_3$, 400 MHz) *δ* = 8.52–8.40 (m, 1H), 7.64 (td, *J* = 7.7, 1.8 Hz, 1H), 7.40 (dd, *J* = 7.9, 1.2 Hz, 1H), 7.21–7.11 (m, 1H), 4.81 (d, *J* = 15.8 Hz, 1H), 4.30 (d, *J* = 15.9 Hz, 1H), 3.10 (q, *J* = 7.5 Hz, 1H), 2.62–2.49 (m, 1H), 2.23–2.08 (m, 1H), 2.08–1.94 (m, 1H), 1.91–1.77 (m, 3H), 1.66–1.46 (m, 2H), 1.19 (d, *J* = 7.6 Hz, 3H).

^{13}C NMR ($CDCl_3$, 101 MHz) *δ* = 207.5, 168.6, 157.1, 149.2, 136.9, 123.1, 122.5, 71.1, 55.6, 46.8, 42.4, 37.2, 25.9, 23.4, 11.0.

HRMS (ESI$^+$) Found $[M+H]^+$ = 259.14413; $[C_{15}H_{19}O_2N_2]^+$ requires 259.14410, Δ 0.12 ppm.

rac-(3S,4S)-1-(furan-2-ylmethyl)-3-methyl-1-azaspiro[3.5]nonane-2,5-dione (**2o**)

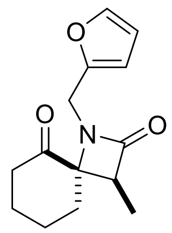

Prepared according to **General Procedure F** with N-(furan-2-ylmethyl)-N-(6-oxocyclohex-1-en-1-yl)propionamide (**1o**) (74.2 mg, 0.300 mmol, 1.00 equiv.), Ir(dFppy)$_3$ (2.3 mg, 0.003 mmol, 0.01 equiv.), and EtOAc (30 mL) for 16 h. Purification by flash column chromatography (2:1→1:1 v/v Petrol ether 40–60:EtOAc) gave *rac*-(3*S*,4*S*)-1-(furan-2-ylmethyl)-3-methyl-1-azaspiro[3.5]nonane-2,5-dione (**2o**) as a yellow oil (46mg, 62%, >20:1 d.r., >20:1 r.r).

IR (neat) ν_{max}/cm^{-1} = 2938, 2865, 1748, 1709, 1600, 1504, 1453, 1424, 1397, 1350, 1309, 1275, 1234, 1148, 1106, 1075, 1014, 972, 954, 922, 884, 856, 818, 749, 683.

^{1}H NMR (CDCl$_3$, 400 MHz) δ = 7.34 (dd, *J* = 1.9, 0.9 Hz, 1H), 6.30 (dd, *J* = 3.2, 1.9 Hz, 1H), 6.24–6.19 (m, 1H), 4.77 (d, *J* = 15.8 Hz, 1H), 4.25 (d, *J* = 15.8 Hz, 1H), 3.07 (q, *J* = 7.5 Hz, 1H), 2.64–2.56 (m, 1H), 2.23–2.12 (m, 1H), 2.10–1.95 (m, 1H), 1.91–1.80 (m, 2H), 1.72–1.51 (m, 3H), 1.20 (d, *J* = 7.5 Hz, 3H).

^{13}C NMR (CDCl$_3$, 101 MHz) δ = 208.0, 168.1, 149.6, 142.3, 110.7, 108.8, 70.6, 55.7, 42.5, 37.1, 36.7, 26.0, 23.4, 11.0.

HRMS (ESI$^+$) Found [M+H]$^+$ = 248.12825; [C$_{14}$H$_{18}$O$_3$N]$^+$ requires 248.12812, Δ 0.52 ppm.

rac-(3S,4S)-3-methyl-1-propyl-1-azaspiro[3.5]nonane-2,5-dione (**2p**)

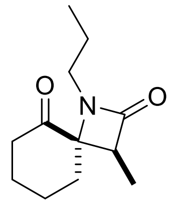

Prepared according to **General Procedure F** with N-(6-oxocyclohex-1-en-1-yl)-N-propylpropionamide (**1p**) (62.8 mg, 0.300 mmol, 1.00 equiv.), Ir(dFppy)$_3$ (2.3 mg, 0.003 mmol, 0.01 equiv.), and EtOAc (30 mL) for 16 h. Purification by flash column chromatography (2:1→1:2 v/v Petrol ether 40–60:EtOAc) gave *rac*-(3*S*,4*S*)-3-methyl-1-propyl-1-azaspiro[3.5]nonane-2,5-dione (**2p**) as a yellow oil (44 mg, 71%, >20:1 d.r., 10:1 r.r.).

IR (neat) ν_{max}/cm^{-1} = 2935, 2874, 1744, 1712, 1454, 1401, 1339, 1236, 1210, 1183, 1156, 1114, 1078, 1059, 956, 916, 854, 779, 680.

^{1}H NMR (CDCl$_3$, 400 MHz) δ = 3.34–3.20 (m, 1H), 3.06–2.92 (m, 2H), 2.64–2.51 (m, 1H), 2.26–1.91 (m, 5H), 1.72–1.54 (m, 4H), 1.15 (d, *J* = 7.5 Hz, 3H), 0.89 (t, *J* = 7.4 Hz, 3H).

^{13}C NMR (CDCl$_3$, 101 MHz) δ = 207.9, 168.7, 70.7, 55.1, 43.0, 42.5, 37.2, 26.1, 23.5, 22.5, 11.8, 11.0.

HRMS (ESI$^+$) Found [M+H]$^+$ = 210.14890; [C$_{12}$H$_{20}$O$_2$N]$^+$ requires 210.14886, Δ 0.19 ppm.

rac-(3S,4S)-1-beznyl-3-methyl-1-azaspiro[3.4]octane-2,5-dione (2q)

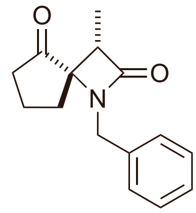

Prepared according to **General Procedure H** with N-benzyl-N-(5-oxocyclopent-1-en-1-yl)propionamide (**1q**) (73.8 mg, 0.300 mmol, 1.00 equiv.), Ir(dFppy)$_3$ (2.3 mg, 0.003 mmol, 0.01 equiv.), NaOAc (24.6 mg, 0.300 mmol, 1.00 equiv.) and EtOAc (30 mL) for 16 h. Purification by flash column chromatography (3:1→2:1 v/v Petrol ether 40–60:EtOAc) gave *rac*-(3*S*,4*S*)-1-beznyl-3-methyl-1-azaspiro[3.4]octane-2,5-dione (**2q**) as a yellow oil (45.5 mg, 0.0797 mmol, 62%, 8:1 d.r.).

IR (neat) ν_{max}/cm^{-1} = 2966, 1738, 1455, 1394, 1158, 1094, 951, 731, 701.

^{1}H NMR ($CDCl_3$, 400 MHz) δ = 7.31–7.21 (m, 5H), 4.75 (d, *J* = 15.3 Hz, 1H), 3.96 (d, *J* = 15.3 Hz, 1H), 3.18 (q, *J* = 7.4 Hz, 1H), 2.29–2.21 (m, 1H), 2.12–2.02 (m, 1H), 1.94–1.86 (m, 2H), 1.71–1.65 (m, 2H), 1.14 (d, *J* = 7.4 Hz, 3H).

^{13}C NMR ($CDCl_3$, 101 MHz) δ = 215.1, 169.0, 136.1, 128.8, 128.5, 127.9, 70.1, 57.1, 44.5, 36.5, 32.6, 17.9, 10.7.

HRMS (ESI$^+$) Found $[M+H]^+$ = 244.1335; $[C_{15}H_{18}O_2N]^+$ requires 244.1332, Δ 1.02 ppm.

***rac-N*-((3*S*,4*S*)-1-benzyl-2,5-dioxo-1-azaspiro[3.5]nonan-3-yl)acetamide** (**2r**)

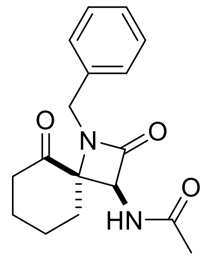

Prepared according to **General Procedure F** with 2-acetamido-*N*-benzyl-*N*-(6-oxocyclohex-1-en-1-yl)acetamide (**1r**) (90.1 mg, 0.300 mmol, 1.00 equiv.), Ir(dFppy)$_3$ (2.3 mg, 0.003 mmol, 0.01 equiv.), NaOAc (24.6 mg, 0.300 mmol, 1.00 equiv.) and EtOAc (30 mL) for 16 h. Purification by flash column chromatography (1:2→1:5 v/v Petrol ether 40–60:EtOAc) gave *rac-N*-((3*S*,4*S*)-1-benzyl-2,5-dioxo-1-azaspiro[3.5]nonan-3-yl)acetamide (**2r**) as an off-white solid (42.3 mg, 0.140 mmol, 47%, >20:1 d.r.).

m.p. = 147–149 °C.

IR (neat) ν_{max}/cm^{-1} = 3275, 3033, 2942, 2865, 1753, 1711, 1667, 1540, 1497, 1453, 1401, 1372, 1286, 1176, 1136, 1102, 1079, 1050, 1005, 918, 804, 731, 701, 646.

^{1}H NMR ($CDCl_3$, 400 MHz) δ = 7.36–7.23 (m, 5H), 6.79 (d, *J* = 8.4 Hz, 1H), 5.06 (d, *J* = 8.4 Hz, 1H), 4.96 (d, *J* = 15.4 Hz, 1H), 4.11 (d, *J* = 15.4 Hz, 1H), 2.55–2.43 (m, 1H), 2.10–1.91 (m, 3H), 2.02 (s, 3H), 1.88–1.78 (m, 1H), 1.78–1.68 (m, 1H), 1.60–1.37 (m, 2H).

^{13}C NMR ($CDCl_3$, 101 MHz) δ = 208.2, 170.8, 165.1, 136.4, 128.8, 128.5, 128.0, 74.5, 66.0, 45.1, 41.9, 35.9, 25.7, 23.1, 22.3.

HRMS (ESI$^+$) Found $[M+Na]^+$ = 323.13678; $[C_{17}H_{20}O_3N_2Na]^+$ requires 323.13661, Δ 0.51 ppm.

***rac*-(3*S*,4*R*)-1-benzyl-3-(phenylthiol)-1-azaspiro[3.5]nonane-2,5-dione** (**2s**)

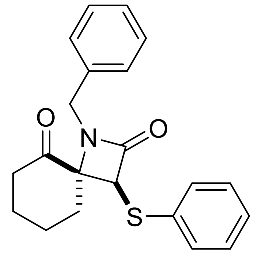

Prepared according to **General Procedure F** with *N*-benzyl-*N*-(6-oxocyclohex-1-en-1-yl)-2-(phenylthiol)acetamide (**1s**) (105.4 mg, 0.300 mmol, 1.00 equiv.), Ir(dFppy)$_3$ (2.3 mg, 0.003 mmol, 0.01 equiv.), and EtOAc (30 mL) for 16 h. Purification by flash column chromatography (4:1→3:1 v/v Petrol ether 40–60:EtOAc) gave *rac*-(3*S*,4*R*)-1-benzyl-3-(phenylthiol)-1-azaspiro[3.5]nonane-2,5-dione (**2s**) as a yellow oil (73 mg, 69%, >20:1 d.r., 10:1 r.r.).

IR (neat) ν_{max}/cm^{-1} = 3060, 2936, 2861, 1756, 1712, 1583, 1496, 1481, 1439, 1391, 1351, 1311, 1164, 1130, 1106, 1077, 1026, 1002, 956, 916, 860, 735, 698.

^{1}H NMR ($CDCl_3$, 400 MHz) δ = 7.55–7.42 (m, 2H), 7.31–7.17 (m, 8H), 4.85 (d, *J* = 15.3 Hz, 1H), 4.24 (s, 1H), 4.09 (d, *J* = 15.3 Hz, 1H), 2.64–2.48 (m, 2H), 1.92–1.84 (m, 1H), 1.70–1.61 (m, 2H), 1.55–1.47 (m, 1H), 1.46–1.27 (m, 2H).

^{13}C NMR ($CDCl_3$, 101 MHz) δ = 206.6, 164.3, 136.8, 133.2, 131.7, 129.4, 128.8, 128.6, 128.1, 127.8, 73.2, 63.1, 45.3, 42.6, 37.9, 26.2, 23.3.

HRMS (ESI$^+$) Found $[M+H]^+$ = 352.13666; $[C_{21}H_{22}O_2NS]^+$ requires 352.13658, Δ 0.23 ppm.

***rac*-(3*S*,4*S*)-1-Benzyl-3-cyclopropyl-1-azaspiro[3.5]nonane-2,5-dione** (**2t**)

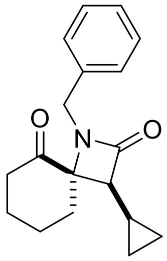

Prepared according to **General Procedure F** with *N*-benzyl-2-cyclopropyl-*N*-(6-oxocyclohex-1-en-1-yl)acetamide (**1t**) (85.0 mg, 0.300 mmol, 1.00 equiv.), Ir(dFppy)$_3$ (2.3 mg, 0.003 mmol, 0.01 equiv.), and EtOAc (30 mL) for 16 h. Purification by flash column chromatography (3:1→2:1 v/v Petrol ether 40–60:EtOAc) gave *rac*-(3*S*,4*S*)-1-benzyl-3-cyclopropyl-1-azaspiro[3.5]nonane-2,5-dione (**2t**) as a yellow oil (56 mg, 66%, >20:1 d.r., n/a r.r.).

IR (neat) ν_{max}/cm^{-1} = 3006, 2935, 2862, 1746, 1709, 1604, 1496, 1453, 1430, 1402, 1384, 1350, 1313, 1163, 1101, 1078, 1063, 1026, 967, 915, 851, 830, 733, 700.

^{1}H NMR ($CDCl_3$, 400 MHz) δ = 7.36–7.10 (m, 5H), 4.85 (d, *J* = 15.4 Hz, 1H), 4.07 (d, *J* = 15.4 Hz, 1H), 2.58–2.42 (m, 3H), 2.04–1.91 (m, 1H), 1.82–1.65 (m, 1H), 1.65–1.54 (m, 1H), 1.54–1.37 (m, 3H), 0.78–0.65 (m, 1H), 0.65–0.56 (m, 1H), 0.56–0.46 (m, 1H), 0.46–0.35 (m, 1H), 0.35–0.22 (m, 1H).

^{13}C NMR ($CDCl_3$, 101 MHz) δ = 208.7, 166.9, 137.4, 128.6, 128.5, 127.6, 71.4, 65.8, 44.7, 42.0, 37.7, 26.1, 23.5, 7.5, 4.4, 3.9.

HRMS (ESI$^+$) Found [M+H]$^+$ = 284.16428; [$C_{18}H_{22}O_2N$]$^+$ requires 284.16451, Δ -0.81 ppm.

***rac*-(3S,4S)-1-benzyl-3-(pyridin-3-yl)-1-azaspiro[3.5]nonane-2,5-dione** (**2u**)

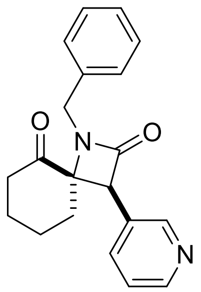

Prepared according to **General Procedure F** with *N*-benzyl-*N*-(6-oxocyclohex-1-en-1-yl)-2(pyridin-3-yl)acetamide (**1u**) (96.1 mg, 0.300 mmol, 1.00 equiv.), Ir(dFppy)$_3$ (2.3 mg, 0.003 mmol, 0.01 equiv.), and EtOAc (30 mL) for 16 h. Purification by flash column chromatography (100:0→96:4 v/v EtOAc:MeOH) gave (3*S**,4*S**)-1-benzyl-3-(pyridin-3-yl)-1-azaspiro[3.5]nonane-2,5-dione (**2k**) as an off-white solid (60.3 mg, 0.188 mmol, 63%, >20:1 d.r.).

m.p. = 161–163 °C.

IR (neat) ν_{max}/cm^{-1} = 3031, 2936, 2861, 1751, 1710, 1574, 1496, 1481, 1453, 1426, 1397, 1350, 1310, 1267, 1170, 1133, 1106, 1078, 1064, 1028, 1003, 955, 917, 865, 785, 731, 714, 646, 623.

^{1}H NMR ($CDCl_3$, 400 MHz) δ = 8.52 (dd, *J* = 4.9, 1.7 Hz, 1H), 8.47 (d, *J* = 2.2 Hz, 1H), 7.44 (dt, *J* = 8.0, 2.0 Hz, 1H), 7.36–7.31 (m, 4H), 7.29 (dd, *J* = 5.5, 3.0 Hz, 1H), 7.26–7.22 (m, 1H), 5.04 (d, *J* = 15.3 Hz, 1H), 4.30 (s, 1H), 4.23 (d, *J* = 15.3 Hz, 1H), 2.20–2.12 (m, 1H), 1.95–1.90 (m, 1H), 1.89–1.80 (m, 2H), 1.68–1.59 (m, 2H), 1.50–1.36 (m, 1H), 1.24–1.15 (m, 1H).

^{13}C NMR ($CDCl_3$, 101 MHz) δ = 207.0, 165.1, 150.7, 149.8, 136.7, 135.8, 128.8, 128.5, 128.4, 127.9, 123.8, 73.5, 64.3, 45.1, 42.0, 38.2, 25.8, 23.3.

HRMS (ESI$^+$) Found [M+H]$^+$ = 321.15985; [$C_{20}H_{21}O_2N$]$^+$ requires 321.15975, Δ 0.30 ppm.

Crossover substrate synthesis

Benzoic-2,3,4,5,6-*d*$_5$ acid (11)

This compound was synthesized by a modification of a literature procedure.[5]

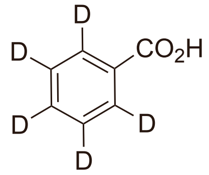

Sodium stearate (1.23 g, 4 mmol, 0.04 eq.) and potassium permanganate (47.4 g, 300 mmol, 3.0 eq) were stirred in a mixture of toluene-*d*$_8$ (10.6 mL, 100 mmol, 1.0 eq), and H_2O (200 mL). The resulting mixture was heated to 100 °C for 6 hours before being allowed to cool and filtered through a short NaCl column. The pH of the filtrate was adjusted to 3 with concentrated HCl (37% aq.) and the solid was filtered, washed with cold H_2O (~20 mL), collected, and recrystallised from H_2O to yield benzoic-2,3,4,5,6-*d*$_5$ acid **11** (5.16 g, 41 mmol, 41%) as a crystalline white solid.

m.p. = 108-110 °C

IR (film) ν_{max}/cm^{-1}: 1680, 1568, 1436, 1369, 1334, 1278, 1089, 1054, 939, 841, 824, 777, 728, 714, 647

^{1}H NMR (400 MHz, $CDCl_3$) δ = 12.48 (s, 1H)

^{2}H NMR (92 MHz, $CHCl_3$) δ = 8.17 (s, 2D), 7.66 (s, 1D), 7.52 (s, 1D).

^{13}C NMR (101 MHz, $CDCl_3$) δ = 172.6, 133.4 (t, *J* = 24 Hz), 129.9 (t, *J* = 24.8 Hz), 129.2, 128.0 (t, *J* = 24.6 Hz)

HRMS (ESI$^+$) $C_7H_2D_5O_2$ $[M+H]^+$ requires 126.0609; found 126.0611, Δ 1.7 ppm.

Benzamide-2,3,4,5,6-*d*$_5$ (12)

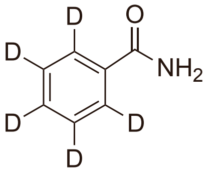

This compound was synthesized by a modification of a literature procedure.[6]

Oxalyl chloride (5.15 ml, 60 mmol, 1.5 eq.) was added dropwise to a stirring solution of benzoic-2,3,4,5,6-*d*$_5$ acid **11** (5.09 g, 40 mmol, 1.0 eq.) in dry CH_2Cl_2 (120 mL, 0.33 M) and dry DMF (3 drops) at 0 °C. The solution was allowed to warm to room temperature. After a further 4 h, the solution was evaporated to dryness and CH_2Cl_2 (10 mL) was added. This solution was added dropwise to a flask containing KOH (4.5 g, 80 mmol, 2.0 eq.) and NH_4Cl (2.6 g, 48 mmol, 1.2 eq.) in H_2O/MeCN (1:5, 170 mL, 0.24 M) at 0 °C. The resulting biphasic mixture was allowed to warm to rt overnight, after which time methanol (40 mL) was added and the solution was evaporated to dryness. The crude was product was then filtered through a pad of silica gel (8:2 CH_2Cl_2:MeOH) to afford benzamide-2,3,4,5,6-*d*$_5$ (**12**) (quantitive).

m.p. = 112-115 °C

IR (film) ν_{max}/cm^{-1}: 3366, 3167, 1655, 1621, 1544, 1421, 1344, 1291, 1264, 1129, 843, 818, 809, 784, 745, 715, 645, 624, 609

^{1}H NMR (500 MHz, $CDCl_3$) δ = 6.36 (s, 2H).

^{2}H NMR (92 MHz, $CHCl_3$) δ = 7.45 (s, 2D)

^{13}C NMR (126 MHz, $CDCl_3$) δ = 169.9, 133.2, 131.5 (t, *J* = 24.8 Hz), 128.1(t, *J* = 24.8 Hz, 2C), 127.0 (t, *J* = 24.3 Hz, 2C)

HRMS (ESI$^+$) $C_7H_3D_5NO$ $[M + H]^+$ requires 127.0914; found 127.0919, Δ 3.70 ppm.

(Phenyl-d_5)methanamineone (13)

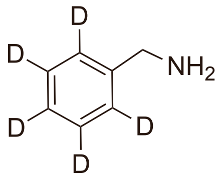

To a solution of benzamide-2,3,4,5,6-d_5 (**12**, 2.4 g, 19 mmol, 1.0 eq**.**) in dry THF (125 mL, 0.15 M) under an argon atmosphere at 0 °C was added lithium aluminium hydride (2.15 g, 57 mmol, 3.0 eq). The resulting suspension was refluxed for 2 hours before being cooled to room temperature. The mixture was then diluted with diethyl ether (100 mL) and cooled to 0 °C before H_2O (2.15 mL) was slowly added, followed by 15% sodium hydroxide (aq., 2.15 mL) and H_2O (6.45 mL). The reaction was then allowed to warm to rt before $MgSO_4$ was added and the suspension was allowed to stir for 15 min. The suspension was filtered and concentrated to dryness, affording crude (phenyl-d_5)methanamineone (**13**, 1.98 g, 17.6 mmol, 93%) which was used without further purification.

IR (film) ν_{max}/cm^{-1}: 2867, 1591, 1454, 1370, 1148, 1063, 1030, 963, 888, 841, 820, 773, 729, 641

^{2}H NMR (92 MHz, $CHCl_3$) δ = 7.53 – 7.16 (m, 5D).

^{13}C NMR (126 MHz, $CDCl_3$) δ = 143.0, 128.1 (t, J = 24.1 Hz, 2C), 126.7 (t, J = 23.5 Hz, 2C), 126.3 (t, J = 24.4 Hz), 46.39.

HRMS (ESI$^+$) $C_7H_5D_5N$ [M+H]$^+$ requires 113.1122; found 113.1123, Δ 1.04 ppm.

4-Iodobenzonitrile-3,5-d_2 (14)

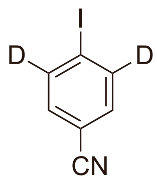

This compound was synthesized by a modification of a literature procedure.[7]

To a 35 mL microwave vial equipped with a stirrer bar was added 4-aminobenzonitrile hydrochloride (773 mg, 5.0 mmol, 1.0 eq.) followed by D_2O (24 mL, 0.2 M). The vial was sealed, warmed to 180 °C in a microwave for 30 min before being allowed to cool. This process was repeated on an identical scale, and the two reaction mixtures were combined. To the combined solutions were added NaOH (3 M aq., 8 mL), brine (4 mL), and Et_2O (8 mL) and the resulting biphasic mixture separated. The organic phase was washed with brine (10 mL) and concentrated to dryness.

To the solid was added H_2O (20 mL, 0.5 M) and then cooled to 0 °C. Concentrated H_2SO_4 (1.5 mL, 27.3 mmol, 2.7 eq.) was added dropwise and the solution was allowed to stir for 30 min. After 30 min, acetone (8 mL) was added and the solution was allowed to stir for another 15 min. A solution of $NaNO_2$ (1.66 g in 10 mL H_2O, 30 mmol, 3.0 eq.) was then added dropwise, and the solution was allowed to stir for 60 min. A solution of KI (6.64 g in 10 mL H_2O, 50 mmol, 5.0 eq.) was then added slowly, and the resulting mixture was allowed to warm to rt overnight. The reaction mixture was then extracted with EtOAc (3 x 10 mL), and the organic phases were combined and washed with 1M HCl (aq., 20 mL). The organic phase was then washed with ascorbic acid (aq., 20% w/w, 3 x 50 mL) and H_2O (3 x 25 mL) before being dried over $MgSO_4$, filtered and evaporated to dryness to yield 4-iodobenzonitrile-3,5-d_2 (**14**, 1.65 g, 7.1 mmol, 71%).

m.p. = 116-117 °C

IR (film) ν_{max}/cm^{-1}: 2229, 1825, 1656, 1573, 1461, 1423, 1365, 1323, 1268, 1087, 1020, 910, 898, 864, 825, 759, 729, 645, 634

^{1}H NMR (400 MHz, $CDCl_3$) δ = 7.36 (s, 2H)

^{2}H NMR (92 MHz, $CHCl_3$) δ = 7.89 (s, 2D).

^{13}C NMR (126 MHz, $CDCl_3$) δ = 138.19 (t, J = 26.4 Hz, 2C), 133.1 (2C), 118.2, 111.7, 100.1

HRMS (ESI^+) $C_7H_3D_2IN$ $[M+H]^+$ requires 231.9587; found 231.9587, Δ -0.09 ppm.

***Tert*-butyl 2-(4-cyanophenyl-2,6-*d*$_2$)acetate** (**15**)

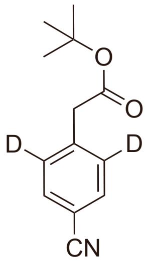

This compound was synthesized by a modification of a literature procedure.[8]

To a 35 mL microwave vial equipped with a stirrer bar were added 4-iodobenzonitrile-3,5-d_2 (**14**, 1.16 g, 5 mmol, 1.0 eq.), CuI (48 mg, 0.25 mmol, 0.05 eq.), 2-piccolinic acid (62 mg, 0.5 mmol, 0.1 eq.), and dry caesium carbonate (4.89 g, 15 mmol, 3.0 eq.). The vial was capped and vacuum and backfilled with argon three times before *tert*-butylacetoacetate (1.66 ml, 10 mmol, 2.0 eq) and dry dioxane (10 mL, 0.5 M) were added. The reaction was warmed to 70 °C for 48 h before H_2O (180 µL, 10 mmol, 2.0 eq) was added and the reaction stirred at 70 °C for a further 18 hours. Upon completion, the reaction mixture was diluted with EtOAc (25 mL), washed with brine (10 mL), dried over $MgSO_4$, filtered and concentrated to dryness. The crude solid was purified by flash column chromatography (49:1→ 19:1 v/v pentane:EtOAc) gave *Tert*-butyl 2-(4-cyanophenyl-2,6-d_2)acetate (**15**) as a white solid (1.01 g, 4.6 mmol, 77%).

m.p. = 77-80 °C

IR (film) ν_{max}/cm^{-1}: 2224, 1730, 1469, 1413, 1386, 1368, 1337, 1247, 1223, 1142, 940, 916, 897, 884, 862, 828, 754, 698, 637

^{1}H NMR (400 MHz, $CDCl_3$) δ = 7.55 – 7.48 (m, 2H), 3.54 – 3.49 (m, 2H), 1.41 – 1.32 (m, 9H)

^{2}H NMR (92 MHz, $CHCl_3$) δ = 7.43 (s, 2D)

^{13}C NMR (126 MHz, $CDCl_3$) δ = 169.6, 139.9, 132.1 (2C), 129.8 (t, J = 24.7 Hz, 2C), 118.8, 110.88, 81.6, 42.44, 28.0 (3C)

HRMS (ESI^+) $C_{13}H_{13}D_2NO_2Na$ $[M+Na]^+$ requires 242.1121; found 242.1120, Δ -0.24 ppm.

2-(4-Cyanophenyl-2,6-*d*$_2$)acetic acid (**16**)

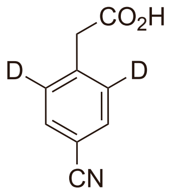

To a solution of *tert*-butyl 2-(4-cyanophenyl-2,6-d_2)acetate (**15**, 658 mg, 3.0 mmol, 1.0 eq) in CH_2Cl_2 (9 mL, 0.33 M) was added trifluoroacetic acid (1.15 mL, 15 mmol, 5.0 eq.). The resulting solution was stirred at room temperature for 1 h before being evaporated to dryness to afford 2-(4-Cyanophenyl-2,6-d_2)acetic acid (**16**, 421 mg, 2.6 mmol, 86%), which required no further purification.

m.p. = 136-139 °C

IR (film) ν_{max}/cm^{-1}: 2228, 1743, 1689, 1469, 1428, 1406, 1382, 1339, 1299, 1247, 1199, 1172, 1049, 939, 909, 876, 699, 672, 635

^{1}H NMR (500 MHz, $CDCl_3$) δ = 7.63 (s, 2H), 3.72 (s, 2H)

^{2}H NMR (92 MHz, $CHCl_3$) δ = 7.45 (s, 2D)

^{13}C NMR (126 MHz, $CDCl_3$) δ = 176.05, 138.24, 132.32, 130.0 (t, J = 24.5 Hz, 2C), 118.55, 111.51, 40.75.

HRMS (ESI^+) $C_9H_4D_2NO_2$ $[M - H]^-$ requires 162.0530; found 162.0522, Δ -4.74 ppm.

2-(((Phenyl-*d_5*)methyl)amino)cyclohex-2-en-1-one (**10a-*d_5***)

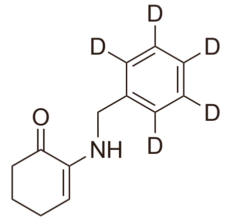

Prepared according to **General Procedure A** with 7-oxabicyclo[4.1.0]heptan-2-one (**9**) (1.15 g, 10.0 mmol, 1.00 equiv.) and (phenyl-*d_5*)methanamineone (**13**, 1.35 g, 12.0 mmol, 1.20 equiv.) for 21 h. Purification by flash column chromatography (9:1→4:1 v/v pentane:Et_2O) gave 2-(((phenyl-*d_5*)methyl)amino)cyclohex-2-en-1-one (**10a-*d_5***) as a yellow solid (753 mg, 3.65 mmol, 37%).

m.p. = 47-50 °C

IR (film) ν_{max}/cm^{-1}: 3408, 1660, 1620, 1487, 1371, 1357, 1348, 1256, 1205, 1167, 1135, 1127, 966, 889, 870, 840, 804, 723, 699, 635

^{1}H NMR (500 MHz, $CDCl_3$) δ = 5.43 (t, *J* = 4.7 Hz, 1H), 4.62 (s, 1H), 4.09 (s, 2H), 2.49 (dd, *J* = 7.3, 6.0 Hz, 2H), 2.34 (q, *J* = 5.6 Hz, 2H), 1.95 (dq, *J* = 7.9, 6.2 Hz, 2H).

^{2}H NMR (92 MHz, $CHCl_3$) δ = 7.53 – 7.20 (m, 5D)

^{13}C NMR (126 MHz, $CDCl_3$) δ = 195.9, 140.4, 138.9, 128.0 (t, *J* = 24.1 Hz, 2C), 127.0 (t, *J* = 23.5 Hz, 2C), 126.6 (t, *J* = 24.0 Hz), 111.8, 47.5, 37.9, 24.5, 23.5

HRMS (ESI^+) $C_{13}H_{11}D_5NO$ $[M+H]^+$ requires 207.1540; found 207.1540, Δ -0.26 ppm.

2-(4-Cyanophenyl-2,6-*d_2*)-*N*-(6-oxocyclohex-1-en-1-yl)-*N*-((phenyl-*d_5*)methyl)acetamideone (**1i-*d_7***)

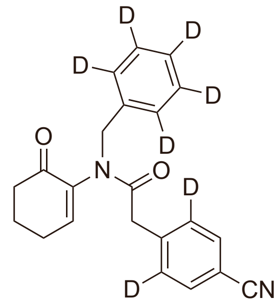

Prepared according to **General Procedure C** with 2-(4-cyanophenyl-2,6-*d_2*)acetic acid (**16**, 180 mg, 1.10 mmol, 1.10 equiv.), oxalyl chloride (190 µL, 2.20 mmol, 2.20 equiv.), DMF (2 drops) and CH_2Cl_2 (5 mL) followed by 2-(((phenyl-*d_5*)methyl)amino)cyclohex-2-en-1-one (**10a-*d_5***) (206 mg, 1.00 mmol, 1.00 equiv.), triethylamine (152 µL, 1.65 mmol, 1.10 equiv.) and CH_2Cl_2 (2.5 mL) for 18 h. Purification by flash column chromatography (2:1→1:1 v/v pentane 40–60:EtOAc) gave2-(4-cyanophenyl-2,6-*d_2*)-*N*-(6-oxocyclohex-1-en-1-yl)-*N*-((phenyl-*d_5*)methyl)acetamideone (**1i-*d_7***) as a white solid (189 mg, 0.54 mmol, 54%).

m.p. = 107-110 °C

IR (film) ν_{max}/cm^{-1}: 2226, 1688, 1658, 1636, 1430, 1405, 1365, 1340, 1318, 1265, 1233, 1189, 1153, 1127, 1055, 976, 918, 908, 853, 817, 772, 710

^{1}H NMR (500 MHz, $CDCl_3$) δ = 7.59 (s, 2H), 6.40 (d, *J* = 4.4 Hz, 1H), 5.31 (d, *J* = 14.4 Hz, 1H), 3.98 (d, *J* = 14.4 Hz, 1H), 3.54 (d, *J* = 15.4 Hz, 1H), 3.48 (d, *J* = 15.6 Hz, 1H), 2.48 (m, 2H), 2.33 (m, 2H), 1.99 (m, 2H)

^{2}H NMR (92 MHz, $CHCl_3$) δ = 7.49 – 7.10 (m, 9H)

^{13}C NMR (126 MHz, $CDCl_3$) δ = 194.9, 169.7, 150.3, 140.8, 138.6, 136.8, 132.1, 129.9 (t, J = 24.5 Hz), 128.7 (t, J = 24.3 Hz), 128.0 (t, J = 24.6 Hz), 127.1 (t, J = 24.0 Hz), 118.9, 110.6, 51.0, 40.8, 38.3, 25.9, 22.4

HRMS (ESI^+) $C_{22}H_{14}D_7N_2O_2$ $[M+H]^+$ requires 352.2037; found 352.2032, Δ -1.41 ppm.

Mechanistic Investigations

Supplemental Methods:

Stern-Volmer Quenching Experiment

A stock solution of Ir(dFppy)$_3$ (2.0 mg) in EtOAc (10.0 mL; 0.26 mM) was prepared by adding argon sparged EtOAc to a volumetric flask under argon containing the photocatalyst. A stock solution of substrate **1a** (10.3 mg) in EtOAc (2.0 mL; 20 mM) was prepared in a similar manner by adding argon sparged EtOAc to a vial containing substrate **1a** sealed with a septum under an atmosphere of argon.

Samples were prepared in 4.0 mL quartz cuvettes equipped with a septum under an argon atmosphere by adding 2.0 mL of the photocatalyst stock solution which was diluted with a further 1.0 mL of argon sparged EtOAc (final concentration of photocatalyst = 0.175 mM). The resulting solution was excited at 400 nm and three emission spectra recorded from 430 to 700 nm. To this solution was then added 50 µL of substrate **1a** stock solution under argon, and three emission spectra recorded as before. This process was then repeated five further times.

Figure S3 shows the stacked, averaged spectra which indicate a decrease in the intensity of the emission spectrum as more quencher (substrate **1a**) is added.

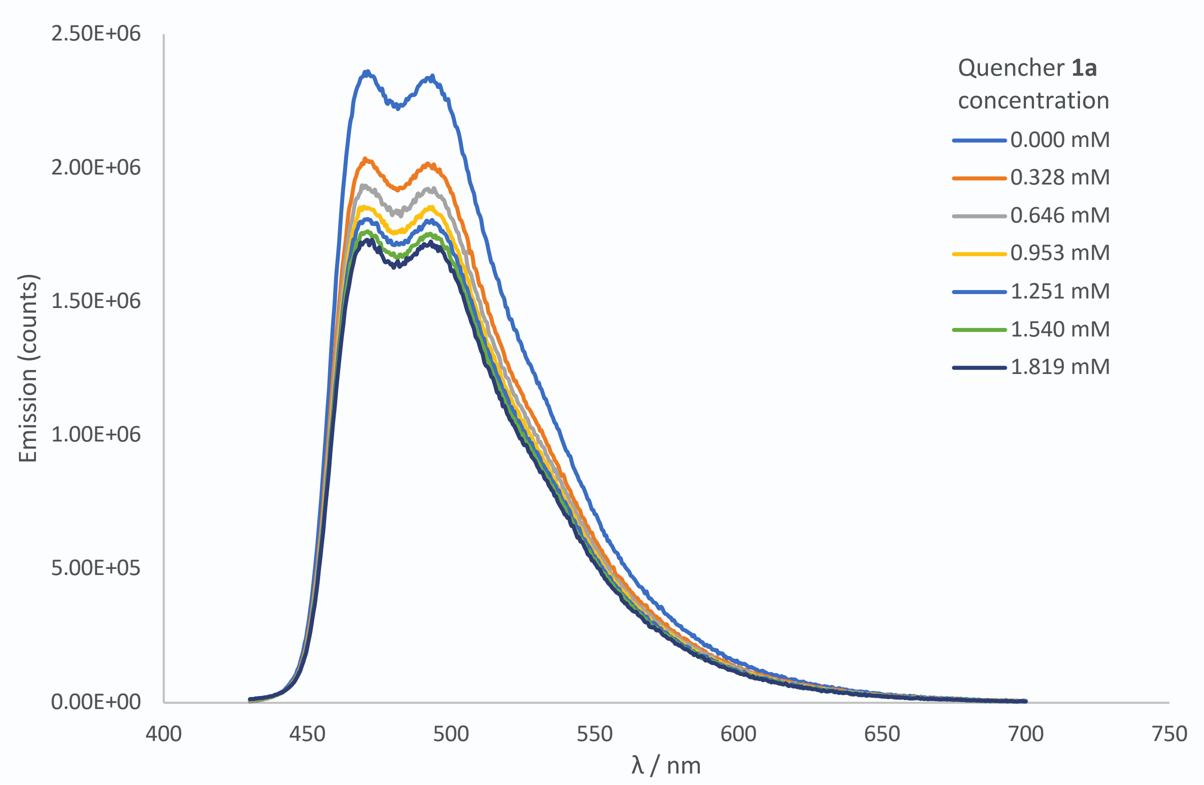

Figure S3. Emission spectra of photocatalyst Ir(dFppy)$_3$ on addition of increasing amounts of substrate **1a**.

By using the Stern-Volmer relationship:

$$\frac{I_0}{I} = k_q[Q] + 1$$

Where I_0 is the emission of the photocatalyst in the absence of quencher, and I is the emission of the photocatalyst at a known quencher concentration, a graph plotting (I_0/I) against quencher

concentration ([Q]) should give a straight line with y intercept of 1 and a gradient quantifying the rate of quenching (k_q), and is shown in **Figure S4**.

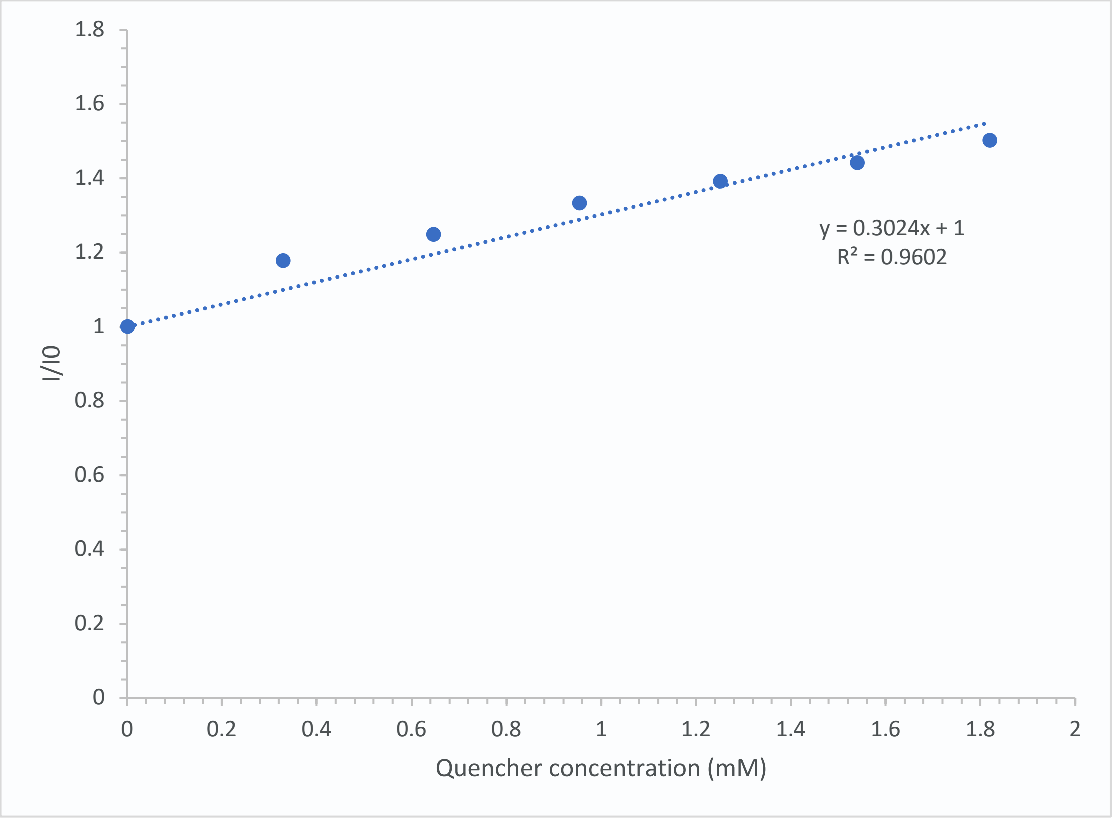

Figure S4. Stern-Volmer plot of I_0/I against quencher concentration.

Deuterium labelling experiments

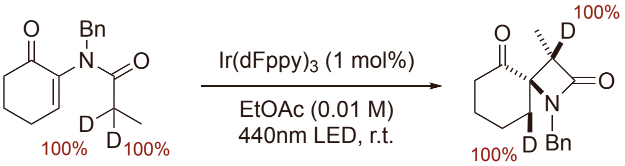

1a-*d*₂ **2a-*d*₂**

Figure S5. Photocyclization of deuterated substrate **1a-*d*₂**.

Deuterated substrate **1a-*d*₂** was subjected to the photocyclization conditions as outlined in **Figure S5**, and the NMR spectra compared with the corresponding non-deuterated compound. In the ^{1}H NMR of deuterated compound **2a-*d*₂,** the signals corresponding to H_{15} and one of H_4 were not present, and the doublet at 1.20 ppm in the protic compound was present as a broad singlet instead (**Figure S6**). In the ^{13}C NMR of deuterated compound **2a-*d*₂**, the signals corresponding to C_4 and C_{15} were not present or significantly weakened in intensity, further supporting the incorporation of deuterium at those carbon atoms (**Figure S7**).

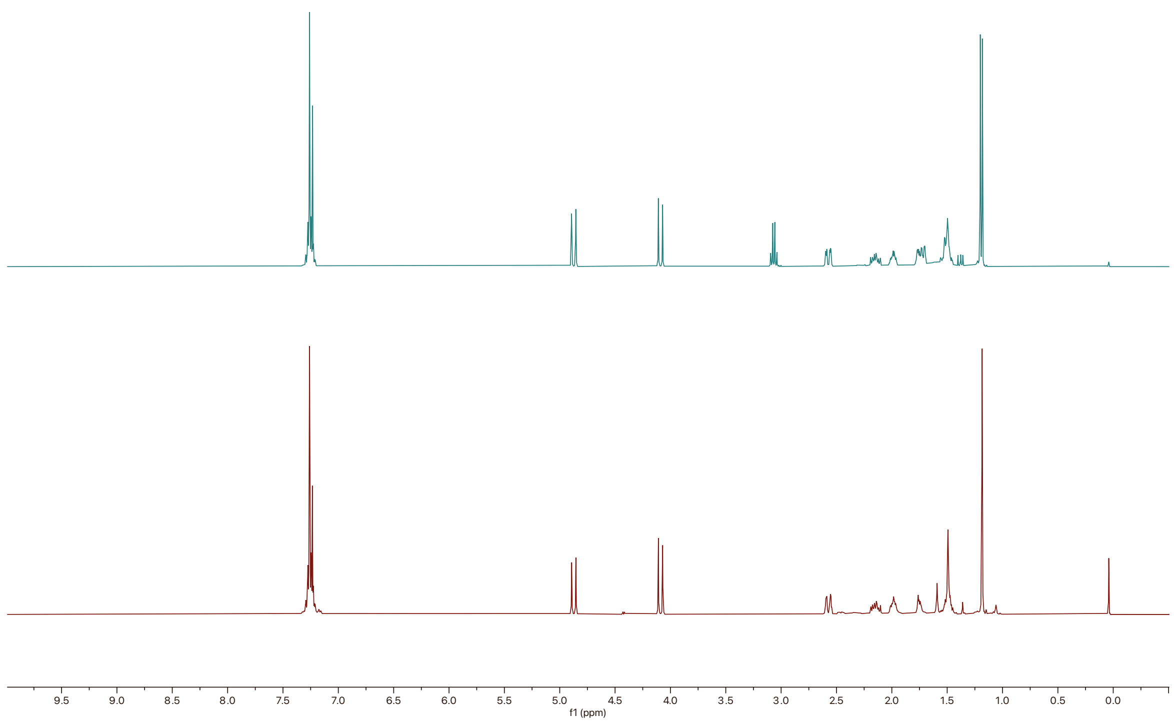

Figure S6. Stacked ^{1}H NMR spectra of compound **2a** (teal) and deuterated analogue **2a-*d*$_2$** (red).

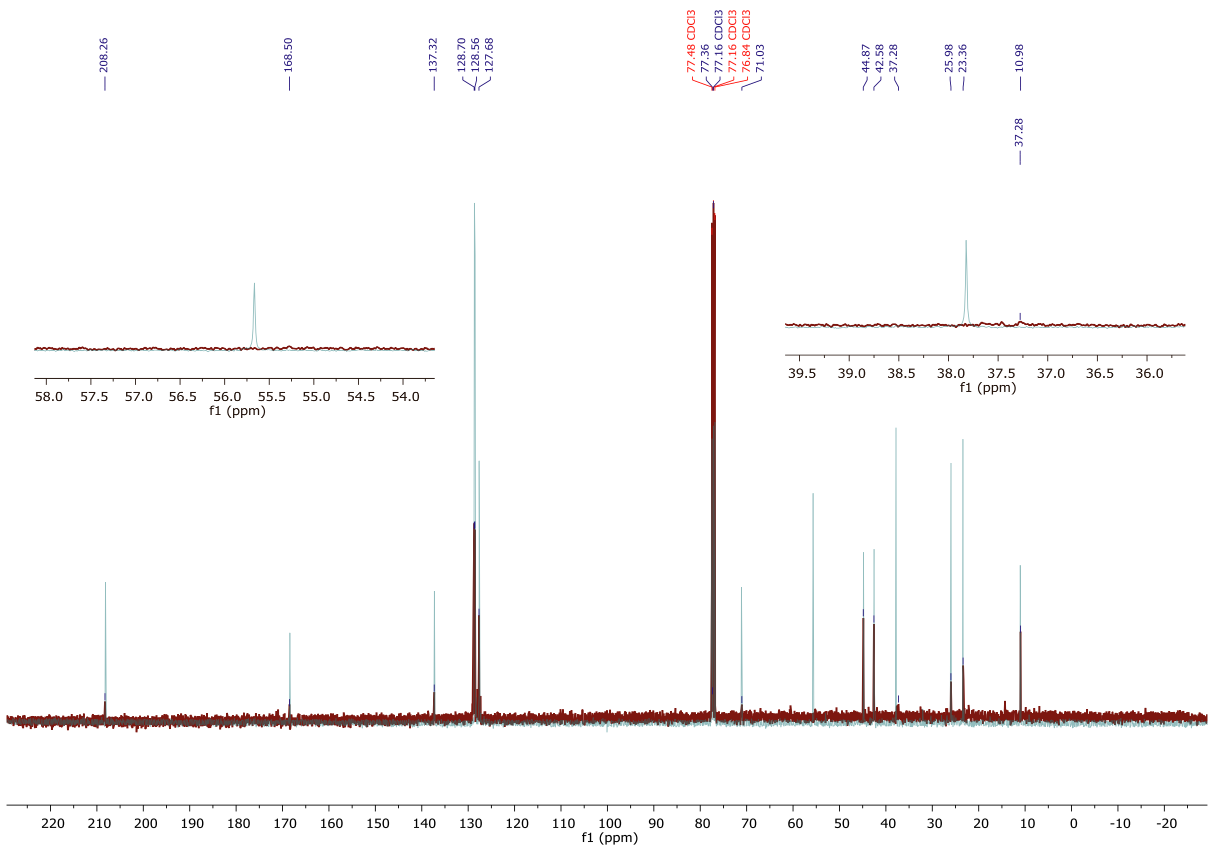

Figure S7. Superimposed ^{13}C NMR spectra of protic compound **2a** (blue) and deuterated **2a-*d*$_2$** (red).

Crossover Experiments.

LCMS analysis was carried out using a Waters Xevo G2-XS QTOF, with a Waters Acquity front end equipped with a TUV detector. Separation was carried out by gradient elution (1 minute dwell at 5% MeCN/H_2O, followed by a gradient to 95% MeCN/H2O over 7 minutes) using an Agilent Poroshell 120 EC-C18 USP L1 Solvent Saver 3.0 x 100 mm column at a flow rate of 0.4 mL min^{-1}. 0.1% Formic acid was added to all solvents.

1. Control reactions.

(i) Enone **1i** is converted to β-lactam **2i** upon irradiation in the presence of Ir(dfppy)$_3$ under our standard reaction conditions. Mass spectrometry confirms the isotopic distribution of the product to be in line with natural abundance (**Figure S8**).

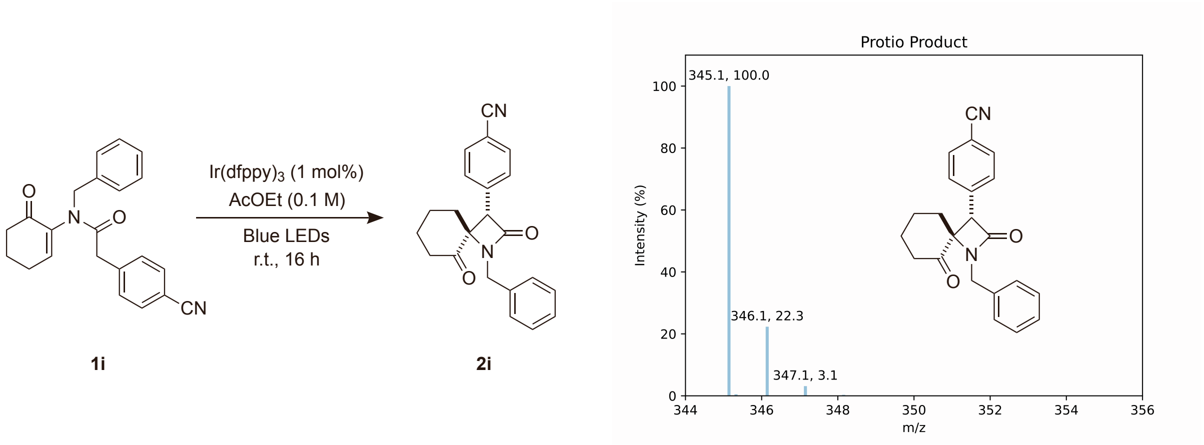

Figure S8. LCMS analysis showing isotopic distribution of d_0 product **2i**.

(ii) Enone **1i**-d_7 was converted to β-lactam **2i**-d_7 upon irradiation in the presence of Ir(dfppy)$_3$ under our standard reaction conditions. Mass spectral analysis shows an isotopic distribution in line with the material being close to 100% d_7 labelled. A small amount of d_6-labelled material present (~6%) gives rise to an M^{-1} mass of 351.2 (**Figure S9**).

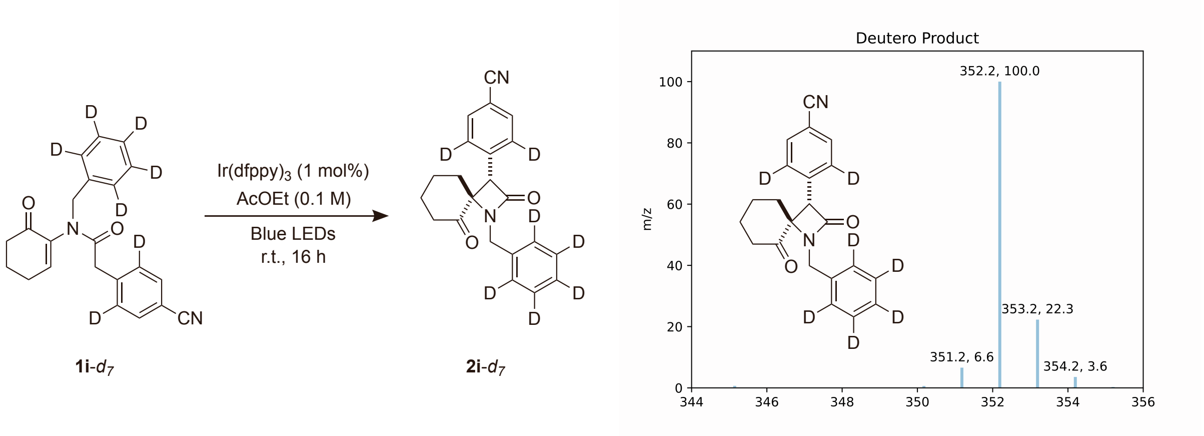

Figure S9. LCMS analysis showing isotopic distribution of **2i**-d_7.

2. Crossover reactions.

(i) **Predicted result for an exclusively intramolecular reaction.** If β-lactam products are formed only *via* a unimolecular cyclisation, then a mixture comprising equimolar amounts of **1i** and **1i**-d_7 should give rise to only two products when subjected to our standard reaction conditions: **2i** and **2i**-d_7. If this is the case, then we would expect mass spectrometric analysis of the reaction mixture by LC-MS to

show an isotopic distribution which is a simple combination of both of the isotopic distributions previously recorded for single component reactions above (**Figure S10**).

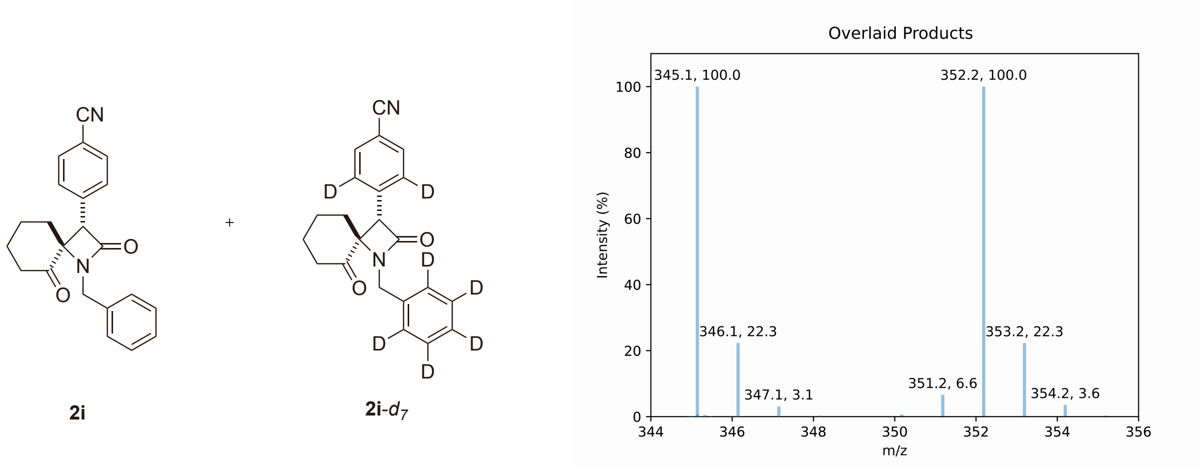

Figure S10: Expected LCMS spectrum of a 1:1 mixture of **2i** and **2i**-d_7, showing isotopic distribution of d_0 and d_7 compounds.

(ii) **Predicted result for a mechanism that involves fragmentation and recombination.** Our DFT studies indicate that zwitterionic intermediates may cyclize directly to β-lactam products or fragment to form ketenes and imines, which can either recombine immediately, undergo alternate reaction, or dissociate and then recombine. Low yields indicate that alternate reaction pathways may be consuming imines and ketenes.

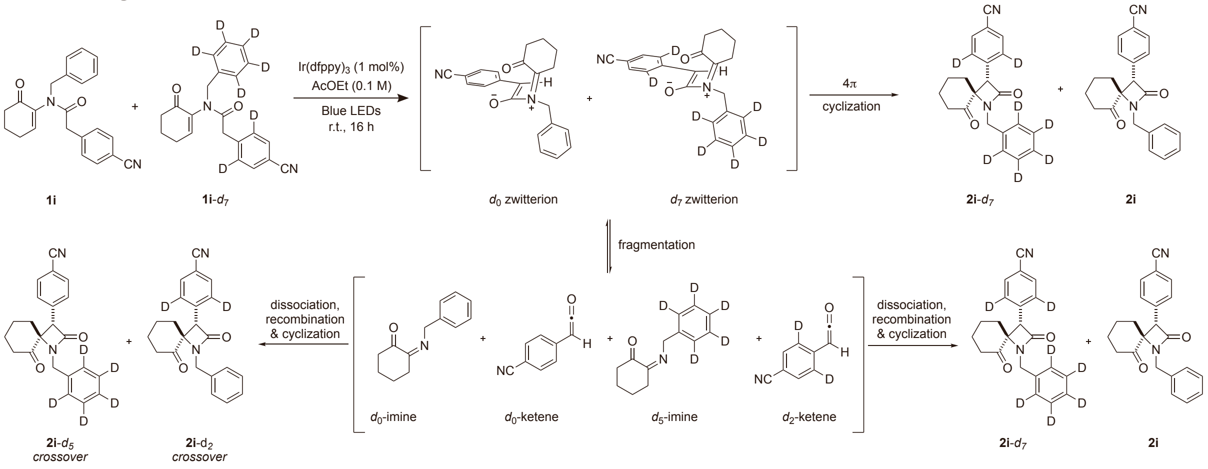

Figure S11: Proposed mechanism involving fragmentation of zwitterions into ketene and imine components and subsequent intermolecular recombination to crossover products.

In the reaction scheme above (**Figure S11**), the fragmentation of d_0 and d_7 zwitterions leads to d_0 and d_5 imines, and d_0 and d_2 ketenes. When these species persist long enough to dissociate, it is just as likely that they will encounter an isotopically labelled species as it is that they will encounter an unlabelled species[1]. This means that recombination can give rise the "standard" **2i**-d_7 and **2i** products, alongside crossed **2i**-d_5 and **2i**-d_2 products. As these are distinct species with different masses, their presence can be detected by LC-MS analysis.

[1] This assumes that fragmentation and dissociation of d_0 and d_7 zwitterions occur at similar rates and that there is no significant KIE for the reactions of deuterated vs undeuterated ketenes and imines

(iii) **Crossover reaction with an equimolar ratio of d_0 and d_7 substrates**

A 1:1 mixture of **1i**-d_7 and **1i** were irradiated in the presence of $Ir(dfppy)_3$ under our standard reaction conditions and the crude reaction mixture was analysed by LC-MS (**Figure S12a**).

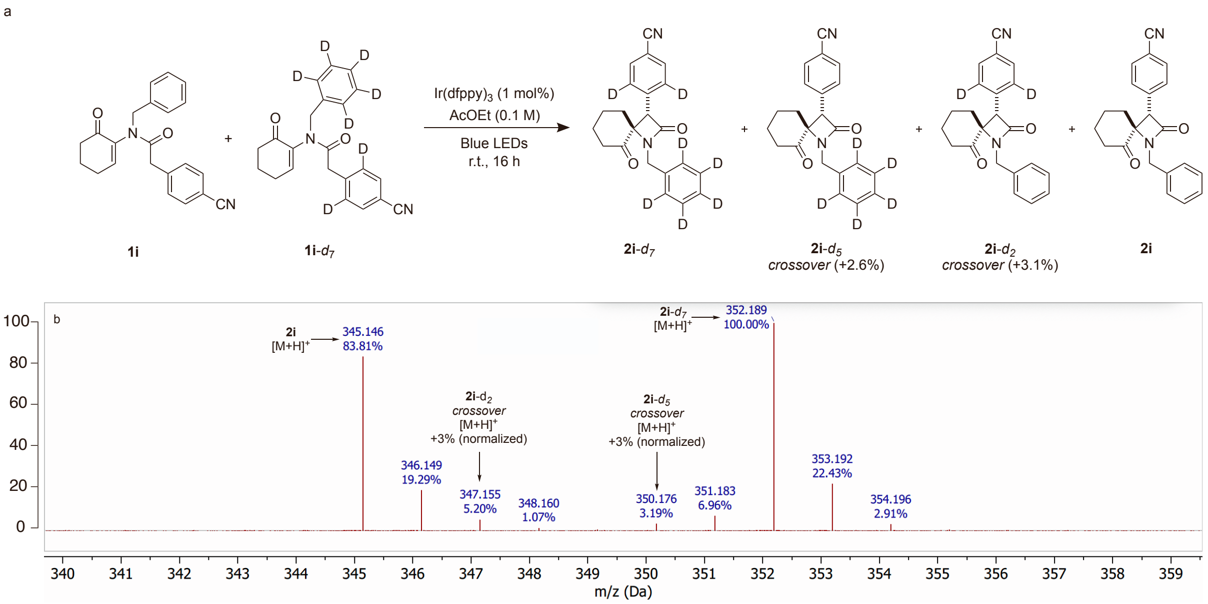

Figure S12: Results of crossover experiments. a) Crossover experiment with **1i** and **1i**-d_7 substrates. b) LCMS (TOF) spectra indicating the presence of "standard" **2i** and **2i**-d_7 products, alongside **2i**-d_2 and **2i**-d_5 crossover products.

Comparing[2] this with the distribution we expect if there is no crossover shows that two additional masses are recorded (348.2 Da (**2i**-d_5 $[(M+1)+H]^+$)) and 350.2 Da (**2i**-d_5 $[M+H]^+$)) and others are enhanced – 347.2 Da (**2i**-d_5 $[M+H]^+$) and 351.2 Da (**2i**-d_5 $[(M+1)+H]^+$), **Figure S12b**.

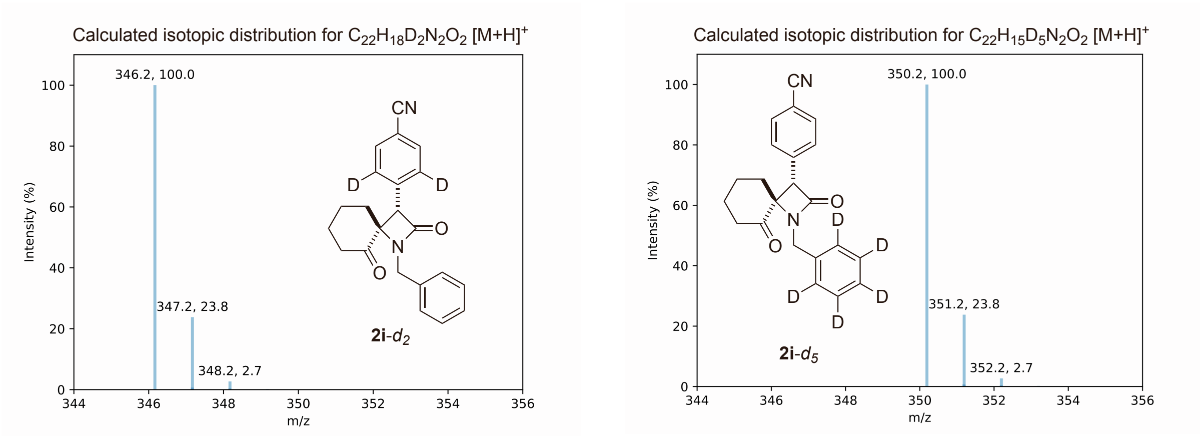

Figure S13: Calculated isotope distributions for **2i**-d_2 $[(M+H)^+]$ and **2i**-d_5 $[(M+H)^+]$ crossover products.

Calculation of the theoretical distributions of **2u**-d_2 and **2s**-d_5 crossed products (**Figure S13**) show that these additional masses indeed correspond to these products. This is consistent with the formation of ketene and imine formation and subsequent recombination to β-lactam products under the reaction conditions and accounts for approximately 3%[3] of the total yield in the case of this specific substrate.

[2] The distribution of the protio product has been scaled to 100% based on the M+1 peak (345.1 Da) to allow comparison.

[3] This figure can be calculated by comparing the abundance of crossed peaks with the abundance of non-crossed peaks. i.e. non-crossed peaks total = 183.8, crossed peaks total = 3.2 + 2.6 = 5.8. For a percentage, 5.8/(183.8 + 5.8) = 3.06%. The figure

Computational Studies

Computational details

Supplemental Methods:

The hybrid meta-GGA M06-2X density functional[9] was used in combination with the 6-31+G(d,p)[10] basis to optimize the geometries of all stationary points. Electronic energies were refined with single point energy calculations at the M06-2X/def2-TZVP[11] level. Previous work from our group has shown that M06-2X in conjunction with triple-ζ basis sets perform well in the study of triplet state transformations; as well as in the prediction of radical properties.[12] Solvent effects were considered using the integral equation formalism variant of the polarizable continuum model (CPCM, solvent = ethylethanoate).[13] Solvents effects were considered in all the calculations unless otherwise specified. For all ground state singlet intermediates and transition states, open shell biradical structures were localized using the broken symmetry formalism and corrections were applied using the Yamaguchi spin projection method. The quasi-harmonic approximation was used to obtain corrections to Gibbs energies using the *GoodVibes*[14] program (see the *Thermochemical data* section). *Gaussian*[15] was employed for all density functional theory (DFT) calculations, using the g09defaults keyword and an “ultrafine” pruned (99,590) grid for numerical integration of the exchange-correlation functional and its derivatives. Additionally, intrinsic reaction coordinate (IRC) calculations[16] were performed to identify which intermediates were connected to the respective transition structures (TSs) on the minimum energy path. IRC calculations were unsuccessful for TSs involving rotations (i.e. **^{3}TS-II**); instead we confirmed the connection between energy minima and TSs using molecular dynamics simulations. For all elementary reaction steps, a manual conformational search was performed. Minimum energy crossing points were localized using MECPro and rates of Intersystem Crossing were evaluated using the Excited State Dynamics (ESD) module implemented in ORCA 5.0.3 (see *ISC rate calculations* section).[17] AQME[18] (Automated Quantum Mechanical Environments) was used to ensure that there were no errors in the Gaussian output files (command line: python -m aqme --qcorr --files "*.log") and to generate inputs for single-point energy calculations for closed-shell singlets and triplets at the optimized stationary points (command line: python -m aqme --qprep --files "*.log" --qm_input "m062x/def2tzvp scrf=(cpcm,solvent=ethylethanoate)" --suffix TZ --program gaussian --mem 16GB --nprocs 8). PyMol[19] was used to create molecular graphics; Our display settings have been made openly accessible.[20] Finally, we used the “stable=opt” option[21] to ensure that all reported energies correspond to species without wavefunction instabilities.

Gaussian 09 optimization (keywords line):

```
# opt freq 6-31+g(d,p) scrf=(cpcm,solvent=ethylethanoate) m062x
```

* The “g09defaults” and “int=grid=ultrafine” keyword was added when using Gaussian 16 to run jobs.

Gaussian 16 single-point energy calculation (keywords line):

```
# def2tzvp scrf=(cpcm,solvent=ethylethanoate) int=grid=ultrafine m062x
```

AQME error check (terminal command):

```
python -m aqme --qcorr --files "*.log"
```

AQME automated imput file generation (terminal command):

```
python -m aqme --qprep --files "*.log" --qm_input "m062x/def2tzvp scrf=(cpcm,solvent=ethylethanoate)" --suffix TZ --
program gaussian --mem 16GB --nprocs 8
```

of 2.6 arises from the 347.2 abundance figure of 5.2, minus the expected overlapping M+2 peak from the protio product (main peak 345.1; 0.838*3.1 (measured natural M+2 abundance) = 2.6).

Nomenclature

Supplemental Methods:

We use prefixes to differentiate the different substrates studied in this work, containing two parts that detail enolate and *N*-amino substituents (**Figure S14**). For most reaction steps, multiple conformers were obtained that lead to analogous transition structures or products. In the *Thermochemistry.dat* file, a suffix is included to separate the different conformers. The multiplicity is included before the name of the species (i.e., ^{1}**B** and ^{3}**B** correspond to the **B** intermediate in singlet and triplet state, respectively). Singlet diradical species contain the suffix "dirad" (i.e., **Me-NMe-^{1}TS-III-dirad_a**).

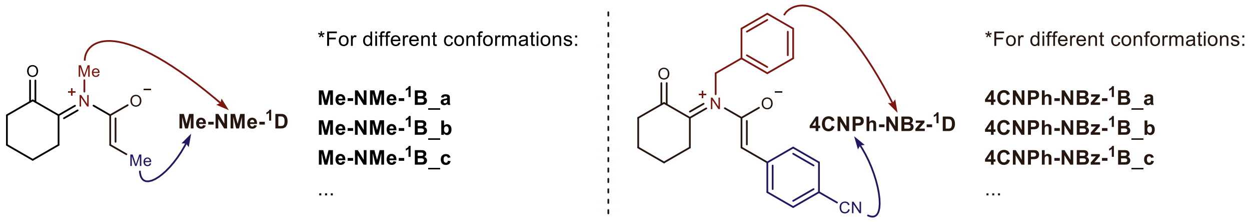

Figure S14. Nomenclature used to differentiate multiple substrates and conformers.

Competitive reaction pathways for Me-NMe substrate 1l

Supplemental Methods:

A computational mechanistic study was conducted for the **Me-NMe** substrate **1l**. The competitive pathways for each of the two possible tautomeric forms are shown in **Figures S13-S16**. The reaction steps of the ketone tautomer (**Figures S15-S16**) had significantly lower activation barriers than the enol tautomer (**Figures S17-S18**). These calculations were performed at the M06-2X/def2-QZVPP//M06-2X/6-31+G(d,p) (CPCM included in all calculations) level of theory, which yields results similar to the standard level of theory used in the main text (TZ vs QZ; see the *Benchmarking* section).

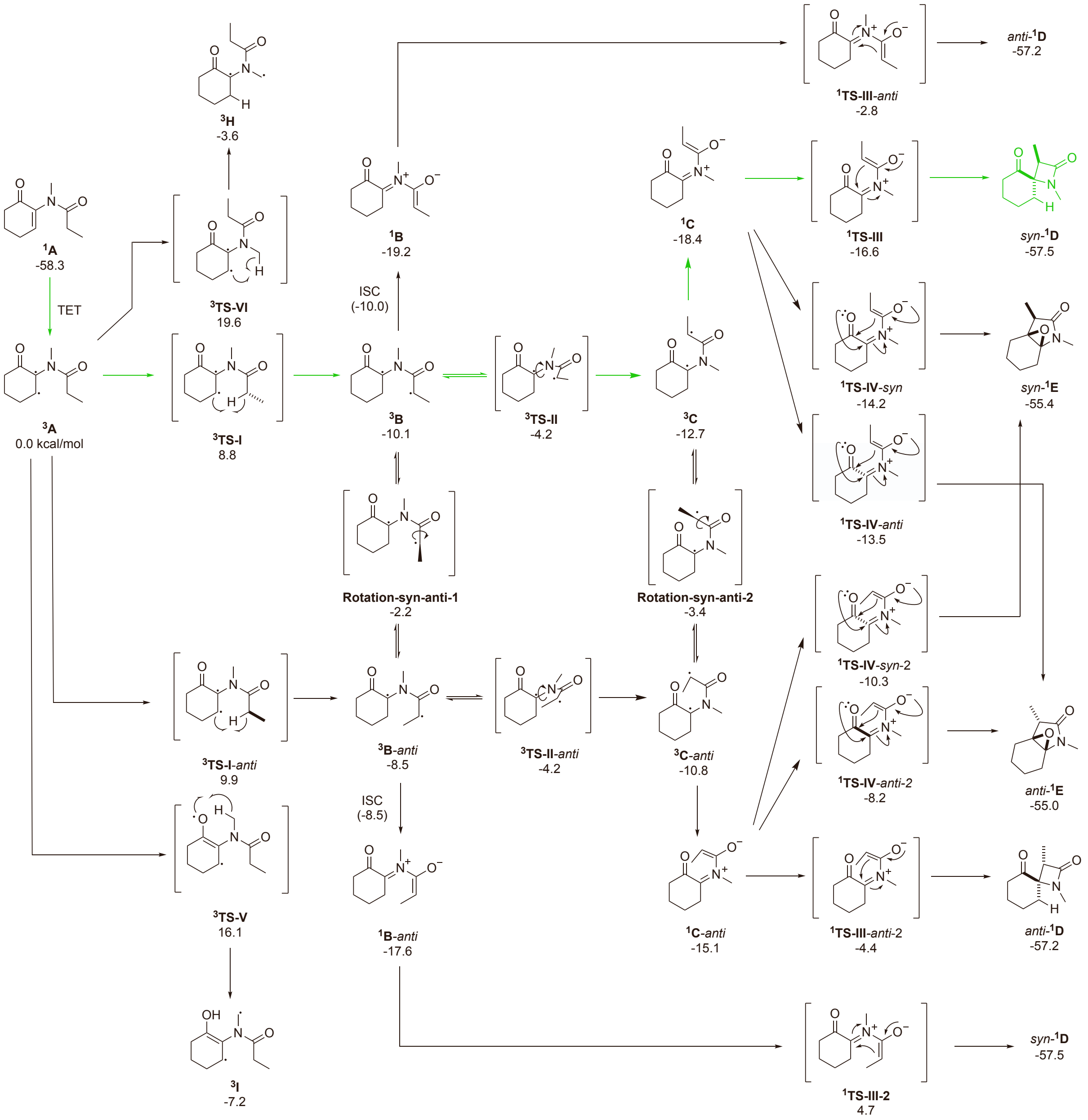

Figure S15. Boltzmann weighted Gibbs energies relative to ^{3}**A** (kcal/mol) of reaction pathways of the ketone tautomer.

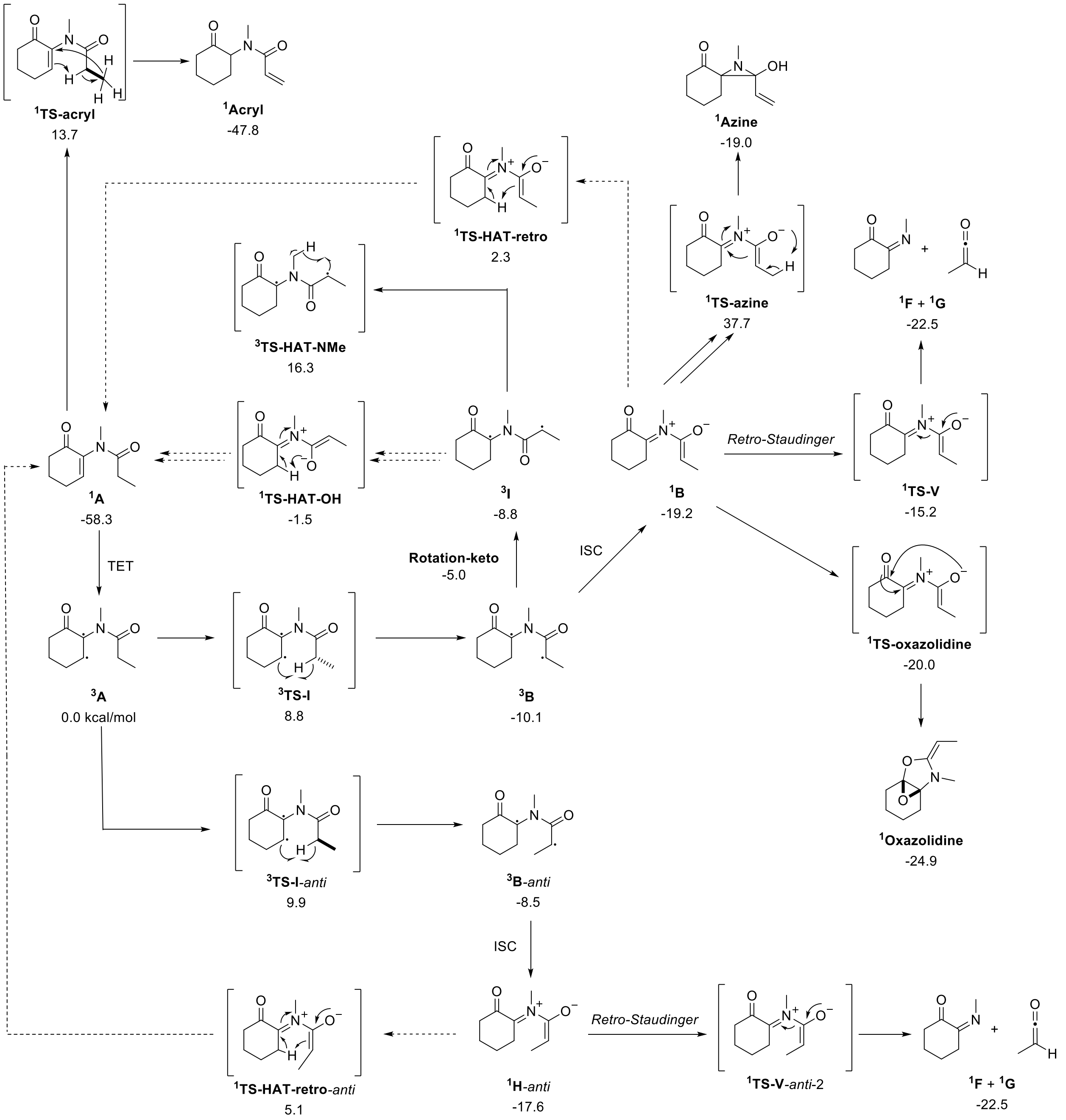

Figure S16. Additional reaction pathways of the ketone tautomer: Gibbs energies relative to ^{3}**A** (kcal/mol).

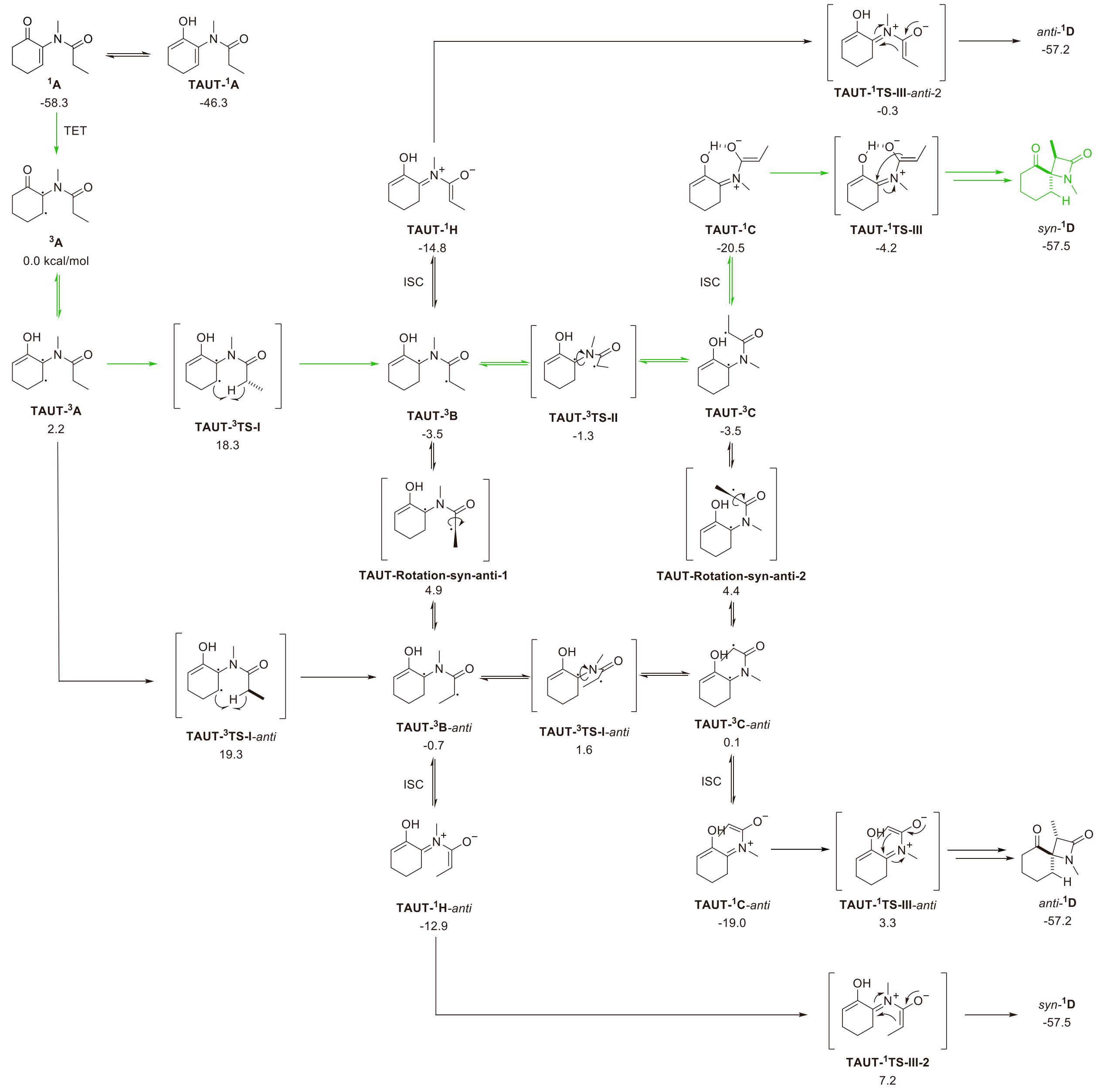

Figure S17. Boltzmann weighted Gibbs energies relative to ^{3}**A** (kcal/mol) of reaction pathways of the enol tautomer.

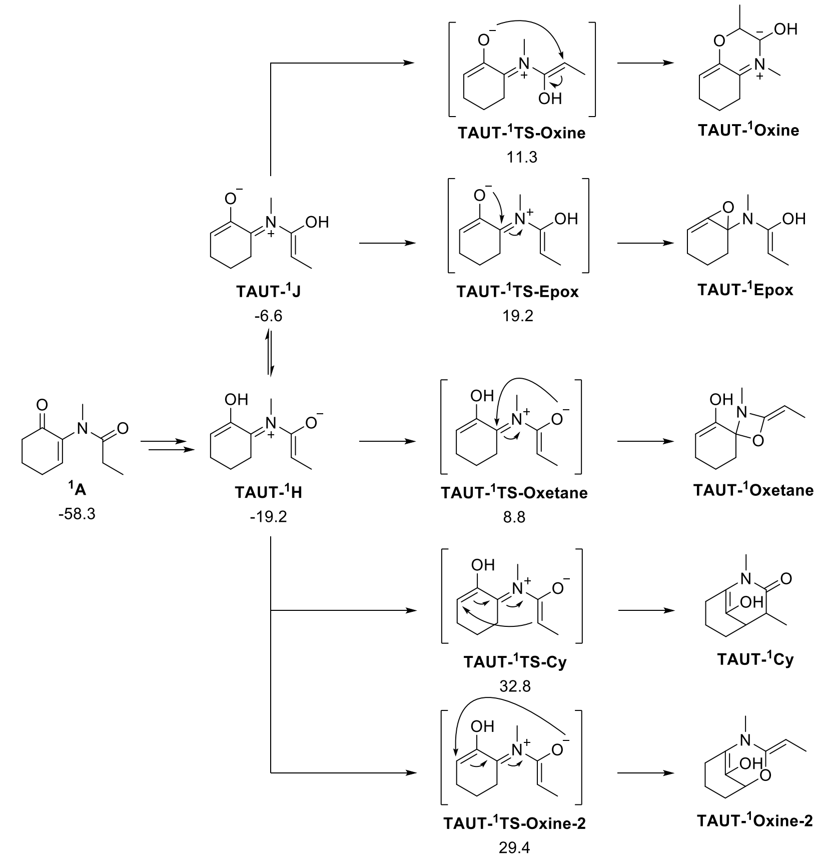

Figure S18. Additional reaction pathways of the enol tautomer: Gibbs energies relative to **^{3}A** (kcal/mol).

Benchmarking Studies

Supplemental Methods:

Geometries were optimized using the M06-2X/6-31+G(d,p) method and calculated the Gibbs free energy (G) of various key reaction steps using M06-2X single-point energy corrections obtained with different basis sets, empirical dispersion corrections, and implicit solvation models. **Figure S19** shows that the variations in Gibbs energies obtained are very small (0.5 kcal/mol or lower in most cases), suggesting that our results are converged with respect to basis set size and that alternative dispersion and solvation protocols yield very similar results. Optimization at the wB97x-D/6-31+G(d,p) and B3LYP-D3(BJ)/6-31+G(d,p) provided very similar results to M06-2X/6-31+G(d,p). Similar results were obtained when optimization with different implicit solvation models (CPCM, IEFPCM and SMD) were carried out.

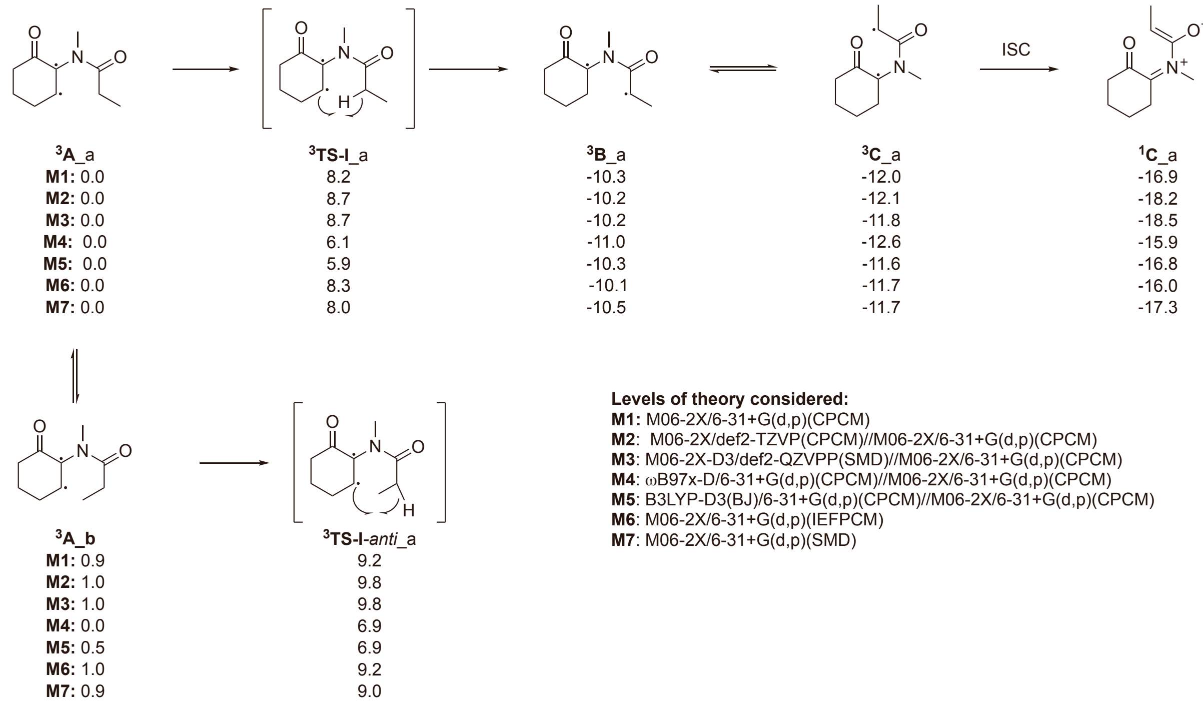

Figure S19. Gibbs energies (kcal/mol) for selected conformers at different levels of theory.

Minimum Energy Crossing Points (MECPs) between singlet and triplet states

Supplemental Methods:

Geometries of MECPs were obtained using Ess's MECPro interfaced with *Gaussian*.[22] An example input is shown below:

```
%mem = 48gb
%nprocshared = 24

[general]
method = 6-31+g(d,p) scrf=(cpcm,solvent=ethylethanoate) um062x g09defaults int=grid=ultrafine
spinstates = 1/3
show_hessian = none
read_later = False
charge = 0

!This job has a max steps of 100. If it fails to converge, resubmit the new mecp file created after the job
max_steps = 100
max_stepsize = 0.1

[route]
A: force integral=ultrafinegrid scf=qc guess=mix
B: force integral=ultrafinegrid scf=qc

[geometry]
0 1/3
MOLECULAR COORDINATES
```

In all cases, the structure and energy of intermediates **Me-NMe-^{3}B** and **Me-NMe-^{3}C** are similar to those found for the corresponding MECPs that lead to **^{1}B** and **^{1}C** (**Table S2** and **Figure S20**).

System	E (^{3}**B**/^{3}**C**) (a.u.)	E (MECP) (a.u.)	ΔE (kcal/mol)
**^{3}B_a**	-595.198893	-595.198691	0.1
**^{3}B_b**	-595.198701	-595.196589	1.3
**^{3}C_a**	-595.202633	-595.202572	< 0.1
**^{3}C_b**	-595.203135	-595.203087	< 0.1

Table S2. Electronic energies of **^{3}B** and **^{3}C** and their connected MECPs at the M06-2X/def2-TZVP level.

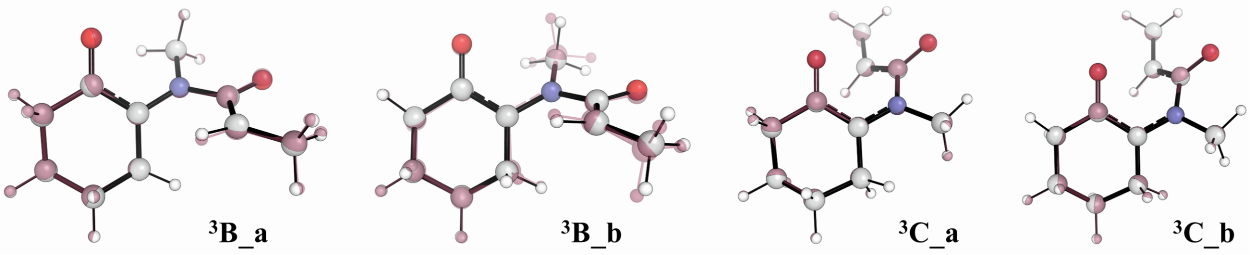

Figure S20. Overlay of the MECP geometries, represented in dark red, with geometries of their corresponding triplet intermediate, represented with standard colours.

Hammett data

Table S3. Hammett *σ* constants and ΔG‡ values of [1]**TS-III**, [1]**TS-IV** and [1]**TS-V** for multiple substrates. ΔG‡ values were calculated relative to the corresponding [1]**C** intermediates.

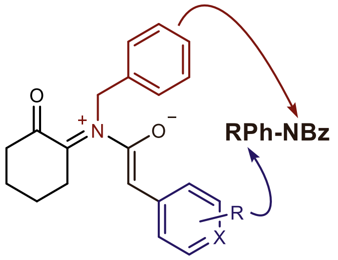

Substrate	σ+	ΔG‡ (kcal/mol)		
		[1]**TS-III**	[1]**TS-IV**	[1]**TS-V**
**4CNPh-NBz**	0.66	5.7	6.6	5.2
**4CF$_3$Ph-NBz**	0.54	5	6.1	5.4
**Ph-NBz**	0	2.2	5.5	4.6
**4OMePh-NBz**	-0.27	-0.2	5.1	5.3
**4BrPh-NBz**	0.23	3.6	5.7	5.5
**4MePh-NBz**	-0.17	1.2	4.9	5
**3FPh-NBz**	0.34	4	6.1	5.2

Table S3. Raw data reported in **Figure 6C(i)** of the main manuscript.

Spin analysis of ^{1}C and ^{1}TS-III

Supplemental Methods:

During the optimization of ^{1}**C**, we found different conformers, some of which were closed-shell singlets and others open-shell diradical singlets. The closed-shell zwitterionic species were more stable than their diradical analogs, which lie 4.3 - 4.7 kcal/mol higher than the most stable closed-shell conformer (**Figure S21**). Electronic structures of the diradicals were characterized by high values of the expectation value of the total spin, $\langle S^2 \rangle$ (approximately 0.9) observed in the broken-symmetry calculations before spin annihilation. However, spin contamination was substantially reduced after spin annihilation, following which the $\langle S^2 \rangle$ values were less than 10% of the initial value. The absence of wavefunction instability was confirmed for all species.

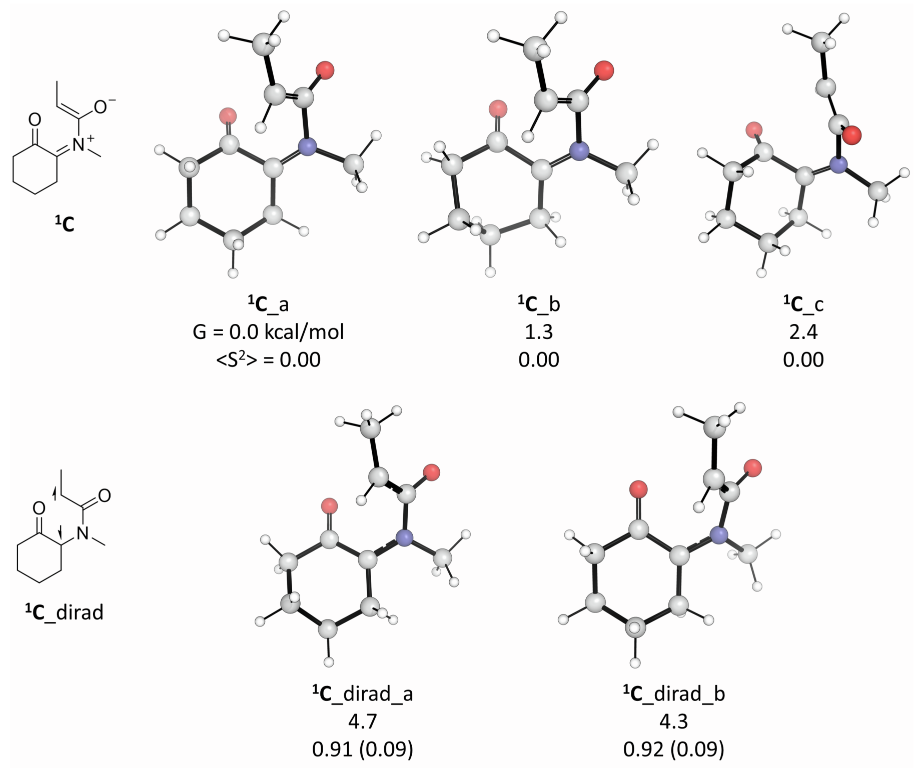

Figure S21. Relative Gibbs energies (kcal/mol) of **Me-NMe-^{1}C** conformers calculated as closed-shell and diradical singlets (M06-2X/def2-TZVP//M06-2X/6-31+G(d,p). Yamaguchi spin corrections are included. The $\langle S^2 \rangle$ values obtained in uM06-2X/def2-TZVP broken-symmetry calculations are also included along with the values after spin annihilation (in parentheses).

We also found different conformers of ^{1}**TS-III** as closed-shell and diradical singlets. In this case, the diradical species were considerably more stable than the zwitterions (3.9 - 4.2 kcal/mol higher than the most stable diradical conformer, **Figure S22**).

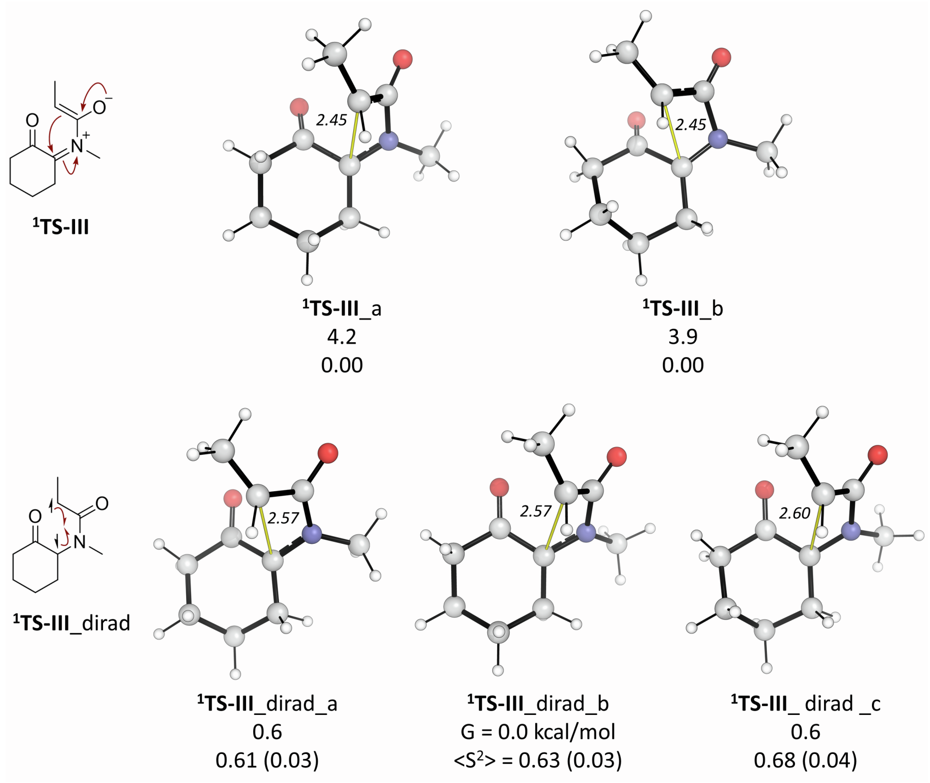

Figure S22. Relative Gibbs energies (kcal/mol) of **Me-NMe-¹TS-III** conformers calculated as closed-shell and diradical singlets (M06-2X/def2-TZVP//M06-2X/6-31+G(d,p)). Yamaguchi corrections are included. The ⟨S^2⟩ values obtained in the broken-symmetry calculations with M06-2X/def2-TZVP are also included along with the values after spin annihilation (in parentheses).

Correlation between thermochemistry with electronic parameters

Supplemental Methods:

The Gibbs energy differences (ΔG) between ^{1}**C** and the products of ^{1}**TS-III** and ^{1}**TS-V** were computed for substrates with electronically varying aromatic rings. As seen in Figure S23, electronic effects influence ΔG in products *syn*-^{1}**D** more than in products ^{1}**G** (slope of 4.96 vs 2.71, respectively).

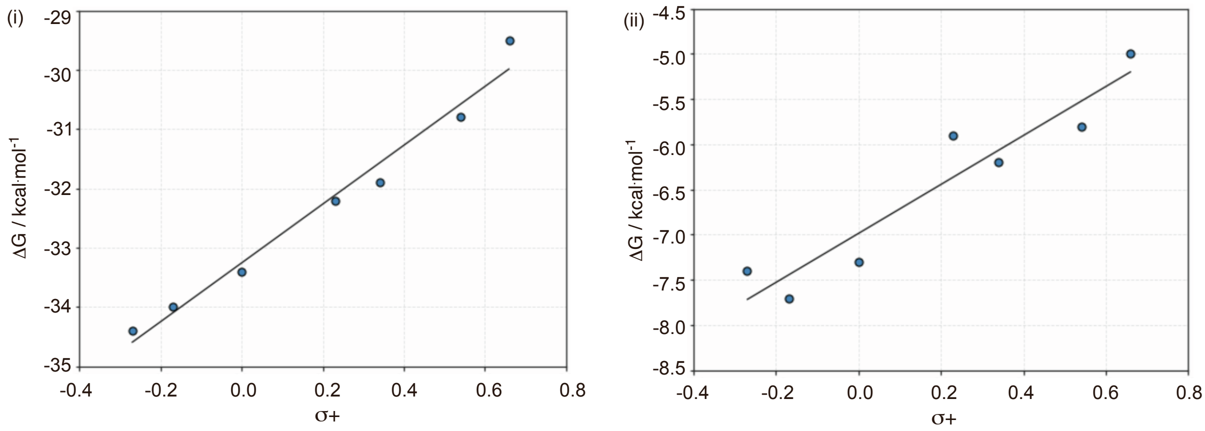

Figure S23. ΔG between ^{1}**C** and the products of (i) ^{1}**TS-III** (*syn*-^{1}**D**) and (ii) ^{1}**TS-V** (^{1}**F** + ^{1}**G**).

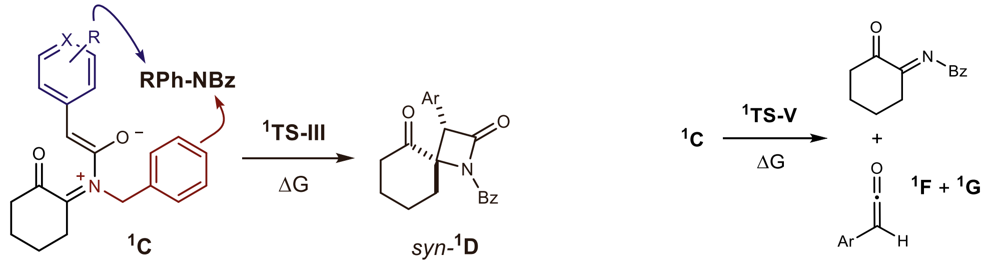

Substrate	σ+	ΔG (*syn*-^{1}**D**)	ΔG (^{1}**F** + ^{1}**G**)
**4CNPh-NBz**	0.66	-29.5	-5.0
**4CF$_3$Ph-NBz**	0.54	-30.8	-5.8
**Ph-NBz**	0.00	-33.4	-7.3
**4OMePh-NBz**	-0.27	-34.4	-7.4
**4BrPh-NBz**	0.23	-32.2	-5.9
**4MePh-NBz**	-0.17	-34.0	-7.7
**3FPh-NBz**	0.34	-31.9	-6.2

Table S4. Raw data presented in Figure S23 (ΔG in kcal/mol).

ISC rate calculations

Supplemental Methods:

Calculations of Intersystem Crossing (ISC) rates were performed using the Excited State Dynamics (ESD) module implemented in ORCA 5.0.3. that evaluates the Fermi golden rule equation using a quantum electrodynamics treatment. The ISC rates were evaluated for the lowest energy conformers of intermediates **^{3}B** and **^{3}C**. The structures were reoptimized using the ORCA package at the CAM-B3LYP[23]/def2-TZVP, LC-BLYP[24]/def2-TZVP and ωB97x[25]/def2-TZVP levels of theory using the SMD solvation model for ethyl ethanoate in both the corresponding S_0 and T_1 states, which were used to assess the adiabatic S_0-T_1 gap, spin orbit couplings (SOCs) and Hessians needed for rate calculations.

For the ISC rate calculations, the ESD implementation for ISC "ESD(ISC)" was used at 298 K for a total of 655360 points. Contributions from the Herzberg-Teller effect was accounted for by evaluating the derivatives of the SOCMEs across the vibrational normal modes using the "DOHT TRUE" keyword. Duschinsky rotations were also computed using the "USEJ TRUE" keyword. The rate of ISC was evaluated for each subspin level of the T_1 state (-1, 0, 1) and the effective ISC rate was determined by summing together all rate for each subspin level (**Table S5**):

Level of Theory	Entry		$^{3}B \rightarrow {}^{1}B$	$^{3}C \rightarrow {}^{1}C$
CAM-B3LYP/def2-TZVP	k(ISC) / s	-1	$3.47 \cdot 10^{3}$	$1.79 \cdot 10^{5}$
		0	$9.86 \cdot 10^{3}$	$1.20 \cdot 10^{5}$
		1	$3.47 \cdot 10^{3}$	$1.79 \cdot 10^{5}$
	Effective k(ISC) / s		$1.68 \cdot 10^{4}$	$4.78 \cdot 10^{5}$
	$t_{1/2}$ / μs		41.3	1.5
LC-BYLP/def2-TZVP	k(ISC) / s	-1	$6.86 \cdot 10^{3}$	$4.86 \cdot 10^{5}$
		0	$1.51 \cdot 10^{4}$	$1.85 \cdot 10^{5}$
		1	$6.86 \cdot 10^{3}$	$4.86 \cdot 10^{5}$
	Effective k(ISC) / s		$2.88 \cdot 10^{4}$	$1.16 \cdot 10^{6}$
	$t_{1/2}$ / μs		24.1	0.6
ωB97x/def2-TZVP	k(ISC) / s	-1	$3.74 \cdot 10^{3}$	$4.12 \cdot 10^{4}$
		0	$1.90 \cdot 10^{3}$	$2.12 \cdot 10^{4}$
		1	$3.74 \cdot 10^{3}$	$4.12 \cdot 10^{4}$
	Effective k(ISC) / s		$9.39 \cdot 10^{3}$	$1.04 \cdot 10^{5}$
	$t_{1/2}$ / μs		73.8	6.7
Average Predicted $t_{1/2}$ / μs			46.4	2.9

Table S5: Compiled rates of ISC for intermediates **^{3}B** and **^{3}C**.

An example of an ESD input file using ORCA can be found below:

```
!pal16 def2-tzvp ESD(ISC) cam-B3LYP
%maxcore 4000
%ESD ISCISHESS "T1.hess"
        ISCFSHESS "S0.hess"
        TEMP 298
        DELE 2998.68
        USEJ TRUE
        DOHT TRUE
        NPOINTS 655360
END
%cpcm smd true
SMDsolvent "Ethyl Ethanoate"
END
%TDDFT NROOTS 1
```

```
        TROOT 1
        SROOT 0
        DOSOC TRUE
        TROOTSSL 1
END

* XYZ 0 1

[coordinates]
*
```

Molecular dynamics (MD) simulations

Supplemental Methods:

The *Jprogdyn*[26] program was used to perform molecular dynamics trajectories, interfaced to *Gaussian 16*. MD trajectories were run starting from **Me-NMe-³TS-I_a** to estimate the lifetime of rotation **³TS-II.** In total, 100 trajectories were considered, leading to the results gathered in **Table S6** and **Figure S24**. The rotation lifetime might be longer in certain cases, since 10 of the trajectories remained as intermediate **³B** during the time of the simulations (1000 fs). However, most of the trajectories in the direction of **³B** crossed **³TS-II** to form **³C** in less than 600 fs. The CPCM implicit solvation model was employed in the simulations instead of explicit solvent molecules. This approach assumes that the energy exchange between solute and ethyl acetate is negligible since the time scale of the reactive steps is considerably inferior to previously measured solute-solvent energy exchanges (on the order of 10 to a few hundred picoseconds).[27]

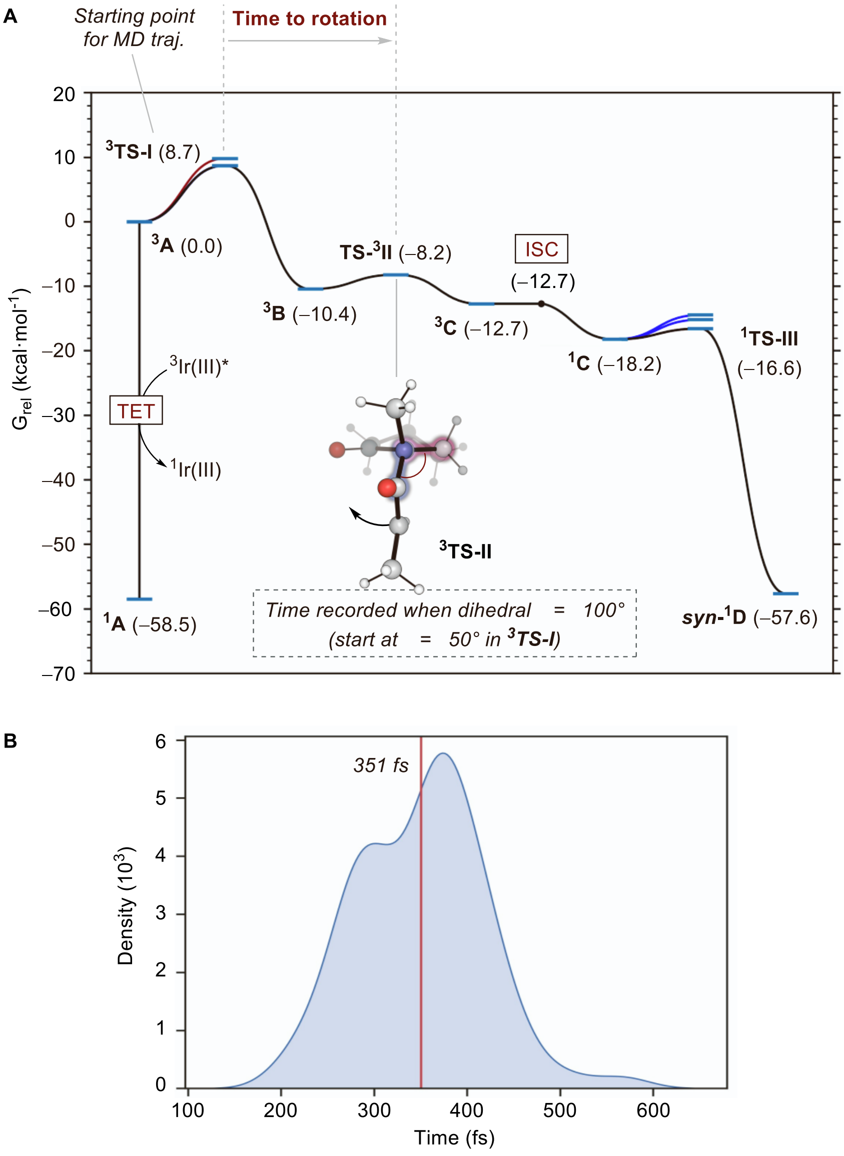

Figure S24. Molecular dynamics simulation results. (A) Detailed description regarding lifetime calculations. (B) Density of lifetimes measured.

Output of the trajectory	Frequency
Recrossing to ^{3}**A**	13
Change to the *anti* pathway	0
Remain as ^{3}**B** after 1000 fs	10
^{3}**C** is formed through ^{3}**TS-II** rotation (successful trajectories)	77

Table S6: Outcomes from the 100 MD trajectories.

The setting input for *Jprogdyn* dynamic calculations can be found below:

```
### Jprogdyn Configuration File ###

# File/Directory Locations
working_directory                    : use_current

frequency_directory                  : STATIC_FILES

frequency_file                       : Me-NMe-3TS-I_a.log

gaussian_directory                   : gaussian

gaussian_max_filenames               : 10000

# Threading Options
number_of_simultaneous_trajectories     : 4

# Gaussian Options
number_of_processors_per_trajectory     : 4

memory_per_trajectory                : 8

# the route card to pass to Gaussian for a regular force job
gaussian_force_route_card            : um062x/6-31+g(d,p) scrf=(cpcm,solvent=ethylethanoate) g09defaults
int=grid=ultrafine pop=none

gaussian_force_footer                : @blank

# Trajectory Options
job_type                             : trajectory

trajectory_type                      : reaction

number_of_total_trajectories         : 4

checkpoint_directory                 : checkpoints

checkpoint_prefix                    : Me-NMe-3TS-I

checkpoint_interval                  : 5

temperature                          : 298.15

timestep                             : 1.0

number_of_forward_points             : 1000

number_of_backward_points            : 1

# Initialization Options
maximum_number_of_initialization_attempts   : 50
```

```
harmonic_tolerance                    : 0.0005

scale_factor                          : 1.0

# draw the initial displacements from...
vibrational_initialization_default       : quasiclassical

vibrational_initialization_override      : 0:ts_negative

rotational_initialization_type           : classical

# Reaction Trajectory Termination Conditions
termination_condition : no_termination_conditions

# Analysis Options
# Note: these options will only be processed if job_type is set to "analysis."
analysis_directory                    : analysis

# MOLDEN movies to
make_molden_movies                    : yes

summarize_trajectories_to_screen         : yes

summary_interval                      : 20

# place multiple analysis_coordinate entries on separate lines

analysis_coordinate                   : bond_length, 3, 24, C-H-bond
analysis_coordinate                   : torsion, 3, 4, 15, 20, dihedral

write_analysis_csv                    : yes
```

Yamaguchi spin corrections of ^{1}TS-III

Supplemental Methods:

Yamaguchi spin corrections[28] were applied to the electronic energies of broken-symmetry calculations of ^{1}**TS-III** with non-zero ⟨S^2⟩. The energy values including Yamaguchi corrections (E^1_{corr}) were calculated using equation 1:

$$E^1_{corr} = \alpha E^1_{BS} - \beta E^3 \qquad (1)$$

where E^1_{BS} is the energy of the broken-symmetry singlet calculations, E^3 is the energy of the triplet state calculated with the geometry of the broken-symmetry calculations, and α and β are:

$$\alpha = \frac{\langle S^2\rangle^3 - \langle S^2\rangle^1_{exact}}{\langle S^2\rangle^3 - \langle S^2\rangle^1_{BS}} = \frac{\langle S^2\rangle^3}{\langle S^2\rangle^3 - \langle S^2\rangle^1_{BS}} \qquad (2)$$

$$\beta = \alpha - 1 \qquad (3)$$

where $\langle S^2\rangle^3$ and $\langle S^2\rangle^1_{BS}$ are the ⟨S^2⟩ values of the triplet and broken-symmetry singlet calculations, respectively, before spin annihilation, and $\langle S^2\rangle^1_{exact}$ is the exact ⟨S^2⟩ value of a singlet state, which is 0. The Yamaguchi corrections correspond to the difference between the corrected and raw energies (E^1_{corr} and E^1_{BS}). All the data used to calculate these corrections are shown in **Table S7**.

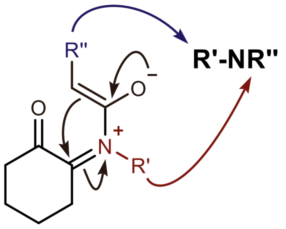

Substrate	E^1_{BS}	E^3	$\langle S^2\rangle^1_{BS}$	$\langle S^2\rangle^3$	E^1_{corr}	Yamaguchi correction
**Me-NMe_a**	-595.201093	-595.182934	0.6073	2.0205	-595.208897	-0.007804
**Me-NMe_b**	-595.20153	-595.183578	0.6299	2.0198	-595.209666	-0.008136
**Me-NMe_c**	-595.200496	-595.184604	0.6835	2.0205	-595.208620	-0.008124
**3FPh-NBz_a**	-1117.240209	-1117.225888	0.656	2.0359	-1117.247017	-0.006808
**3FPh-NBz_b**	-1117.237872	-1117.225808	0.7183	2.0358	-1117.244449	-0.006577
**3FPh-NBz_c**	-1117.237988	-1117.22555	0.6906	2.0362	-1117.244372	-0.006384
**3FPh-NBz_d**	-1117.239859	-1117.226188	0.6636	2.0363	-1117.246468	-0.006609
**3FPh-NBz_e**	-1117.237635	-1117.226159	0.7322	2.0358	-1117.244081	-0.006446
**3FPh-NBz_f**	-1117.237773	-1117.22634	0.7188	2.0362	-1117.244011	-0.006238
**3FPh-NBz_g**	-1117.239636	-1117.226537	0.6808	2.0364	-1117.246214	-0.006578
**3Py-NBz_a**	-1034.028229	-1034.014232	0.6703	2.036	-1034.035099	-0.006870
**3Py-NBz_b**	-1034.025857	-1034.014933	0.7454	2.0356	-1034.032168	-0.006311
**3Py-NBz_c**	-1034.025943	-1034.015789	0.7551	2.0359	-1034.031929	-0.005986
**3Py-NBz_d**	-1034.027823	-1034.015068	0.6945	2.0363	-1034.034425	-0.006602
**3Py-NBz_e**	-1034.026893	-1034.014912	0.7195	2.0355	-1034.033443	-0.006550
**3Py-NBz_f**	-1034.027009	-1034.015807	0.728	2.0359	-1034.033244	-0.006235
**3Py-NBz_g**	-1034.028897	-1034.015741	0.6819	2.0361	-1034.035522	-0.006625
**4BrPh-NBz_a**	-3591.584465	-3591.572456	0.7183	2.0367	-3591.591008	-0.006543
**4BrPh-NBz_b**	-3591.582087	-3591.571813	0.758	2.0365	-3591.588178	-0.006091
**4BrPh-NBz_c**	-3591.582226	-3591.572075	0.7533	2.0368	-3591.588184	-0.005958
**4BrPh-NBz_d**	-3591.584119	-3591.572917	0.7311	2.037	-3591.590390	-0.006271
**4CF3Ph-NBz_a**	-1355.079711	-1355.066259	0.6774	2.0369	-1355.086414	-0.006703
**4CF3Ph-NBz_b**	-1355.07736	-1355.066843	0.753	2.0367	-1355.083529	-0.006169
**4CF3Ph-NBz_c**	-1355.077456	-1355.067462	0.7517	2.037	-1355.083301	-0.005845
**4CF3Ph-NBz_d**	-1355.079337	-1355.067119	0.7021	2.0373	-1355.085762	-0.006425
**4CNPh-NBz_a**	-1110.239892	-1110.226493	0.6694	2.0406	-1110.246433	-0.006541

**4CNPh-NBz_b**	-1110.237573	-1110.227668	0.7634	2.0403	-1110.243495	-0.005922
**4CNPh-NBz_c**	-1110.237639	-1110.228164	0.7551	2.0405	-1110.243205	-0.005566
**4CNPh-NBz_d**	-1110.239496	-1110.227482	0.6987	2.0409	-1110.245750	-0.006254
**4MePh-NBz_a**	-1057.298188	-1057.283448	0.6435	2.0362	-1057.304999	-0.006811
**4MePh-NBz_b**	-1057.295822	-1057.283952	0.7221	2.036	-1057.302346	-0.006524
**4MePh-NBz_c**	-1057.296004	-1057.283401	0.6941	2.0364	-1057.302521	-0.006517
**4MePh-NBz_d**	-1057.297887	-1057.284406	0.6725	2.0365	-1057.304534	-0.006647
**4OMePh-NBz_a**	-1132.517097	-1132.503399	0.6641	2.0361	-1132.523727	-0.006630
**4OMePh-NBz_b**	-1132.514645	-1132.502329	0.7029	2.0359	-1132.521139	-0.006494
**4OMePh-NBz_c**	-1132.514867	-1132.501971	0.6805	2.0363	-1132.521340	-0.006473
**4OMePh-NBz_d**	-1132.516798	-1132.503322	0.6659	2.0364	-1132.523346	-0.006548
**Ph-NBz_a**	-1017.986645	-1017.972213	0.6564	2.0358	-1017.993513	-0.006868
**Ph-NBz_b**	-1017.984298	-1017.972349	0.7222	2.0356	-1017.990868	-0.006570
**Ph-NBz_c**	-1017.984454	-1017.972759	0.7197	2.036	-1017.990848	-0.006394
**Ph-NBz_d**	-1017.98633	-1017.973011	0.6795	2.0361	-1017.993001	-0.006671

Table S7. E^1_{BS}, E^3, $\langle S^2\rangle^1_{BS}$, $\langle S^2\rangle^3$, E^1_{corr} and Yamaguchi corrections (in a.u.) of **^{1}TS-III** steps calculated with M06-2X/def2-TZVP.

Dynamic Vertical Triplet Energy Analysis

Supplemental Methods:

Calculation of dynamic vertical triplet energies has been performed using the recently dissolved protocol.[29] A total of 50 quasi-classical initiate ab-initio molecular dynamics trajectories (298K, 1 fs timestep, 500 fs simulation length) were collected for system ^{1}**A** (**1l**) using the MILO[30] package at the M06-2X/MIDI![31] basis set in conjunction with the CPCM solvation model for ethyl ethanoate. For every 8th frame, a vertical S_0-T_1 gap measured collected at the M06-2X/6-31G(d) (CPCM=AcOEt) level of theory, leading to a total of 3125 datapoints (**Figure S25**). The resulting distribution was found to pass the quartile-quartile normality test (**Figure S26**). The resulting triplet energy of 62.9 kcal/mol was determined using the previously empirically determined 0.8% population of the fitted gaussian distribution (μ = 79.0, σ = 6.3).

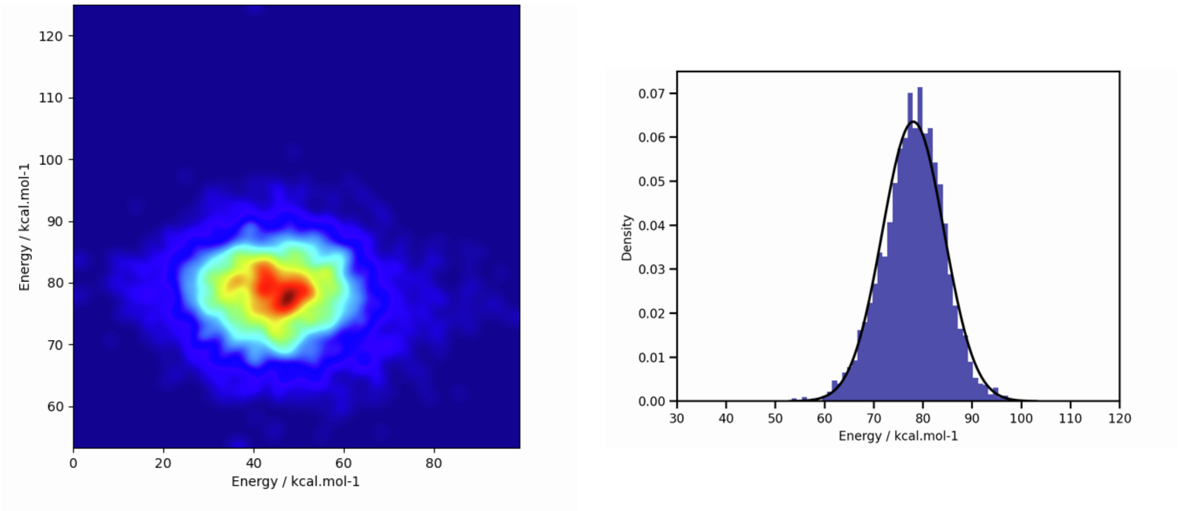

Figure S25. (left) Heatmap of kinetic energy versus vertical S_0-T_1 gap; (right) Histogram of vertical S_0-T_1 gaps.

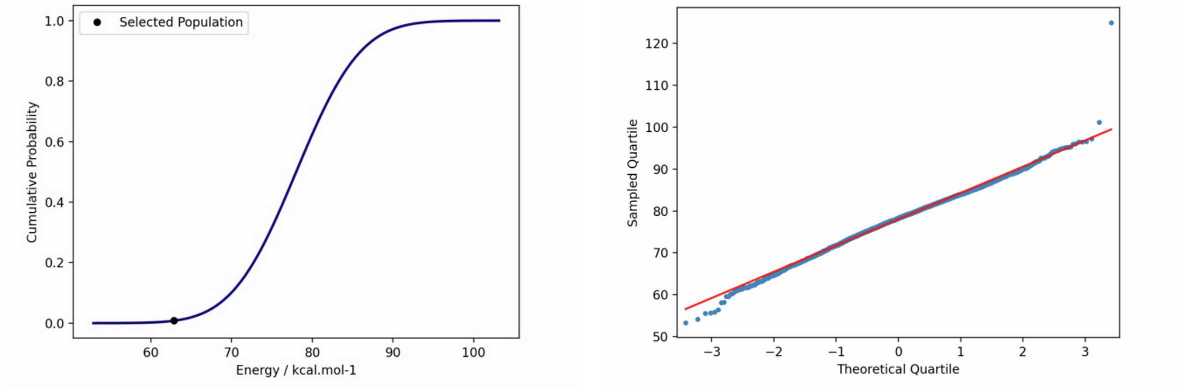

Figure S26. (left) Cumulative normal distribution of vertical S_0-T_1 gaps, with the highlighted 0.8% population; (right) Quartile-quartile normality test plot.

Thermochemical data

Supplemental Methods:

Using the *GoodVibes* program, quasi-harmonic (QHA) corrections were introduced to the computed vibrational entropies using a frequency cut-off value of 100.0 cm^{-1}, following the model proposed by Grimme[32] at the temperature indicated in the figures or tables. Also, a correction for the change in standard state from gas phase at 1 atm to a 1 M solution was introduced (option "-c 1" in *GoodVibes*). A few of the calculations showed low imaginary frequencies lower than 50 cm^{-1}. Imaginary frequencies lower than 10 cm^{-1} were detected and inverted to their respective positive values before the QHA entropic corrections were computed as seen in previous examples (option "--invertifreq -10").[33] After all geometry optimizations were done, all duplicated geometries were excluded from the study (duplicate detection with the "--check" option of *GoodVibes*).

Entropy corrections due to entropy of symmetry (option "--ssym"), mixing, and multi-structural effects (option "--pes") were also included. Boltzmann weighted G (G_{av}) were also calculated with *GoodVibes* (option "--pes").[34]

The Yamaguchi corrections (see the *Yamaguchi corrections* section) were applied to the electronic energies by individually modifying the raw outputs from *Gaussian* before running *GoodVibes*. The corrections were indicated in the DFT *Thermochemistry.dat* file by adding the suffix "Yamaguchi_corr" to calculations that included these corrections.

All the thermochemical data including absolute energies, zero-point energies (ZPE) and T·S, among other parameters, at the M06-2X/6-31+G(d,p) level, as well as the absolute and relative G including the M06-2X/def2-TZVP single point energy corrections (option "--spc"), were generated in an automated way using *GoodVibes* and tabulated in a separate file of the ESI called *DFT Thermochemistry.dat*. Additionally, the "--check" option of *GoodVibes* was employed to detect any potential input errors in the calculations (i.e. always using same level of theory, program version and solvation model, geometries of single-point corrections match their corresponding optimizations, etc.). This process for creating G profiles in an automated manner provides a useful method to avoid errors related to human manipulation of the data. The keyword input line used in *GoodVibes* is also included in the document uploaded with the ESI.

Example GoodVibes thermochemistry:

```
python -m goodvibes *.log --c 1 --spc TZ --check --csv
```

Molecular coordinates

An xyz file containing all the geometries studied is provided separately along with the ESI. The creation of the xyz files was automated with the *GoodVibes* software (option "--xyz").

Example GoodVibes xyz generation:

```
python -m goodvibes *.log --xyz
```

X-ray Crystallographic Data

Crystal data for (3*S**,4*S**)-1-benzyl-3-(*p*-tolyl)-1-azaspiro[3.5]nonane-2,5-dione, **2c**

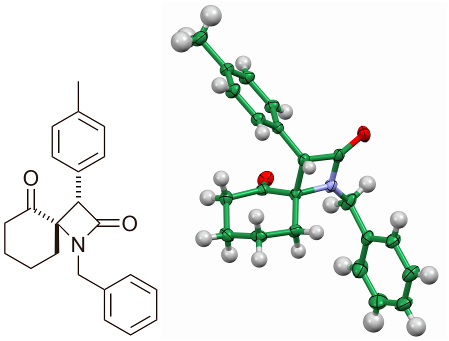

Identification code	CCDC 2033320	
Empirical formula	C_{22} H_{23} N_1 O_2	
Formula weight	333.43	
Temperature	150K	
Wavelength	1.54180 Å	
Crystal system	Orthorhombic	
Space group	P b c a	
Unit cell dimensions	a = 18.8707(3) Å	α = 90°
	b = 8.72890(10) Å	β = 90°
	c = 21.5772(3) Å	γ = 90°
Volume	3554.21(9) $Å^3$	
Z	8	
Density (calculated)	1.25 Mg m^{-3}	
Absorption coefficient	0.624 mm^{-1}	
F(000)	1424.000	
Crystal size	0.19 × 0.22 × 0.30 mm	
Theta range for data collection	4.686 ° to 76.120°	
Index ranges	-21≤h≤23, -9≤k≤10, -17≤l≤26	
Reflections collected	12038	
Independent reflections	3667 [R(int) = 0.0278]	
Completeness to θ = 76.120°	98.9%	
Absorption correction	Multi-scan	
Refinement method	Full-matrix least squares on F^2	
Goodness-of-fit on F^2	0.9772	
Final R indices [I>2σ(I)]	R^1 = 0.0398, wR^2 = 0.1027	
R indices (all data)	R^1 = 0.0467, wR^2 = 0.1108	

Crystal data for (3*S**,4*S**)-1-benzyl-3-(4-methoxyphenyl)-1-azaspiro[3.5]nonane-2,5-dione, **2b**

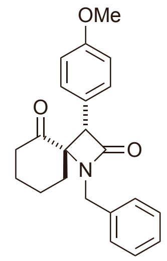

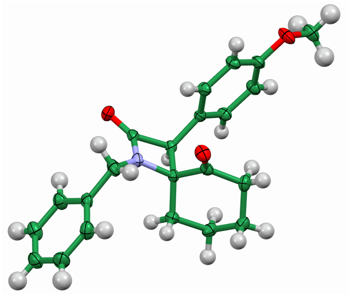

Identification code	CCDC 2033323	
Empirical formula	$C_{22}\ H_{23}\ N_1\ O_3$	
Formula weight	349.43	
Temperature	150K	
Wavelength	1.54180 Å	
Crystal system	Orthorhombic	
Space group	P b c a	
Unit cell dimensions	a = 19.4408(2) Å	α = 90°
	b = 8.60420(10) Å	β = 90°
	c = 21.4307(3) Å	γ = 90°
Volume	3584.77(7) $Å^3$	
Z	8	
Density (calculated)	1.29 Mg m^{-3}	
Absorption coefficient	0.687 mm^{-1}	
F(000)	1488	
Crystal size	0.15 × 0.16 × 0.20 mm	
Theta range for data collection	4.549 ° to 76.126°	
Index ranges	-23≤h≤24, -9≤k≤10, -20≤l≤26	
Reflections collected	11699	
Independent reflections	3720 [R(int) = 0.0252]	
Completeness to θ = 76.126°	99.3%	
Absorption correction	Multi-scan	
Refinement method	Full-matrix least squares on F^2	
Goodness-of-fit on F^2	0.9687	
Final R indices [I>2σ(I)]	R^1 = 0.0349, wR^2 = 0.0951	
R indices (all data)	R^1 = 0.0403, wR^2 = 0.1010	

Crystal data for (3*S**,4*S**)-1-benzyl-3-(4-fluorophenyl)-1-azaspiro[3.5]nonane-2,5-dione, **2e**

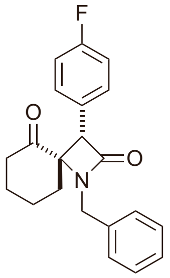

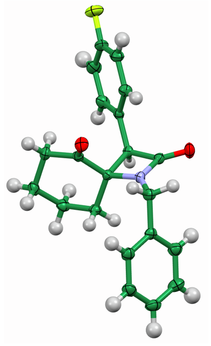

Identification code	CCDC 2033324	
Empirical formula	$C_{21}\ H_{20}\ F_1\ N_1\ O_2$	
Formula weight	337.39	
Temperature	150K	
Wavelength	1.54180 Å	
Crystal system	Orthorhombic	
Space group	P b c a	
Unit cell dimensions	a = 17.1683(3) Å	α = 90°
	b = 9.93350(10) Å	β = 90°
	c = 19.9820(3) Å	γ = 90°
Volume	3407.76(9) $Å^3$	
Z	8	
Density (calculated)	1.32 Mg m^{-3}	
Absorption coefficient	0.747 mm^{-1}	
F(000)	1424.000	
Crystal size	0.12 × 0.17 × 0.23 mm	
Theta range for data collection	5.122 ° to 76.1091°	
Index ranges	-21≤h≤19, -7≤k≤12, -22≤l≤24	
Reflections collected	13187	
Independent reflections	3529 [R(int) = 0.0270]	
Completeness to θ = 76.091°	99.0%	
Absorption correction	Multi-scan	
Refinement method	Full-matrix least squares on F^2	
Goodness-of-fit on F^2	0.9649	
Final R indices [I>2σ(I)]	R^1 = 0.0345, wR^2 = 0.0912	
R indices (all data)	R^1 = 0.0402, wR^2 = 0.0969	

References

[1] Pangborn, A.B., Giardello, M.A., Grubbs, R.H., Rosen, R.K. and Timmers, F.J. (1996) Safe and Convenient Procedure for Solvent Purification. Organometallics *15*, 1518–1520.

[2] Teegardin, K.A. and Weaver, J.D. (2018) Preparation of *Fac*-Tris(2-Phenylpyridinato) Iridium(III). Org. Synth. *95*, 29–45.

[3] Arnould, J.C., Cossy, J. and Pete, J. P. (1980) Reactivite Photochmique Des α-aminoenones: Reactions De Cyclisation Et Nouveau Type De Reaction Dans Les α-sulfonamido-cyclohexenones. Tetrahedron *36*, 1585–1592.

[4] Parsons, A.F. and Williams, D.A. (2000) Radical Cyclisation Reactions Leading to Polycyclics Related to the Amaryllidaceae and Erythrina Alkaloids. Tetrahedron *56*, 7217–7228.

[5] Jursic, B. (1989) Surfactant assisted permanganate oxidation of aromatic compounds. Can. J. Chem. *67*, 1381–1383.

[6] Caner, J. and Villarrasa, J. (2010) ^{15}N Double-Labeled Guanosine from Inosine through Ring-Opening-Closing and One-Pot Pd-Catalyzed C–O and C–N Cross-Coupling Reactions. J. Org. Chem. *75*, 4880–4883.

[7] (a) Martins, A. and Lautens, M. (2008) A Simple, Cost-Effective Method for the Regioselective Deuteration of Anilines. Org. Lett. *10*, 4351–4353. (b) Xiang, Y., Caron, P.-Y., Lillie, B.M. and Vaidyanathan (2008) R. Sulfur Contamination Due to Quenching of Halogenation Reactions with Sodium Thiosulfate: Resolution of Process Problems via Improved Quench Protocols. Org. Process Res. Dev. *12*, 116–119.

[8] Zhao, D., Jiang, Y. and Ma, D. (2014) Copper-catalyzed coupling of aryl iodides and *tert*-butyl β-keto esters: efficient access to α-aryl ketones and α-arylacetic acid *tert*-butyl esters. Tetrahedron *70*, 3327–3332.

[9] Zhao, Y. and Truhlar, D.G. (2008) The M06 suite of density functionals for main group thermochemistry, thermochemical kinetics, noncovalent interactions, excited states, and transition elements: two new functionals and systematic testing of four M06-class functionals and 12 other functionals. Theor. Chem. Acc. *120*, 215−241.

[10] (a) Hehre, W.J., Ditchfield, R. and Pople, J.A. (1972) Self-consistent molecular orbital methods. XII. Further extensions of gaussian-type basis sets for use in molecular orbital studies of organic molecules. J. Chem. Phys. *56*, 2257–2261. (b) Hariharan, P.C. and Pople, J.A. (1973) The influence of polarization functions on molecular orbital hydrogenation energies. Theoret. chim. Acta *28*, 213–222. (c) Krishnan, R., Binkley, J. S., Seeger, R. and Pople, J.A. (1980) Self-consistent molecular orbital methods. XX. A basis set for correlated wave functions. J. Chem. Phys. *72*, 650–654. (d) McLean, A.D. and Chandler, G.S. (1980) Contracted Gaussian basis sets for molecular calculations. I. Second row atoms, Z=11–18. J. Chem. Phys. *72*, 5639–5648. (e) Francl, M.M., Pietro, W.J., Hehre, W.J., Binkley, J.S. Gordon, M.S., DeFrees, D.J. and Pople, J.A. (1982) Self-consistent molecular orbital methods. XXIII. A polarization-type basis set for second-row elements. J. Chem. Phys. *77*, 3654–3665. (f) Rassolov, V.A., Ratner, M.A., Pople, J.A., Redfern, P.C. and Curtiss, L.A. (2001) 6-31G* basis set for third-row atoms. J. Comp. Chem. *22*, 976–984.

[11] (a) Weigend, F. and Ahlrichs, R. (2005) Balanced basis sets of split valence, triple zeta valence and quadruple zeta valence quality for H to Rn: Design and assessment of accuracy. Phys. Chem. Chem. Phys. *7*, 3297−3305. (b) Weigend, F. (2006) Accurate coulomb-fitting basis sets for H to Rn. Phys. Chem. Chem. Phys. *8*, 1057−1065.

[12] (a) Jones, B.A., Solon, P., Popescu, M.V., Du, J.-Y., Paton, R. and Smith, M.D. (2023) Catalitic Enantioselective 6π Photocyclization of Acrylanilides. J. Am. Chem. Soc. *145*, 171-178. (b) Munster, N., Parker, N.A., van Dijk, L., Paton, R.S. and Smith, M.D. (2017) Visible Light Photocatalysis of 6π Heterocyclization. Angew. Chem. Int. Ed. *56*, 9468-9472. (c) Popescu, M.V., Mekereeya, A., Alegre-Requena, J. V., Paton, R.S. and Smith, M.D. (2020) Visible-Light-Mediated Heterocycle Functionalization via Geometrically Interrupted [2+2] Cycloaddition. Angew. Chem. Int. Ed. *59*, 23020-23024; (d) St. John, P.C., Guan, Y., Kim, S. and Paton, R.S. (2020) Prediction of Organic homolytic bond dissociation enthalpies at near chemical accuracy with sub-second computational cost. Nat. Commun. *11*, 2328; (e) S.V., S. S., St. John, P.C. and Paton, R.S. (2021) A quantitative metric for organic radical stability and persistence using thermodynamic and kinetic features. Chem. Sci. *12*, 13158-13166.

[13] (a) Cancès, E., Mennucci, B. and Tomasi, J. (1997) A new integral equation formalism for the polarizable continuum model: Theoretical background and applications to isotropic and anisotropic dielectrics. J. Chem. Phys. *107*, 3032–3041. (b) Mennucci, B., Cancès, E. and Tomasi, J. (1997) Evaluation of solvent effects in isotropic and anisotropic dielectrics and in ionic solutions with a unified integral equation method: theoretical bases, computational implementation, and numerical applications. J. Phys. Chem. B. *101*, 10506–10517. (c) Mennucci, B. and Tomasi, J. (1997) Continuum solvation models: A new approach to the problem of solute's charge

distribution and cavity boundaries. J. Chem. Phys. *106*, 5151–5158. (d) Tomasi, J., Mennucci, T. B. and Cancès, E. (1999) The IEF version of the PCM solvation method: an overview of a new method addressed to study molecular solutes at the QM ab initio level. J. Mol. Struct. THEOCHEM *464*, 211–226. (e) Scalmani, G. and Frisch, M. J. (2010) Continuous surface charge polarizable continuum models of solvation. I. General formalism. J. Chem. Phys. *132*, 114110.

[14] Luchini, G., Alegre-Requena, J.V., Funes-Ardoiz, I. and Paton, R.S. (2020) GoodVibes: Automated Thermochemistry for Heterogeneous Computational Chemistry Data. F1000Research *9*, 291.

[15] Gaussian 16, Revision B.01 (2016) Frisch, M.J., Trucks, G.W., Schlegel, H.B., Scuseria, G.E., Robb, M.A., Cheeseman, J.R., Scalmani, G., Barone, V., Petersson, G.A., Nakatsuji, H., Li, X., Caricato, M., Marenich, J., Bloino, A., Janesko, B.G., Gomperts, R., Mennucci, B., Hratchian, H.P., Ortiz, J.V., Izmaylov, A.F., Sonnenberg, J.L., Williams-Young, D.; Ding, F., Lipparini, F., Egidi, F., Goings, J., Peng, B., Petrone, A., Henderson, T., Ranasinghe, D., Zakrzewski, V.G., Gao, J., Rega, N., Zheng, G., Liang, W., Hada, M., Ehara, M., Toyota, K., Fukuda, R., Hasegawa, J., Ishida, M., Nakajima, T., Honda, Y., Kitao, O., Nakai, H., Vreven, T., Throssell, K., Montgomery, Jr. J.A., Peralta, J.E., Ogliaro, F., Bearpark, M., Heyd, J.J., Brothers, E., Kudin, K.N., Staroverov, V.N., Keith, T., Kobayashi, R., Normand, J., Raghavachari, K., Rendell, A., Burant, J.C., Iyengar, S.S., Tomasi, J., Cossi, M., Millam, J.M., Klene, M., Adamo, C., Cammi, R., Ochterski, J.W., Martin, R.L., Morokuma, K., Farkas, O., Foresman, J.B., Fox, D.J., Gaussian, Inc., Wallingford CT.

[16] Fukui, K. (1981) The path of chemical reactions - the IRC approach. Acc. Chem. Res. *14*, 363–368.

[17] Neese, F. (2012) The ORCA program system. Wiley Interdiscip. Rev. Comput. Mol. Sci., *2*, 73–78.

[18] Alegre-Requena, J.V., S.V., S.S., Alturaifi, T., Pérez-Soto, R. and Paton, R. (2020) AQME: Automated quantum mechanical environments for researchers and educators. WIREs Comp. Mol. Sci. *13*, e1663.

[19] The PyMOL Molecular Graphics System v. 2.0.7, Schrödinger, LLC.

[20] https://gist.github.com/bobbypaton (accessed 13 April 2018).

[21] (a) Seeger, R. and Pople, J.A. (1997) Self-consistent molecular orbital methods. 28. Constraints and stability in Hartree-Fock theory. J. Chem. Phys. *66*, 3045–3050. (b) Bauernschmitt, R. and Ahlrichs, R. (1996) Stability analysis for solutions of the closed shell Kohn-Sham equation. J. Chem. Phys. *104*, 9047–9052. (c) Robb, M.A. and McDouall, J.J.W. (1991) Do you Have SCF Stability and Convergence Problems?. In Computational Advances in Organic Chemistry: Molecular Structure and Reactivity, Ögretir, C. ed. and Csizmadia, I.G. ed. (Kluwer Academic, The Netherlands), pp. 167–185.

[22] Snyder, J.D., Hamill, L.-A., Faleumu, K. E. and Ess, D.H. (2019) MECPro v. 1.0.5: Minimum Energy Crossing Program, Brigham Young University.

[23] Yanai, T., Tew, D. and Handy, N. (2004) A new hybrid exchange-correlation functional using the Coulomb-attenuating method (CAM-B3LYP). Chem. Phys. Lett. *393*, 51-57.

[24] Tawada, Y., Tsuneda, T., Yanagisawa, S., Yanai, T. and Hirao, K. A (2004) Long-range-corrected time-dependent density functional theory. J. Chem. Phys. 120, 8425-8433.

[25] Chai, J.-D. and Head-Gordon, M. (2008) Systematic optimization of long-range corrected hybrid density functionals. J. Chem. Phys. 128, 084106

[26] Kwan, E.E. and Liu, R.Y. (2015) Enhancing NMR Prediction for Organic Compounds Using Molecular Dynamics. J. Chem. Theory Comput. *11,* 5083–5089.

[27] Essafi, S. and Harvey, J.N. (2018) Rates of molecular vibrational energy transfer in organic solutions. J. Phys. Chem. A*122*, 3535–3540.

[28] Kitagawa, Y., Saito, T. and Yamaguchi, K. (2008) Approximate Spin Projection for Broken-Symmetry Method and Its Application. In Symmetry (Group Theory) and Mathematical Treatment in Chemistry, Akitsu, T. ed. (IntechOpen), pp. 121–139.

[29] Popescu, M.V. and Paton, R.S. (2024) Dynamic vertical triplet energies: Understanding and predicting triplet energy transfer. Chem *10*, 3428–3443.

[30] Teynor, M.S., Wohlgemuth, N., Carlson, L., Huang, J., Pugh, S.L., Grant, B.O., Hamilton, R.S., Carlsen, R. and Ess, D.H. (2021) Milo, revision 1.0.3, Brigham Young Uni- versity, Provo UT (https://github.com/DanielEss-lab/milo).

[31] Easton, R.E., Giesen, D.J., Welch, A., Cramer, C.J. and Truhlar, D.G. (1996) The MIDI! basis set for quantum mechanical calculations of molecular geometries and partial charges. Theo. chim. Acta *93*, 281–301.

[32] Grimme, S. (2012) Supramolecular Binding Thermodynamics by Dispersion-corrected Density Functional Theory. Chem. Eur. J. *18*, 9955–9964.

[33] Sure, R. and Grimme, S. (2015) Comprehensive benchmark of association (free) energies of realistic host–guest complexes. J. Chem. Theory Comput. *11*, 3785–3801.

[34] (a) Bailey, W.F. and Monahan, A.S. (1978) Statistical effects and the evaluation of entropy differences in equilibrium processes. Symmetry corrections and entropy of mixing. J. Chem. Ed *55*, 489. (b) Plata, R.E. and Singleton, D.A. (2015) A Case study of the mechanism of alcohol-mediated Morita Baylis−Hillman reactions. The importance of experimental observations. J. Am. Chem. Soc. *137*, 3811–3826.
