# Supplementary material for: Photocatalysis as a mechanistic probe for the Staudinger β-lactam synthesis
Source: Chem Catal. Author manuscript; Available in PMC 2026 May 15. (PMC13175146; doi:10.1016/j.checat.2025.101493)
Supplement: NMR spectra [file NIHMS2173764-supplement-NMR_spectra.pdf]

NMR Data

Table of Contents

Compound 8 3
Compound 10a 4
Compound 10l 5
Compound 10m 6
Compound 10o 7
Compound 10p 8
Compound 1a 9
Compound 1a-d_2 10
Compound 1b 11
Compound 1c 12
Compound 1d 13
Compound 1e 14
Compound 1f 16
Compound 1g 17
Compound 1h 19
Compound 1i 21
Compound 1j 22
Compound 1l 23
Compound 1m 24
Compound 1n 25
Compound 1o 26
Compound 1p 27
Compound 1q 28
Compound 1r 29
Compound 1t 30
Compound 1u 31
Compound 2a 32
Compound 2b 33
Compound 2c 34
Compound 2d 35
Compound 2e 36
Compound 2f 38
Compound 2g 39

Compound 2h 41
Compound 2i 43
Compound 2j 44
Compound 2l 45
Compound 2m 46
Compound 2n 47
Compound 2o 48
Compound 2p 49
Compound 2q 50
Compound 2r 51
Compound 2s 52
Compound 2t 53
Compound 2u 54
Compound 2a-d_2 55
Compound 7a 56
Compound 11 57
Compound 12 59
Compound 13 61
Compound 14 63
Compound 15 65
Compound 16 67
Compound 1i-d_7 69

Compound 8

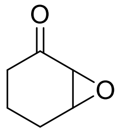
O
O

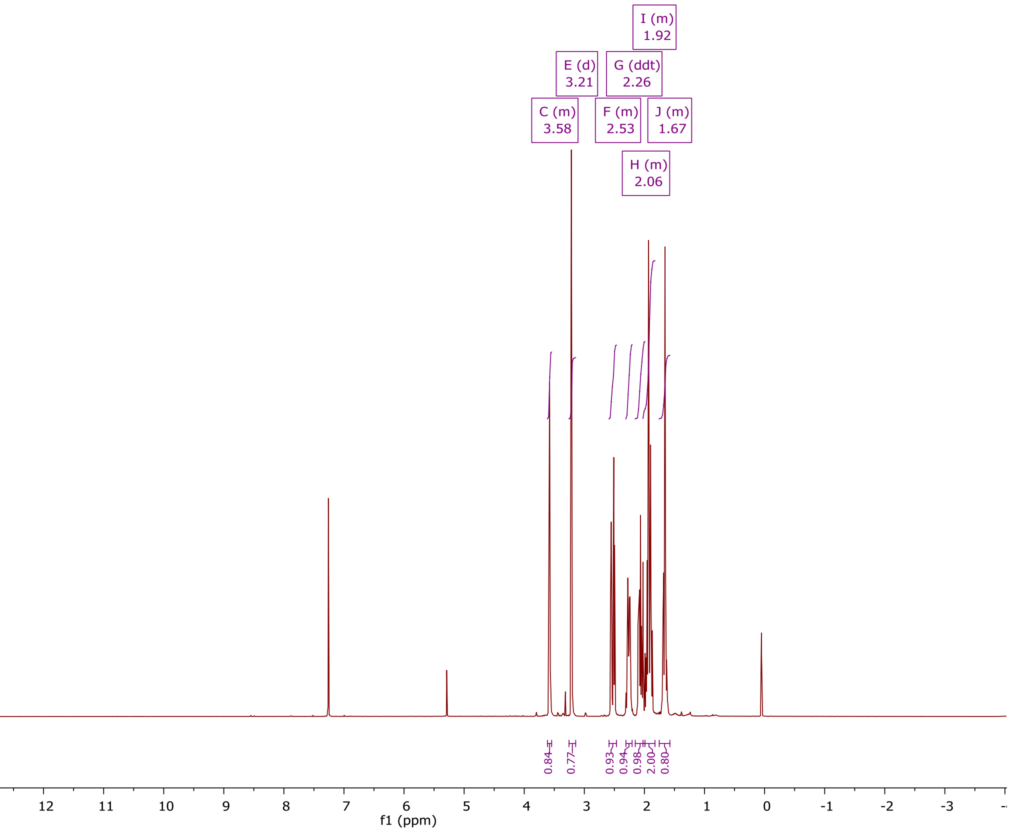
I (m)
1.92
E (d)
3.21
G (ddt)
2.26
C (m)
3.58
F (m)
2.53
J (m)
1.67
H (m)
2.06
0.84
0.77
0.93
0.94
0.98
2.00
0.80
15 14 13 12 11 10 9 8 7 6 5 4 3 2 1 0 -1 -2 -3
f1 (ppm)

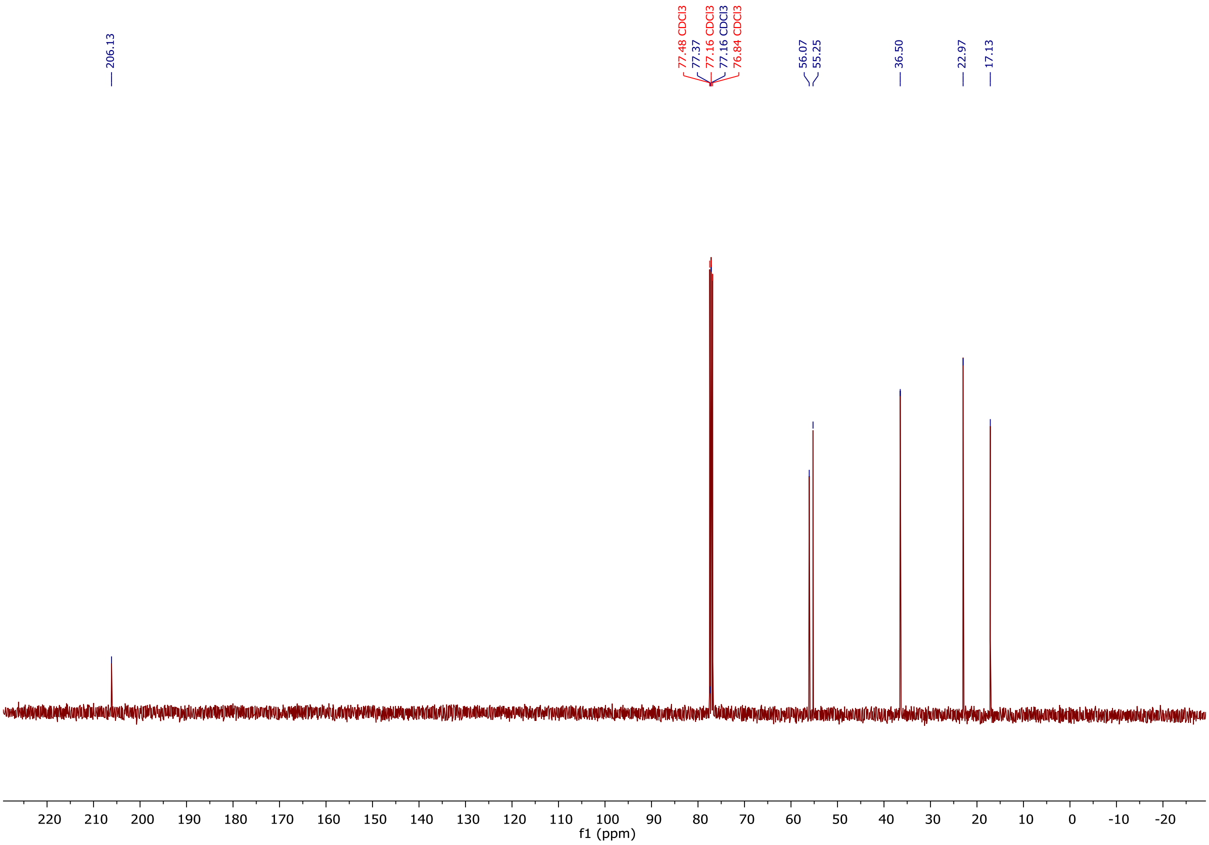
206.13
77.48 CDCl3
77.37
77.16 CDCl3
77.16 CDCl3
76.84 CDCl3
56.07
55.25
36.50
22.97
17.13
220 210 200 190 180 170 160 150 140 130 120 110 100 90 80 70 60 50 40 30 20 10 0 -10 -20
f1 (ppm)

Compound 10a

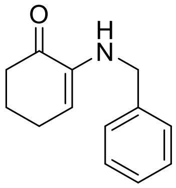

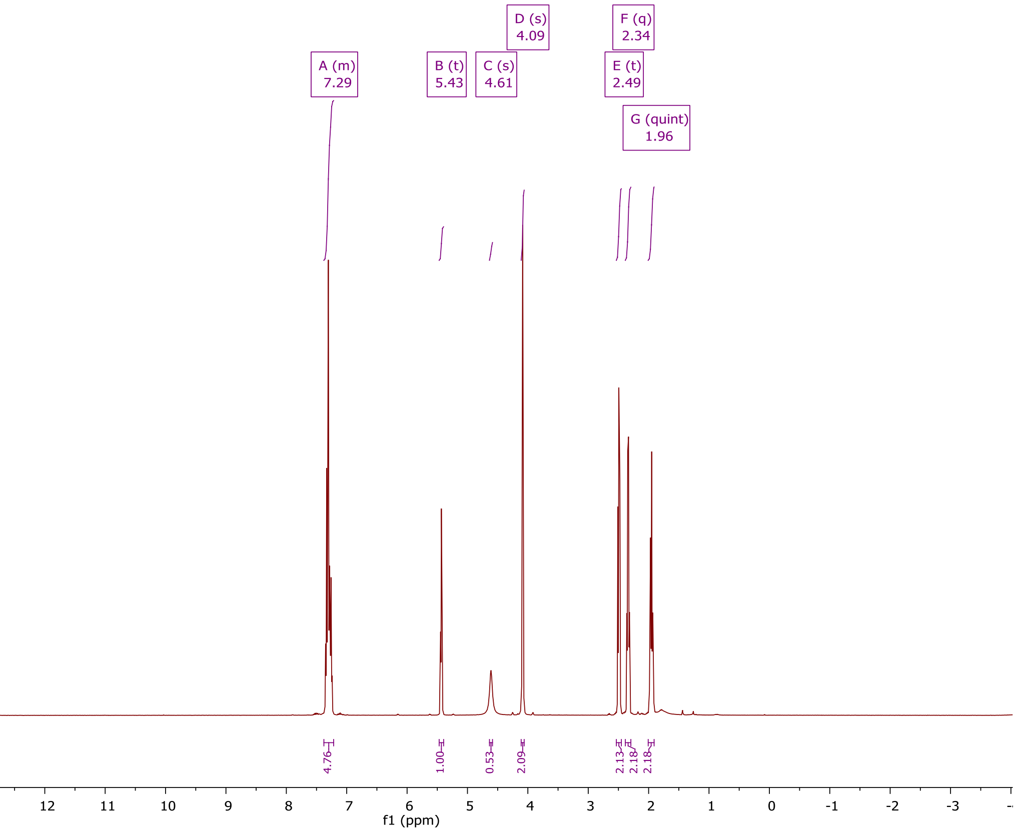

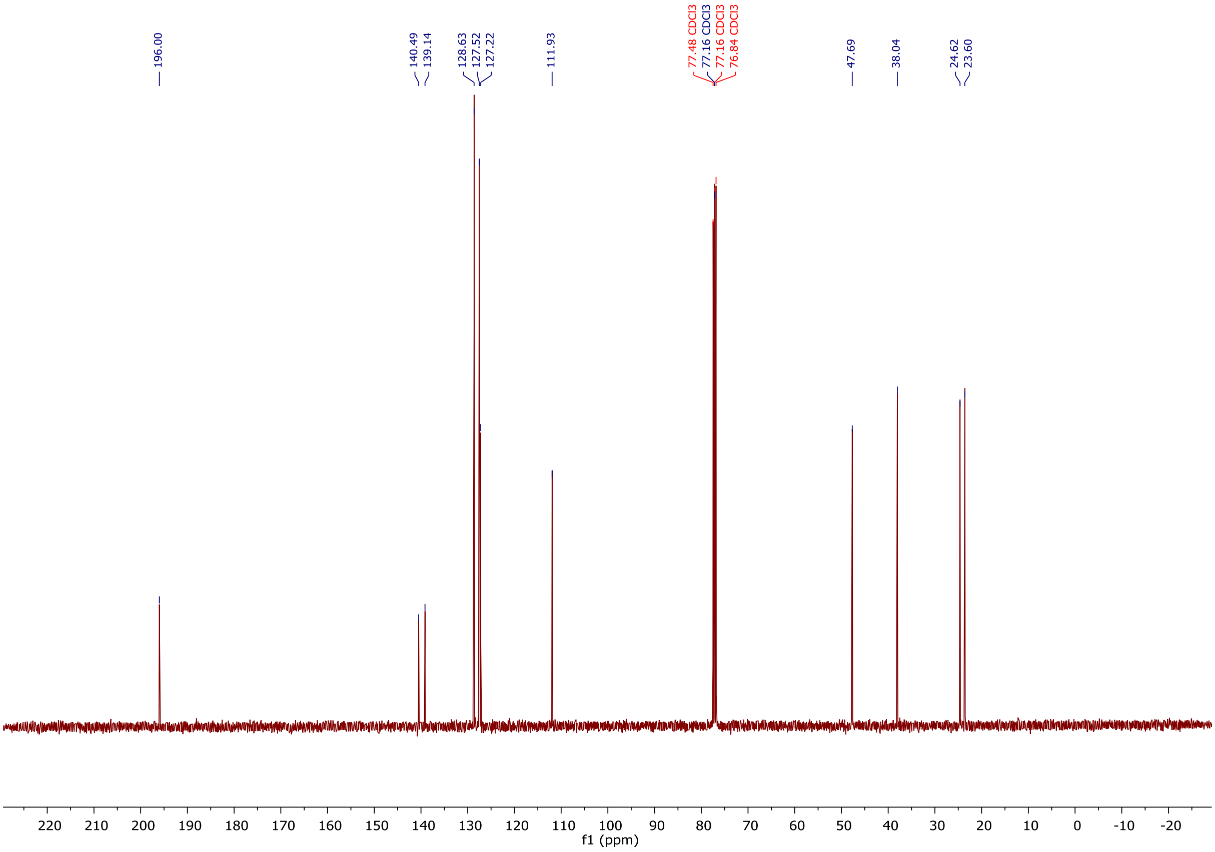

Compound 10l

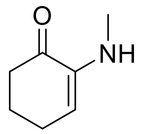

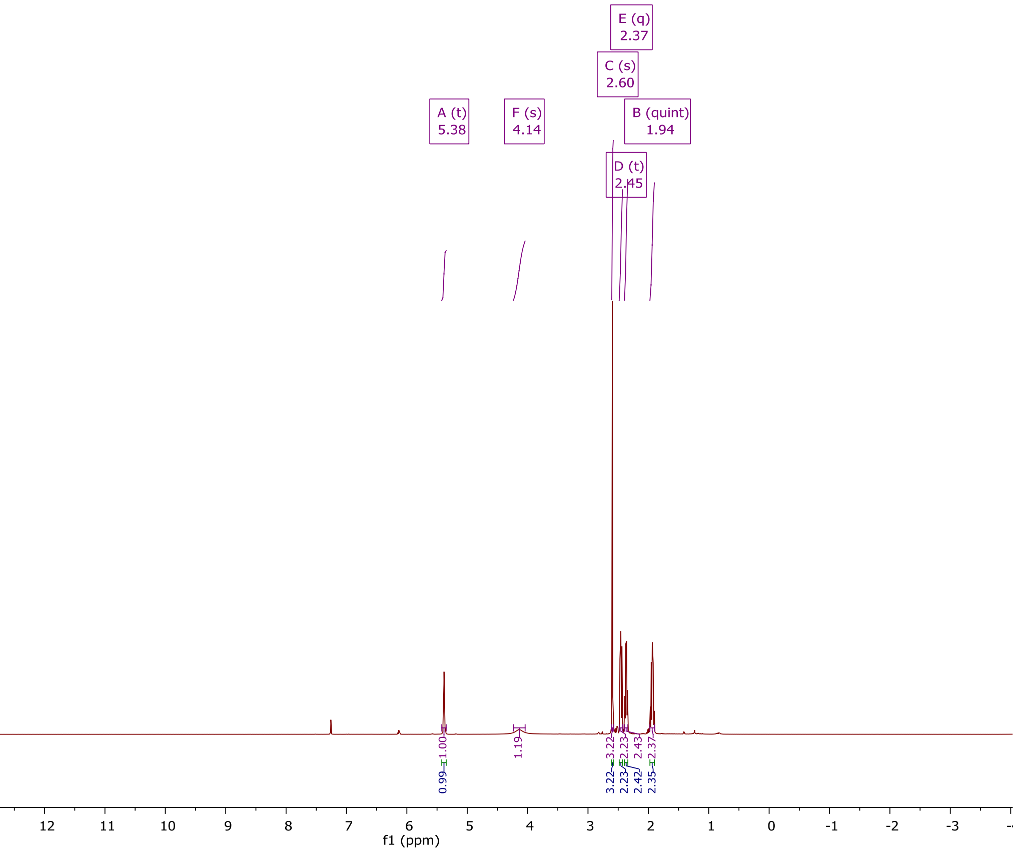

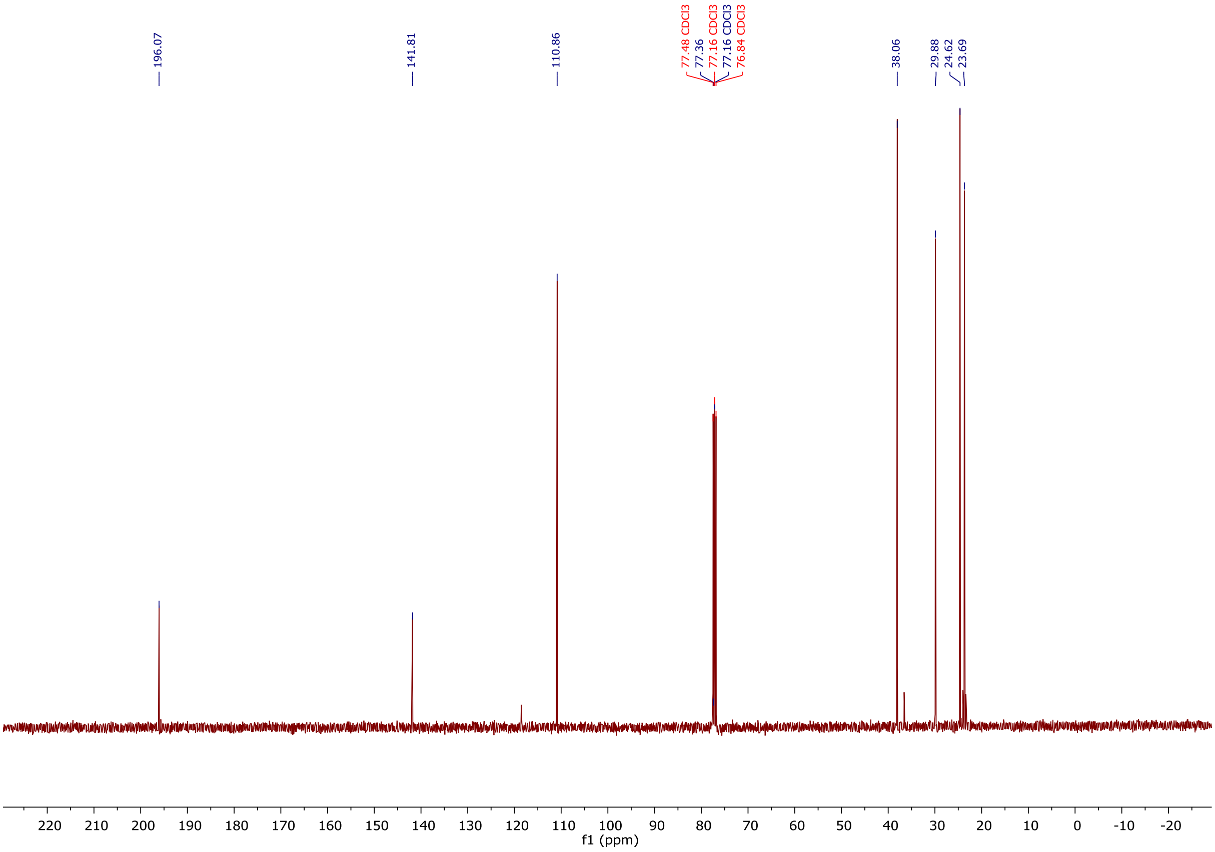

Compound 10m

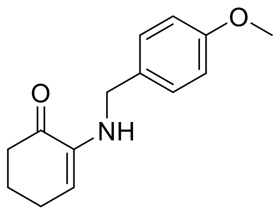

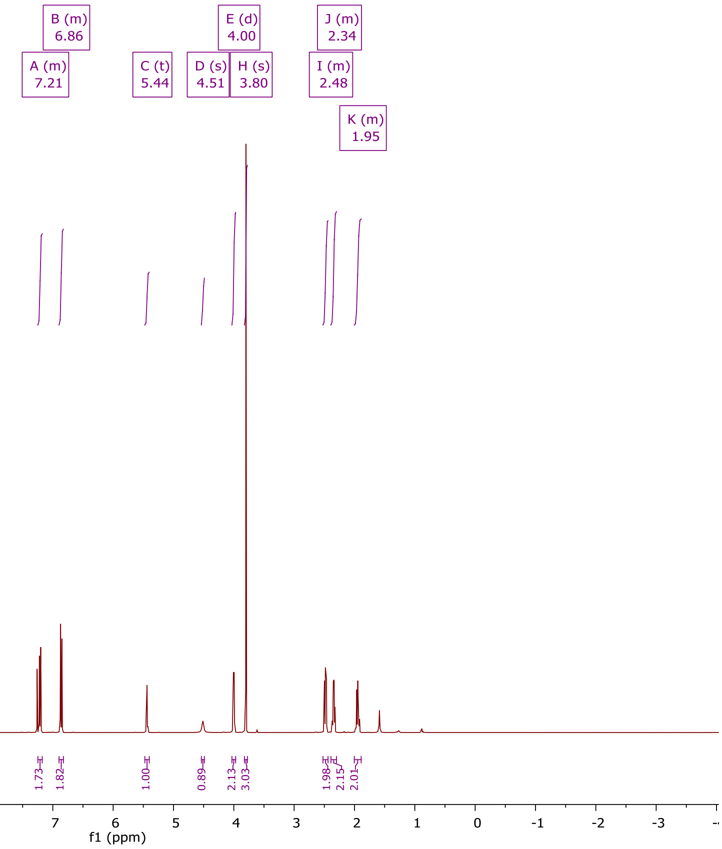

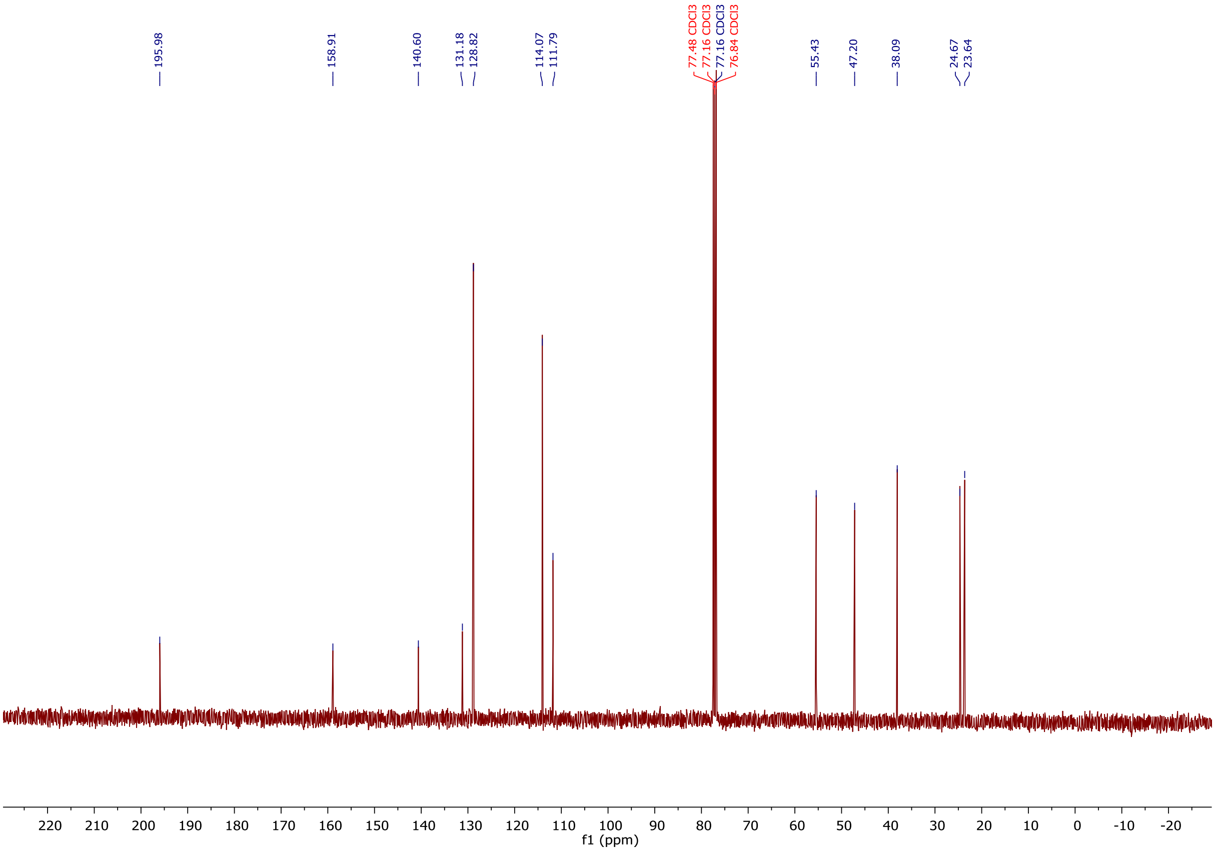

Compound 10o

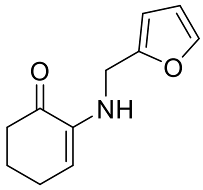

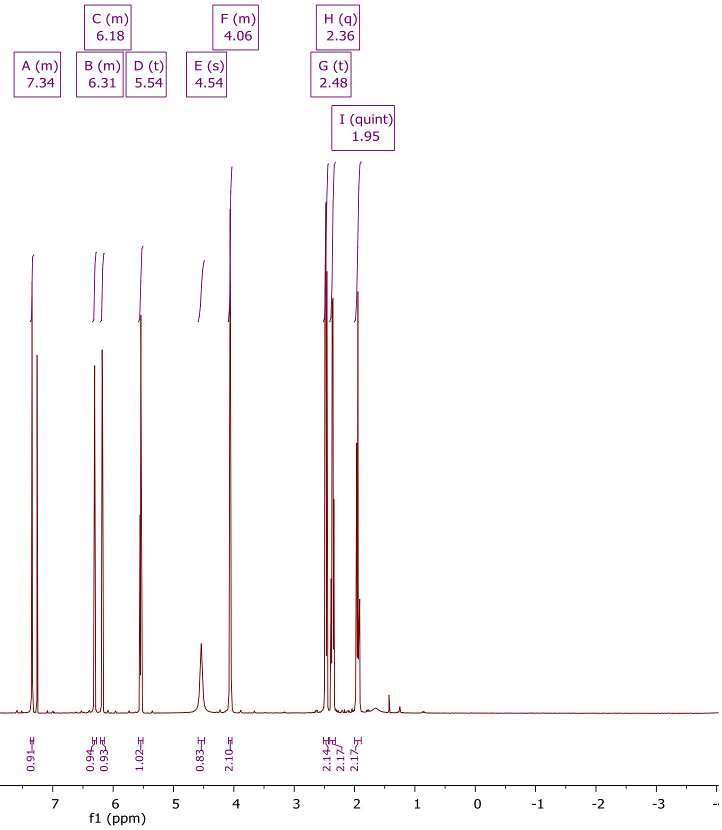

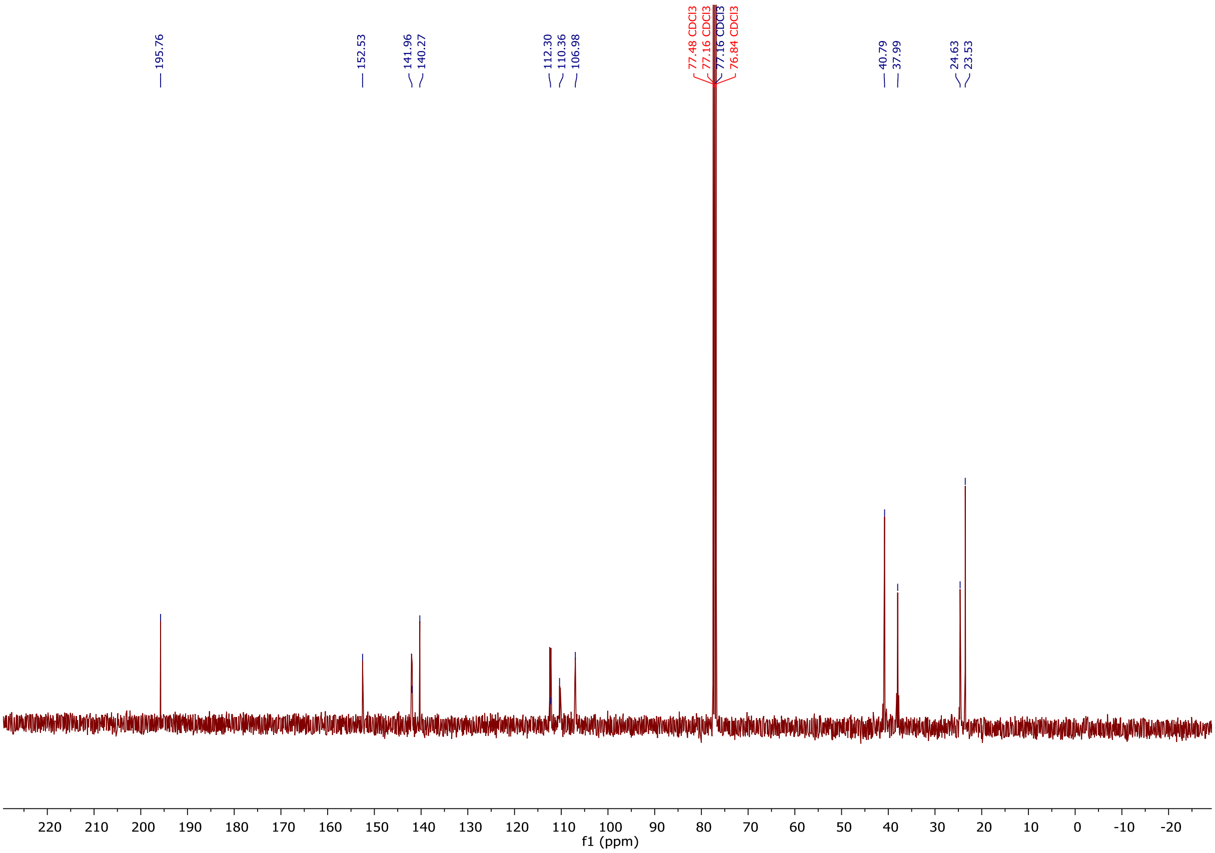

Compound 10p

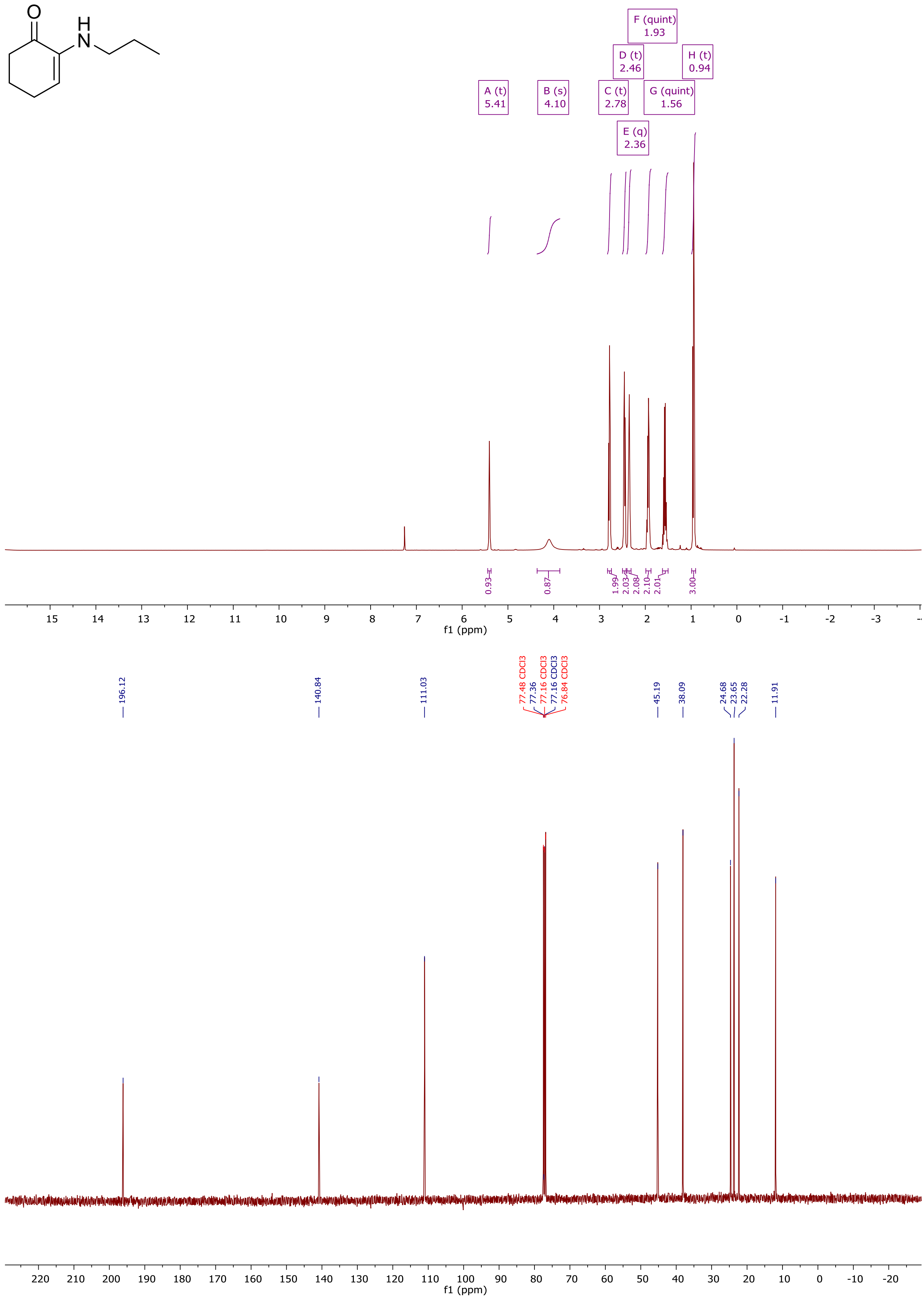

Compound 1a

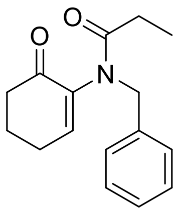

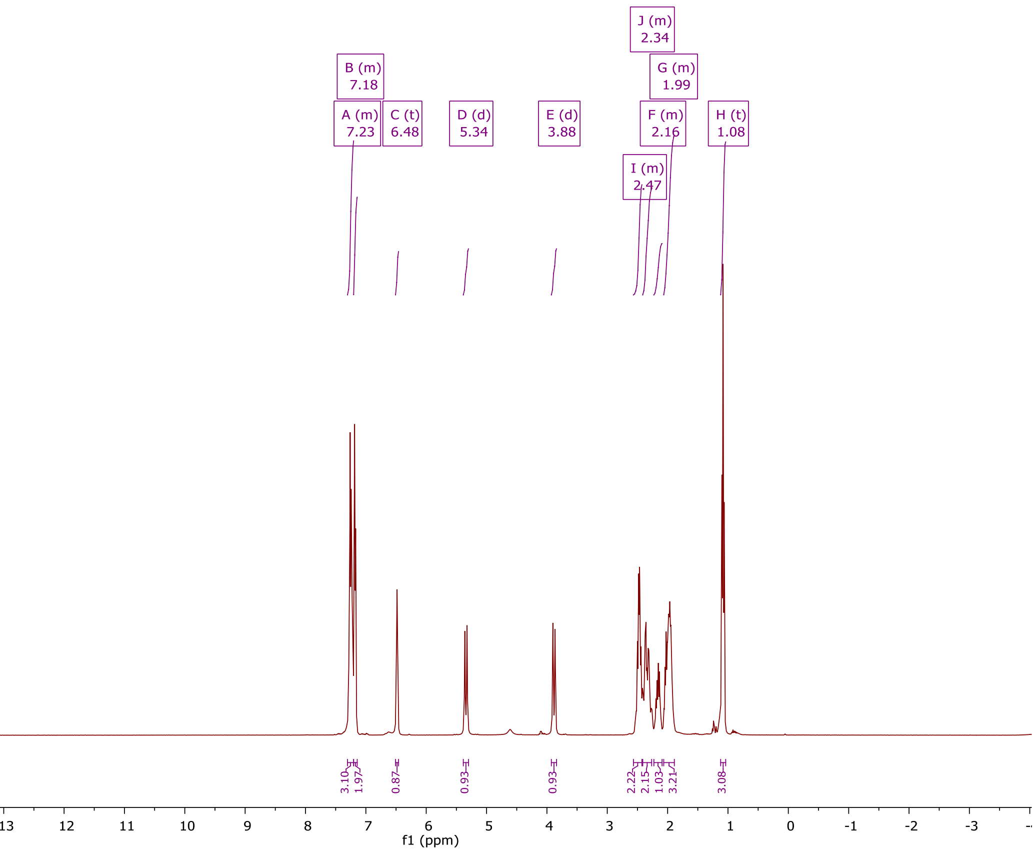
J (m)
2.34
B (m)
7.18
G (m)
1.99
A (m)
7.23
C (t)
6.48
D (d)
5.34
E (d)
3.88
F (m)
2.16
H (t)
1.08
I (m)
2.47
3.10
1.97
0.87
0.93
0.93
2.22
2.15
1.03
3.21
3.08
f1 (ppm)

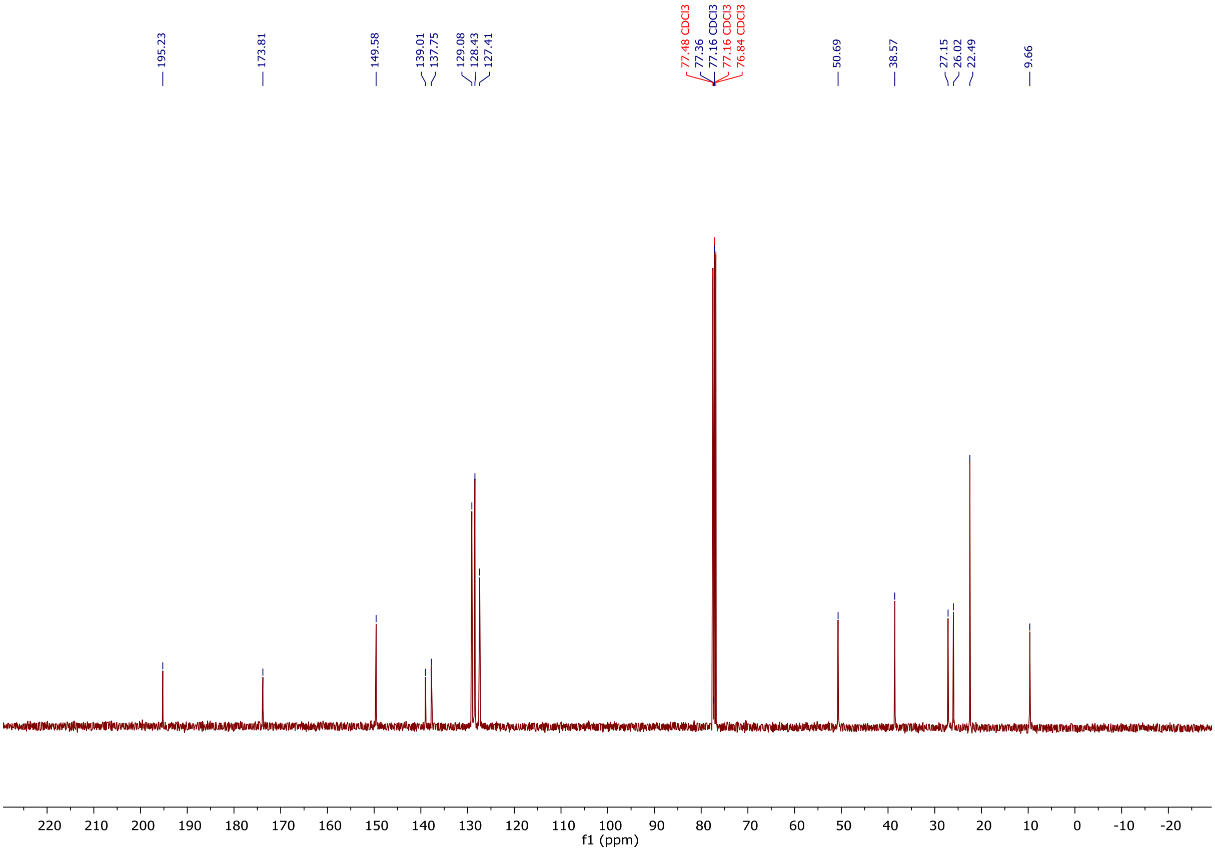
195.23
173.81
149.58
139.01
137.75
129.08
128.43
127.41
77.48 CDCl3
77.36
77.16 CDCl3
77.16 CDCl3
76.84 CDCl3
50.69
38.57
27.15
26.02
22.49
9.66
f1 (ppm)

Compound 1a-d_2

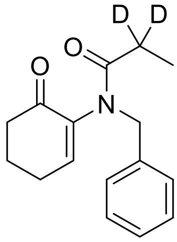

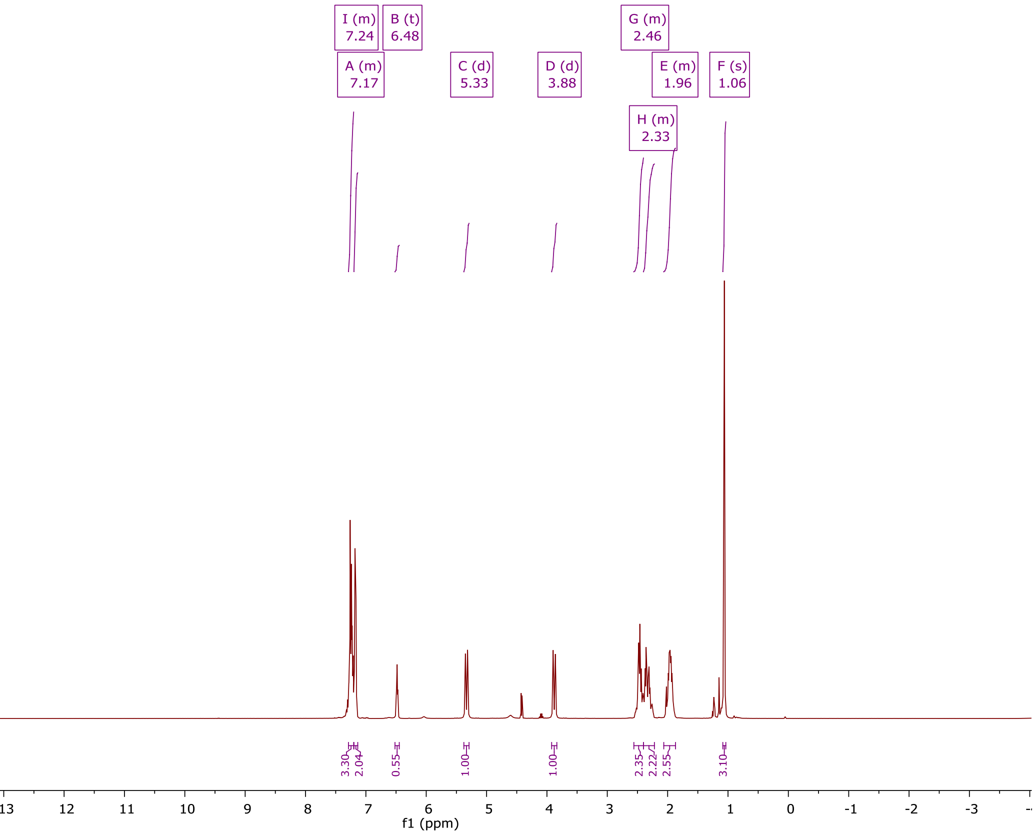

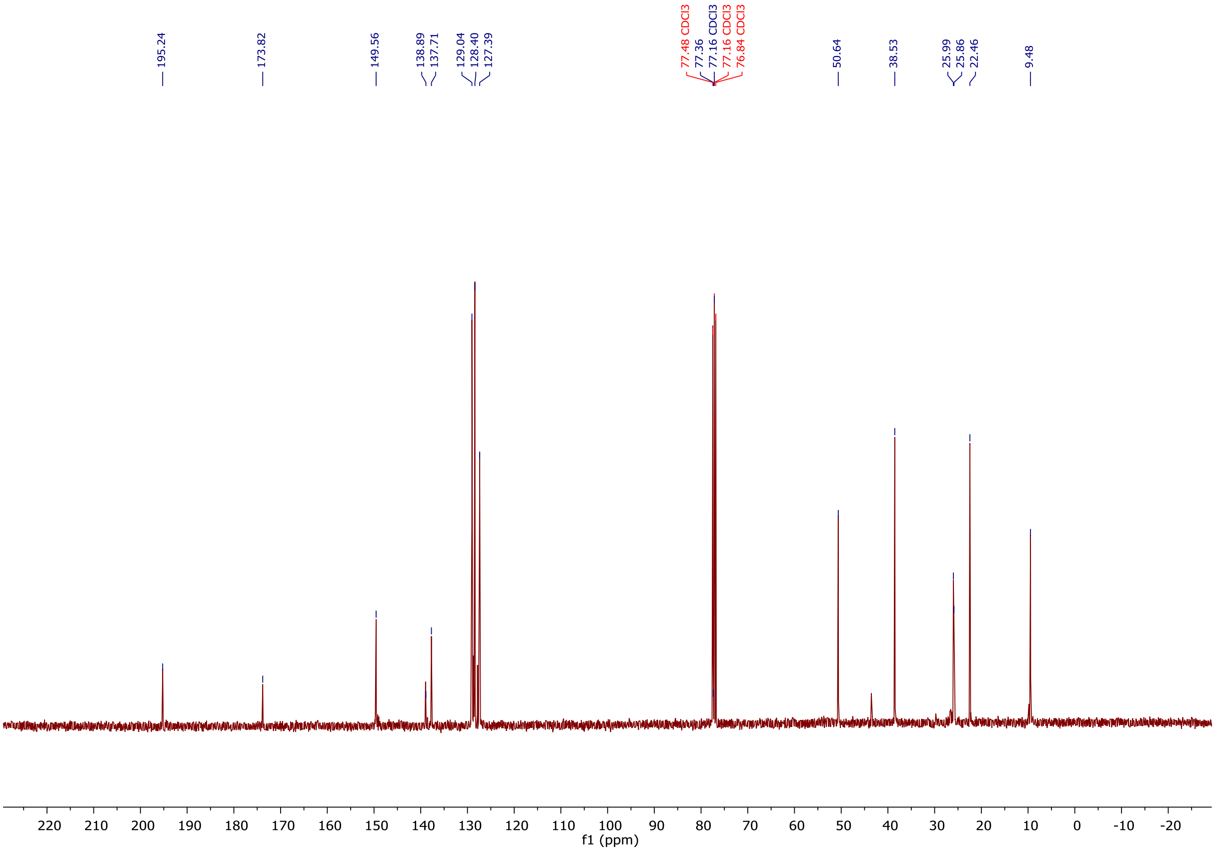

Compound 1b

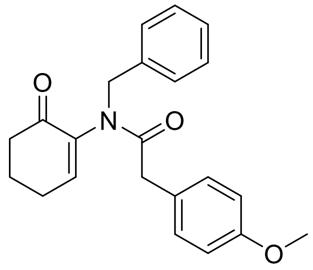

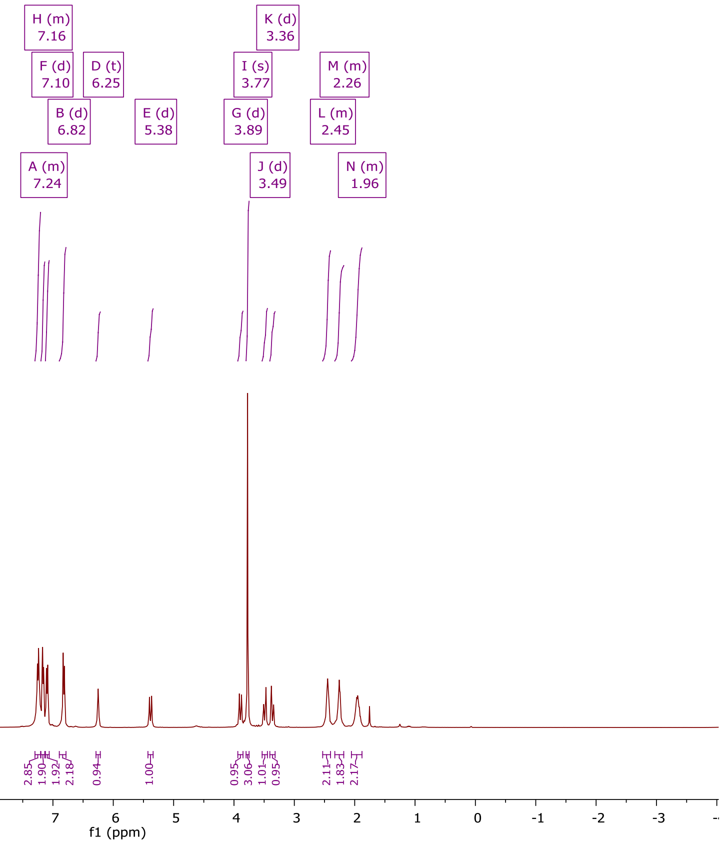

H (m) 7.16
K (d) 3.36
F (d) 7.10
D (t) 6.25
I (s) 3.77
M (m) 2.26
B (d) 6.82
E (d) 5.38
G (d) 3.89
L (m) 2.45
A (m) 7.24
J (d) 3.49
N (m) 1.96
2.85
1.90
1.92
2.18
0.94
1.00
0.95
3.06
1.01
0.95
2.11
1.83
2.17
f1 (ppm)

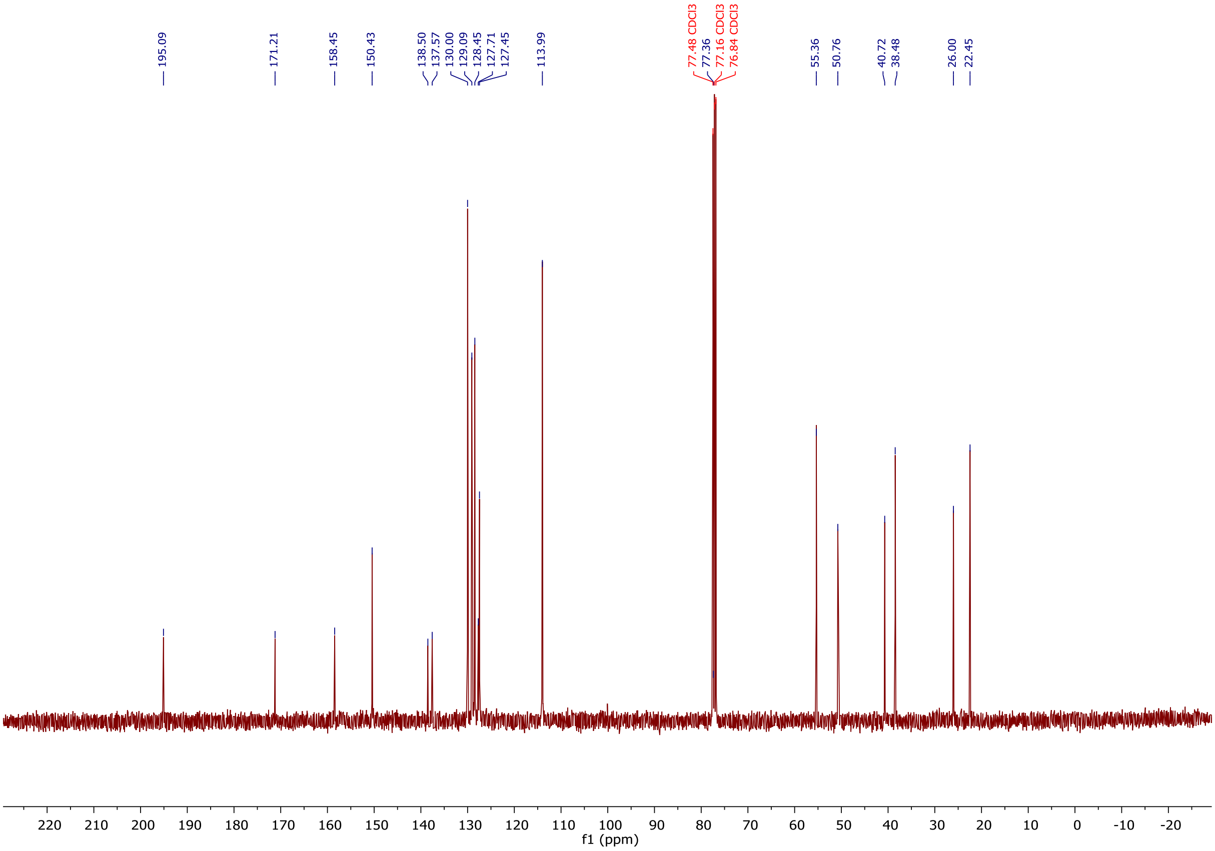

195.09
171.21
158.45
150.43
138.50
137.57
130.00
129.09
128.45
127.71
127.45
113.99
77.48 CDCl3
77.36
77.16 CDCl3
76.84 CDCl3
55.36
50.76
40.72
38.48
26.00
22.45
f1 (ppm)

Compound 1c

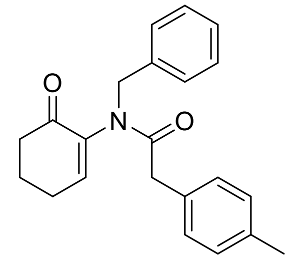

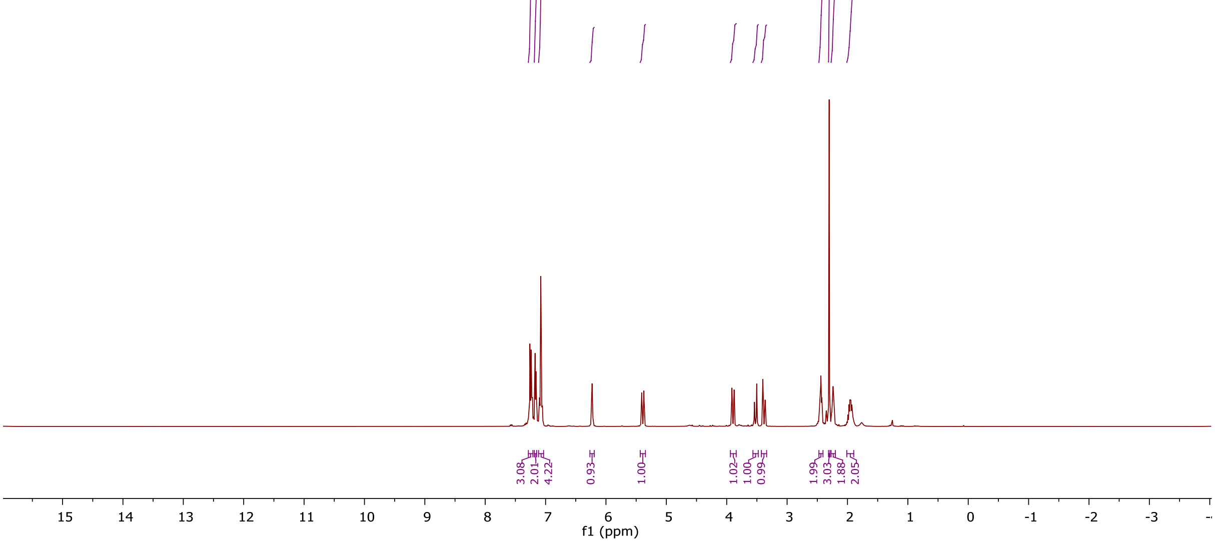
K (m)
1.95
B (m)
7.17
F (d)
3.52
I (s)
2.30
A (m)
7.24
C (t)
6.23
D (d)
5.39
E (d)
3.90
H (m)
2.44
L (m)
7.08
G (d)
3.38
J (m)
2.24
3.08
2.01
4.22
0.93
1.00
1.02
1.00
0.99
1.99
3.03
1.88
2.05
f1 (ppm)

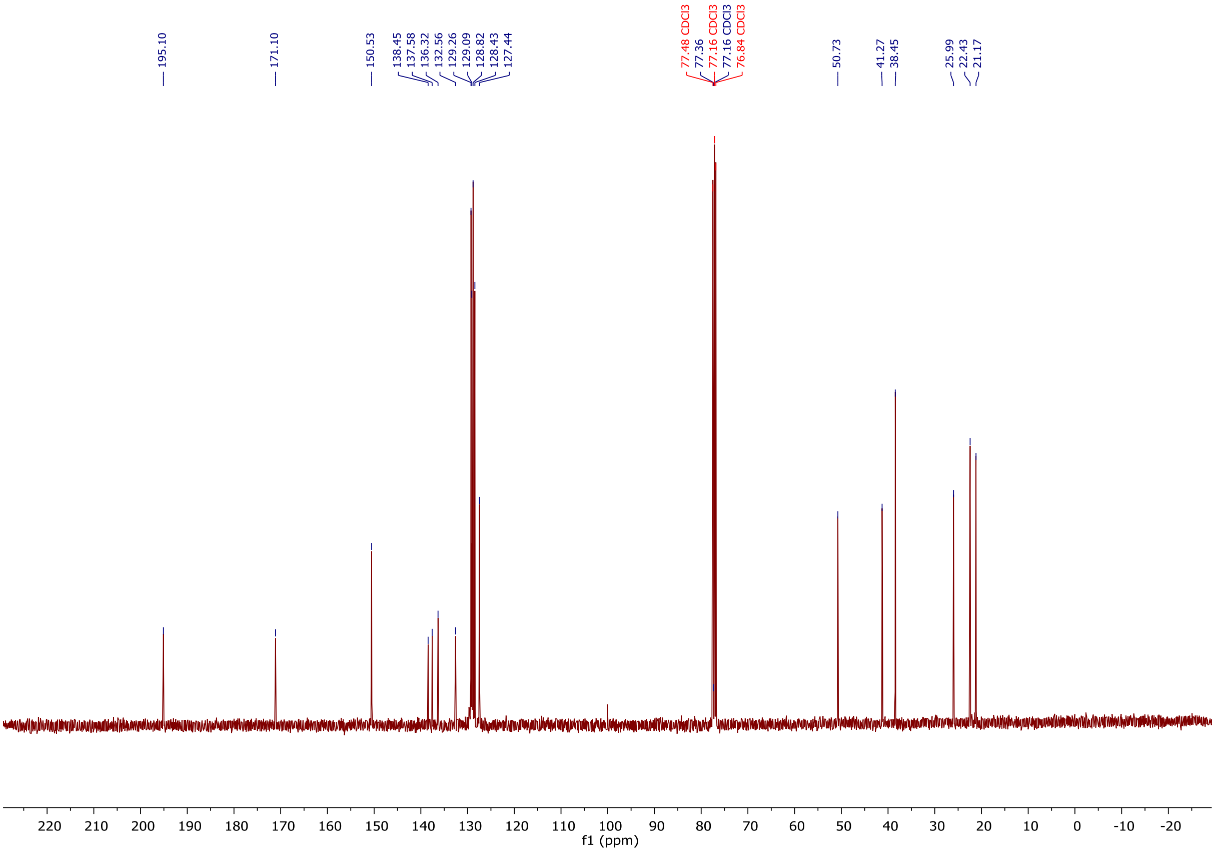
195.10
171.10
150.53
138.45
137.58
136.32
132.56
129.26
129.09
128.82
128.43
127.44
77.48 CDCl3
77.36
77.16 CDCl3
77.16 CDCl3
76.84 CDCl3
50.73
41.27
38.45
25.99
22.43
21.17
f1 (ppm)

Compound 1d

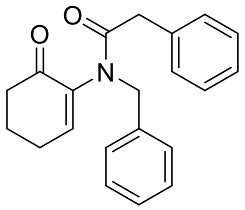

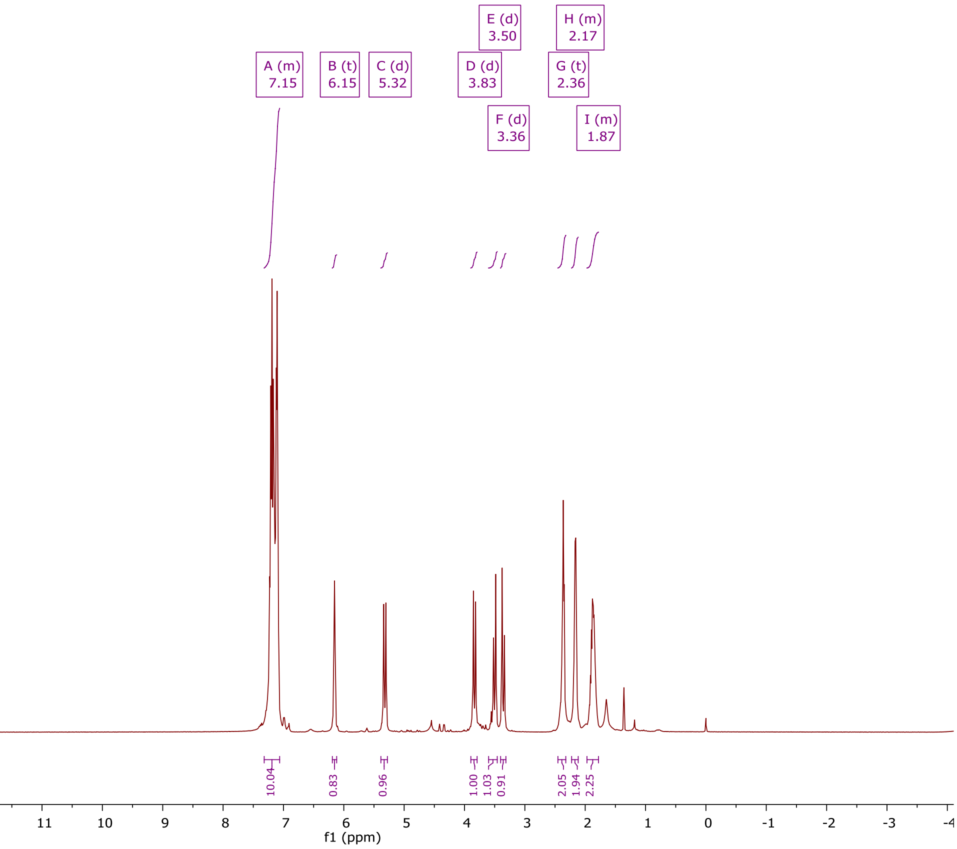

A (m) 7.15
B (t) 6.15
C (d) 5.32
D (d) 3.83
E (d) 3.50
F (d) 3.36
G (t) 2.36
H (m) 2.17
I (m) 1.87
10.04
0.83
0.96
1.00
1.03
0.91
2.05
1.94
2.25
15 14 13 12 11 10 9 8 7 6 5 4 3 2 1 0 -1 -2 -3 -4
f1 (ppm)

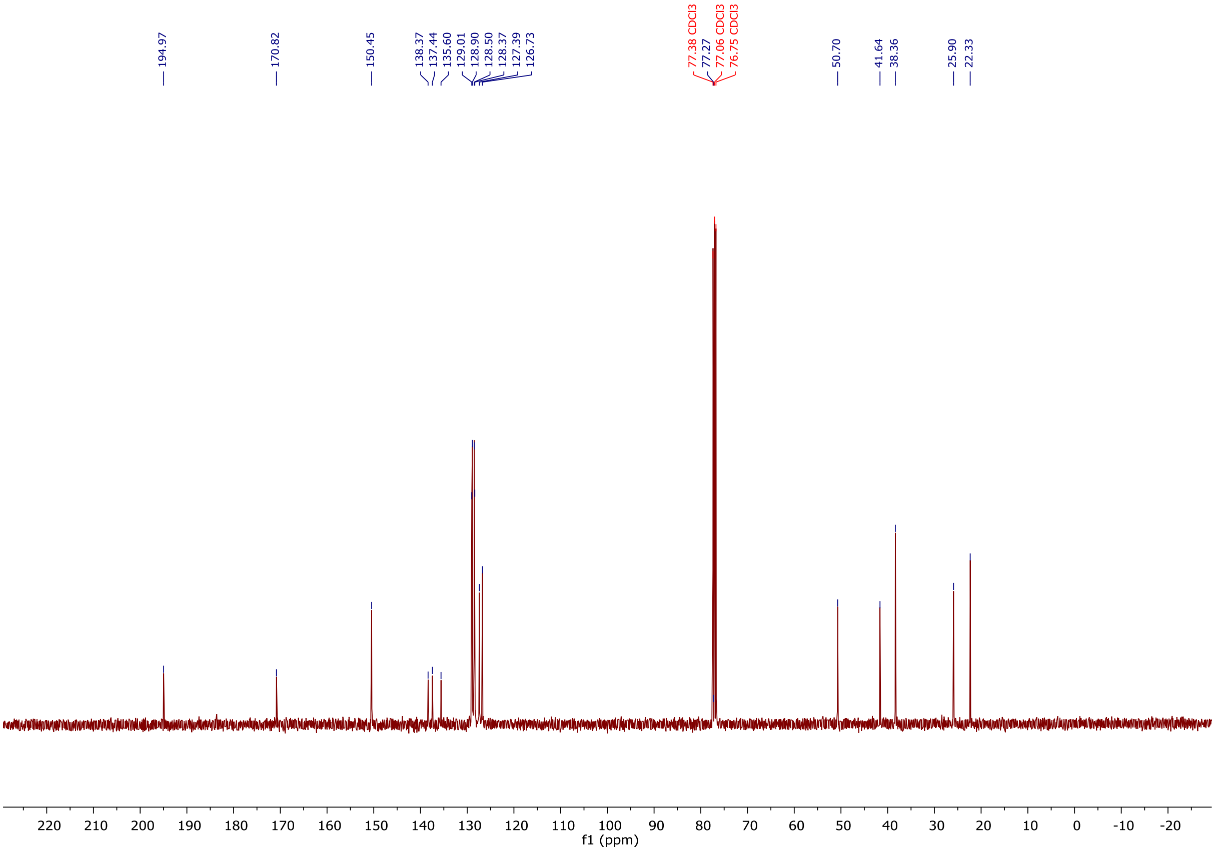

194.97
170.82
150.45
138.37
137.44
135.60
129.01
128.90
128.50
128.37
127.39
126.73
77.38 CDCl3
77.27
77.06 CDCl3
76.75 CDCl3
50.70
41.64
38.36
25.90
22.33
220 210 200 190 180 170 160 150 140 130 120 110 100 90 80 70 60 50 40 30 20 10 0 -10 -20
f1 (ppm)

Compound 1e

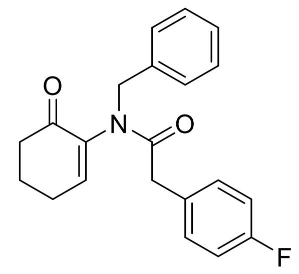

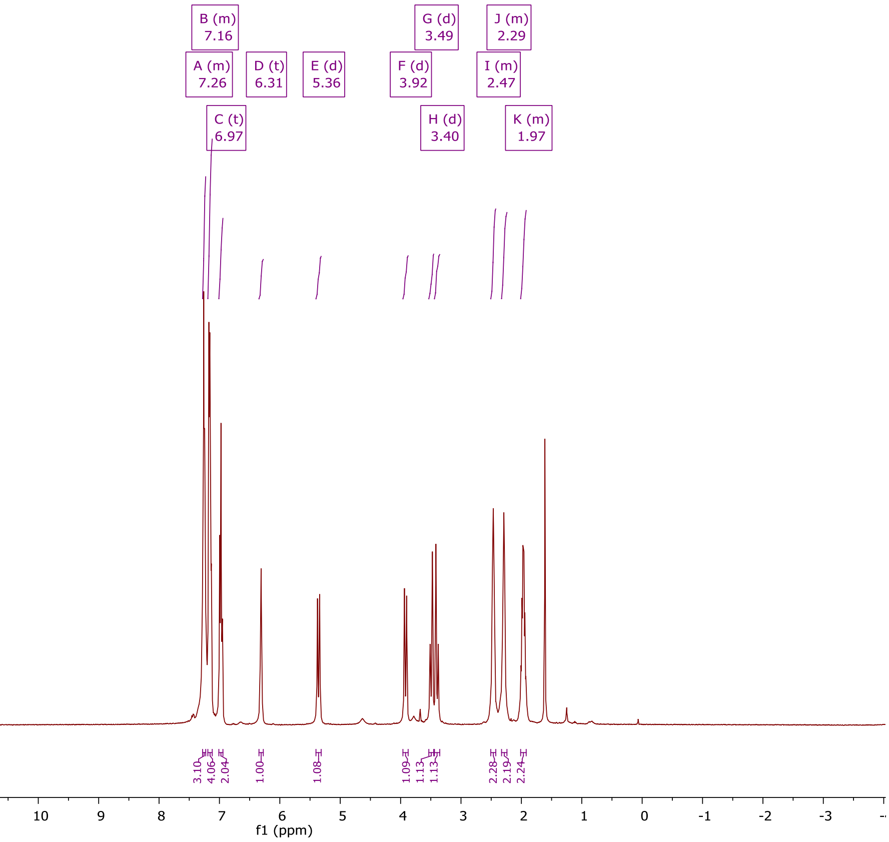

B (m)
7.16
A (m)
7.26
C (t)
6.97
D (t)
6.31
E (d)
5.36
F (d)
3.92
G (d)
3.49
H (d)
3.40
I (m)
2.47
J (m)
2.29
K (m)
1.97
3.10
4.06
2.04
1.00
1.08
1.09
1.13
1.13
2.28
2.19
2.24
15 14 13 12 11 10 9 8 7 6 5 4 3 2 1 0 -1 -2 -3
f1 (ppm)

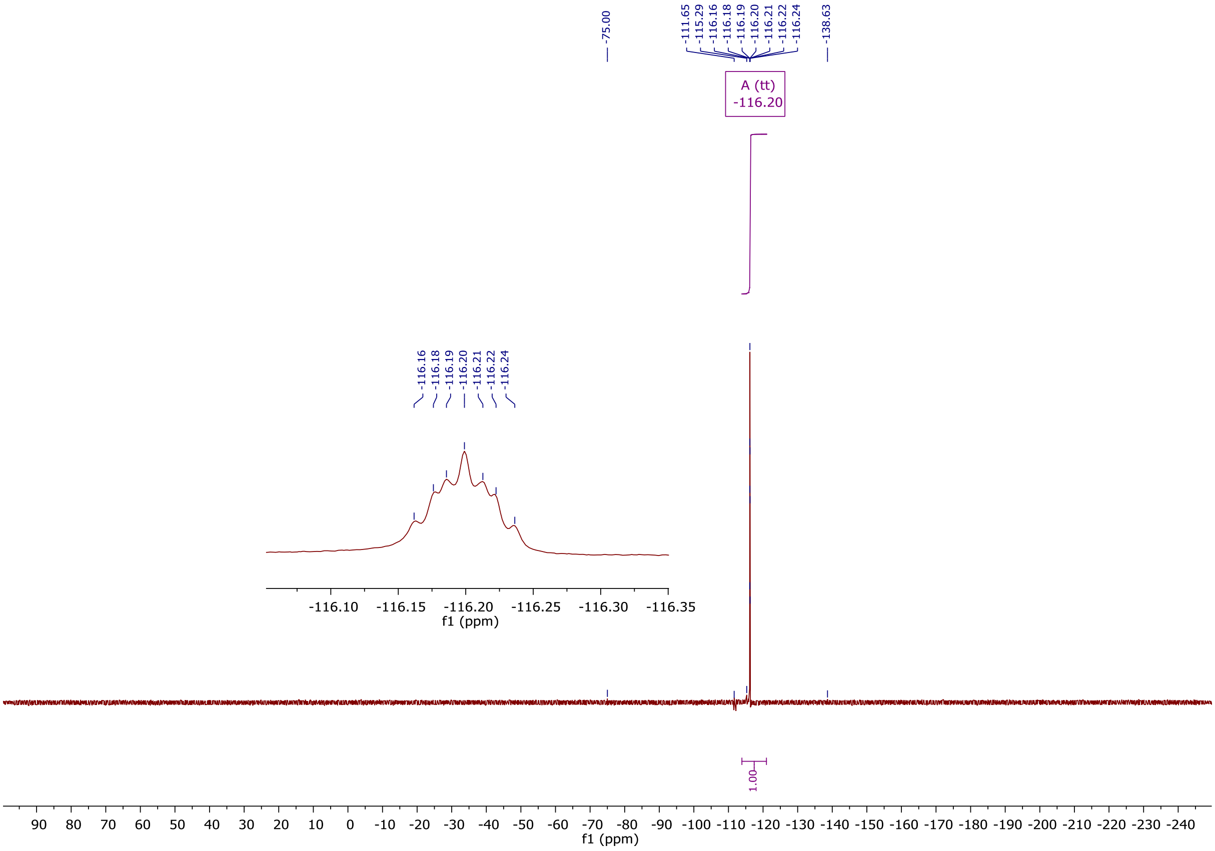

-75.00
-111.65
-115.29
-116.16
-116.18
-116.19
-116.20
-116.21
-116.22
-116.24
-138.63
A (tt)
-116.20
-116.16
-116.18
-116.19
-116.20
-116.21
-116.22
-116.24
-116.10 -116.15 -116.20 -116.25 -116.30 -116.35
f1 (ppm)
1.00
90 80 70 60 50 40 30 20 10 0 -10 -20 -30 -40 -50 -60 -70 -80 -90 -100 -110 -120 -130 -140 -150 -160 -170 -180 -190 -200 -210 -220 -230 -240
f1 (ppm)

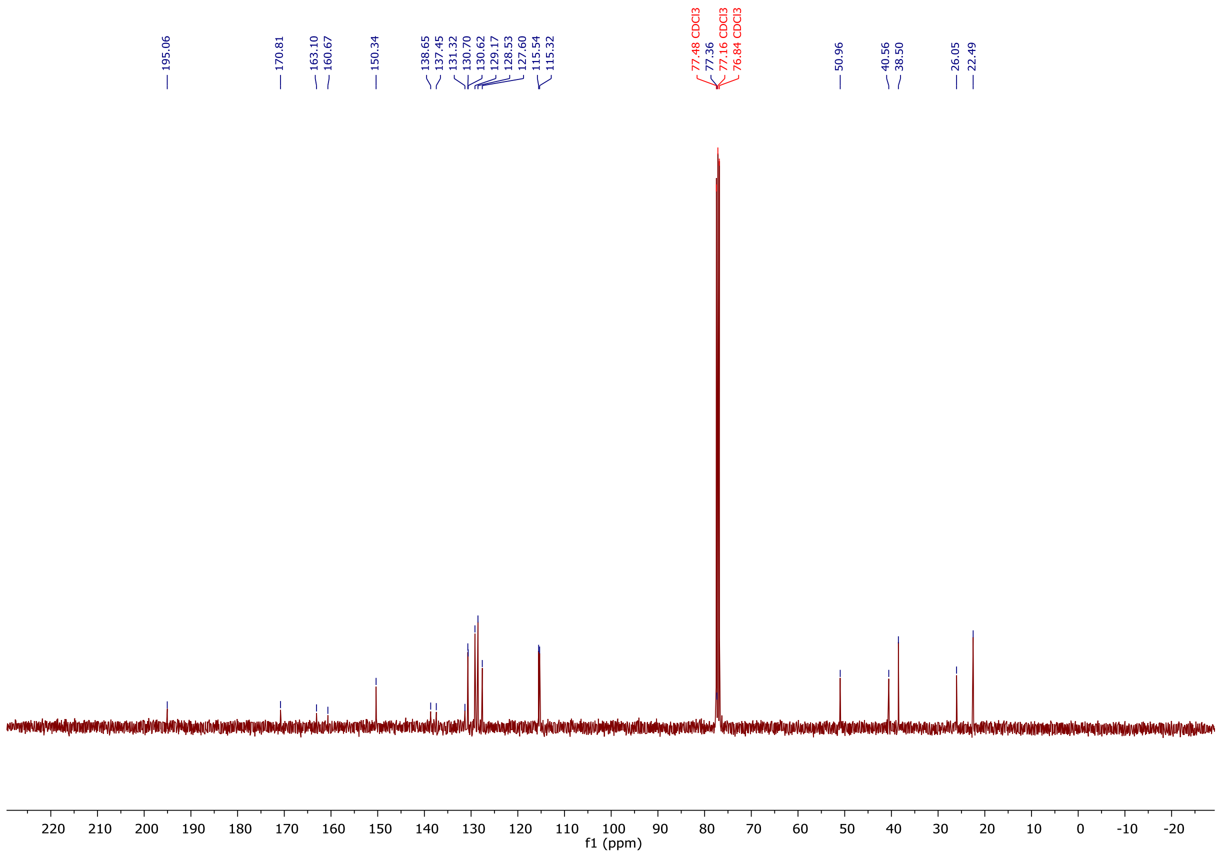
195.06
170.81
163.10
160.67
150.34
138.65
137.45
131.32
130.70
130.62
129.17
128.53
127.60
115.54
115.32
77.48 CDCl3
77.36
77.16 CDCl3
76.84 CDCl3
50.96
40.56
38.50
26.05
22.49
f1 (ppm)

Compound 1f

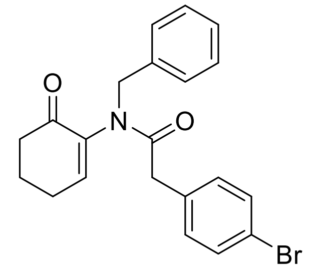

O
N
O
Br

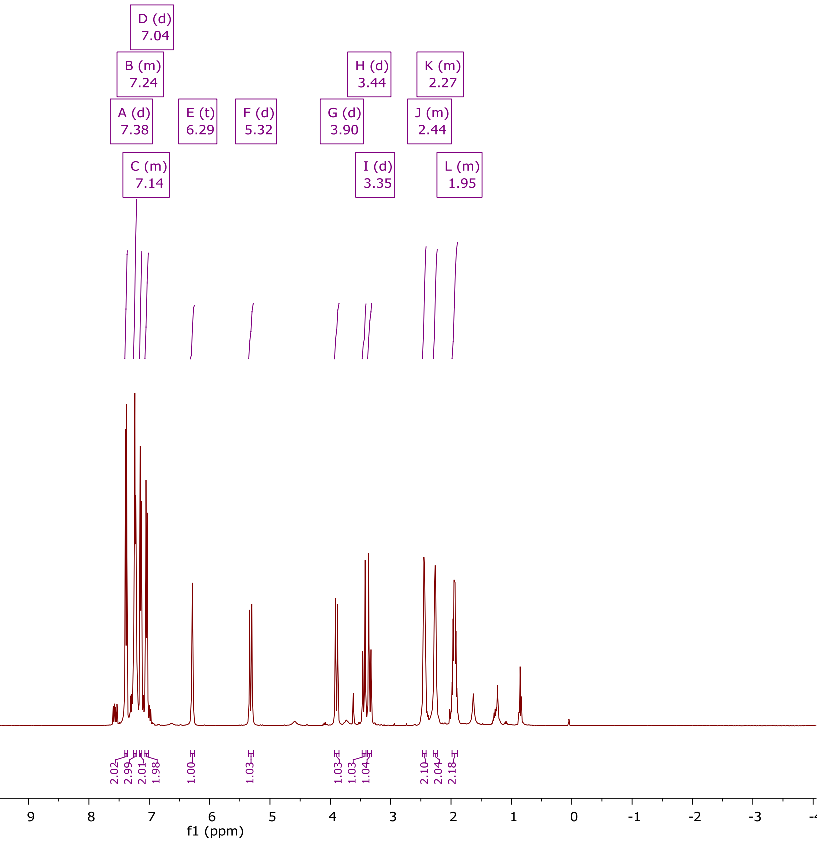

D (d) 7.04
B (m) 7.24
A (d) 7.38
C (m) 7.14
E (t) 6.29
F (d) 5.32
G (d) 3.90
H (d) 3.44
I (d) 3.35
J (m) 2.44
K (m) 2.27
L (m) 1.95
2.02
2.99
2.01
1.98
1.00
1.03
1.03
1.03
1.04
2.10
2.04
2.18
f1 (ppm)

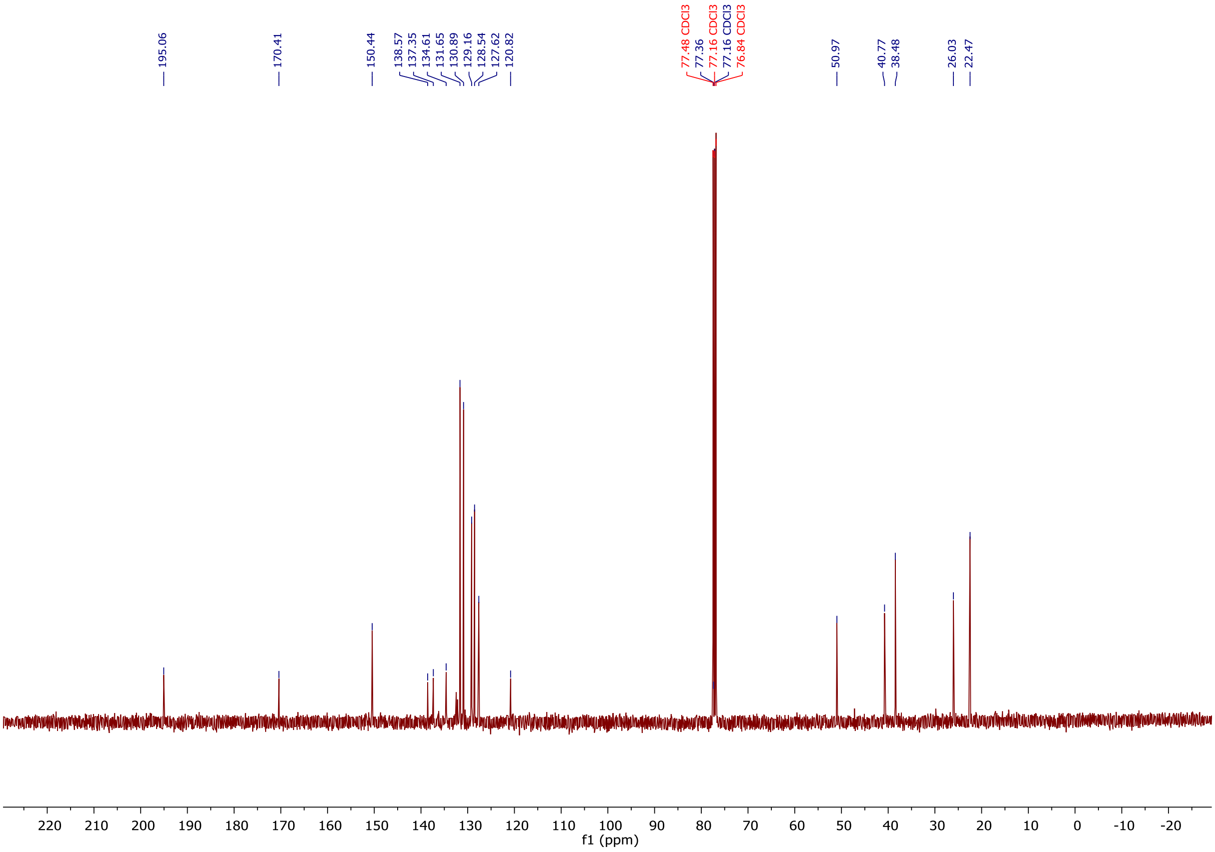

195.06
170.41
150.44
138.57
137.35
134.61
131.65
130.89
129.16
128.54
127.62
120.82
77.48 CDCl3
77.36
77.16 CDCl3
77.16 CDCl3
76.84 CDCl3
50.97
40.77
38.48
26.03
22.47
f1 (ppm)

Compound 1g

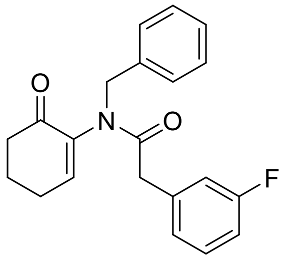

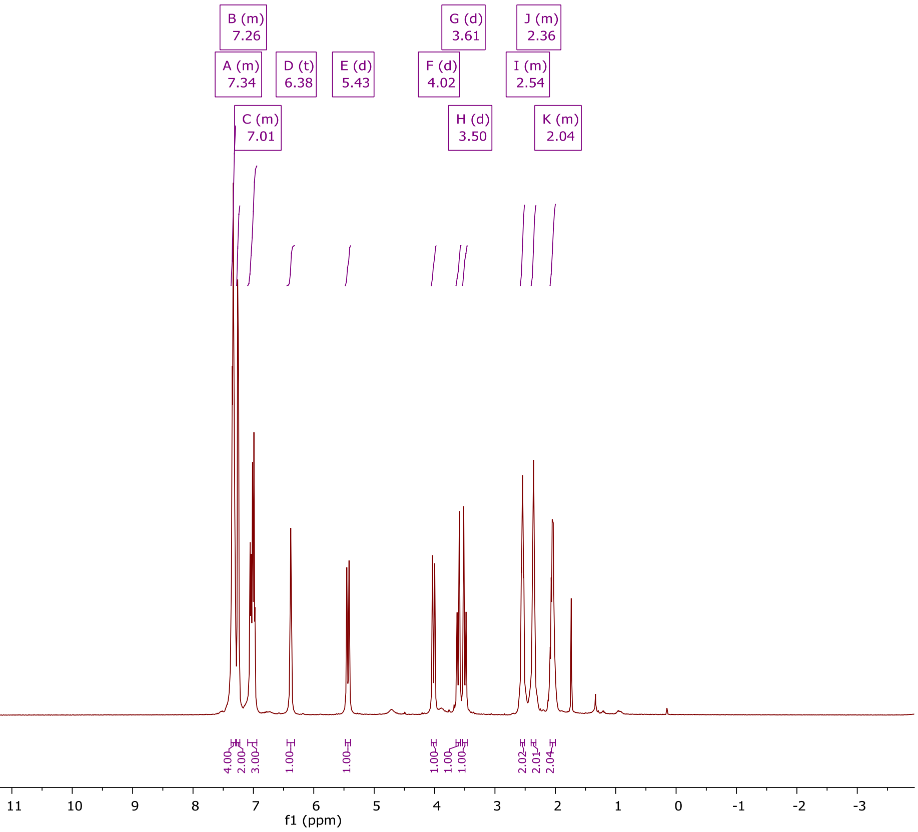

B (m) 7.26
A (m) 7.34
C (m) 7.01
D (t) 6.38
E (d) 5.43
F (d) 4.02
G (d) 3.61
H (d) 3.50
I (m) 2.54
J (m) 2.36
K (m) 2.04
f1 (ppm)

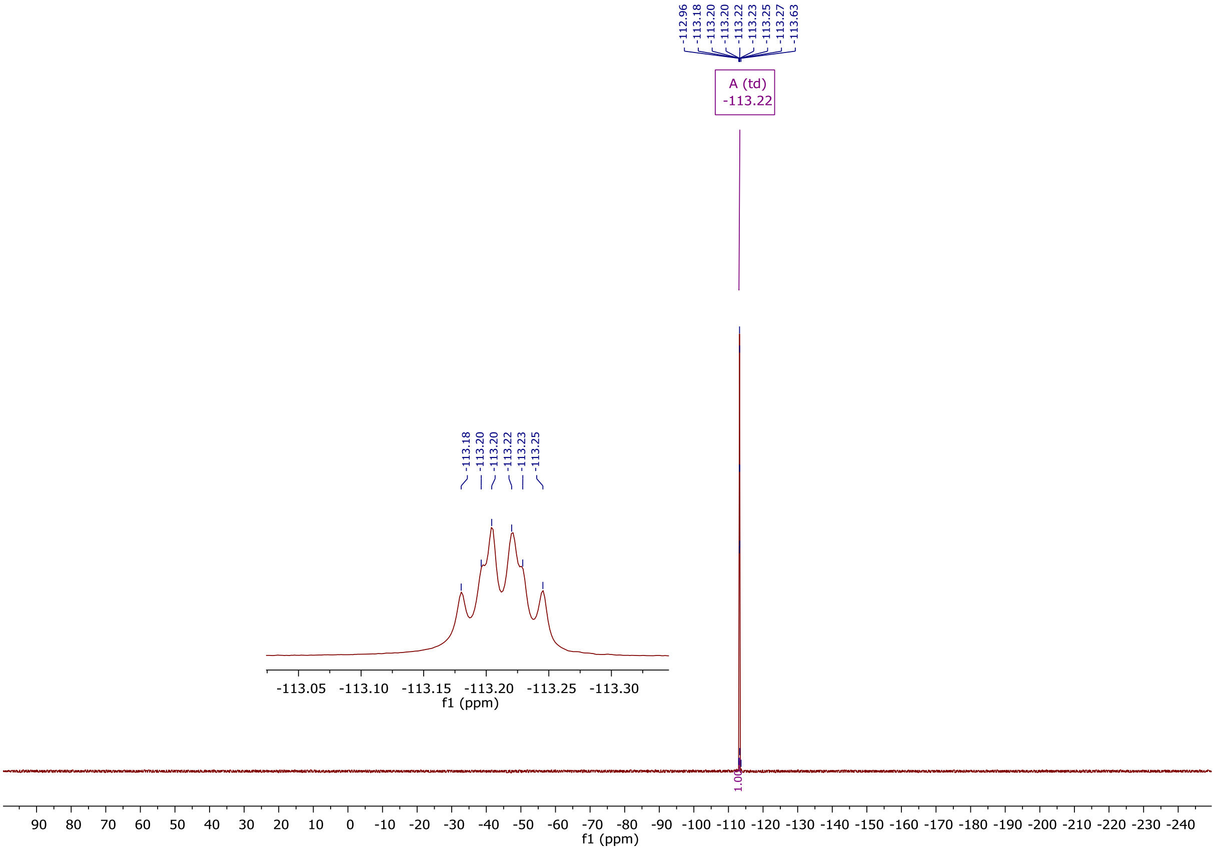

-112.96 -113.18 -113.20 -113.20 -113.22 -113.23 -113.25 -113.27 -113.63
A (td) -113.22
-113.18 -113.20 -113.20 -113.22 -113.23 -113.25
f1 (ppm)

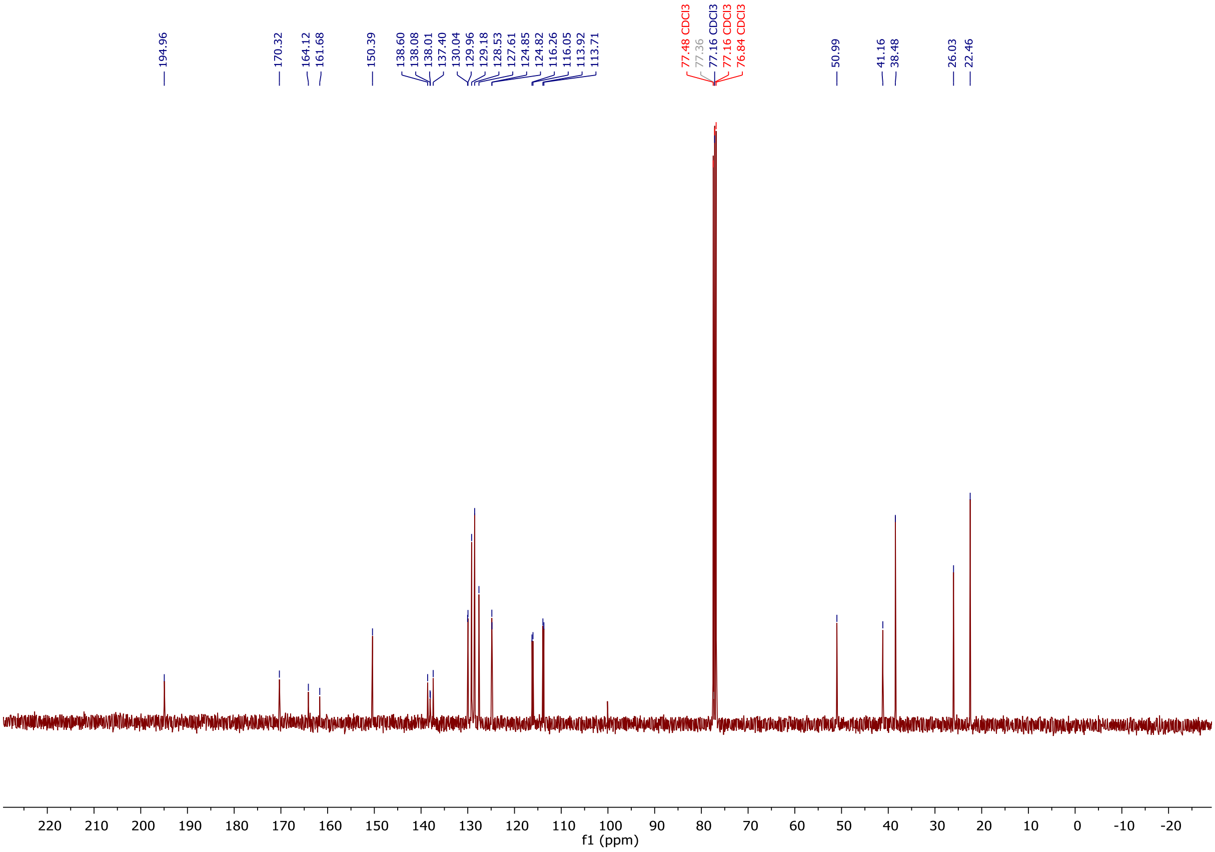
194.96
170.32
164.12
161.68
150.39
138.60
138.08
138.01
137.40
130.04
129.96
129.18
128.53
127.61
124.85
124.82
116.26
116.05
113.92
113.71
77.48 CDCl3
77.36
77.16 CDCl3
77.16 CDCl3
76.84 CDCl3
50.99
41.16
38.48
26.03
22.46
220 210 200 190 180 170 160 150 140 130 120 110 100 90 80 70 60 50 40 30 20 10 0 -10 -20
f1 (ppm)

Compound 1h

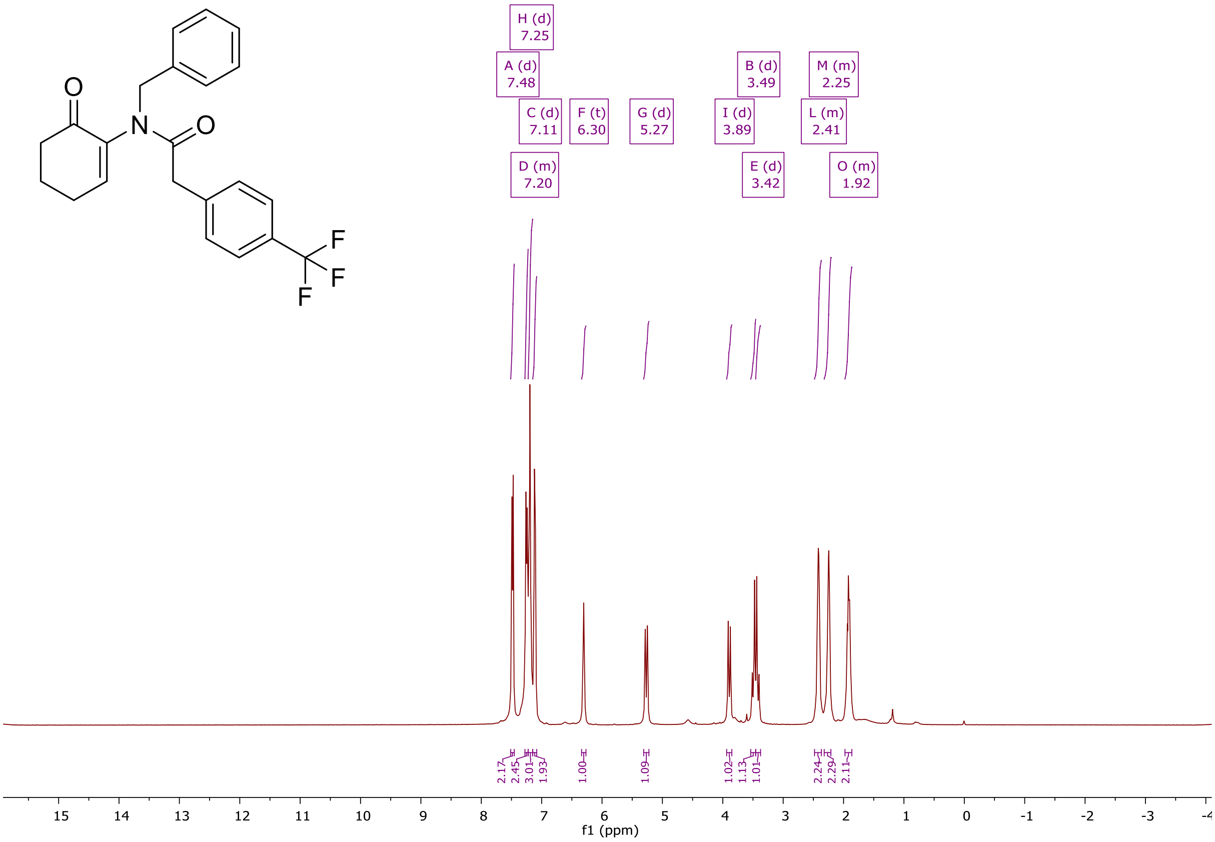

H (d) 7.25
A (d) 7.48
C (d) 7.11
F (t) 6.30
G (d) 5.27
D (m) 7.20
I (d) 3.89
B (d) 3.49
E (d) 3.42
M (m) 2.25
L (m) 2.41
O (m) 1.92
2.17
2.45
3.01
1.93
1.00
1.09
1.02
1.13
1.01
2.24
2.29
2.11
15 14 13 12 11 10 9 8 7 6 5 4 3 2 1 0 -1 -2 -3
f1 (ppm)

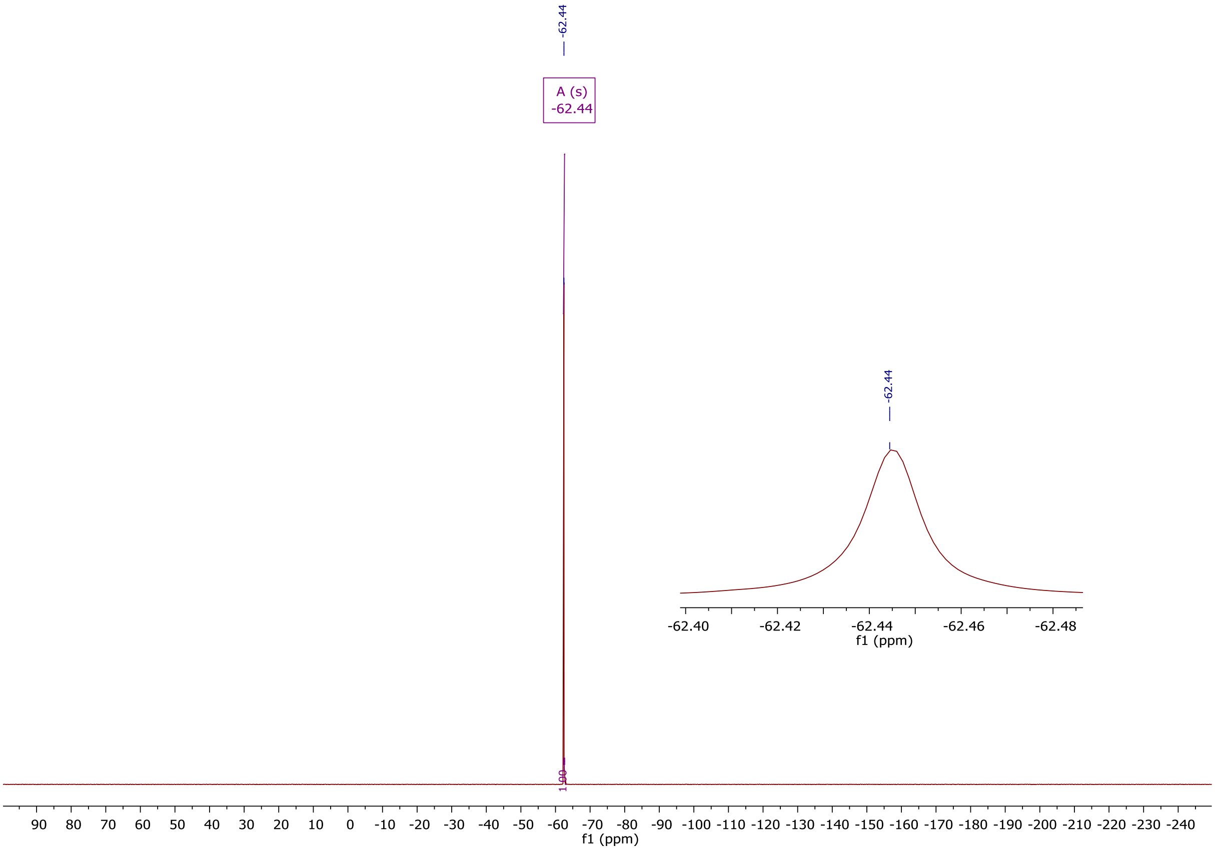

-62.44
A (s) -62.44
-62.44
-62.40 -62.42 -62.44 -62.46 -62.48
f1 (ppm)
1.00
90 80 70 60 50 40 30 20 10 0 -10 -20 -30 -40 -50 -60 -70 -80 -90 -100 -110 -120 -130 -140 -150 -160 -170 -180 -190 -200 -210 -220 -230 -240
f1 (ppm)

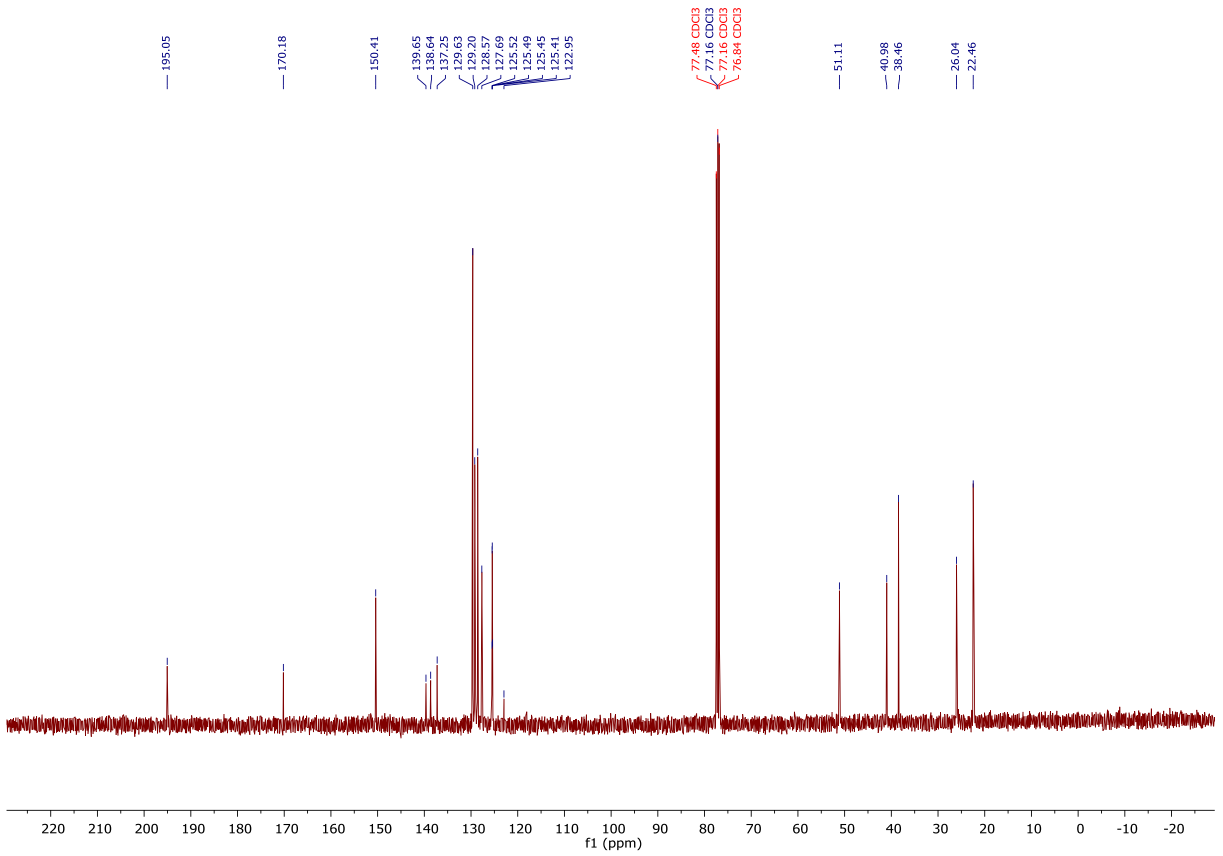
195.05
170.18
150.41
139.65
138.64
137.25
129.63
129.20
128.57
127.69
125.52
125.49
125.45
125.41
122.95
77.48 CDCl3
77.16 CDCl3
77.16 CDCl3
76.84 CDCl3
51.11
40.98
38.46
26.04
22.46
f1 (ppm)

Compound 1i

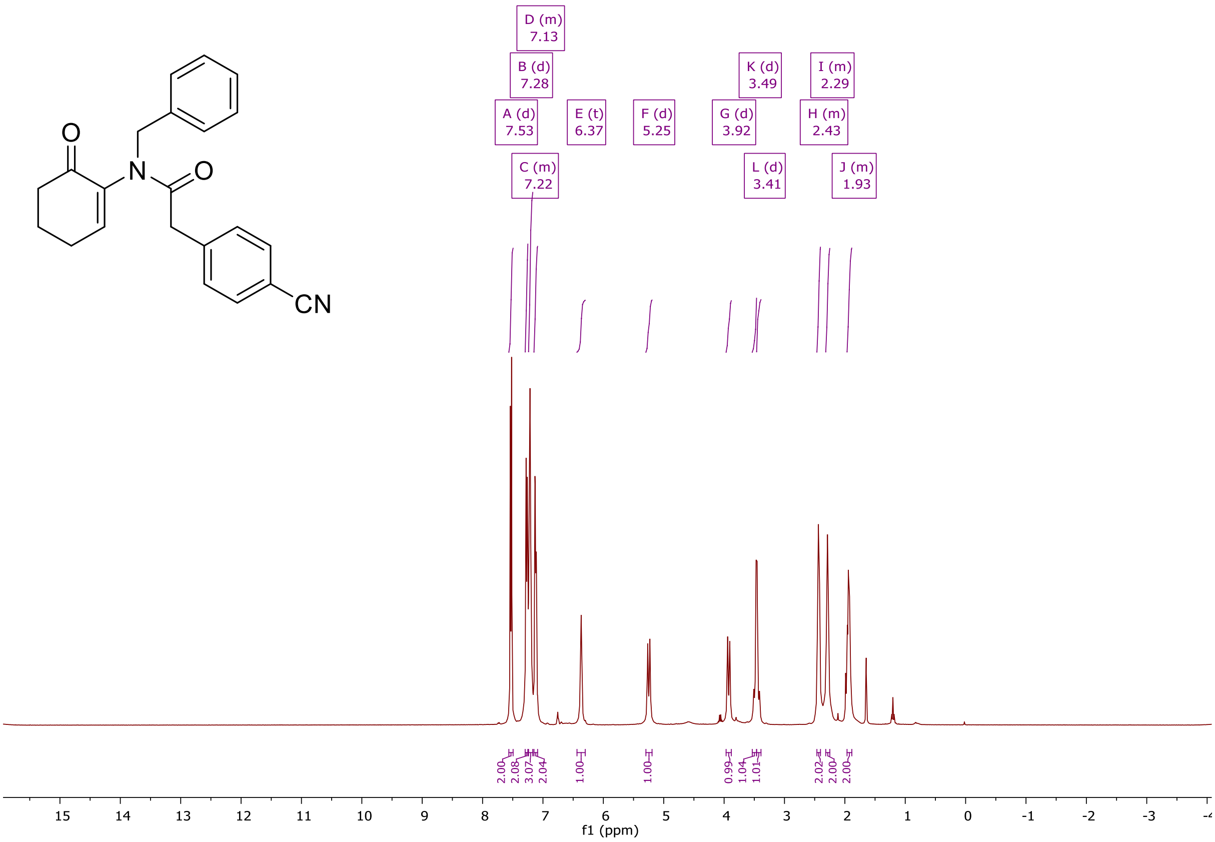
O
N
O
CN
D (m) 7.13
B (d) 7.28
A (d) 7.53
C (m) 7.22
E (t) 6.37
F (d) 5.25
G (d) 3.92
K (d) 3.49
L (d) 3.41
I (m) 2.29
H (m) 2.43
J (m) 1.93
2.00
2.08
3.07
2.04
1.00
1.00
0.99
1.04
1.01
2.02
2.00
2.00
15 14 13 12 11 10 9 8 7 6 5 4 3 2 1 0 -1 -2 -3
f1 (ppm)

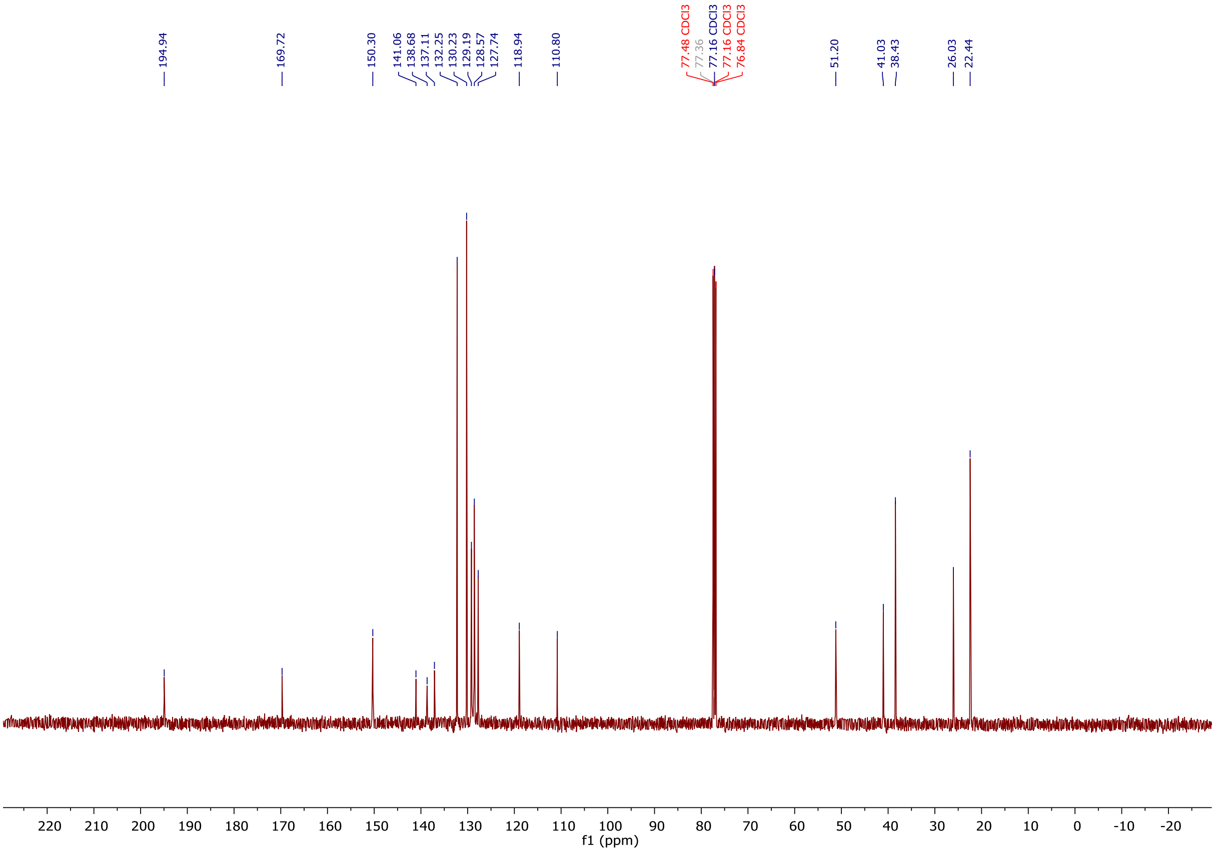
194.94
169.72
150.30
141.06
138.68
137.11
132.25
130.23
129.19
128.57
127.74
118.94
110.80
77.48 CDCl3
77.36
77.16 CDCl3
77.16 CDCl3
76.84 CDCl3
51.20
41.03
38.43
26.03
22.44
220 210 200 190 180 170 160 150 140 130 120 110 100 90 80 70 60 50 40 30 20 10 0 -10 -20
f1 (ppm)

Compound 1j

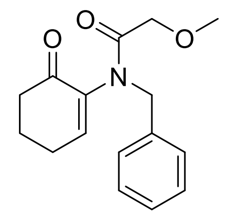

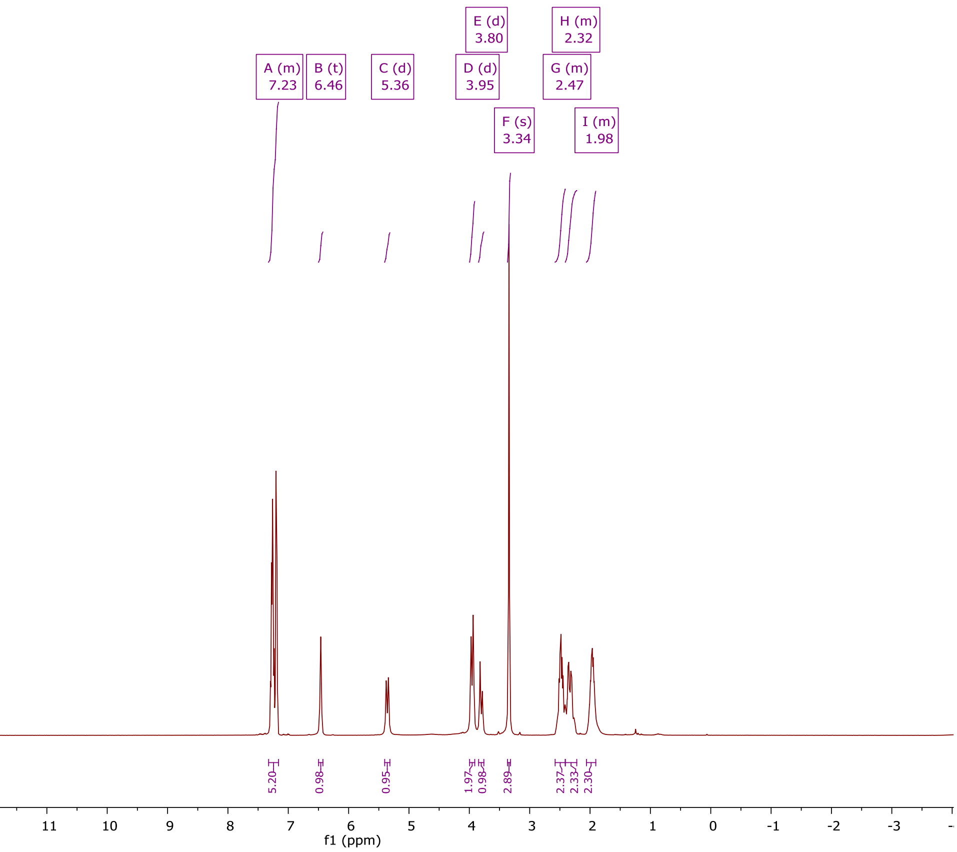
A (m)
7.23
B (t)
6.46
C (d)
5.36
D (d)
3.95
E (d)
3.80
F (s)
3.34
G (m)
2.47
H (m)
2.32
I (m)
1.98
5.20
0.98
0.95
1.97
0.98
2.89
2.37
2.33
2.30
f1 (ppm)

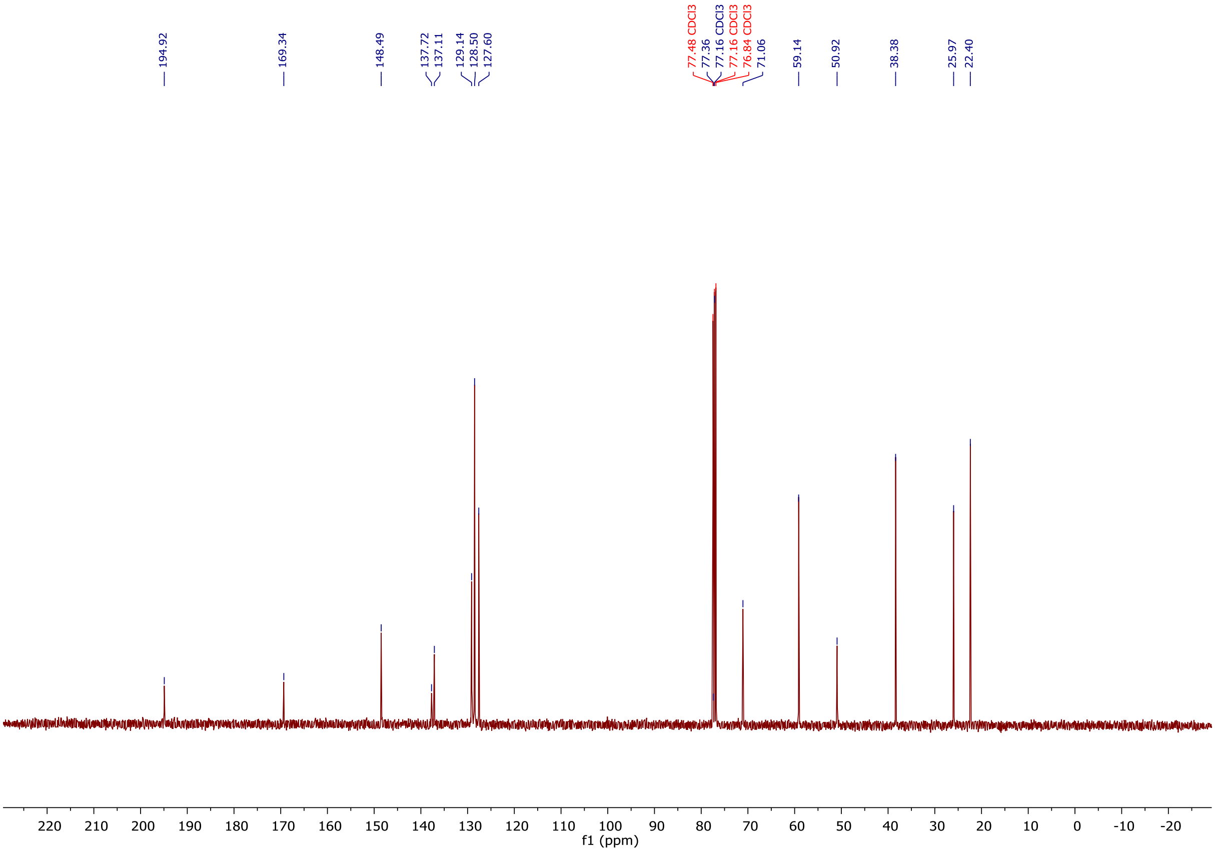
194.92
169.34
148.49
137.72
137.11
129.14
128.50
127.60
77.48 CDCl3
77.36
77.16 CDCl3
77.16 CDCl3
76.84 CDCl3
71.06
59.14
50.92
38.38
25.97
22.40
f1 (ppm)

Compound 1l

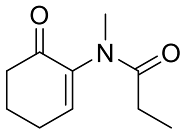

O
N
O

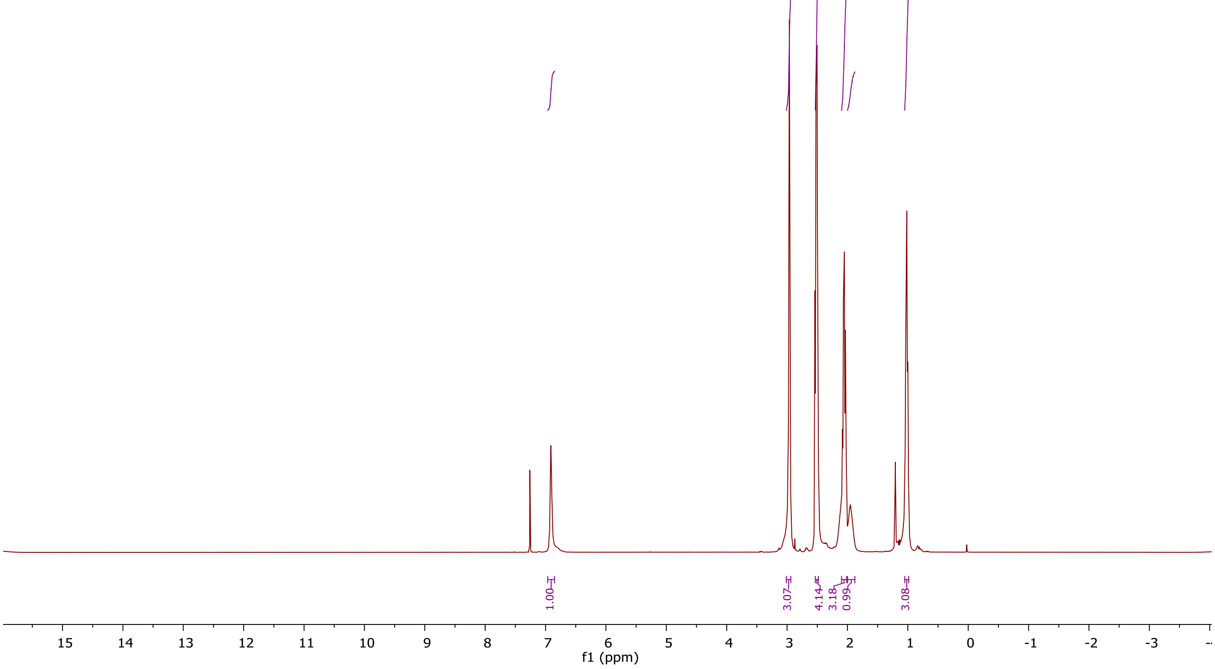

A (t) 6.91
B (s) 2.96
C (m) 2.51
D (m) 2.06
E (m) 1.94
F (t) 1.02
1.00
3.07
4.14
3.18
0.99
3.08
f1 (ppm)

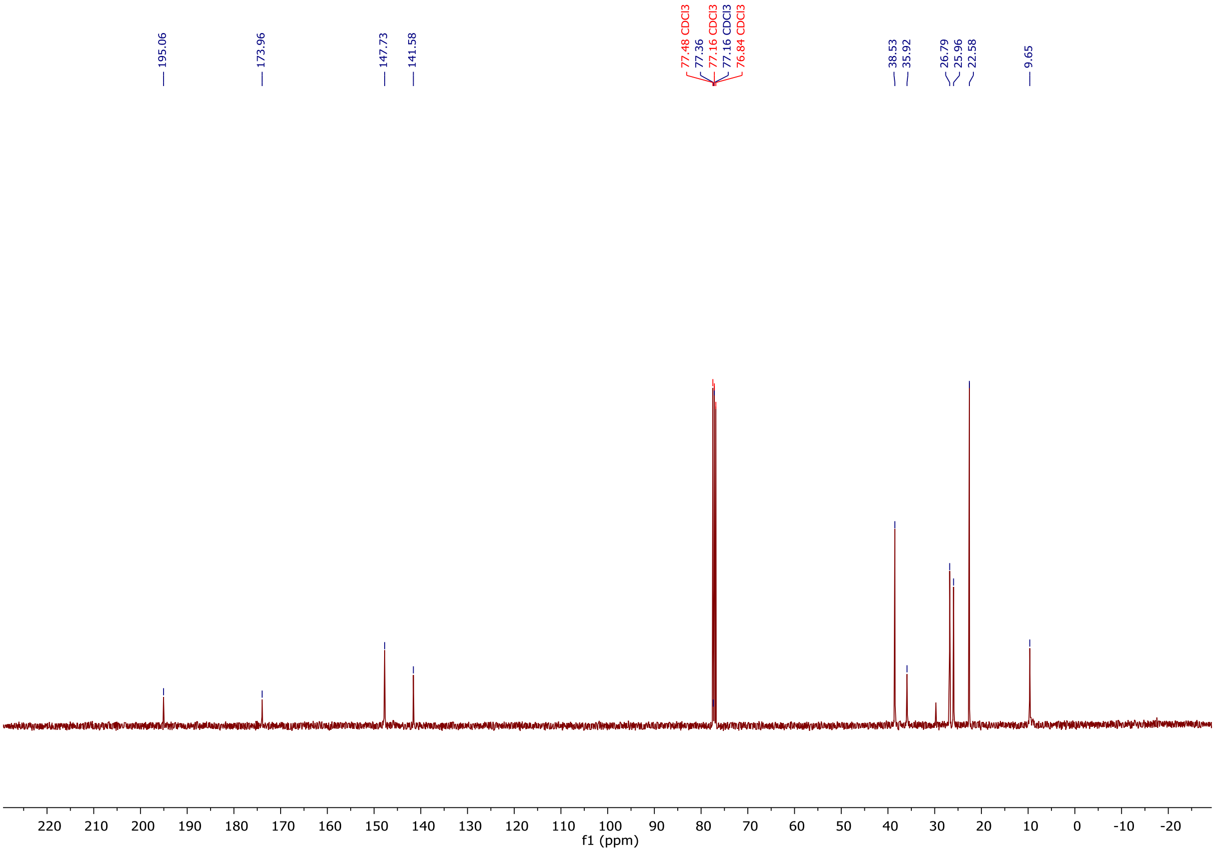

195.06
173.96
147.73
141.58
77.48 CDCl3
77.36
77.16 CDCl3
77.16 CDCl3
76.84 CDCl3
38.53
35.92
26.79
25.96
22.58
9.65
f1 (ppm)

Compound 1m

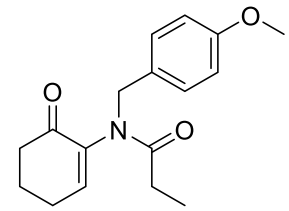

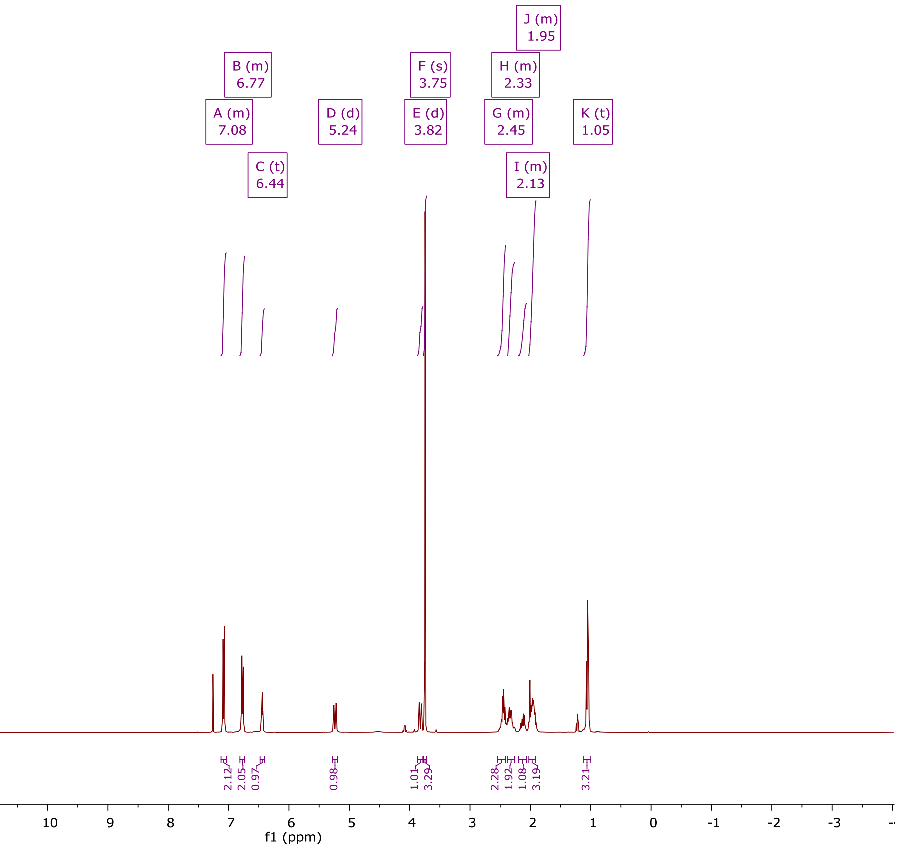
A (m)
7.08
B (m)
6.77
C (t)
6.44
D (d)
5.24
E (d)
3.82
F (s)
3.75
G (m)
2.45
H (m)
2.33
I (m)
2.13
J (m)
1.95
K (t)
1.05
2.12
2.05
0.97
0.98
1.01
3.29
2.28
1.92
1.08
3.19
3.21
f1 (ppm)

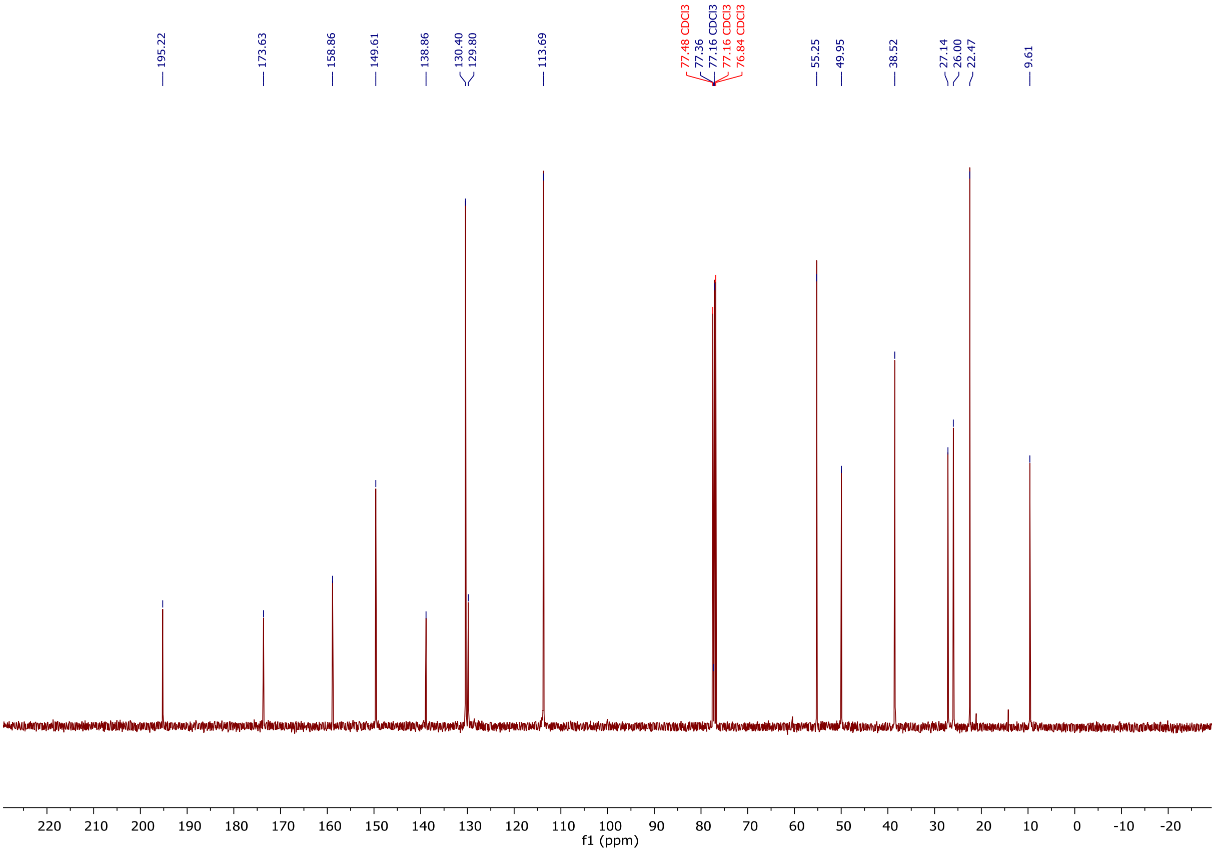
195.22
173.63
158.86
149.61
138.86
130.40
129.80
113.69
77.48 CDCl3
77.36
77.16 CDCl3
77.16 CDCl3
76.84 CDCl3
55.25
49.95
38.52
27.14
26.00
22.47
9.61
f1 (ppm)

Compound 1n

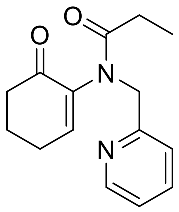

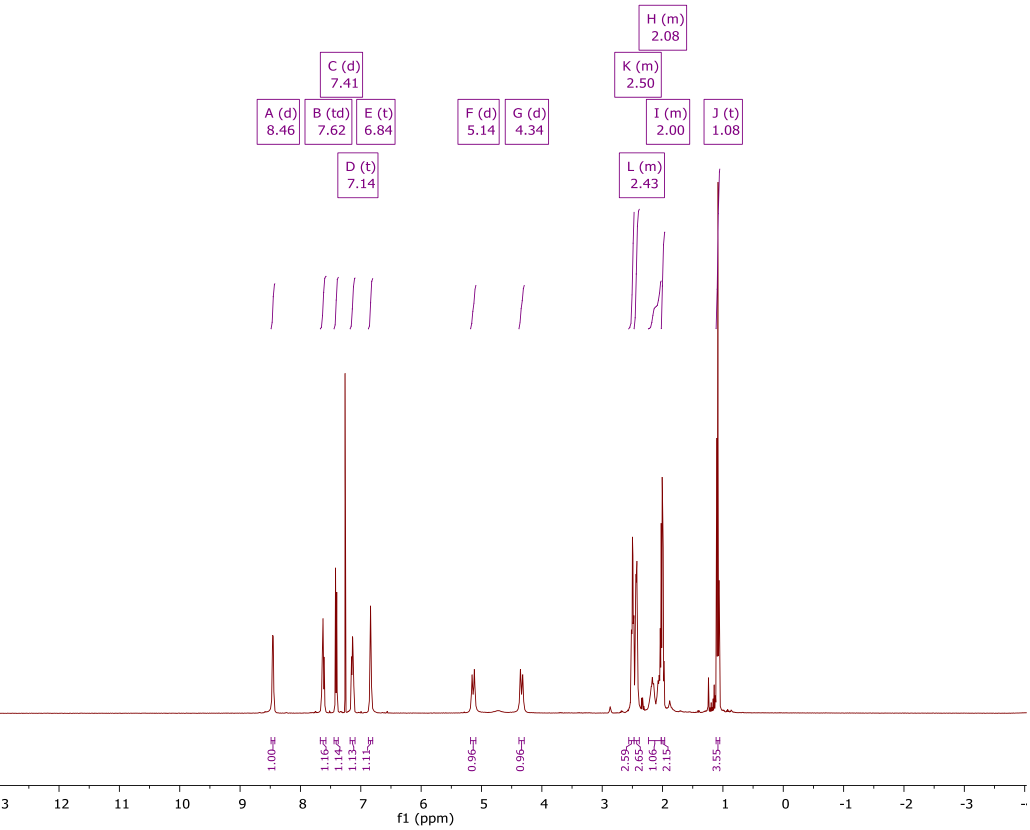
A (d) 8.46
B (td) 7.62
C (d) 7.41
D (t) 7.14
E (t) 6.84
F (d) 5.14
G (d) 4.34
H (m) 2.08
I (m) 2.00
J (t) 1.08
K (m) 2.50
L (m) 2.43
1.00 1.16 1.14 1.13 1.11 0.96 0.96 2.59 2.65 1.06 2.15 3.55
f1 (ppm)

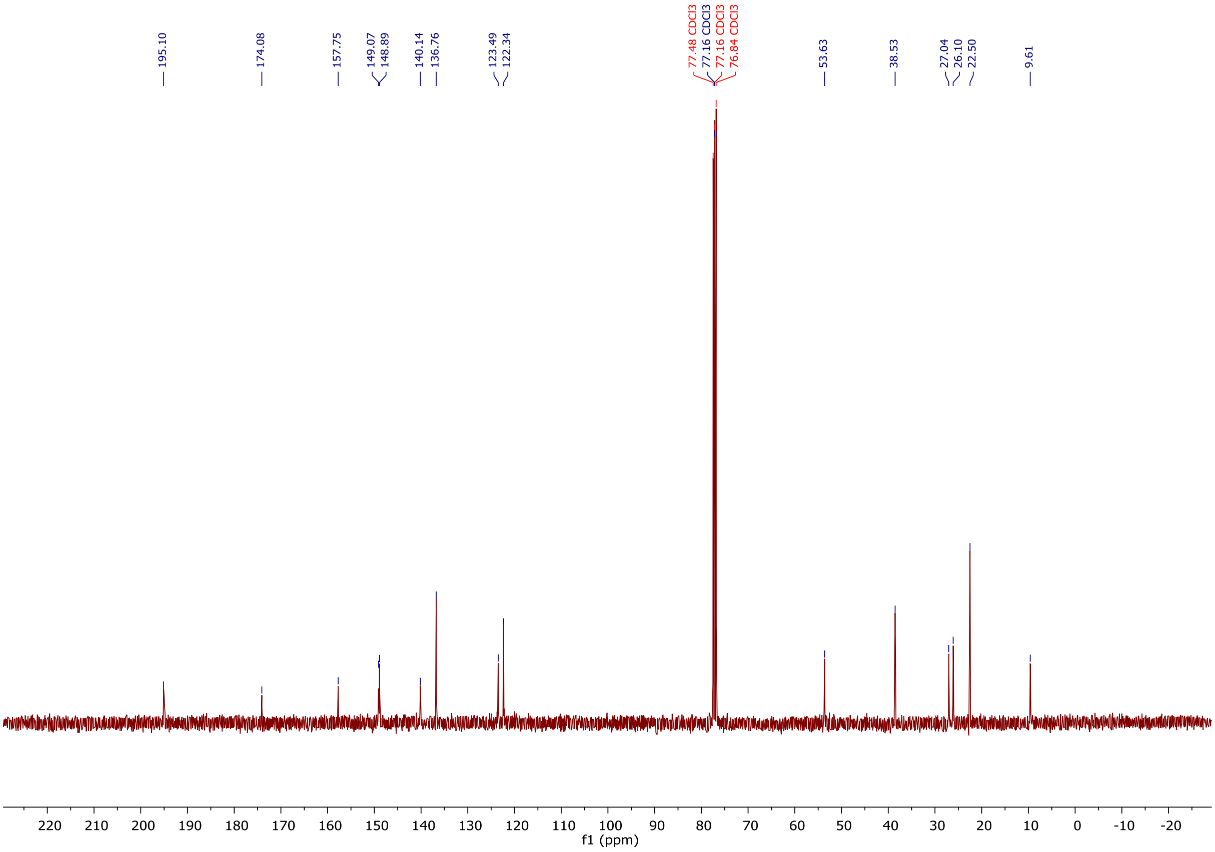
195.10
174.08
157.75
149.07
148.89
140.14
136.76
123.49
122.34
77.48 CDCl3
77.16 CDCl3
77.16 CDCl3
76.84 CDCl3
53.63
38.53
27.04
26.10
22.50
9.61
f1 (ppm)

Compound 1o

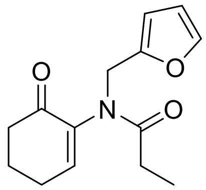

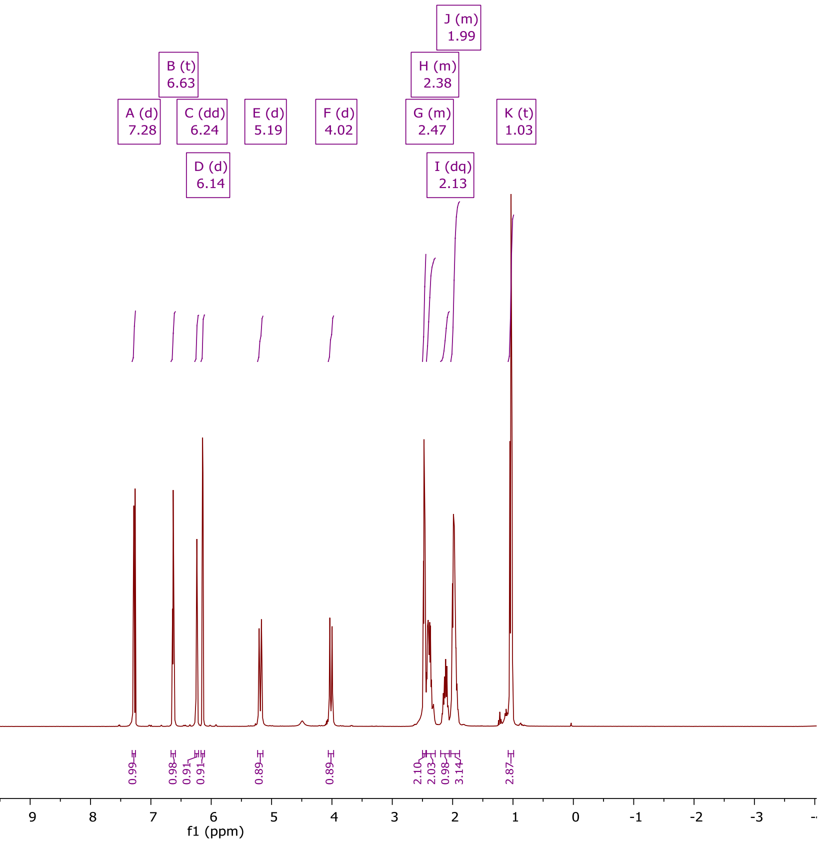

J (m)
1.99
B (t)
6.63
H (m)
2.38
A (d)
7.28
C (dd)
6.24
E (d)
5.19
F (d)
4.02
G (m)
2.47
K (t)
1.03
D (d)
6.14
I (dq)
2.13
0.99
0.98
0.91
0.91
0.89
0.89
2.10
2.03
0.98
3.14
2.87
f1 (ppm)

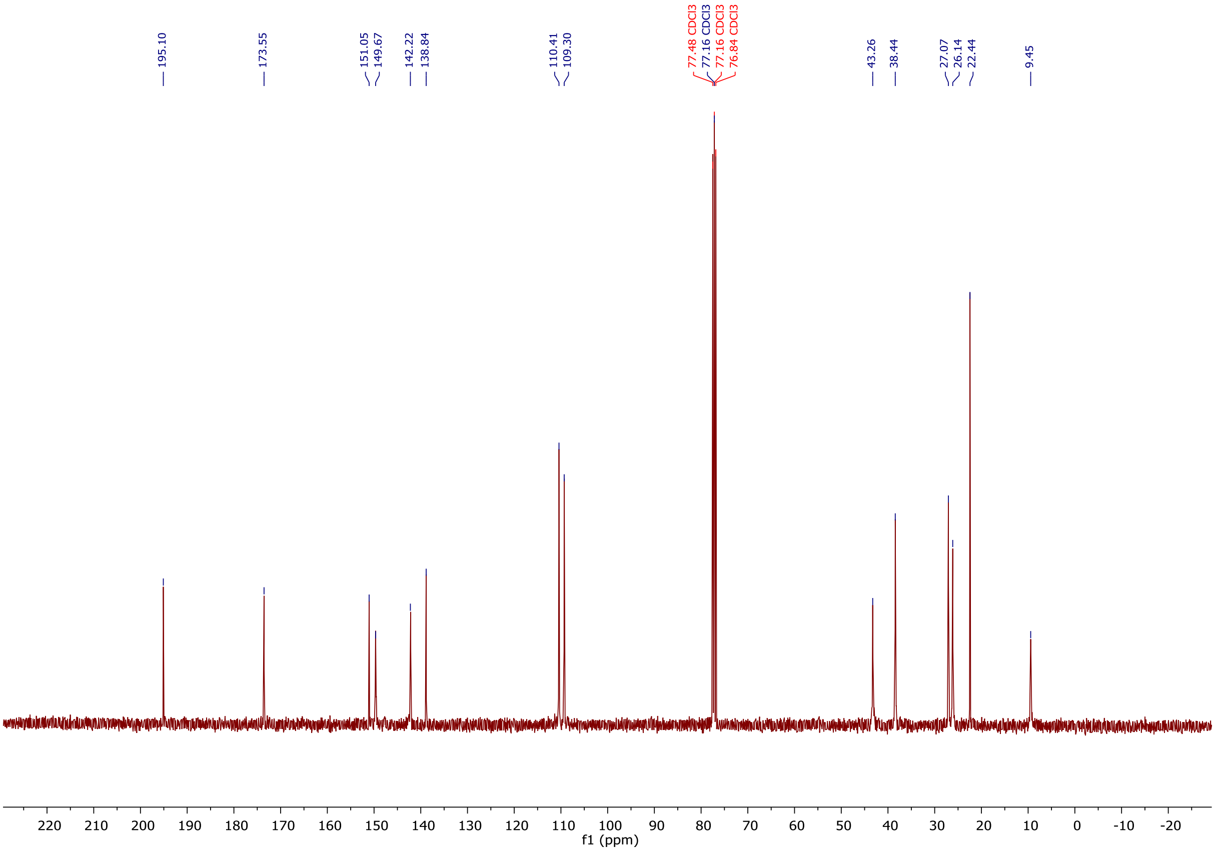

195.10
173.55
151.05
149.67
142.22
138.84
110.41
109.30
77.48 CDCl3
77.16 CDCl3
77.16 CDCl3
76.84 CDCl3
43.26
38.44
27.07
26.14
22.44
9.45
f1 (ppm)

Compound 1p

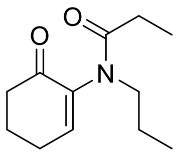

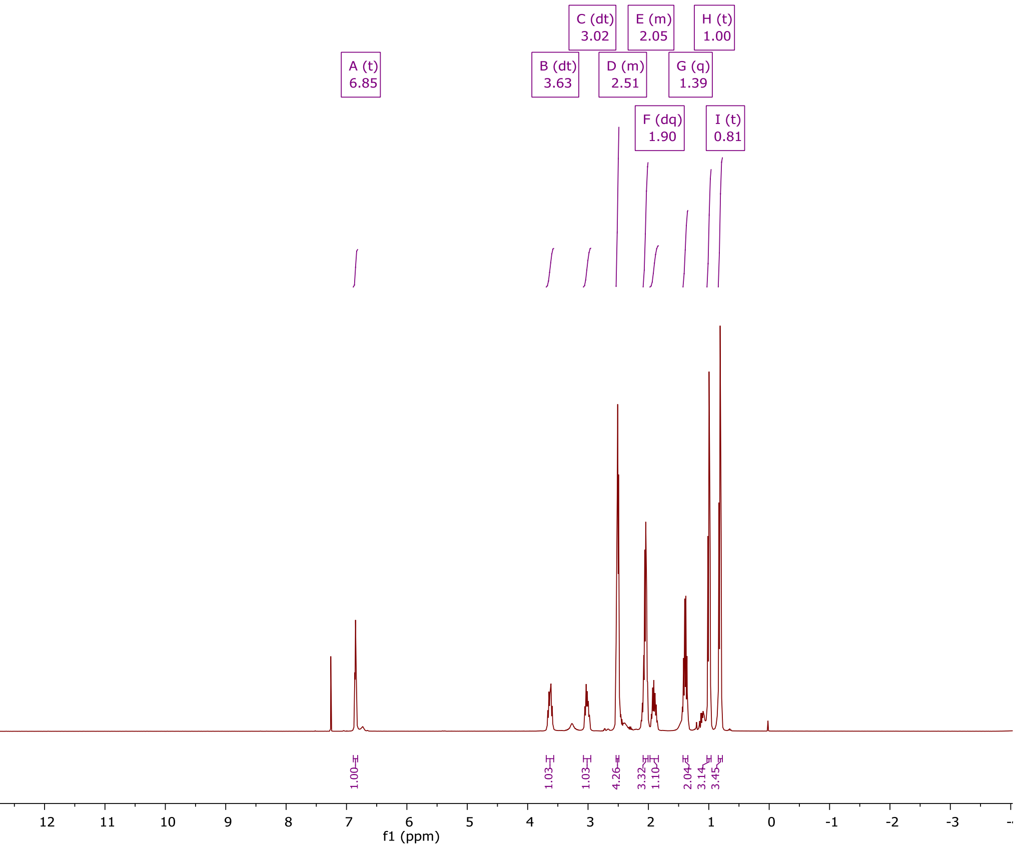

A (t)
6.85
B (dt)
3.63
C (dt)
3.02
D (m)
2.51
E (m)
2.05
F (dq)
1.90
G (q)
1.39
H (t)
1.00
I (t)
0.81
1.00
1.03
1.03
4.26
3.32
1.10
2.04
3.14
3.45
f1 (ppm)

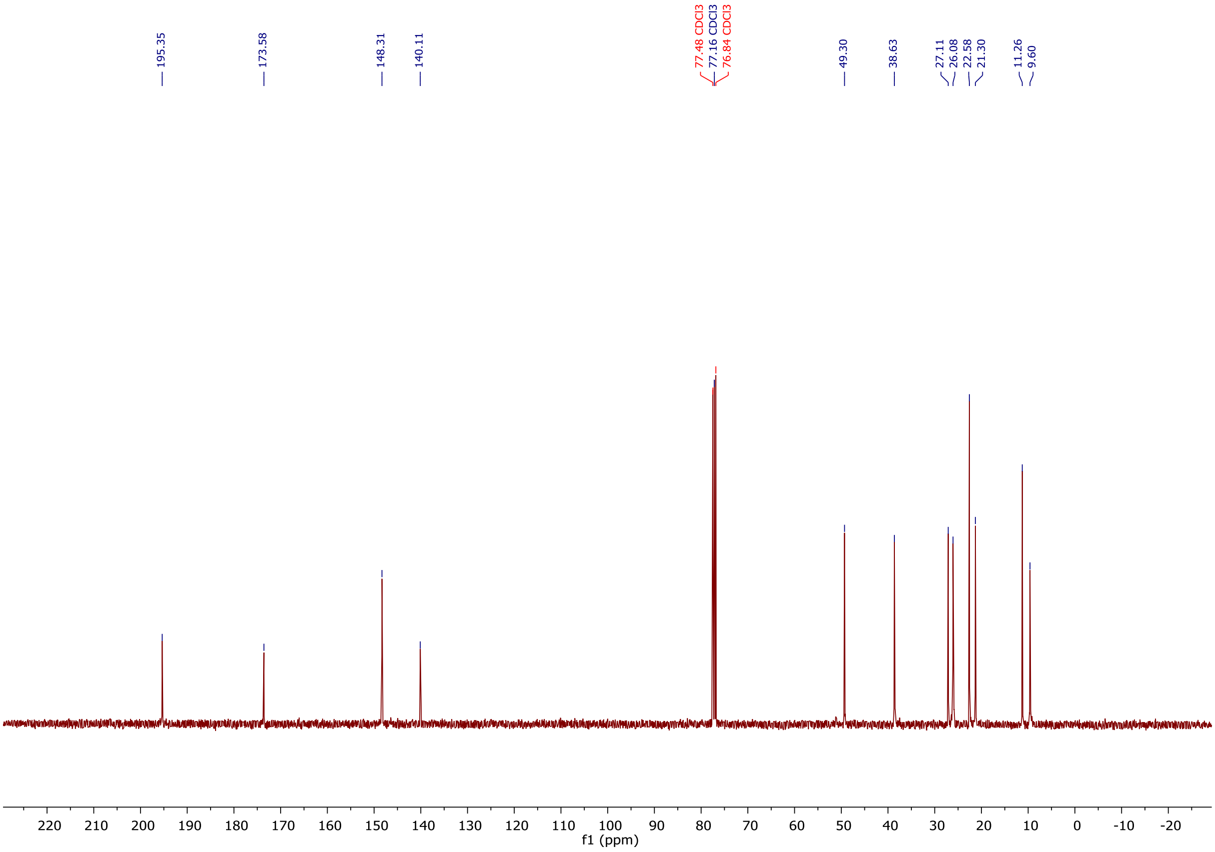

195.35
173.58
148.31
140.11
77.48 CDCl3
77.16 CDCl3
76.84 CDCl3
49.30
38.63
27.11
26.08
22.58
21.30
11.26
9.60
f1 (ppm)

Compound 1q

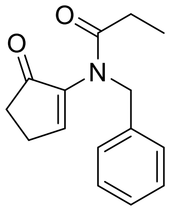

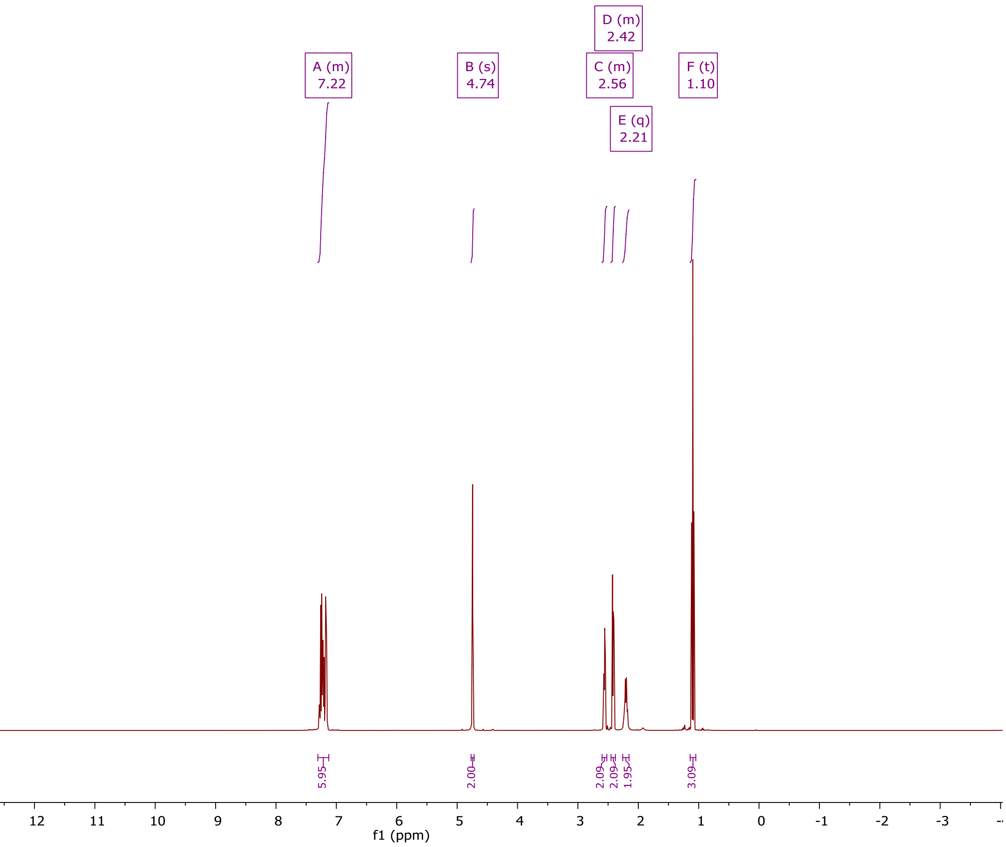
A (m)
7.22
B (s)
4.74
D (m)
2.42
C (m)
2.56
E (q)
2.21
F (t)
1.10
5.95
2.00
2.09
2.09
1.95
3.09
f1 (ppm)

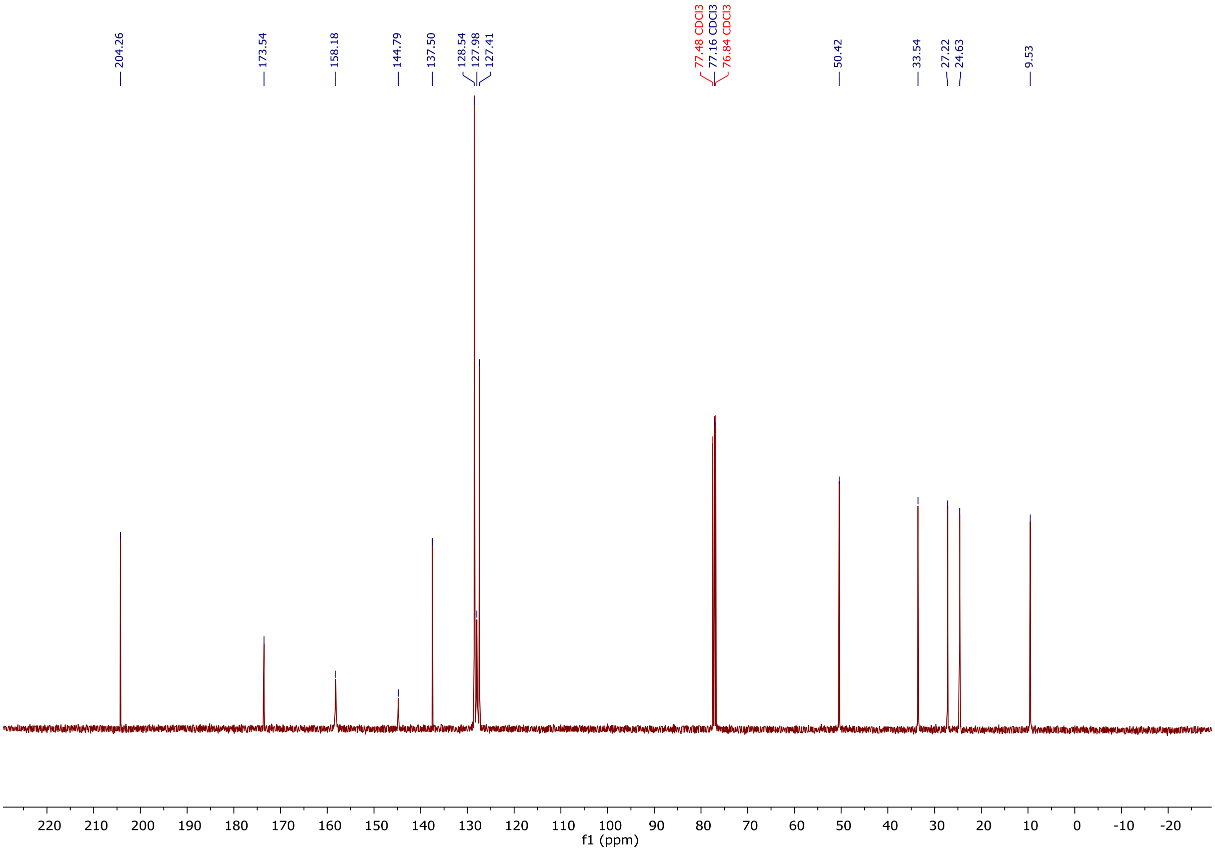
204.26
173.54
158.18
144.79
137.50
128.54
127.98
127.41
77.48 CDCl3
77.16 CDCl3
76.84 CDCl3
50.42
33.54
27.22
24.63
9.53
f1 (ppm)

Compound 1r

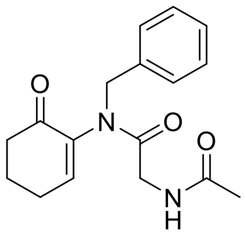

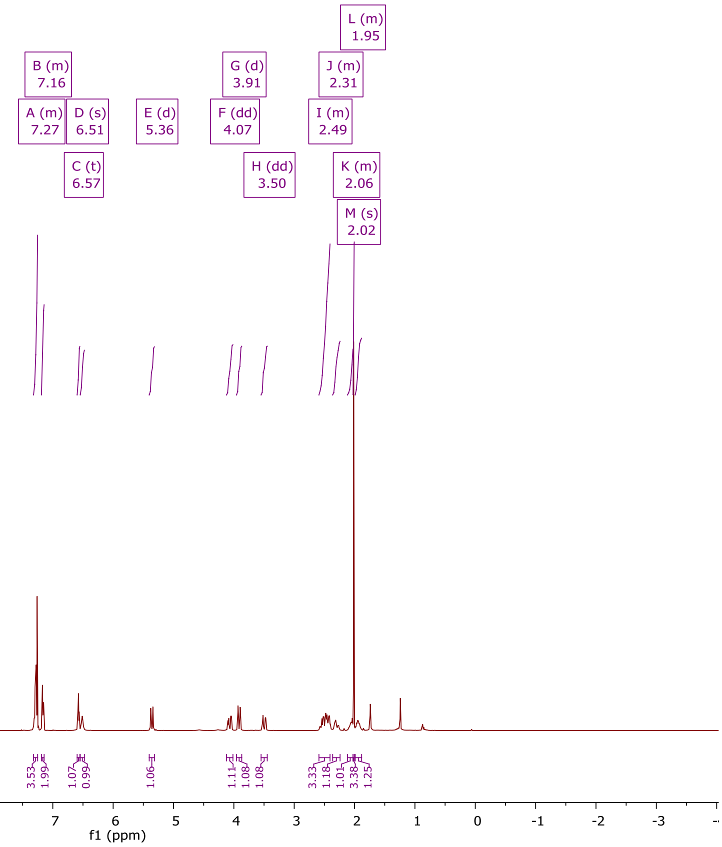

B (m) 7.16
A (m) 7.27
D (s) 6.51
C (t) 6.57
E (d) 5.36
G (d) 3.91
F (dd) 4.07
H (dd) 3.50
L (m) 1.95
J (m) 2.31
I (m) 2.49
K (m) 2.06
M (s) 2.02
3.53
1.99
1.07
0.99
1.06
1.11
1.08
1.08
3.33
1.18
1.01
3.38
1.25
f1 (ppm)

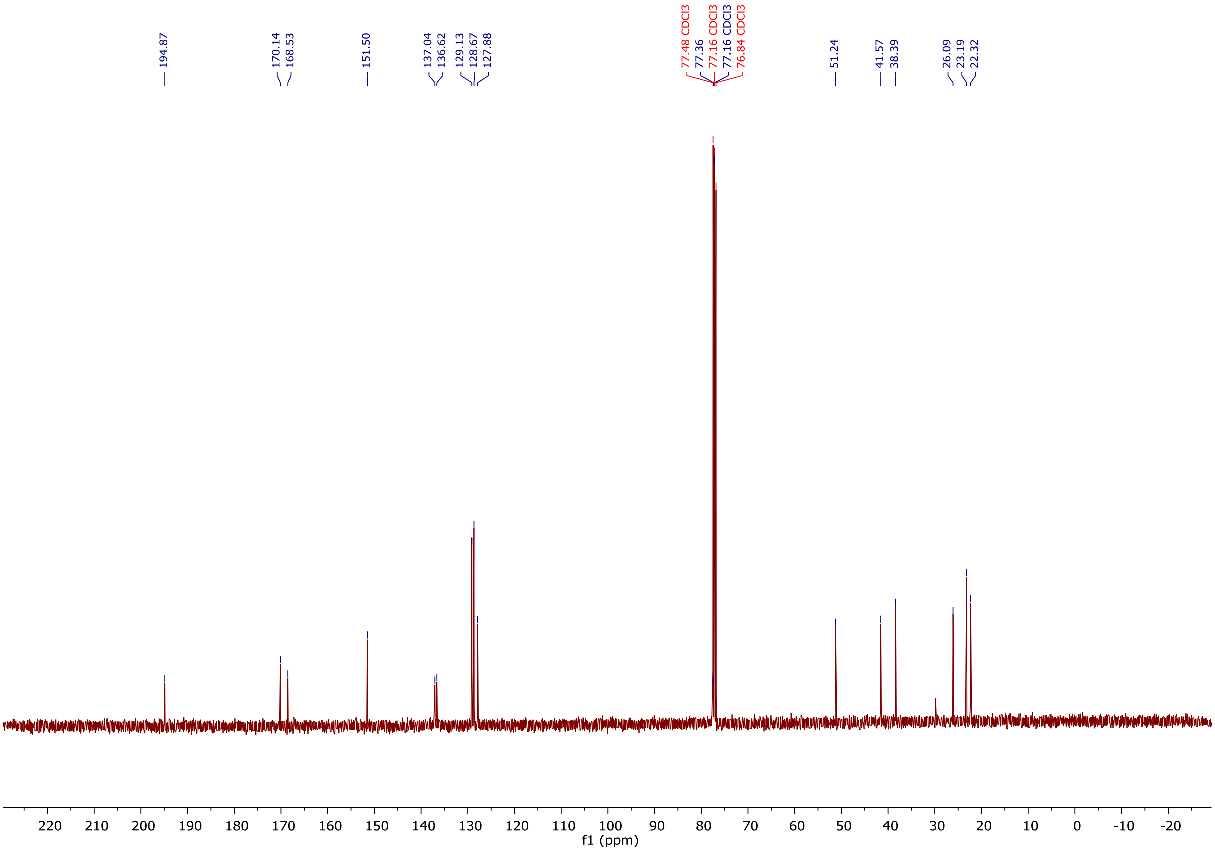

194.87
170.14
168.53
151.50
137.04
136.62
129.13
128.67
127.88
77.48 CDCl3
77.36
77.16 CDCl3
77.16 CDCl3
76.84 CDCl3
51.24
41.57
38.39
26.09
23.19
22.32
f1 (ppm)

Compound 1t

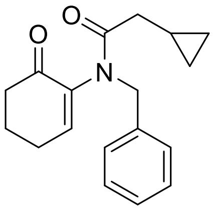

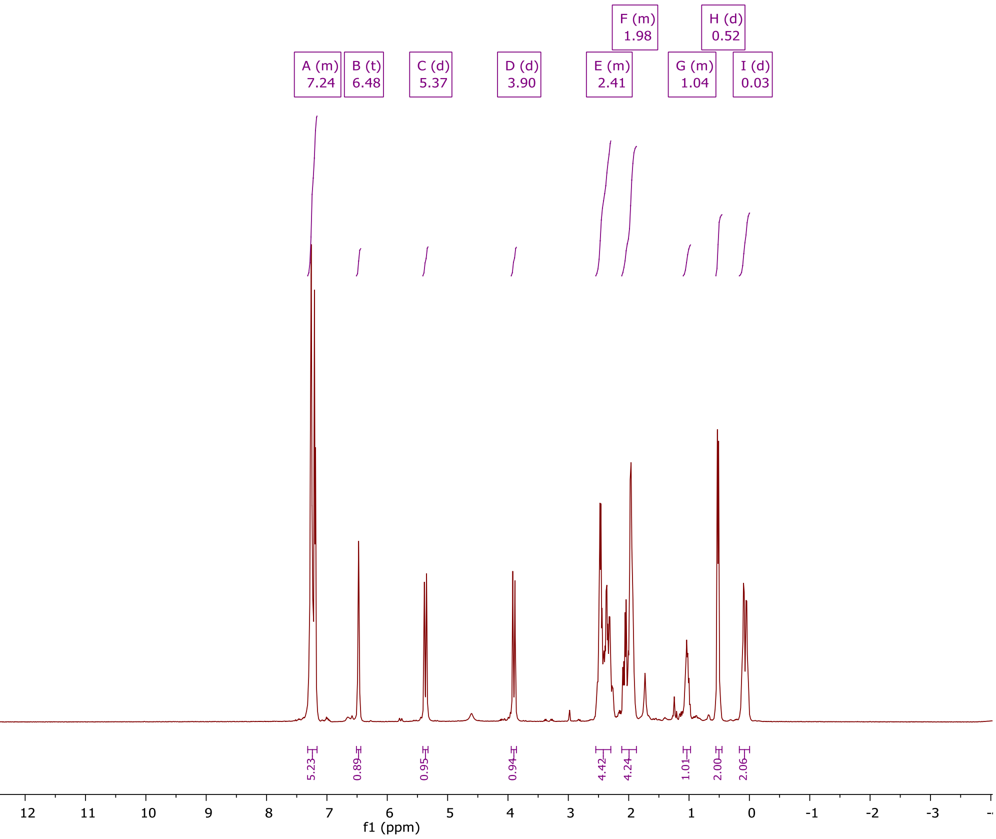

A (m) 7.24
B (t) 6.48
C (d) 5.37
D (d) 3.90
E (m) 2.41
F (m) 1.98
G (m) 1.04
H (d) 0.52
I (d) 0.03
5.23
0.89
0.95
0.94
4.42
4.24
1.01
2.00
2.06
f1 (ppm)

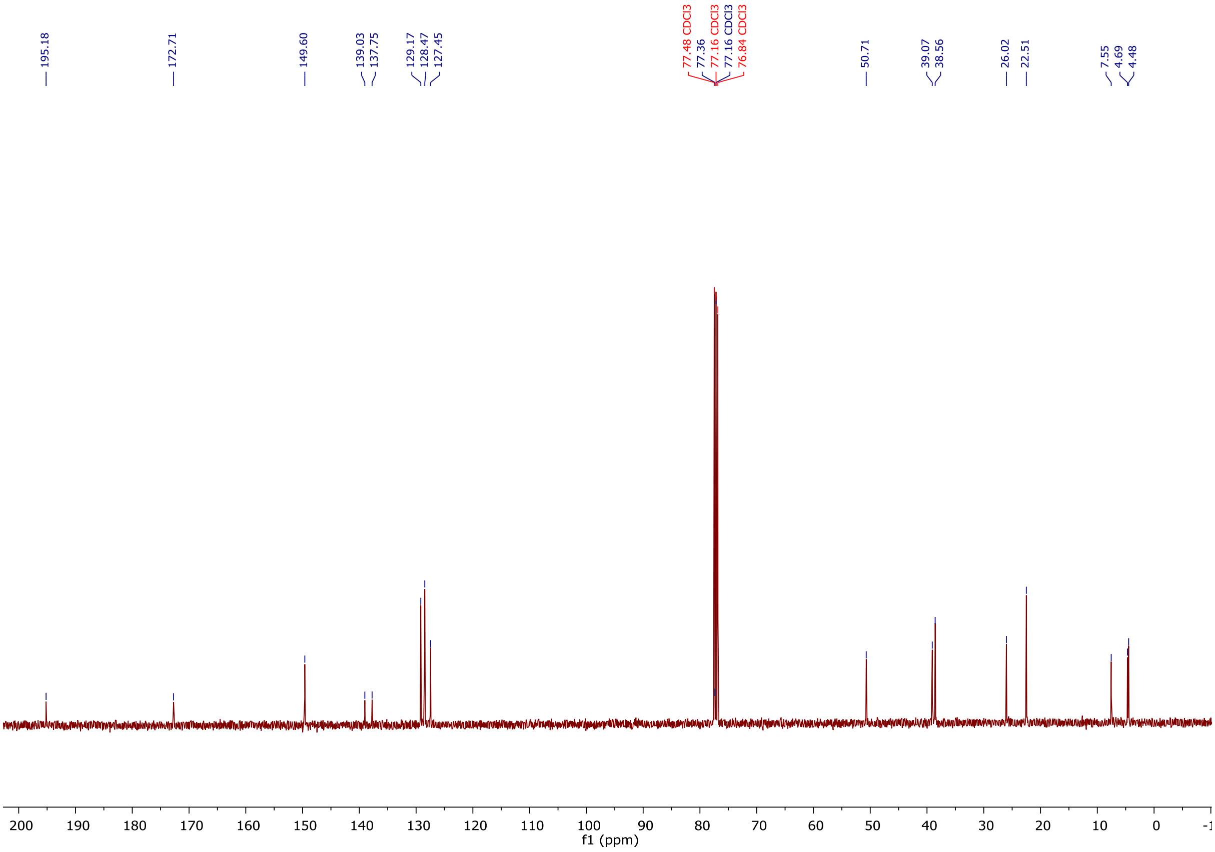

195.18
172.71
149.60
139.03
137.75
129.17
128.47
127.45
77.48 CDCl3
77.36
77.16 CDCl3
77.16 CDCl3
76.84 CDCl3
50.71
39.07
38.56
26.02
22.51
7.55
4.69
4.48
f1 (ppm)

Compound 1u

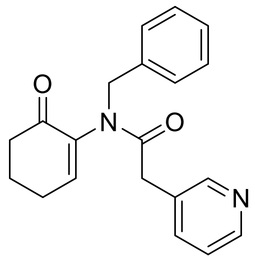

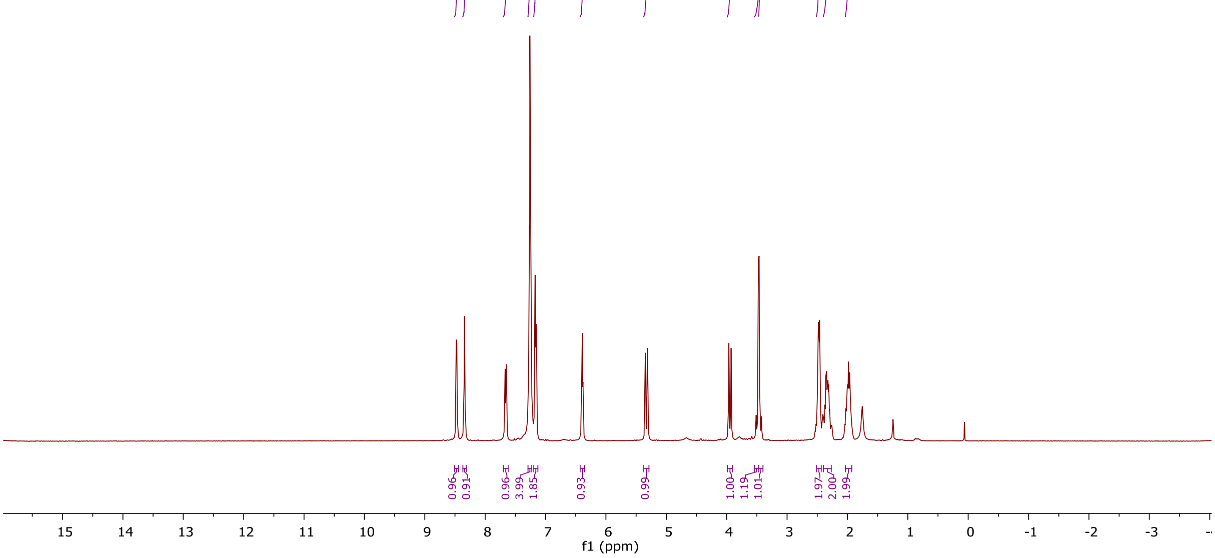

B (s) 8.34
D (m) 7.26
M (d) 3.49
K (m) 2.33
A (m) 8.47
C (m) 7.66
F (t) 6.39
G (d) 5.33
H (d) 3.95
J (m) 2.47
E (m) 7.17
N (d) 3.44
L (m) 1.98
0.96
0.91
0.96
3.99
1.85
0.93
0.99
1.00
1.19
1.01
1.97
2.00
1.99
f1 (ppm)

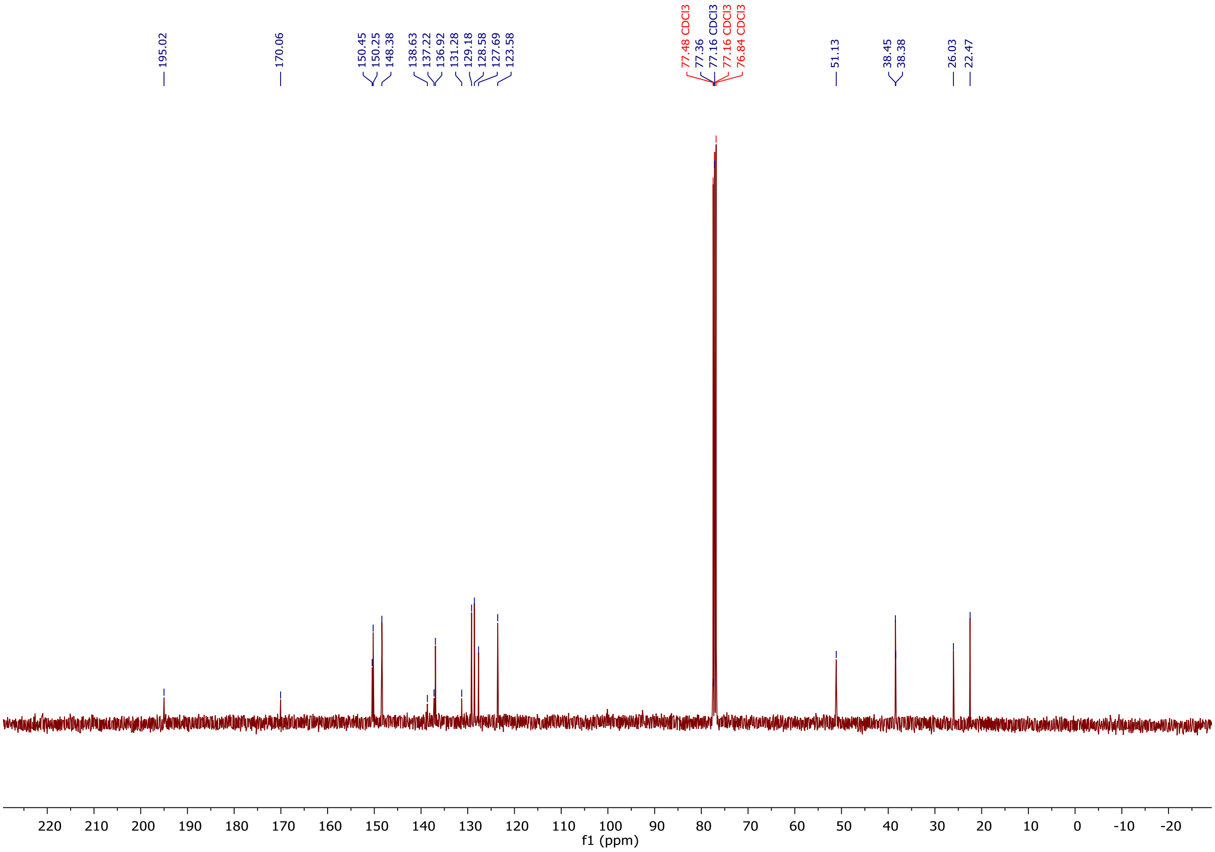

195.02
170.06
150.45
150.25
148.38
138.63
137.22
136.92
131.28
129.18
128.58
127.69
123.58
77.48 CDCl3
77.36
77.16 CDCl3
77.16 CDCl3
76.84 CDCl3
51.13
38.45
38.38
26.03
22.47
f1 (ppm)

Compound 2a

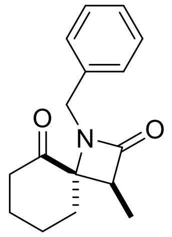

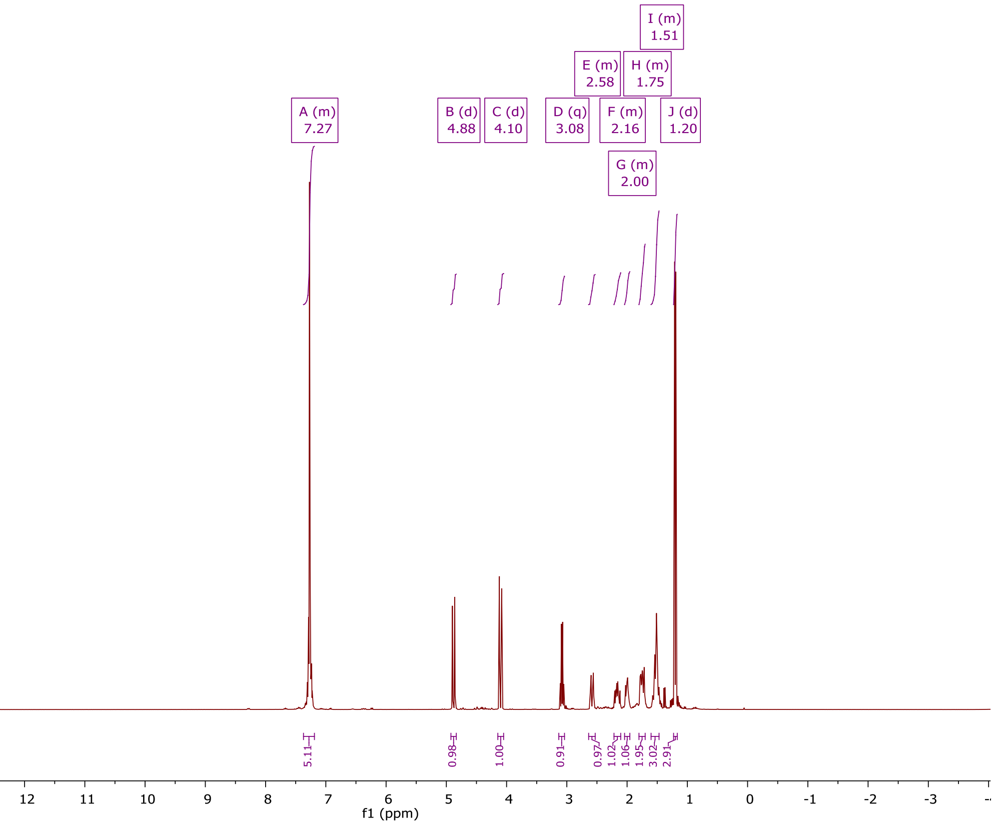
A (m) 7.27
B (d) 4.88
C (d) 4.10
D (q) 3.08
E (m) 2.58
F (m) 2.16
G (m) 2.00
H (m) 1.75
I (m) 1.51
J (d) 1.20
5.11
0.98
1.00
0.91
0.97
1.02
1.06
1.95
3.02
2.91
f1 (ppm)

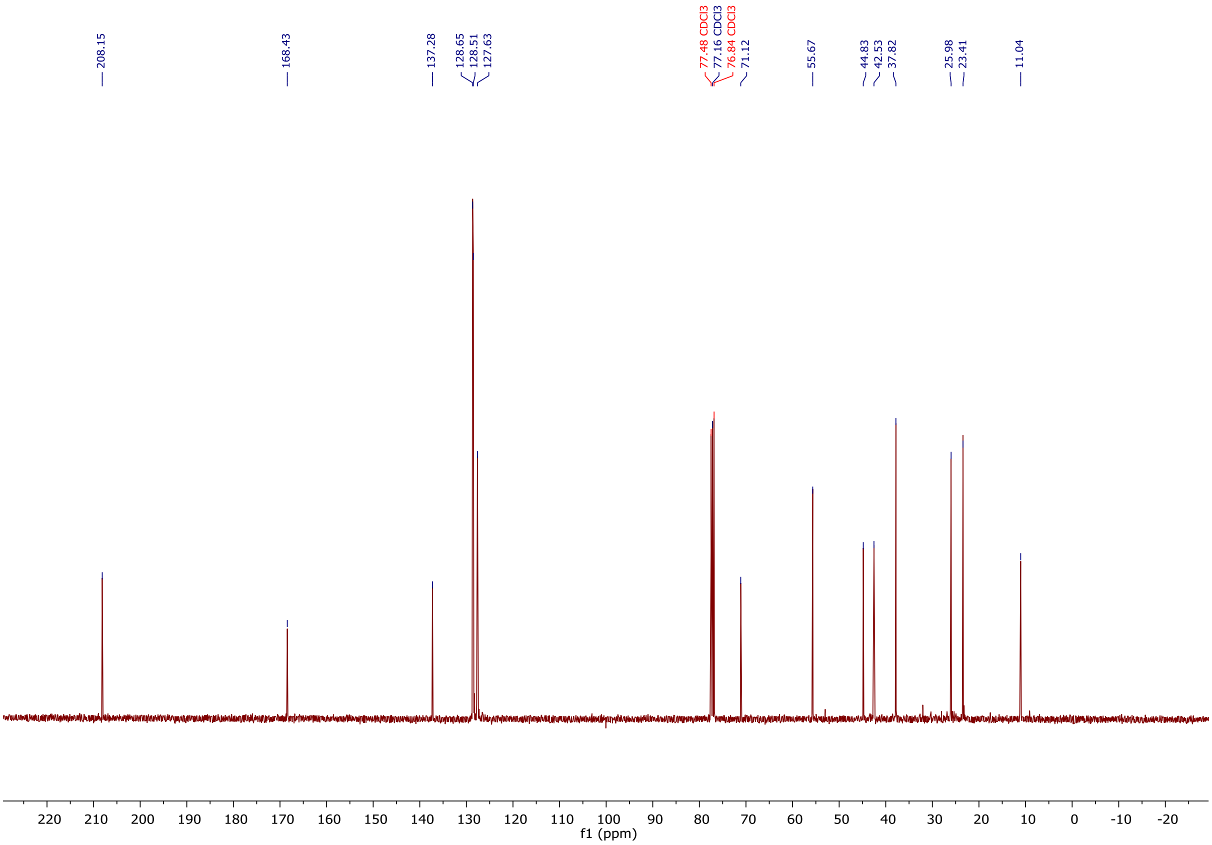
208.15
168.43
137.28
128.65
128.51
127.63
77.48 CDCl3
77.16 CDCl3
76.84 CDCl3
71.12
55.67
44.83
42.53
37.82
25.98
23.41
11.04
f1 (ppm)

Compound 2b

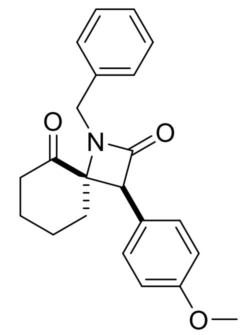

O
N
O
O

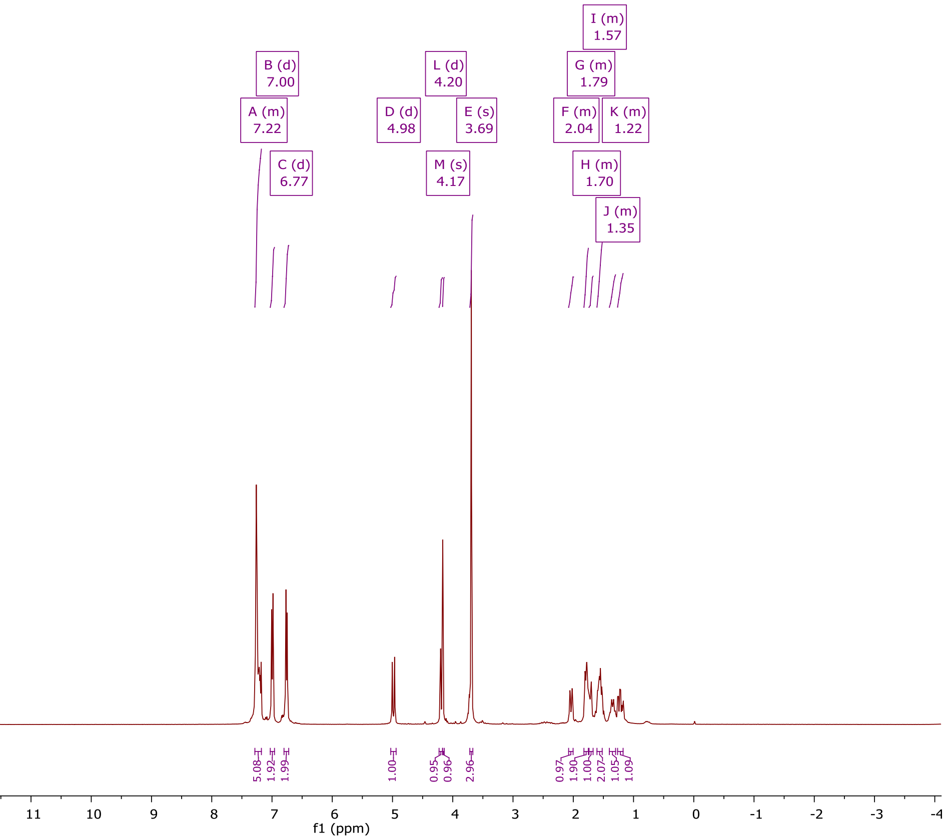

I (m) 1.57
B (d) 7.00
L (d) 4.20
G (m) 1.79
A (m) 7.22
D (d) 4.98
E (s) 3.69
F (m) 2.04
K (m) 1.22
C (d) 6.77
M (s) 4.17
H (m) 1.70
J (m) 1.35
5.08
1.92
1.99
1.00
0.95
0.96
2.96
0.97
1.90
1.00
2.07
1.05
1.09
15 14 13 12 11 10 9 8 7 6 5 4 3 2 1 0 -1 -2 -3 -4
f1 (ppm)

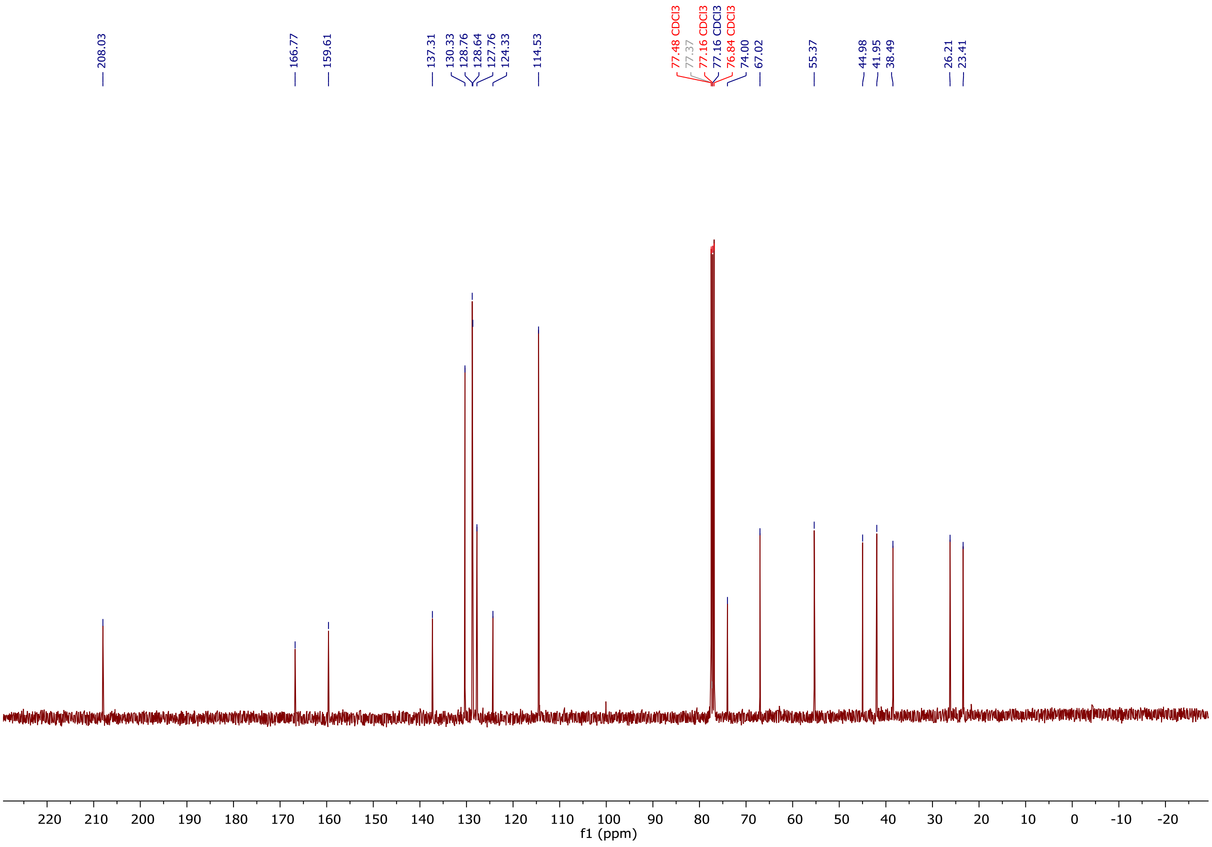

208.03
166.77
159.61
137.31
130.33
128.76
128.64
127.76
124.33
114.53
77.48 CDCl3
77.37
77.16 CDCl3
77.16 CDCl3
76.84 CDCl3
74.00
67.02
55.37
44.98
41.95
38.49
26.21
23.41
220 210 200 190 180 170 160 150 140 130 120 110 100 90 80 70 60 50 40 30 20 10 0 -10 -20
f1 (ppm)

Compound 2c

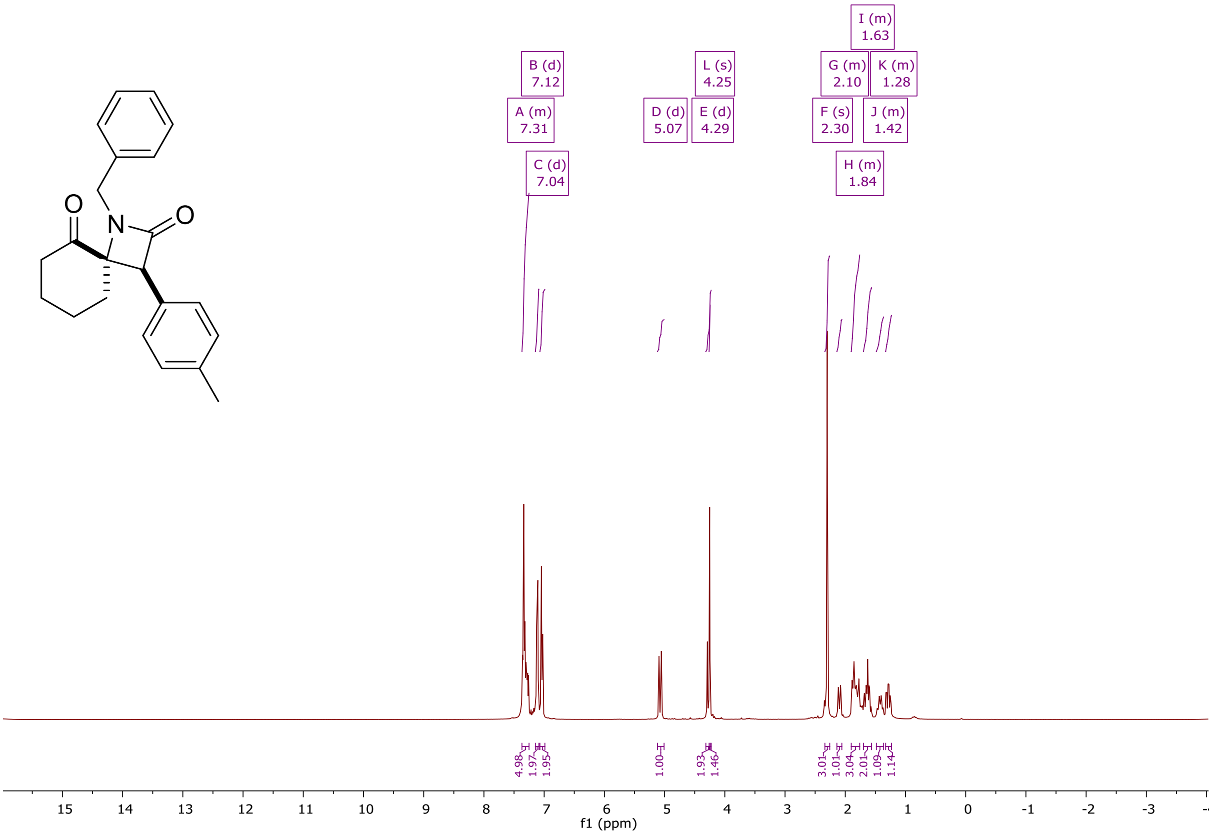

O
N
O
I (m) 1.63
B (d) 7.12
L (s) 4.25
G (m) 2.10
K (m) 1.28
A (m) 7.31
D (d) 5.07
E (d) 4.29
F (s) 2.30
J (m) 1.42
C (d) 7.04
H (m) 1.84
4.98
1.97
1.95
1.00
1.93
1.46
3.01
1.01
3.04
2.01
1.09
1.14
15
14
13
12
11
10
9
8
7
6
5
4
3
2
1
0
-1
-2
-3
f1 (ppm)

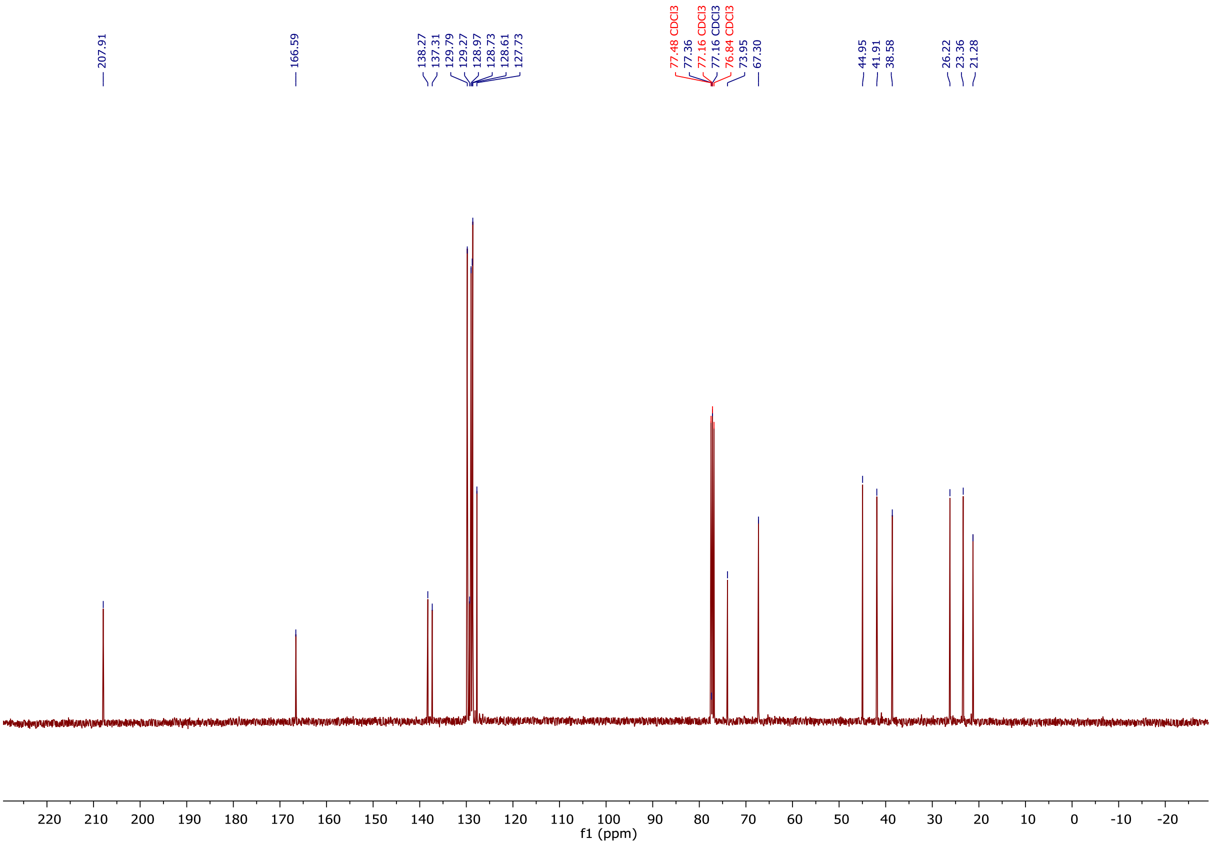

207.91
166.59
138.27
137.31
129.79
129.27
128.97
128.73
128.61
127.73
77.48 CDCl3
77.36
77.16 CDCl3
77.16 CDCl3
76.84 CDCl3
73.95
67.30
44.95
41.91
38.58
26.22
23.36
21.28
220
210
200
190
180
170
160
150
140
130
120
110
100
90
80
70
60
50
40
30
20
10
0
-10
-20
f1 (ppm)

Compound 2d

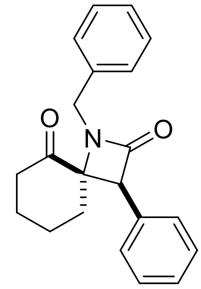

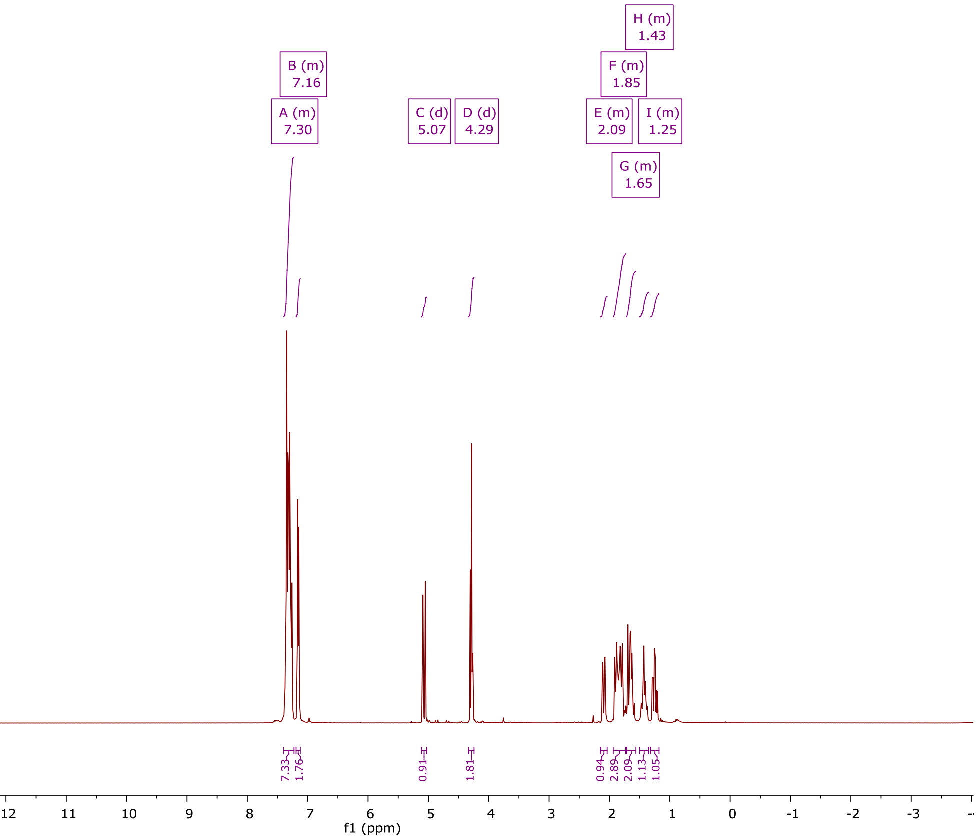

A (m) 7.30
B (m) 7.16
C (d) 5.07
D (d) 4.29
E (m) 2.09
F (m) 1.85
G (m) 1.65
H (m) 1.43
I (m) 1.25
7.33
1.76
0.91
1.81
0.94
2.89
2.09
1.13
1.05
f1 (ppm)

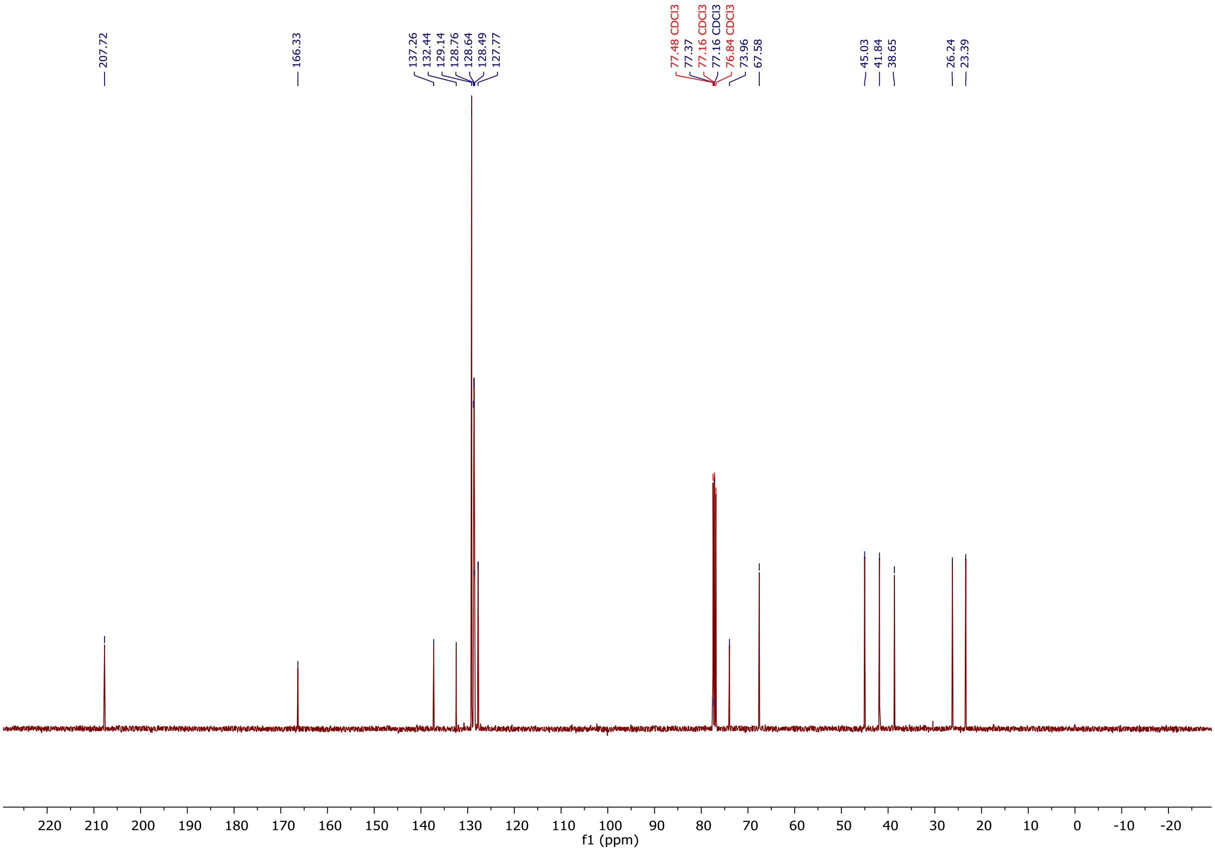

207.72
166.33
137.26
132.44
129.14
128.76
128.64
128.49
127.77
77.48 CDCl3
77.37
77.16 CDCl3
77.16 CDCl3
76.84 CDCl3
73.96
67.58
45.03
41.84
38.65
26.24
23.39
f1 (ppm)

Compound 2e

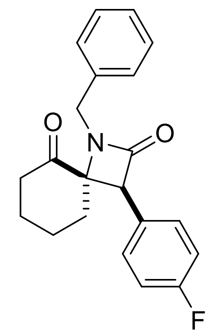
O
N
O
F

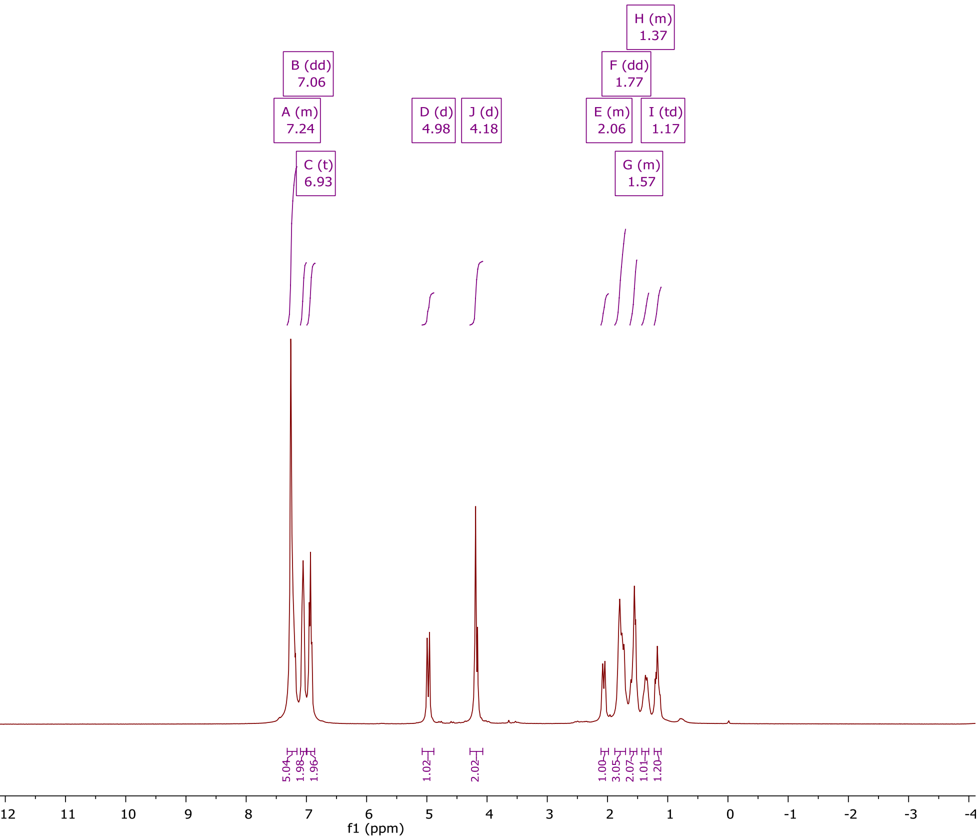
H (m) 1.37
B (dd) 7.06
F (dd) 1.77
A (m) 7.24
D (d) 4.98
J (d) 4.18
E (m) 2.06
I (td) 1.17
C (t) 6.93
G (m) 1.57
5.04
1.98
1.96
1.02
2.02
1.00
3.05
2.07
1.01
1.20
15 14 13 12 11 10 9 8 7 6 5 4 3 2 1 0 -1 -2 -3 -4
f1 (ppm)

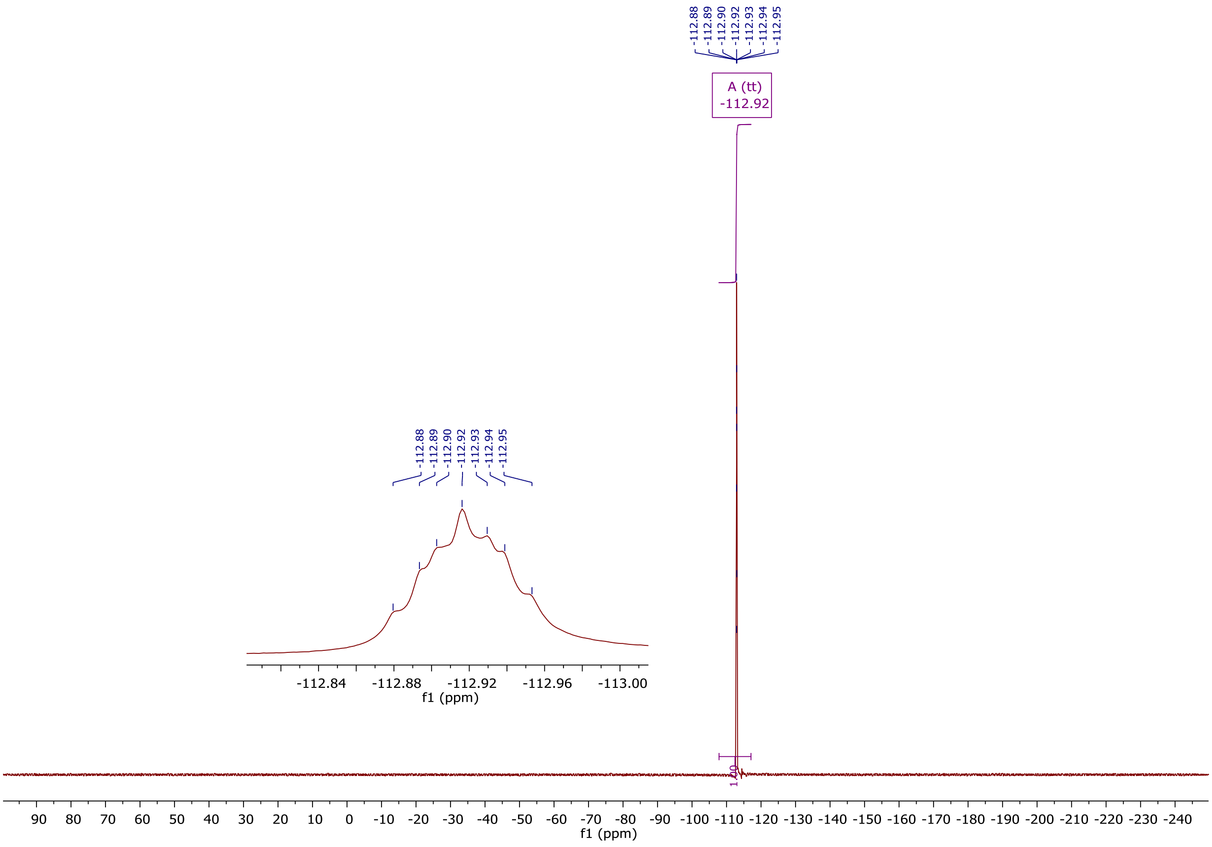
-112.88
-112.89
-112.90
-112.92
-112.93
-112.94
-112.95
A (tt)
-112.92
-112.84 -112.88 -112.92 -112.96 -113.00
f1 (ppm)
90 80 70 60 50 40 30 20 10 0 -10 -20 -30 -40 -50 -60 -70 -80 -90 -100 -110 -120 -130 -140 -150 -160 -170 -180 -190 -200 -210 -220 -230 -240
f1 (ppm)

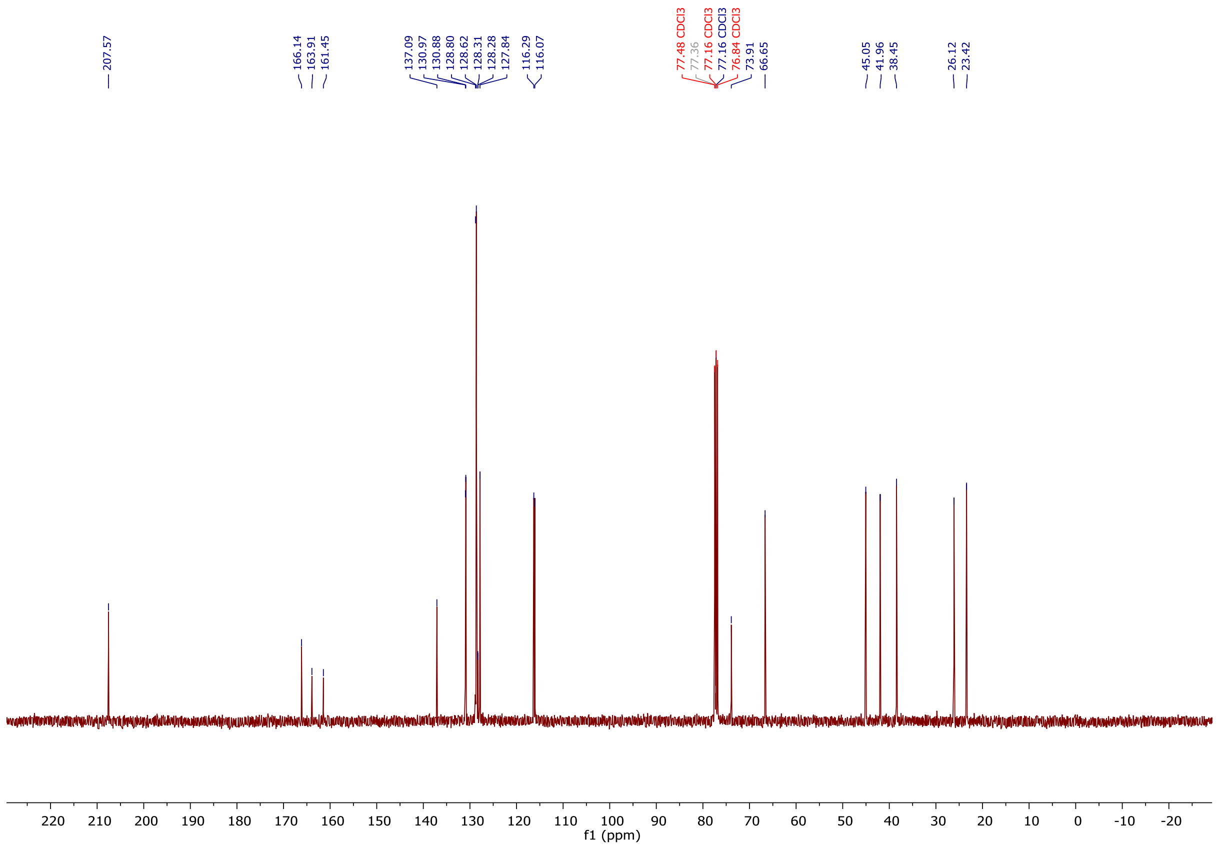
207.57
166.14
163.91
161.45
137.09
130.97
130.88
128.80
128.62
128.31
128.28
127.84
116.29
116.07
77.48 CDCl3
77.36
77.16 CDCl3
77.16 CDCl3
76.84 CDCl3
73.91
66.65
45.05
41.96
38.45
26.12
23.42
220 210 200 190 180 170 160 150 140 130 120 110 100 90 80 70 60 50 40 30 20 10 0 -10 -20
f1 (ppm)

Compound 2f

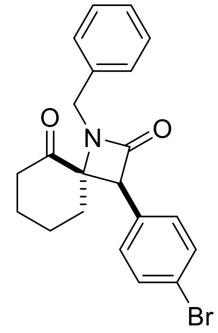
O
N
O
Br

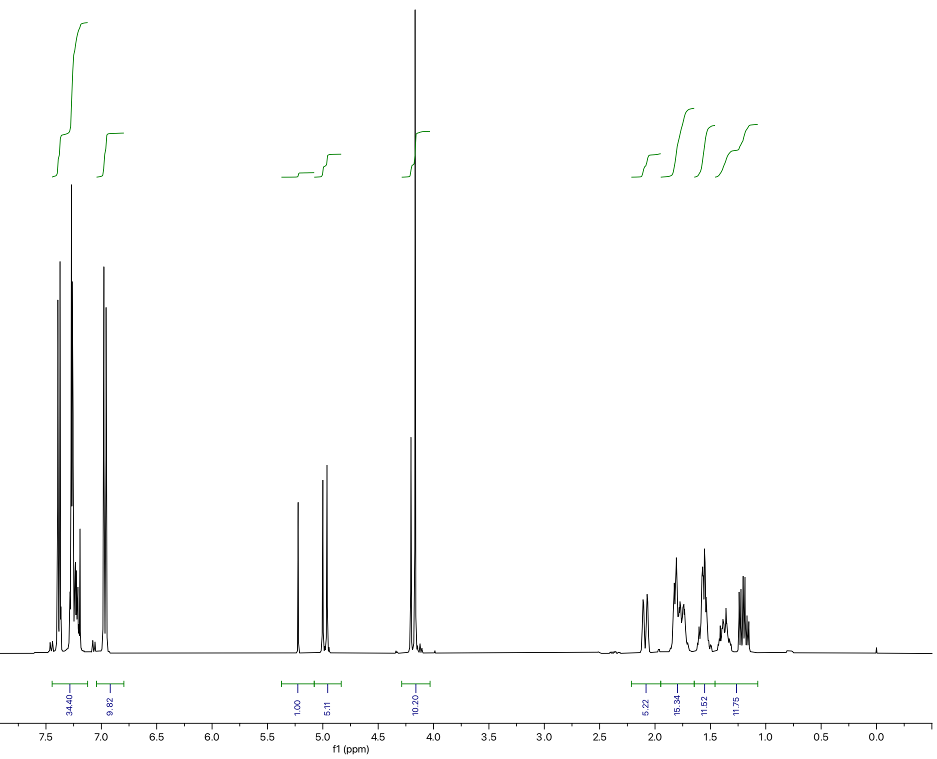
34.40
9.82
1.00
5.11
10.20
5.22
15.34
11.52
11.75
9.5
9.0
8.5
8.0
7.5
7.0
6.5
6.0
5.5
5.0
4.5
4.0
3.5
3.0
2.5
2.0
1.5
1.0
0.5
0.0
f1 (ppm)

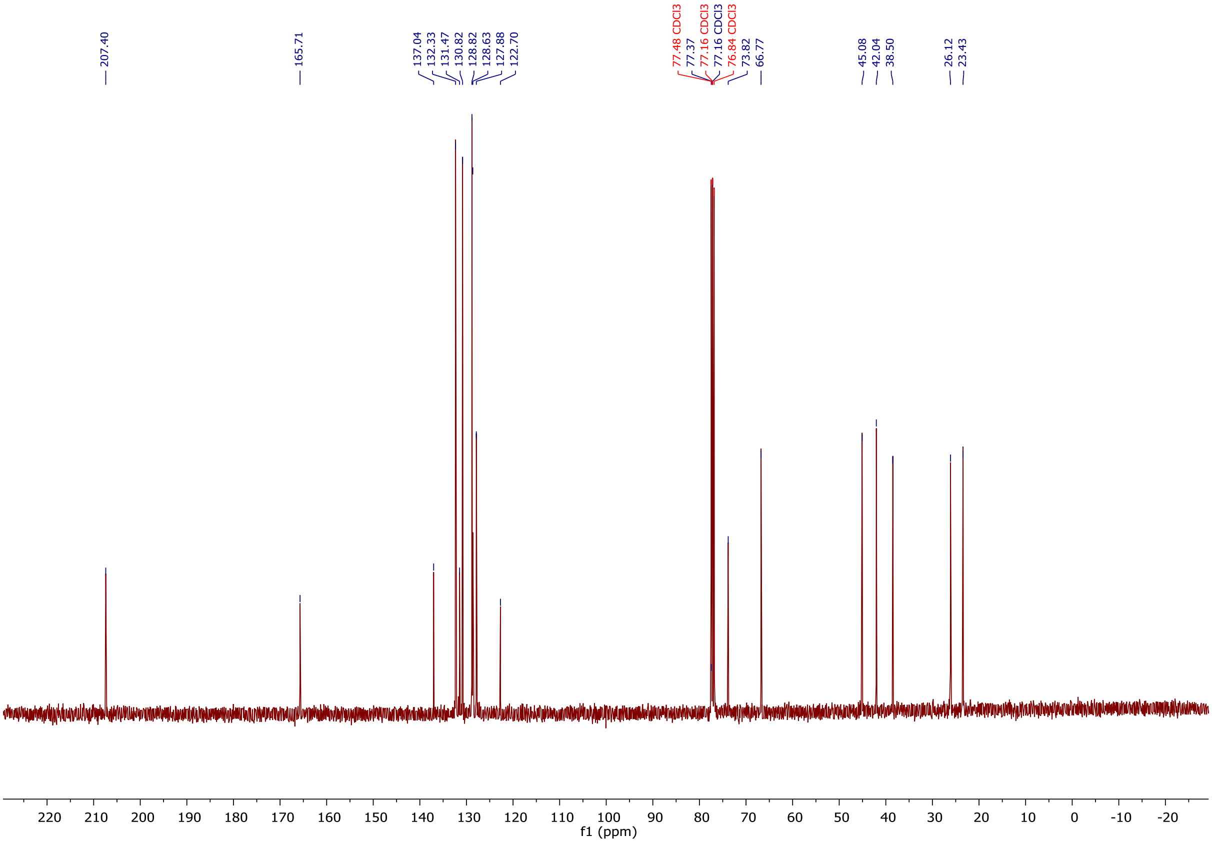
207.40
165.71
137.04
132.33
131.47
130.82
128.82
128.63
127.88
122.70
77.48 CDCl3
77.37
77.16 CDCl3
77.16 CDCl3
76.84 CDCl3
73.82
66.77
45.08
42.04
38.50
26.12
23.43
220
210
200
190
180
170
160
150
140
130
120
110
100
90
80
70
60
50
40
30
20
10
0
-10
-20
f1 (ppm)

Compound 2g

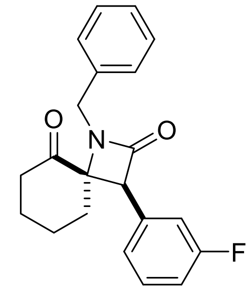
O
N
O
F

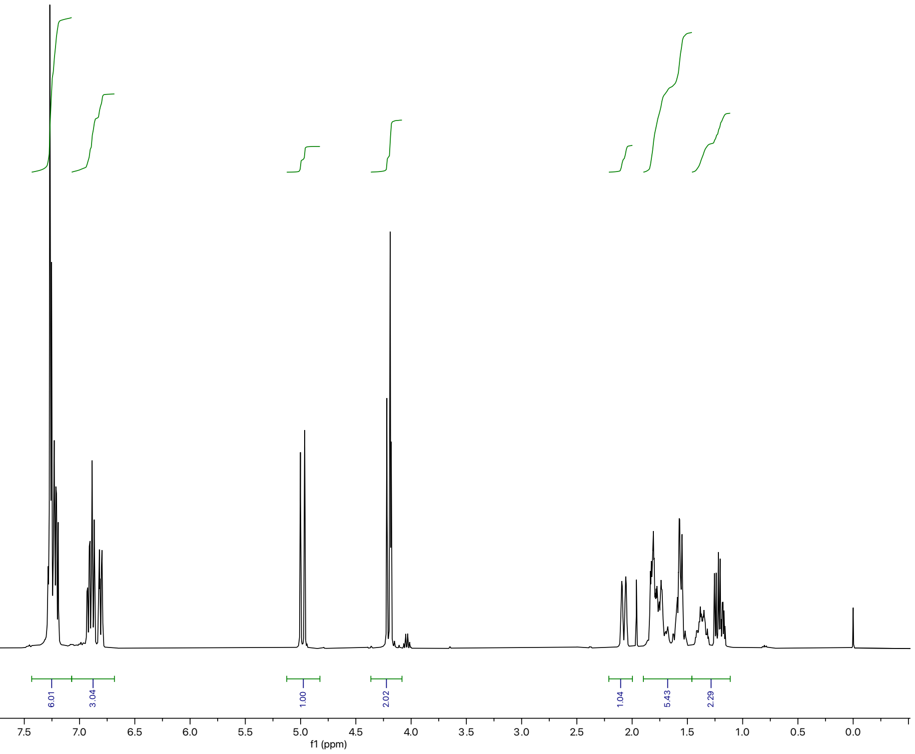
6.01
3.04
1.00
2.02
1.04
5.43
2.29
f1 (ppm)

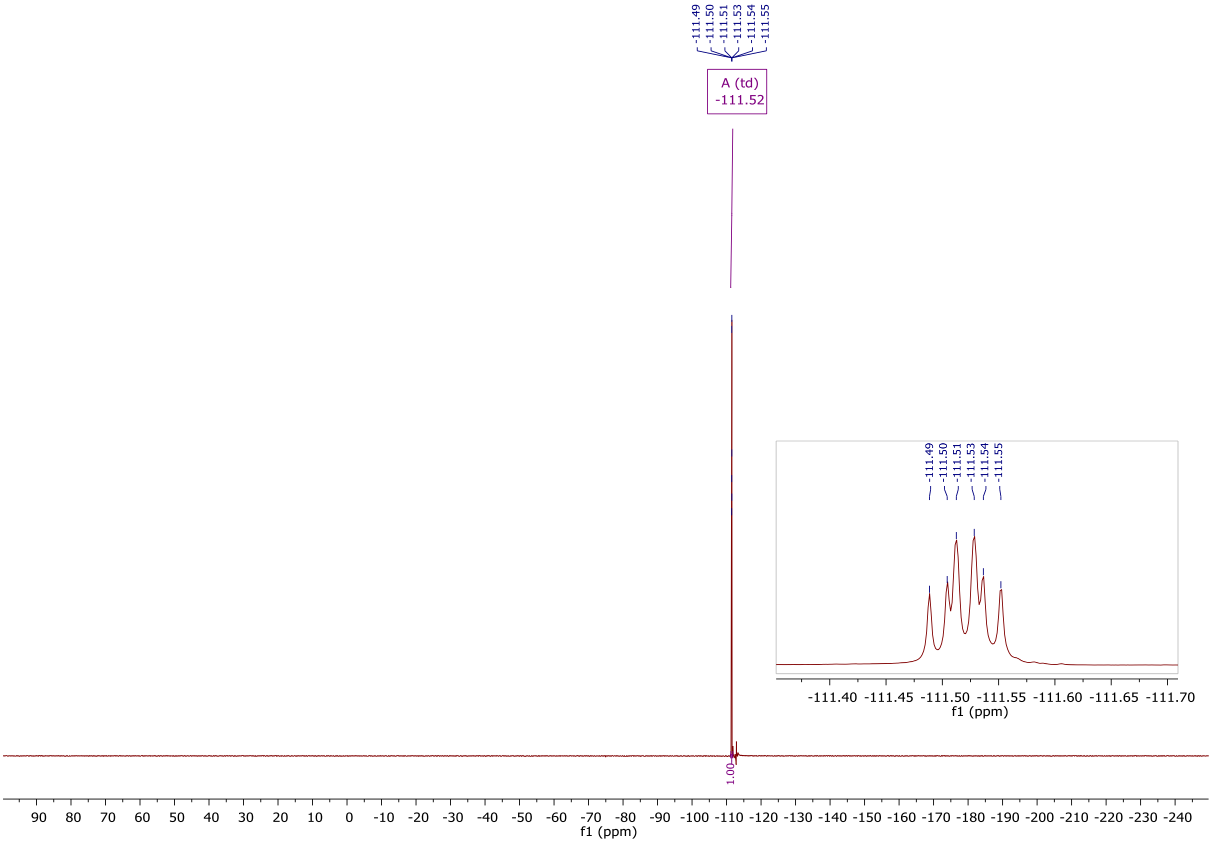
-111.49
-111.50
-111.51
-111.53
-111.54
-111.55
A (td)
-111.52
1.00
f1 (ppm)

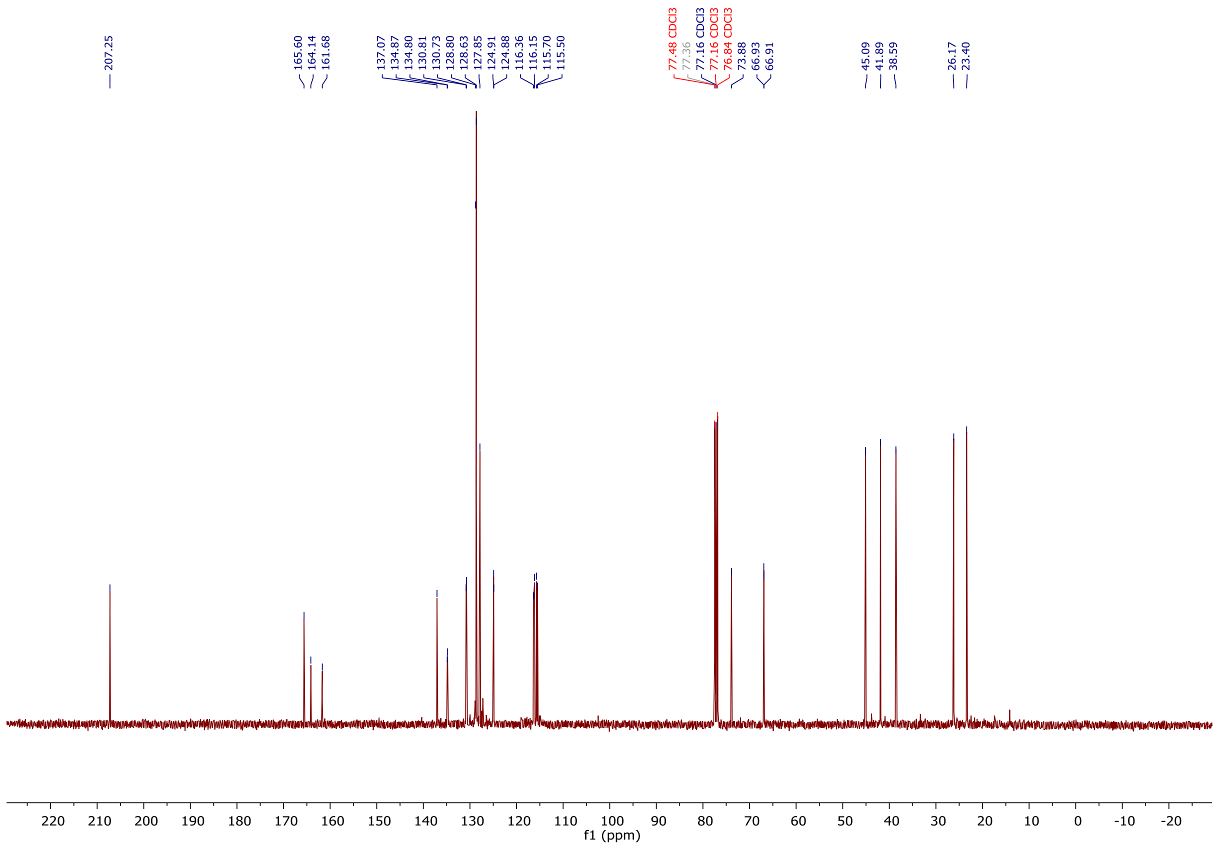

207.25
165.60
164.14
161.68
137.07
134.87
134.80
130.81
130.73
128.80
128.63
127.85
124.91
124.88
116.36
116.15
115.70
115.50
77.48 CDCl3
77.36
77.16 CDCl3
77.16 CDCl3
76.84 CDCl3
73.88
66.93
66.91
45.09
41.89
38.59
26.17
23.40
220 210 200 190 180 170 160 150 140 130 120 110 100 90 80 70 60 50 40 30 20 10 0 -10 -20
f1 (ppm)

Compound 2h

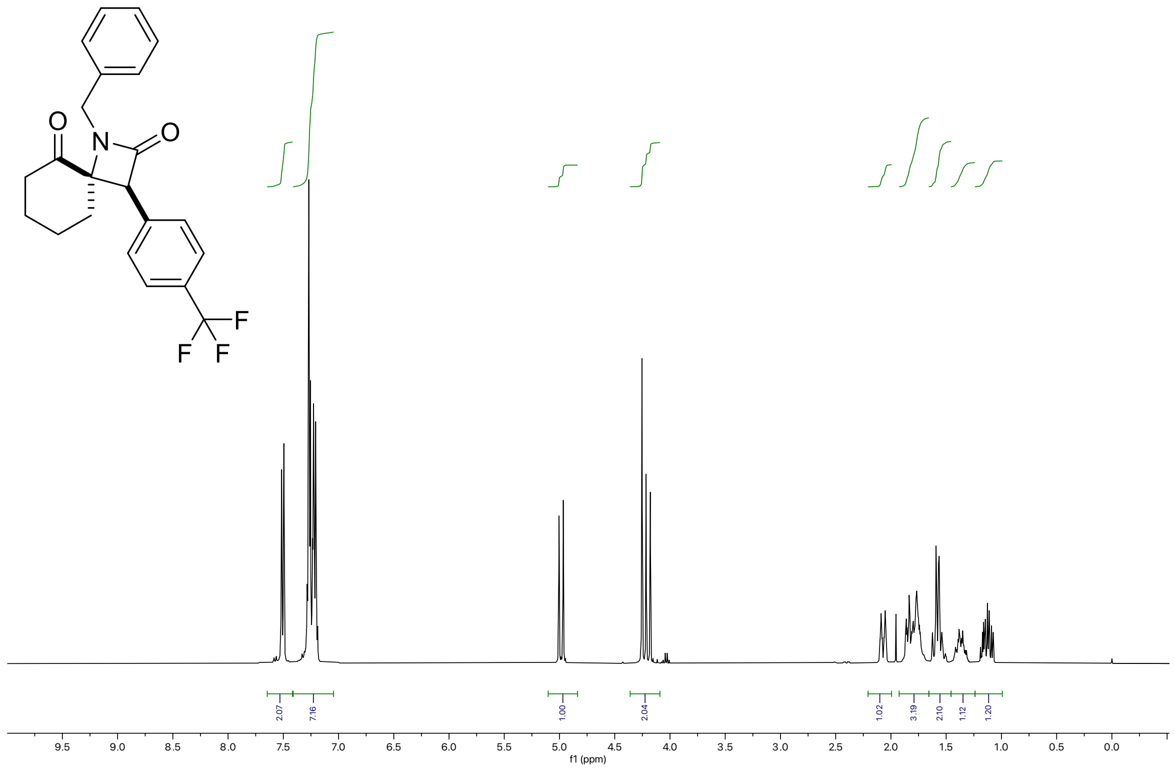

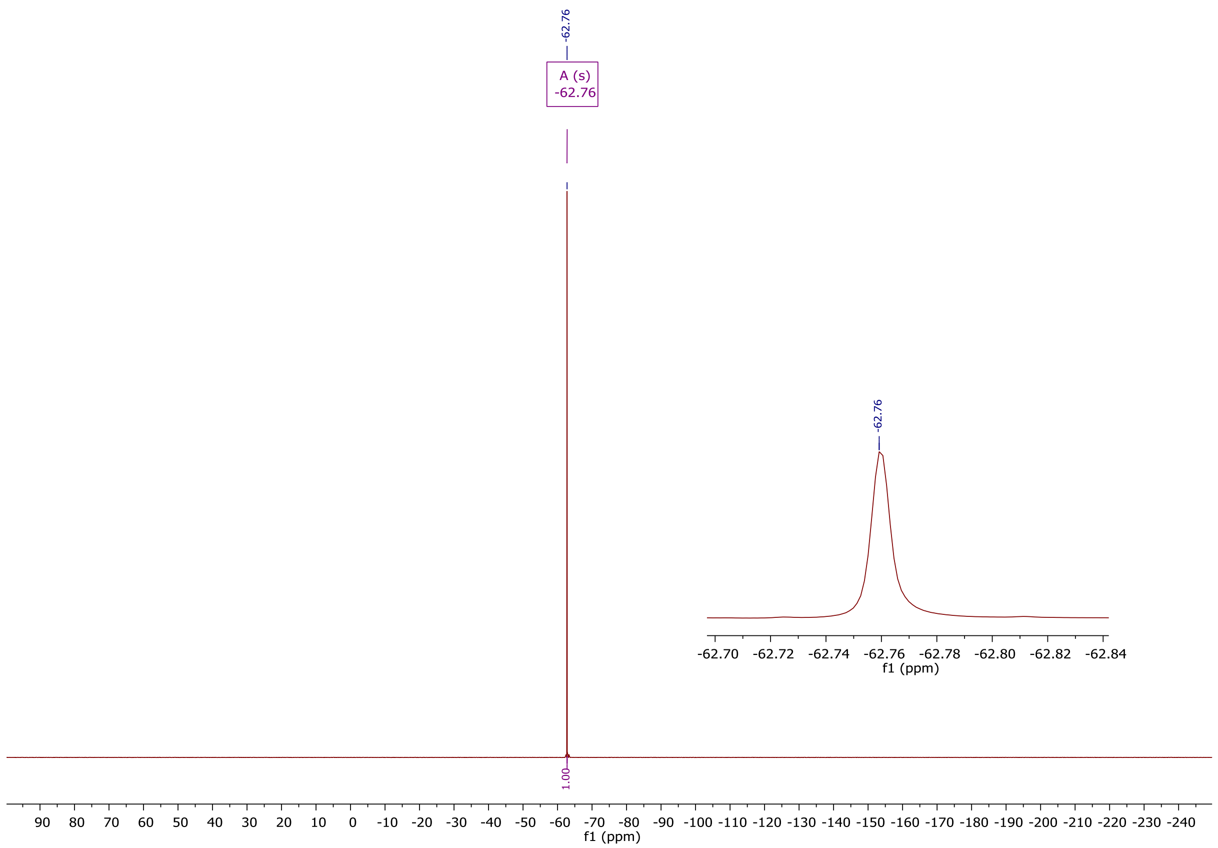

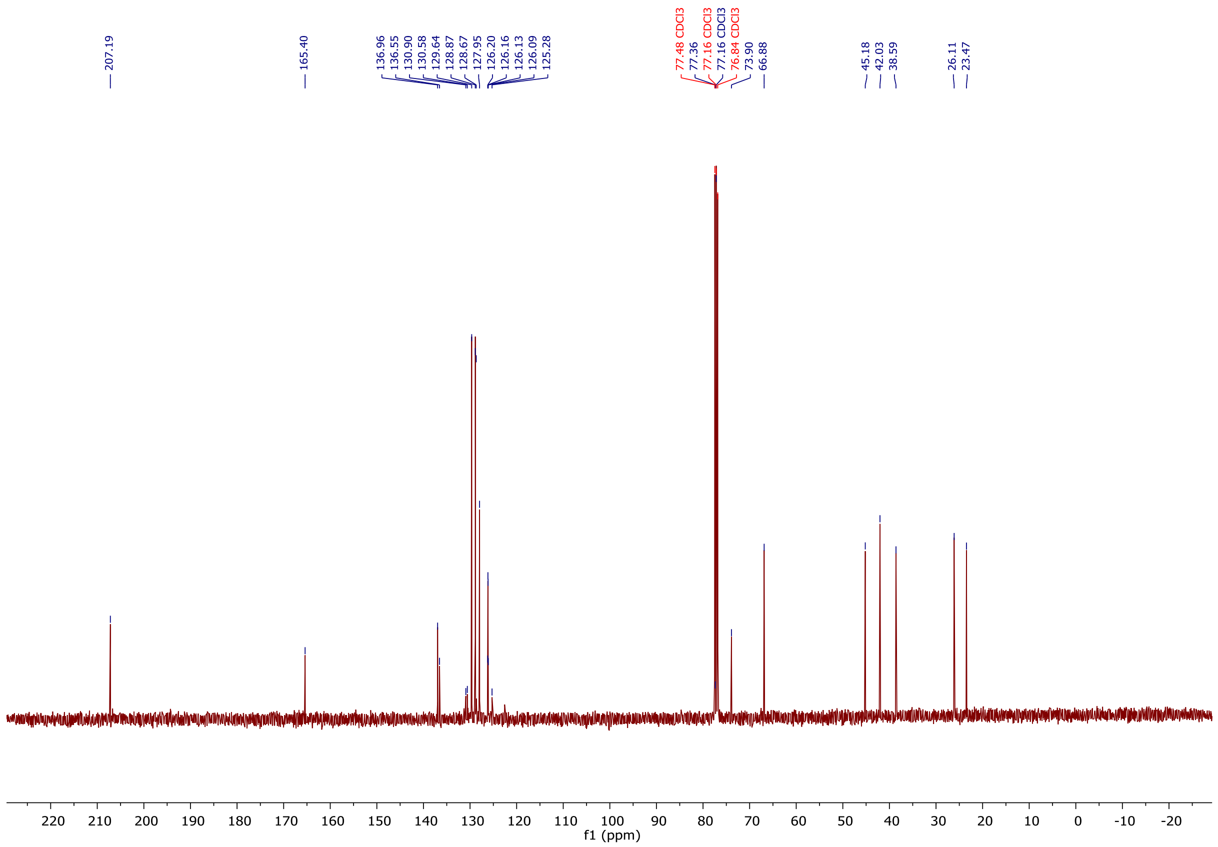
207.19
165.40
136.96
136.55
130.90
130.58
129.64
128.87
128.67
127.95
126.20
126.16
126.13
126.09
125.28
77.48 CDCl3
77.36
77.16 CDCl3
77.16 CDCl3
76.84 CDCl3
73.90
66.88
45.18
42.03
38.59
26.11
23.47
220 210 200 190 180 170 160 150 140 130 120 110 100 90 80 70 60 50 40 30 20 10 0 -10 -20
f1 (ppm)

Compound 2i

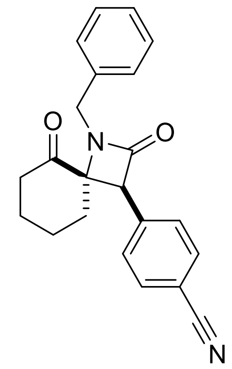

O
N
O
N

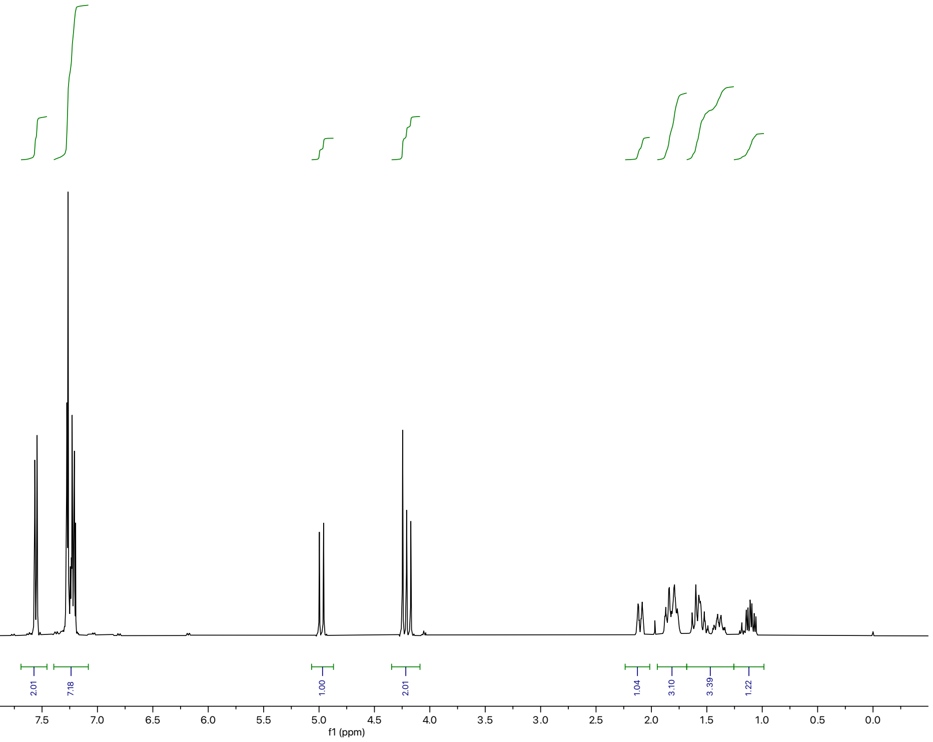

2.01
7.18
1.00
2.01
1.04
3.10
3.39
1.22
9.5 9.0 8.5 8.0 7.5 7.0 6.5 6.0 5.5 5.0 4.5 4.0 3.5 3.0 2.5 2.0 1.5 1.0 0.5 0.0
f1 (ppm)

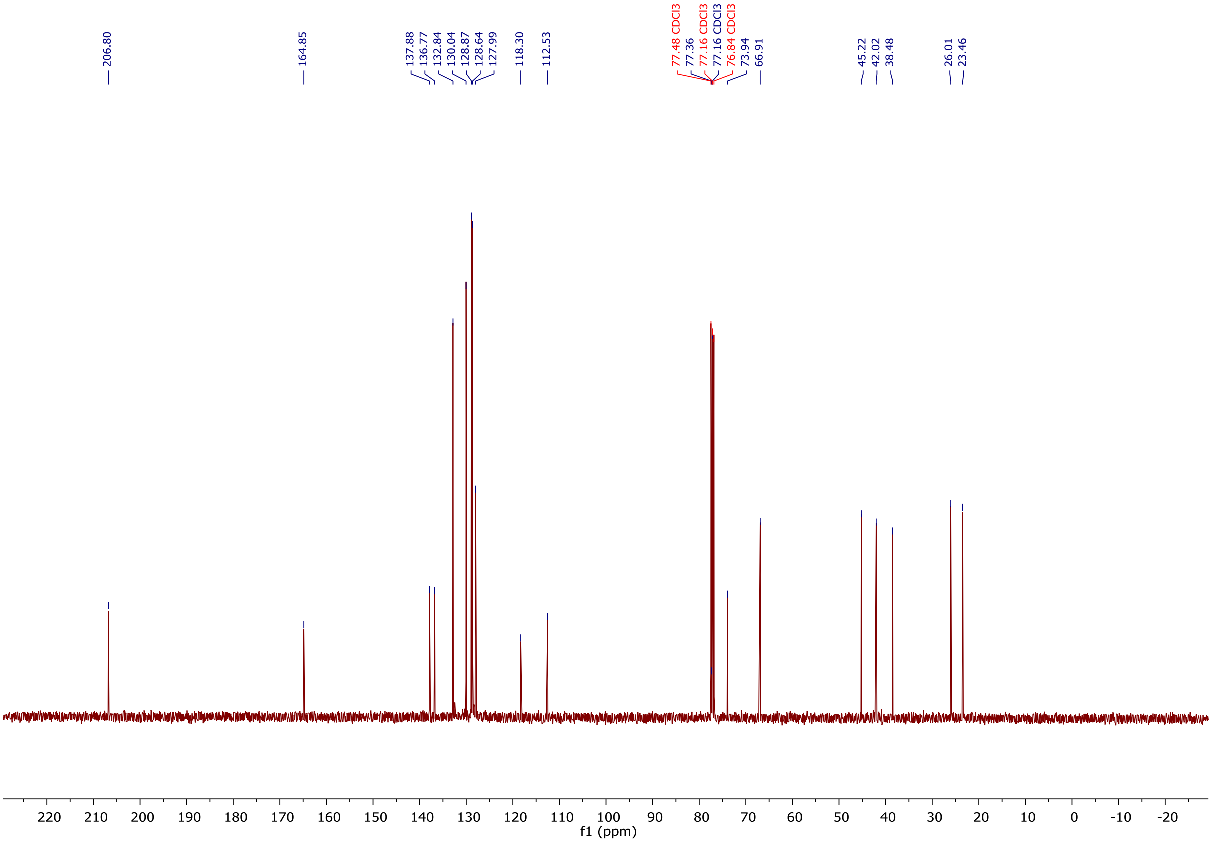

206.80
164.85
137.88
136.77
132.84
130.04
128.87
128.64
127.99
118.30
112.53
77.48 CDCl3
77.36
77.16 CDCl3
77.16 CDCl3
76.84 CDCl3
73.94
66.91
45.22
42.02
38.48
26.01
23.46
220 210 200 190 180 170 160 150 140 130 120 110 100 90 80 70 60 50 40 30 20 10 0 -10 -20
f1 (ppm)

Compound 2j

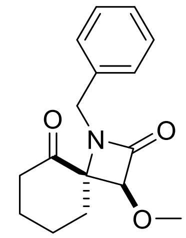

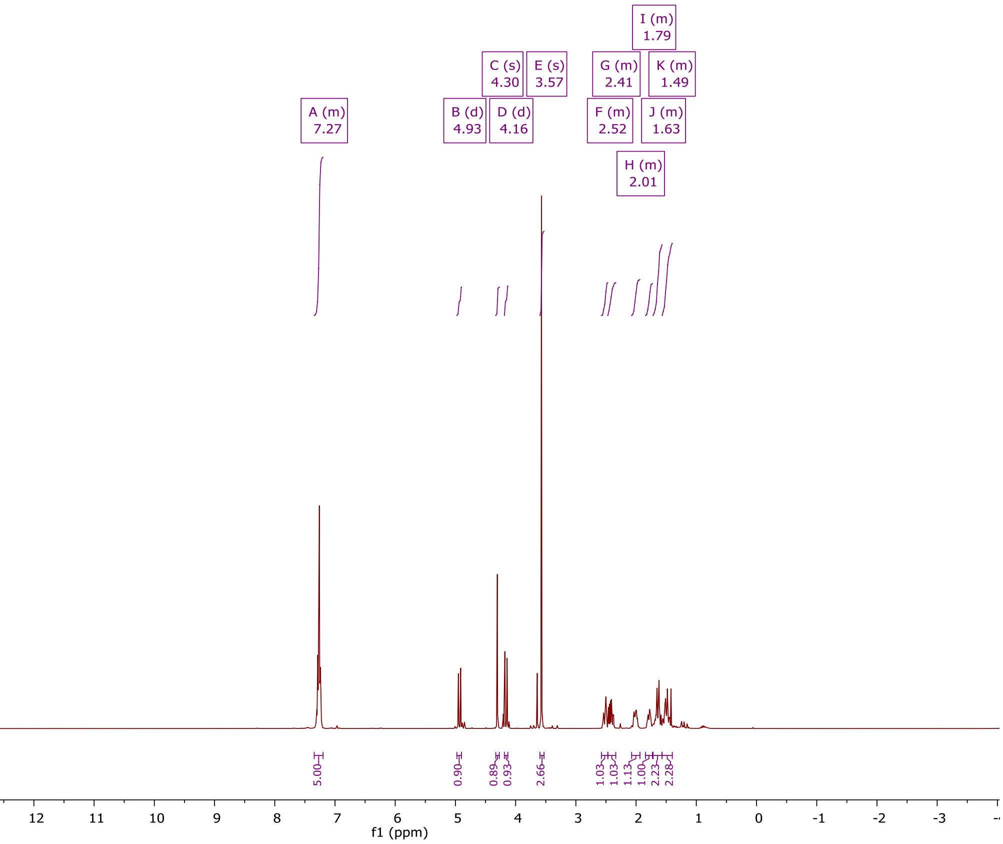
A (m) 7.27
B (d) 4.93
C (s) 4.30
D (d) 4.16
E (s) 3.57
F (m) 2.52
G (m) 2.41
H (m) 2.01
I (m) 1.79
J (m) 1.63
K (m) 1.49
5.00
0.90
0.89
0.93
2.66
1.03
1.03
1.13
1.00
2.23
2.28
f1 (ppm)

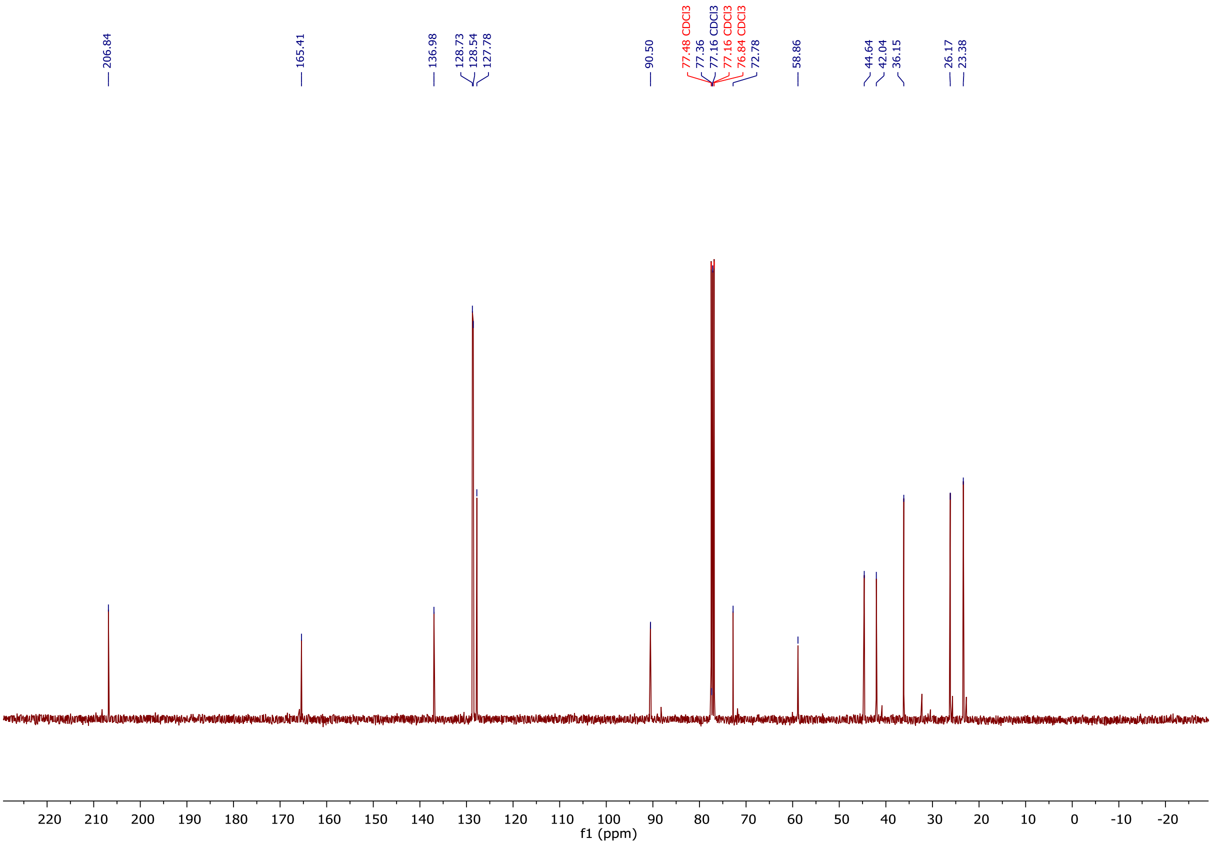
206.84
165.41
136.98
128.73
128.54
127.78
90.50
77.48 CDCl3
77.36
77.16 CDCl3
77.16 CDCl3
76.84 CDCl3
72.78
58.86
44.64
42.04
36.15
26.17
23.38
f1 (ppm)

Compound 2l

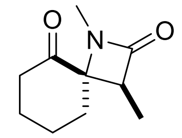

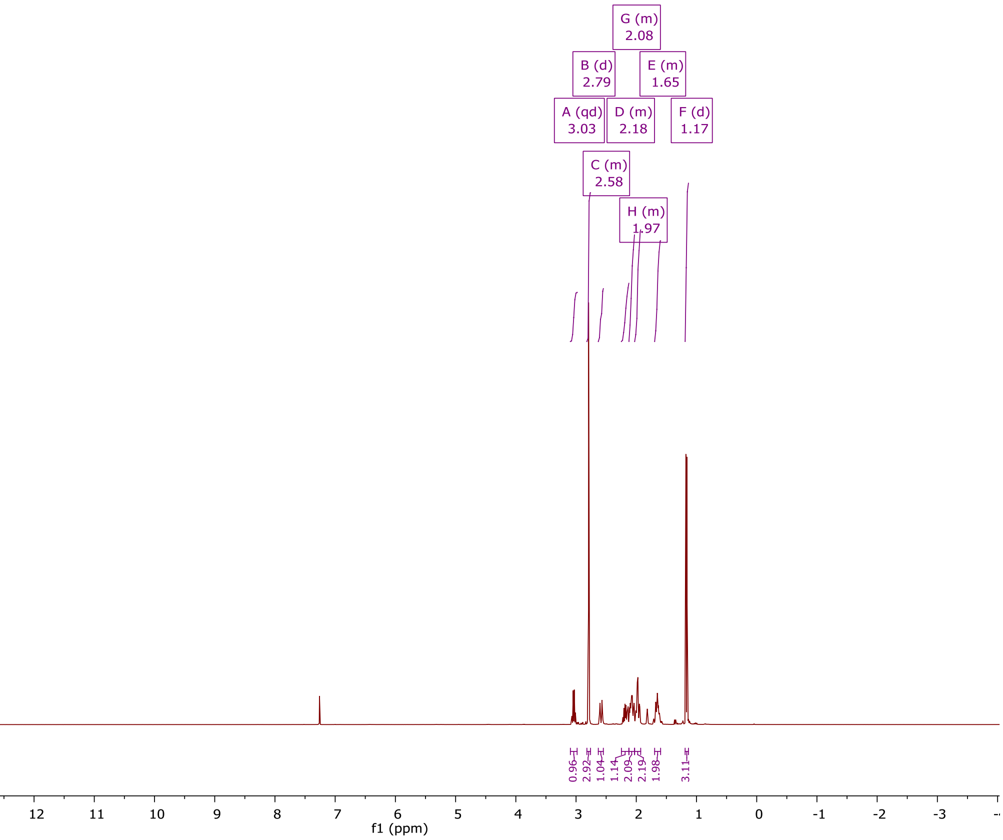
G (m)
2.08
B (d)
2.79
E (m)
1.65
A (qd)
3.03
D (m)
2.18
F (d)
1.17
C (m)
2.58
H (m)
1.97
0.96
2.92
1.04
1.14
2.09
2.19
1.98
3.11
f1 (ppm)

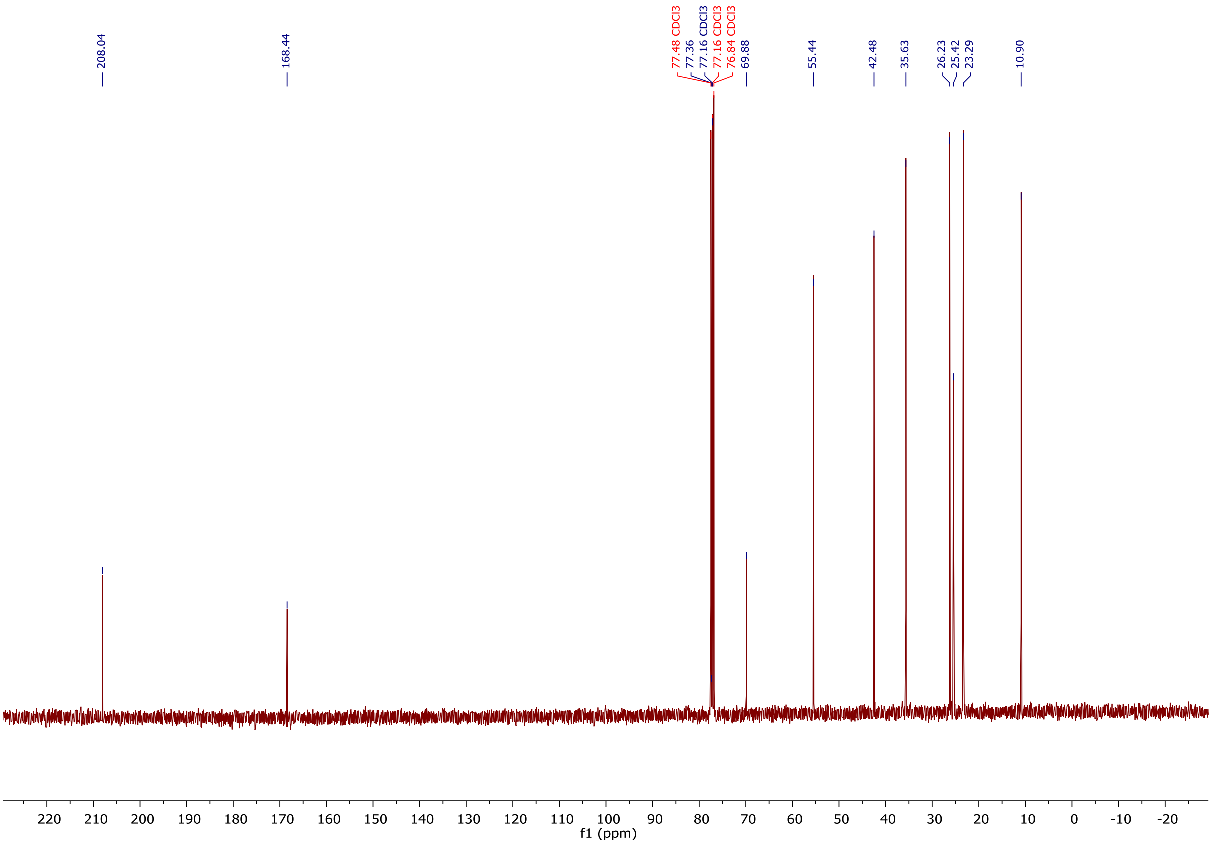
208.04
168.44
77.48 CDCl3
77.36
77.16 CDCl3
77.16 CDCl3
76.84 CDCl3
69.88
55.44
42.48
35.63
26.23
25.42
23.29
10.90
f1 (ppm)

Compound 2m

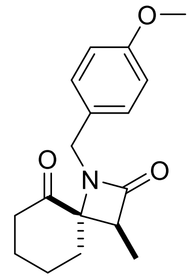

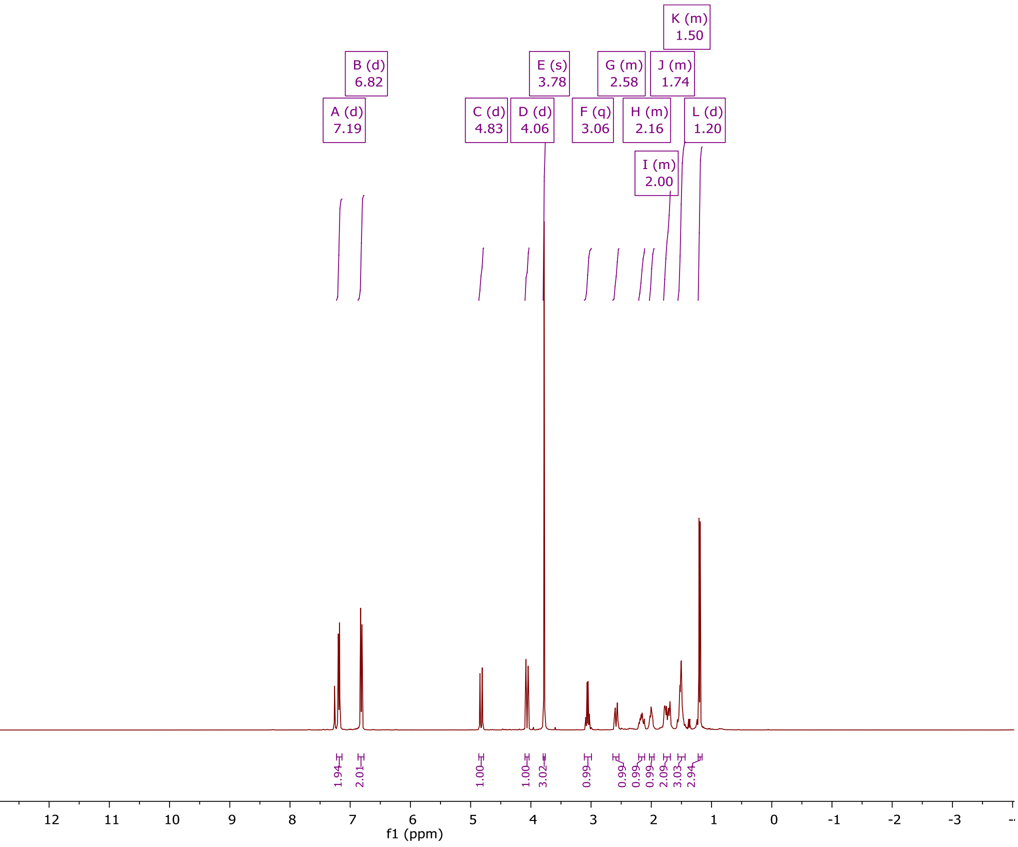

A (d) 7.19
B (d) 6.82
C (d) 4.83
D (d) 4.06
E (s) 3.78
F (q) 3.06
G (m) 2.58
H (m) 2.16
I (m) 2.00
J (m) 1.74
K (m) 1.50
L (d) 1.20
1.94
2.01
1.00
1.00
3.02
0.99
0.99
0.99
0.99
2.09
3.03
2.94
f1 (ppm)

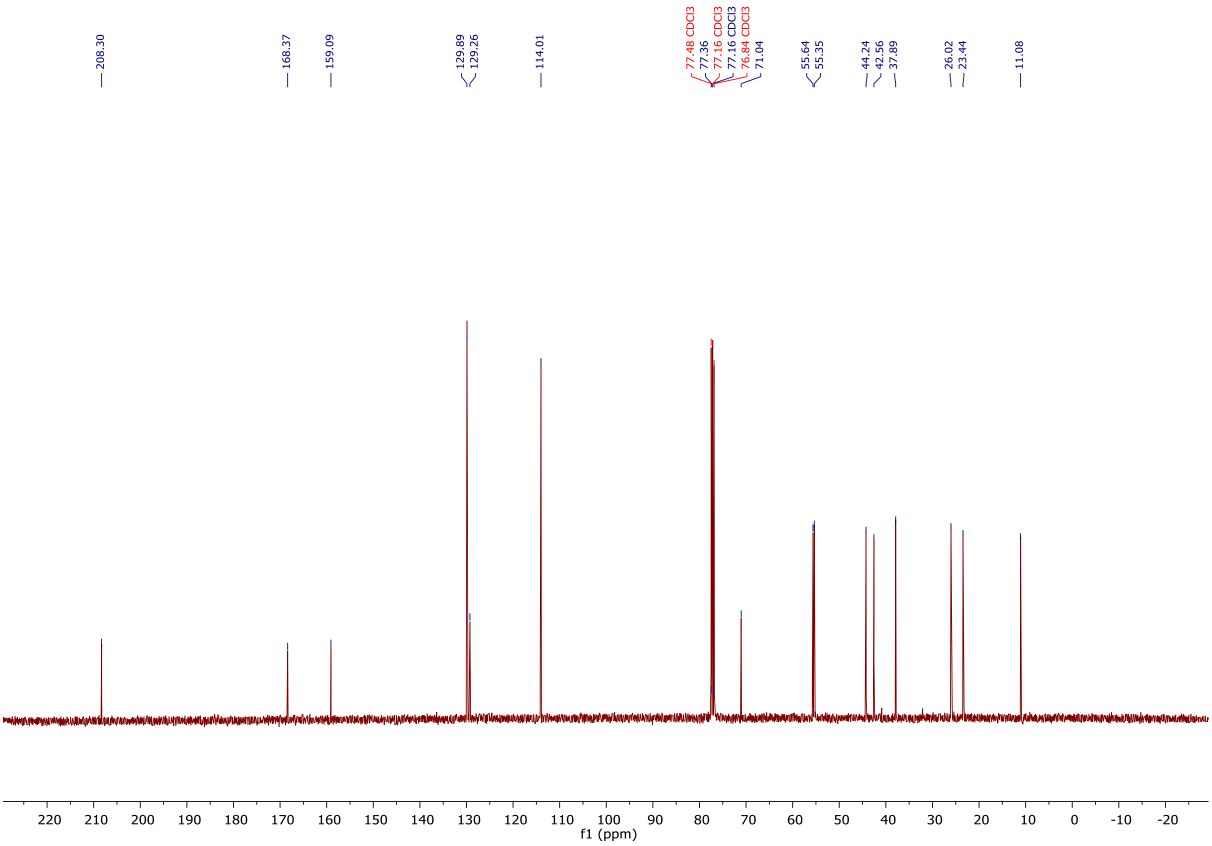

208.30
168.37
159.09
129.89
129.26
114.01
77.48 CDCl3
77.36
77.16 CDCl3
77.16 CDCl3
76.84 CDCl3
71.04
55.64
55.35
44.24
42.56
37.89
26.02
23.44
11.08
f1 (ppm)

Compound 2n

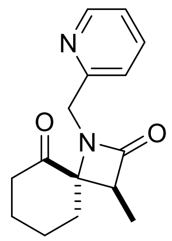
N
O
N
O

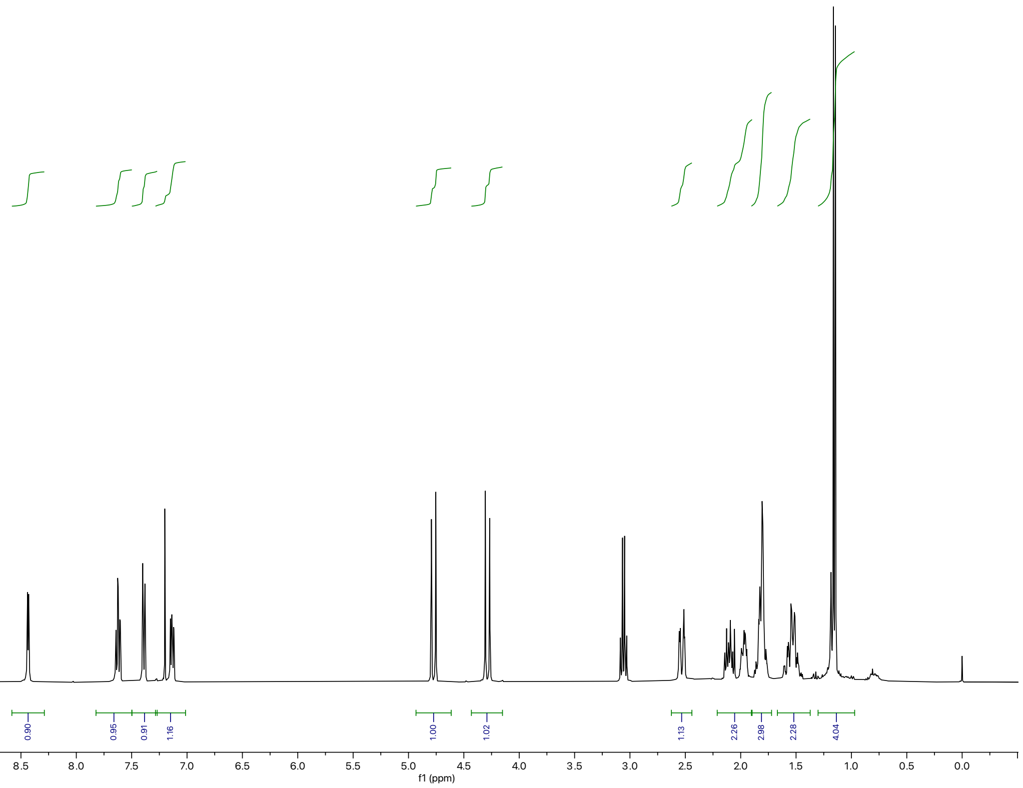
0.90
0.95
0.91
1.16
1.00
1.02
1.13
2.26
2.98
2.28
4.04
9.5 9.0 8.5 8.0 7.5 7.0 6.5 6.0 5.5 5.0 4.5 4.0 3.5 3.0 2.5 2.0 1.5 1.0 0.5 0.0
f1 (ppm)

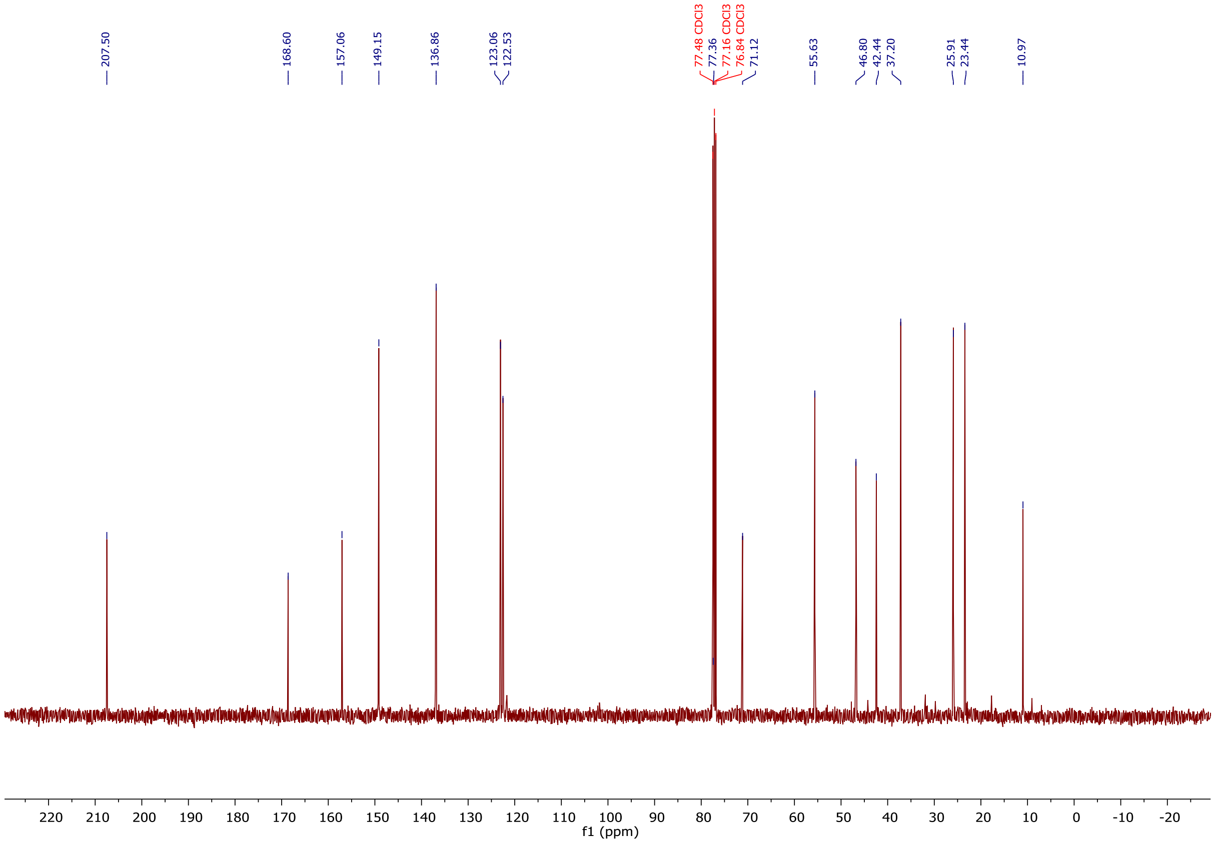
207.50
168.60
157.06
149.15
136.86
123.06
122.53
77.48 CDCl3
77.36
77.16 CDCl3
76.84 CDCl3
71.12
55.63
46.80
42.44
37.20
25.91
23.44
10.97
220 210 200 190 180 170 160 150 140 130 120 110 100 90 80 70 60 50 40 30 20 10 0 -10 -20
f1 (ppm)

Compound 2o

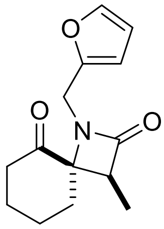

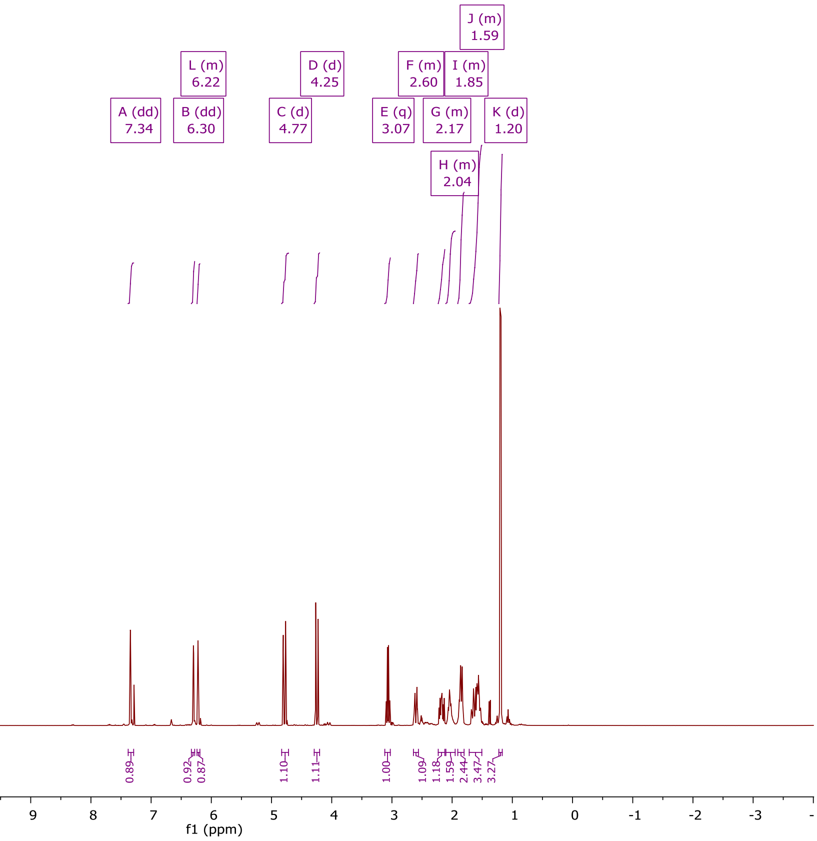

J (m) 1.59
L (m) 6.22
D (d) 4.25
F (m) 2.60
I (m) 1.85
A (dd) 7.34
B (dd) 6.30
C (d) 4.77
E (q) 3.07
G (m) 2.17
K (d) 1.20
H (m) 2.04
0.89
0.92
0.87
1.10
1.11
1.00
1.09
1.18
1.59
2.44
3.47
3.27
f1 (ppm)

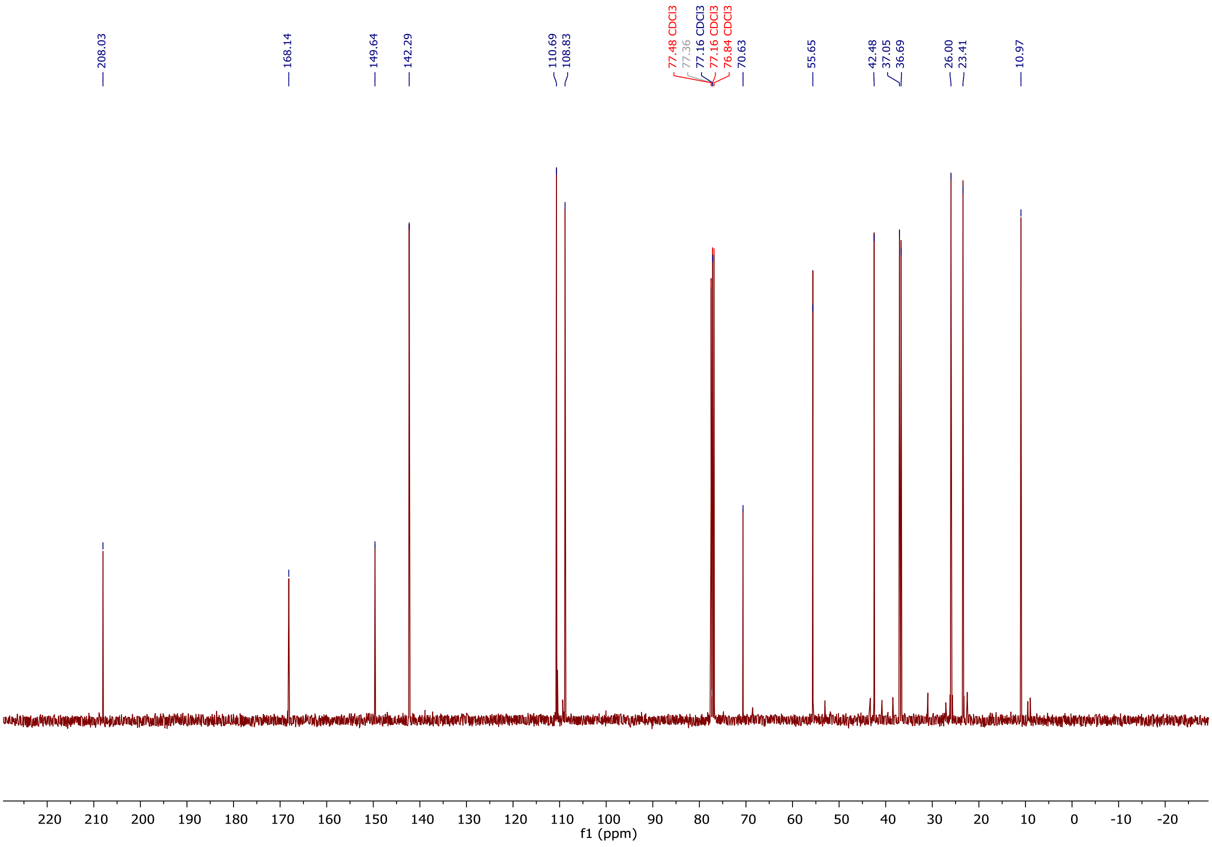

208.03
168.14
149.64
142.29
110.69
108.83
77.48 CDCl3
77.36
77.16 CDCl3
77.16 CDCl3
76.84 CDCl3
70.63
55.65
42.48
37.05
36.69
26.00
23.41
10.97
f1 (ppm)

Compound 2p

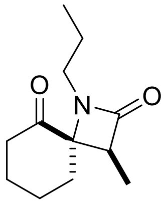

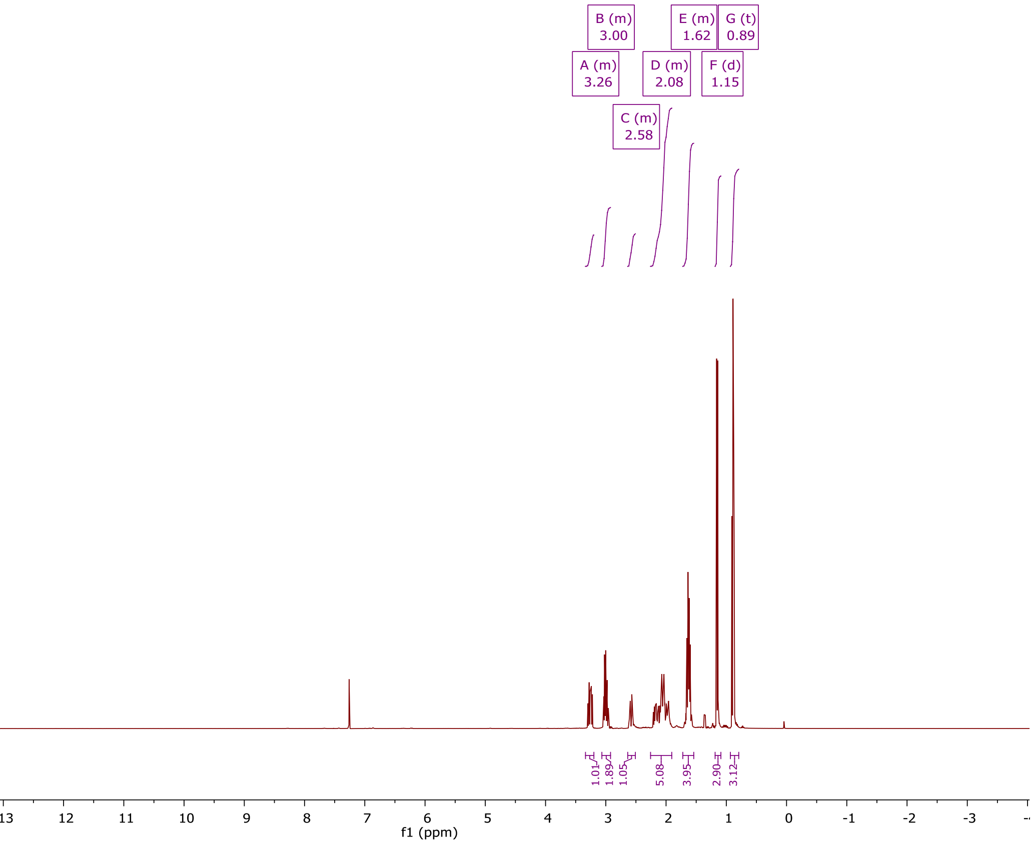

A (m)
3.26
B (m)
3.00
C (m)
2.58
D (m)
2.08
E (m)
1.62
F (d)
1.15
G (t)
0.89
1.01
1.89
1.05
5.08
3.95
2.90
3.12
f1 (ppm)

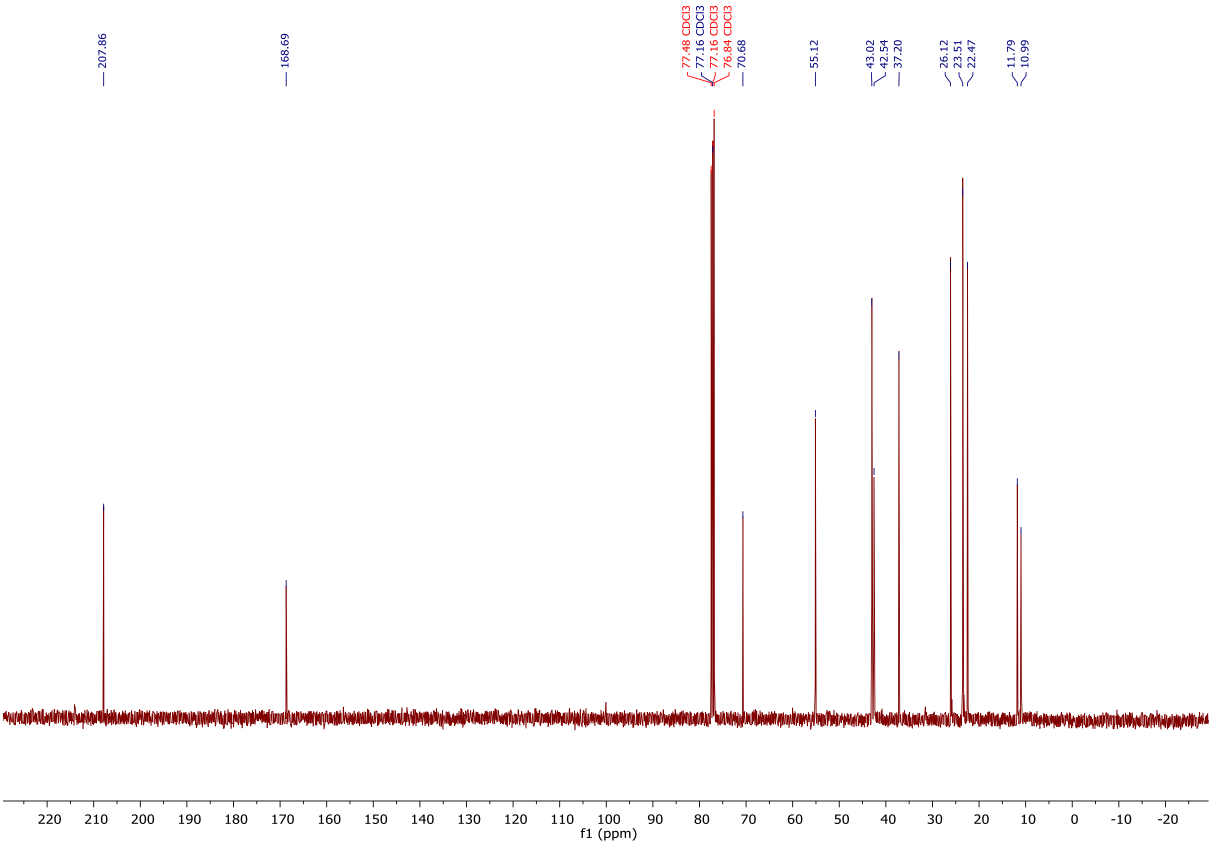

207.86
168.69
77.48 CDCl3
77.16 CDCl3
77.16 CDCl3
76.84 CDCl3
70.68
55.12
43.02
42.54
37.20
26.12
23.51
22.47
11.79
10.99
f1 (ppm)

Compound 2q

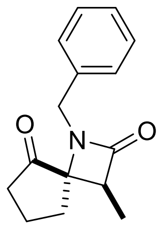

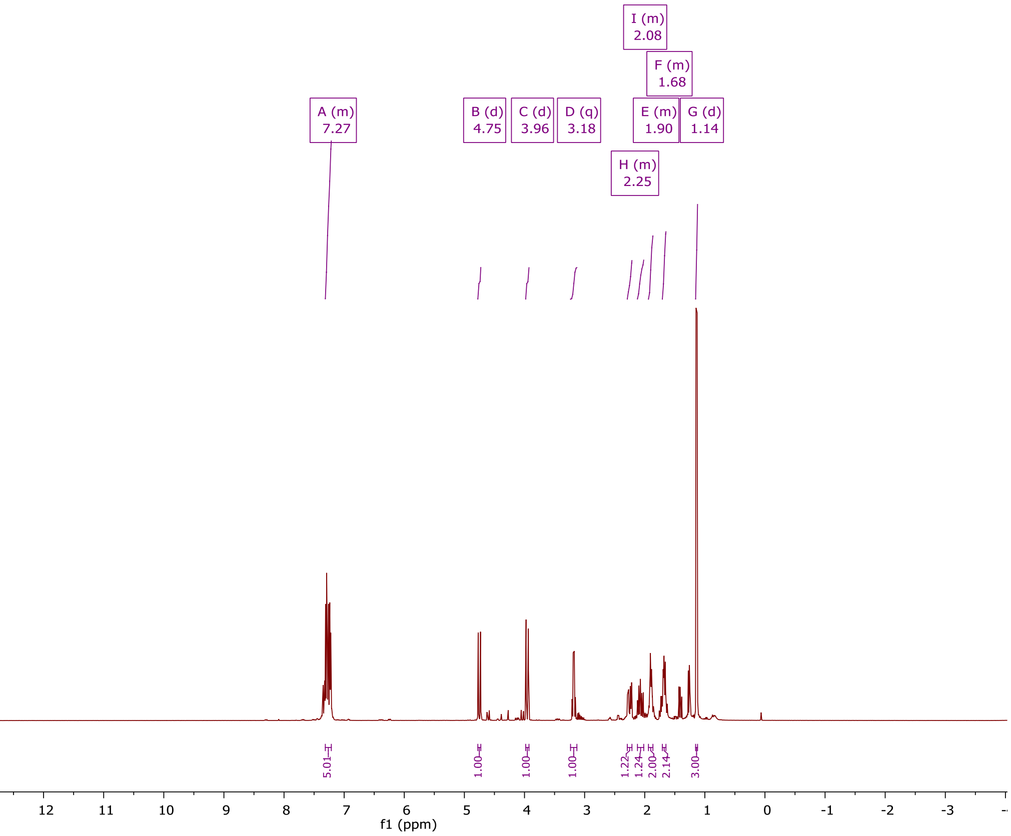

I (m) 2.08
F (m) 1.68
A (m) 7.27
B (d) 4.75
C (d) 3.96
D (q) 3.18
E (m) 1.90
G (d) 1.14
H (m) 2.25
5.01
1.00
1.00
1.00
1.22
1.24
2.00
2.14
3.00
f1 (ppm)

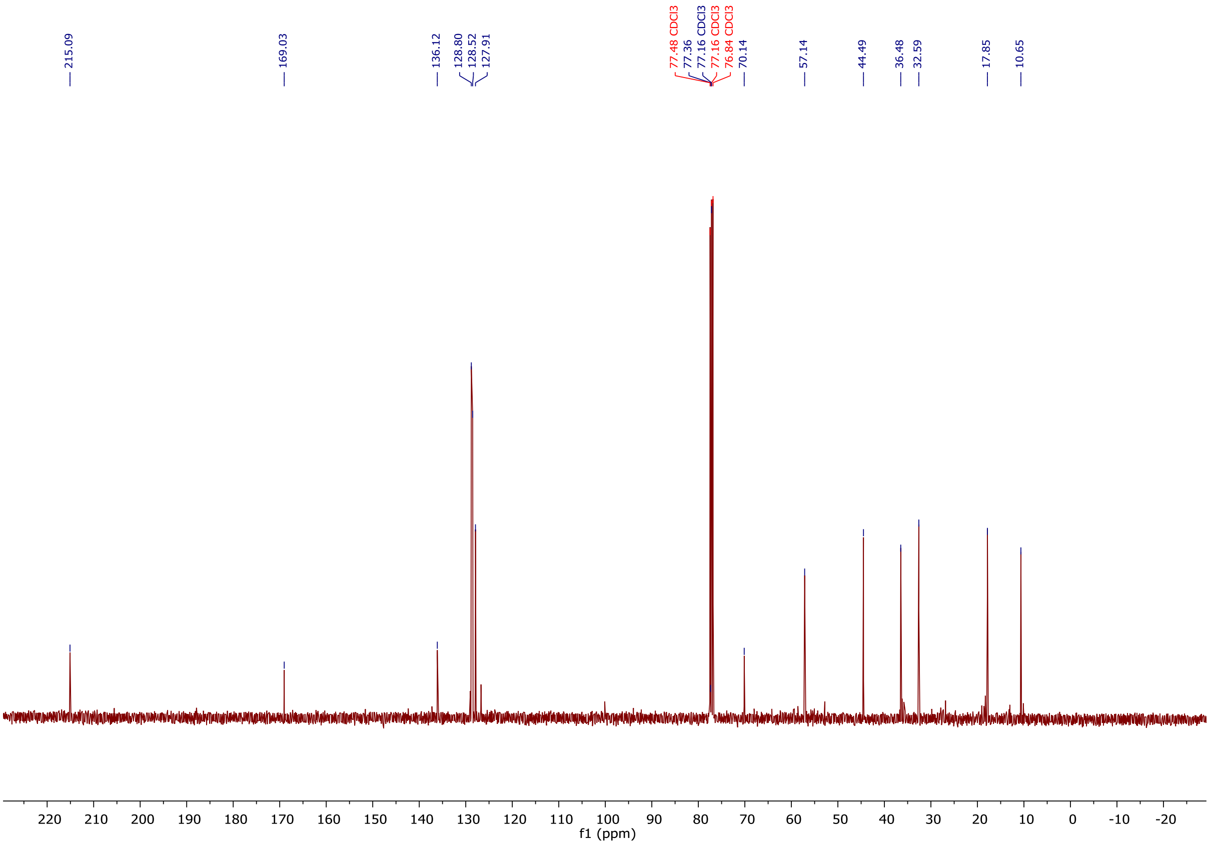

215.09
169.03
136.12
128.80
128.52
127.91
77.48 CDCl3
77.36 CDCl3
77.16 CDCl3
76.84 CDCl3
70.14
57.14
44.49
36.48
32.59
17.85
10.65
f1 (ppm)

Compound 2r

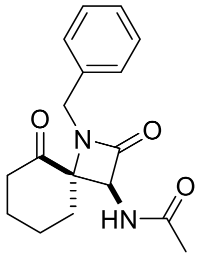

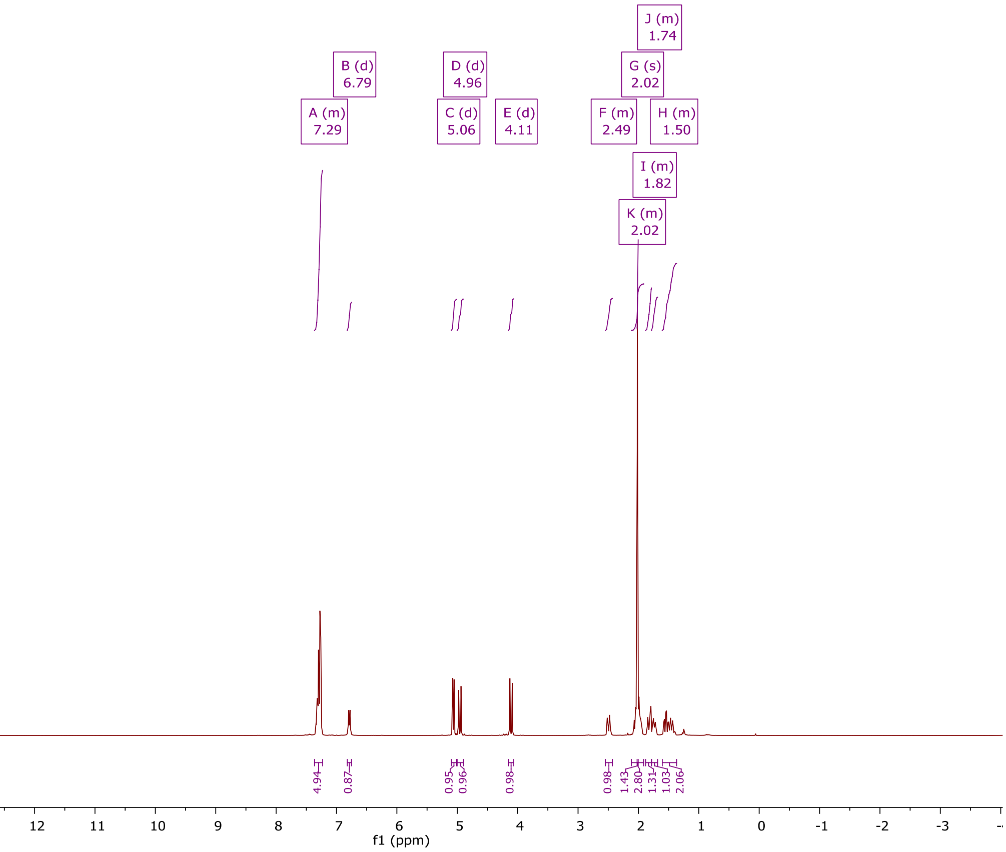

A (m) 7.29
B (d) 6.79
C (d) 5.06
D (d) 4.96
E (d) 4.11
F (m) 2.49
G (s) 2.02
H (m) 1.50
I (m) 1.82
J (m) 1.74
K (m) 2.02
4.94
0.87
0.95
0.96
0.98
0.98
1.43
2.80
1.31
1.03
2.06
f1 (ppm)

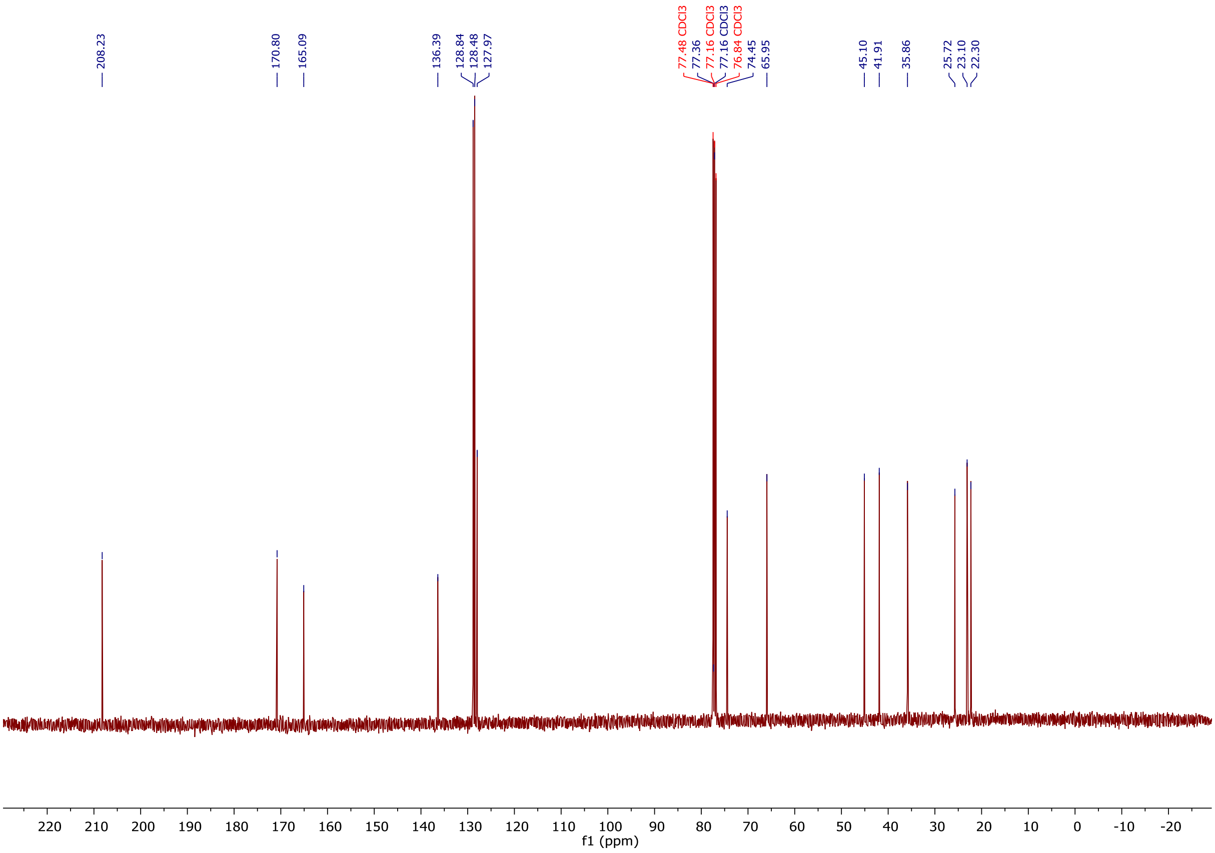

208.23
170.80
165.09
136.39
128.84
128.48
127.97
77.48 CDCl3
77.36
77.16 CDCl3
77.16 CDCl3
76.84 CDCl3
74.45
65.95
45.10
41.91
35.86
25.72
23.10
22.30
f1 (ppm)

Compound 2s

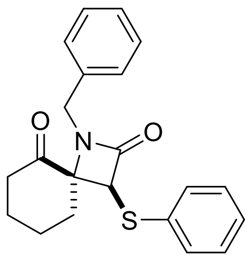
O
N
O
S

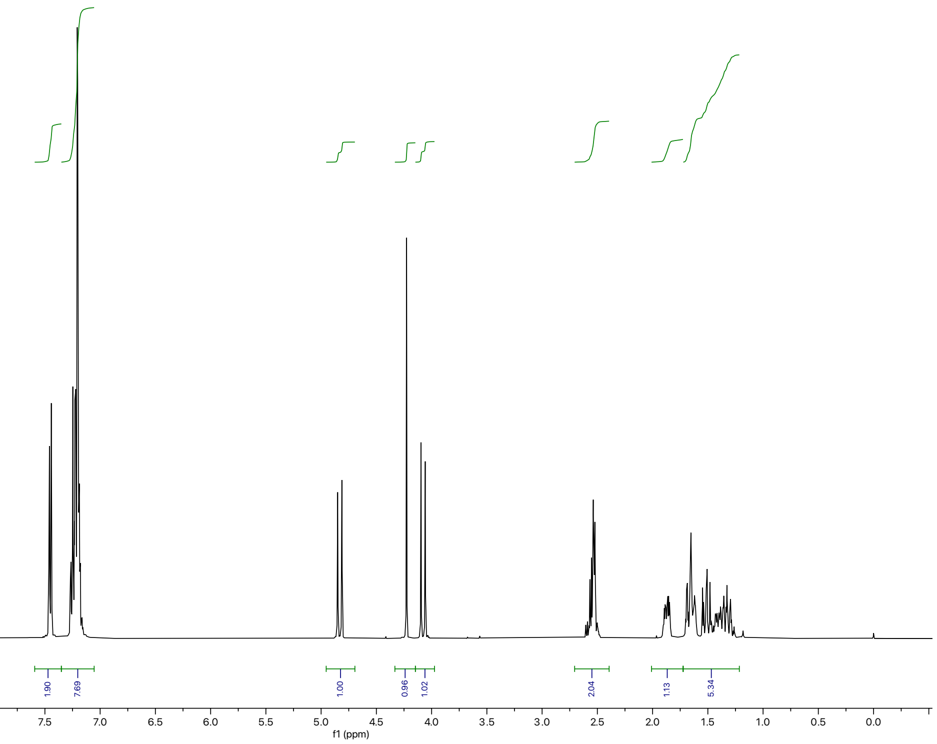
1.90
7.69
1.00
0.96
1.02
2.04
1.13
5.34
9.5 9.0 8.5 8.0 7.5 7.0 6.5 6.0 5.5 5.0 4.5 4.0 3.5 3.0 2.5 2.0 1.5 1.0 0.5 0.0
f1 (ppm)

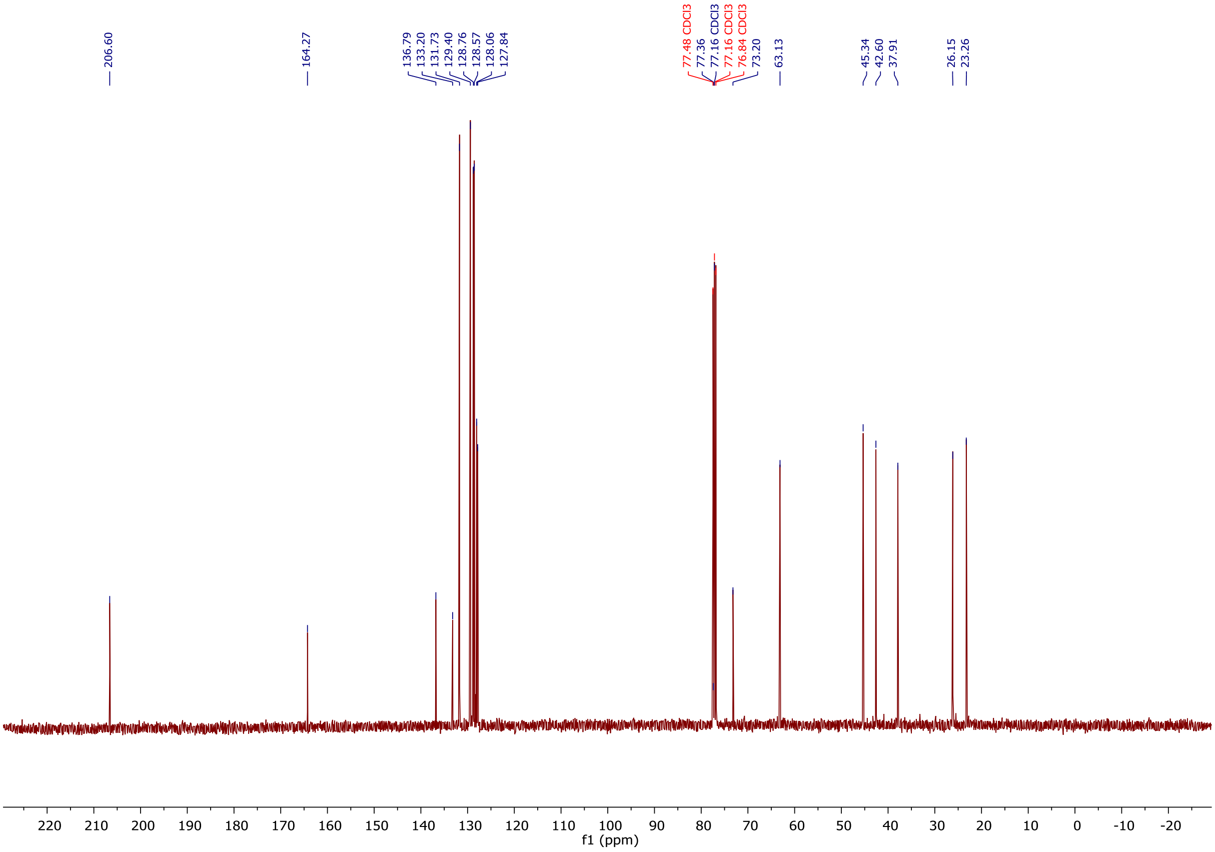
206.60
164.27
136.79
133.20
131.73
129.40
128.76
128.57
128.06
127.84
77.48 CDCl3
77.36 CDCl3
77.16 CDCl3
77.16 CDCl3
76.84 CDCl3
73.20
63.13
45.34
42.60
37.91
26.15
23.26
220 210 200 190 180 170 160 150 140 130 120 110 100 90 80 70 60 50 40 30 20 10 0 -10 -20
f1 (ppm)

Compound 2t

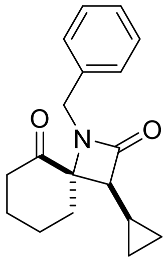

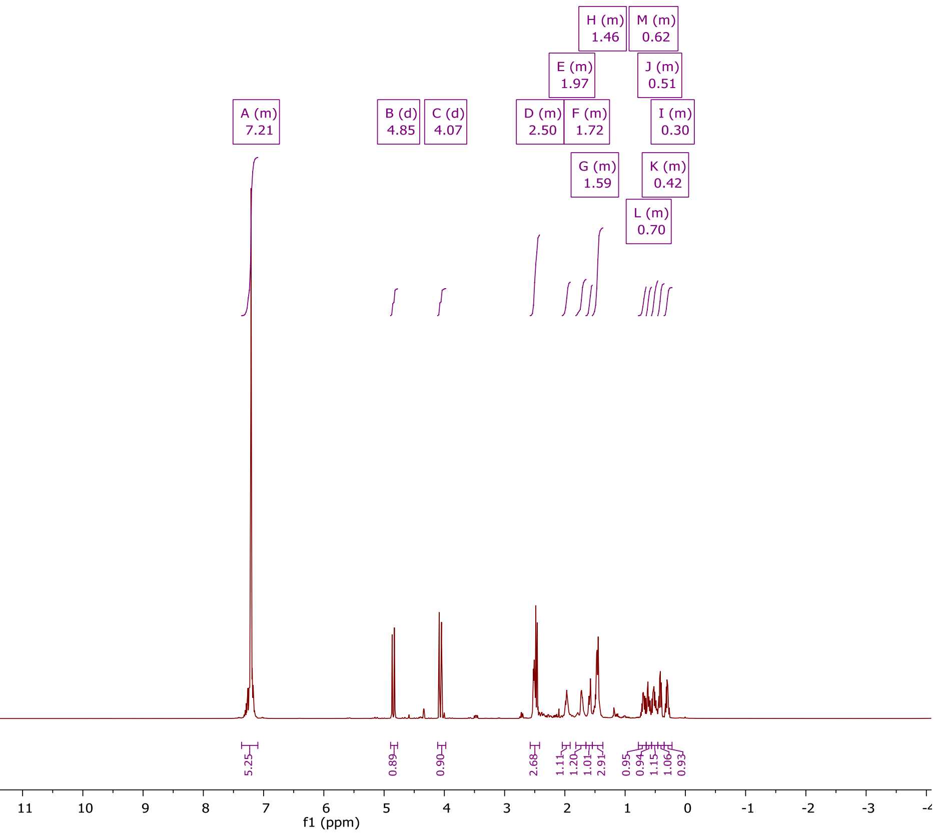

A (m)
7.21
B (d)
4.85
C (d)
4.07
D (m)
2.50
E (m)
1.97
F (m)
1.72
G (m)
1.59
H (m)
1.46
I (m)
0.30
J (m)
0.51
K (m)
0.42
L (m)
0.70
M (m)
0.62
5.25
0.89
0.90
2.68
1.11
1.20
1.01
2.91
0.95
0.94
1.15
1.06
0.93
f1 (ppm)

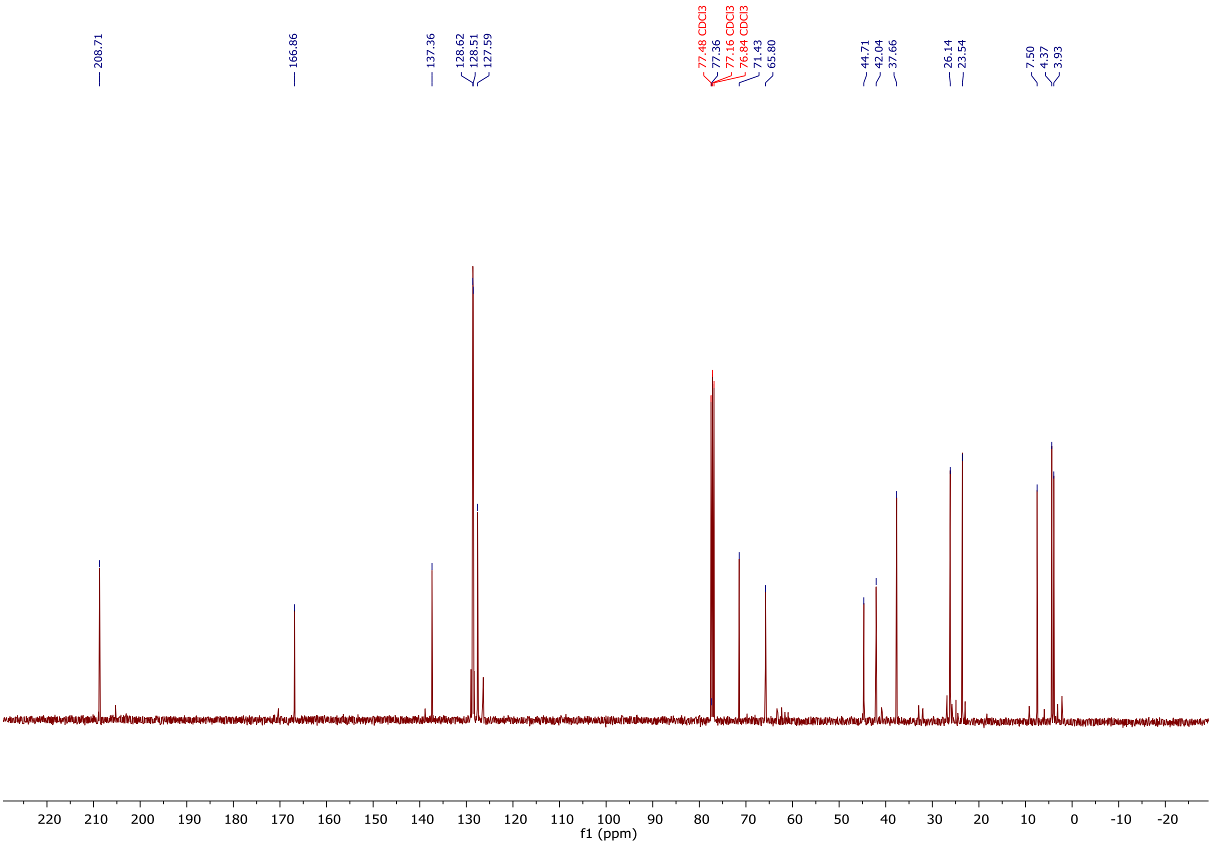

208.71
166.86
137.36
128.62
128.51
127.59
77.48 CDCl3
77.36
77.16 CDCl3
76.84 CDCl3
71.43
65.80
44.71
42.04
37.66
26.14
23.54
7.50
4.37
3.93
f1 (ppm)

Compound 2u

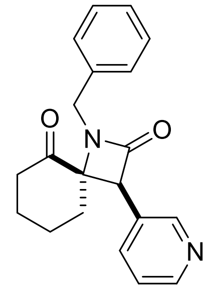
O
N
O
N

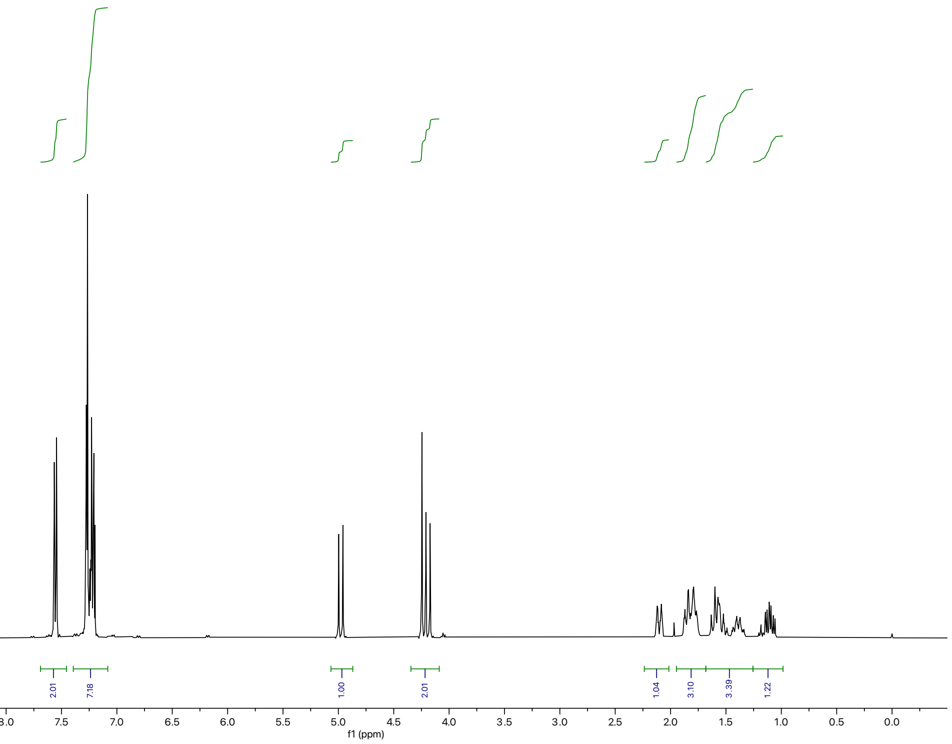
2.01
7.18
1.00
2.01
1.04
3.10
3.39
1.22
9.5 9.0 8.5 8.0 7.5 7.0 6.5 6.0 5.5 5.0 4.5 4.0 3.5 3.0 2.5 2.0 1.5 1.0 0.5 0.0
f1 (ppm)

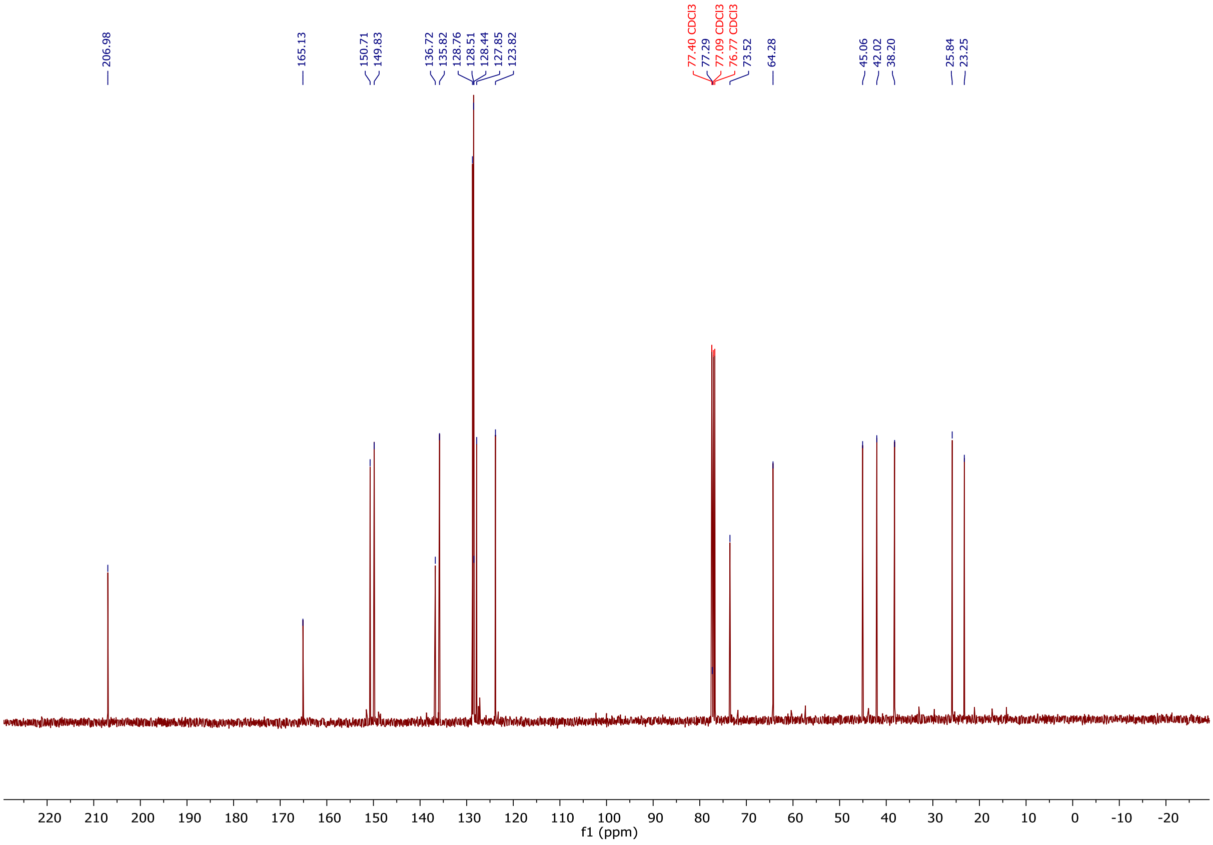
206.98
165.13
150.71
149.83
136.72
135.82
128.76
128.51
128.44
127.85
123.82
77.40 CDCl3
77.29
77.09 CDCl3
76.77 CDCl3
73.52
64.28
45.06
42.02
38.20
25.84
23.25
220 210 200 190 180 170 160 150 140 130 120 110 100 90 80 70 60 50 40 30 20 10 0 -10 -20
f1 (ppm)

Compound 2a-d_2

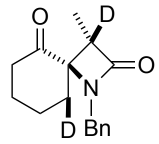

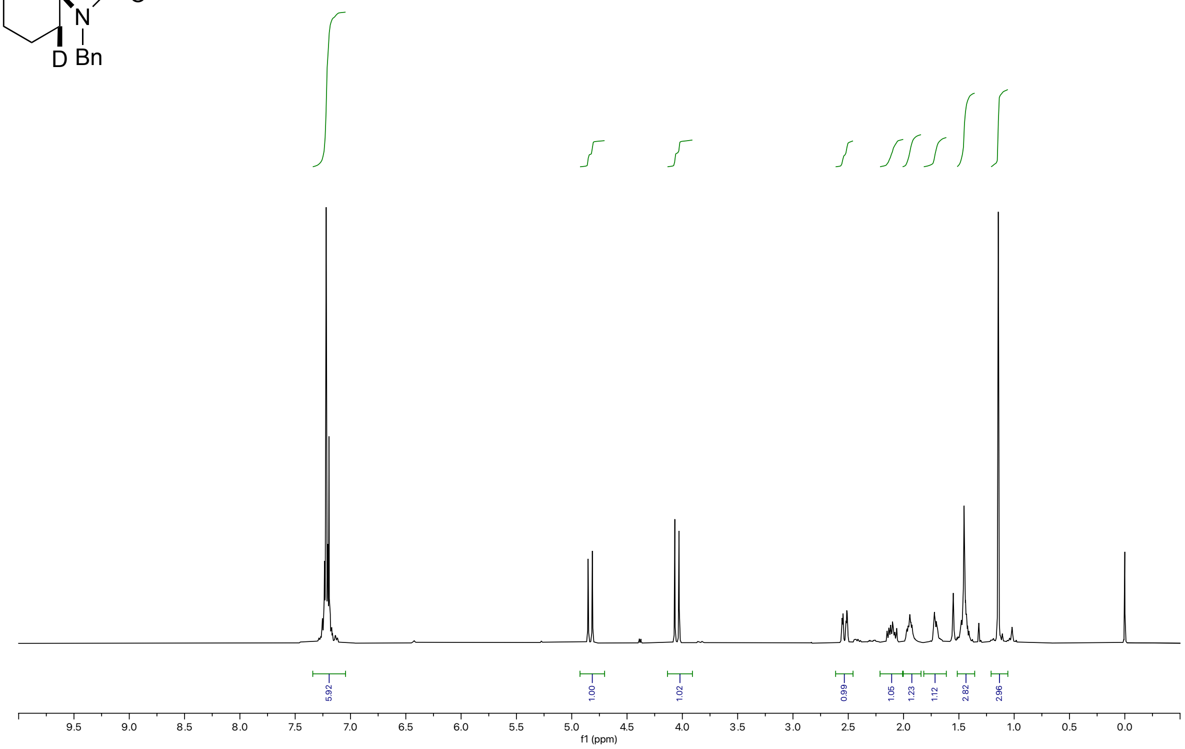

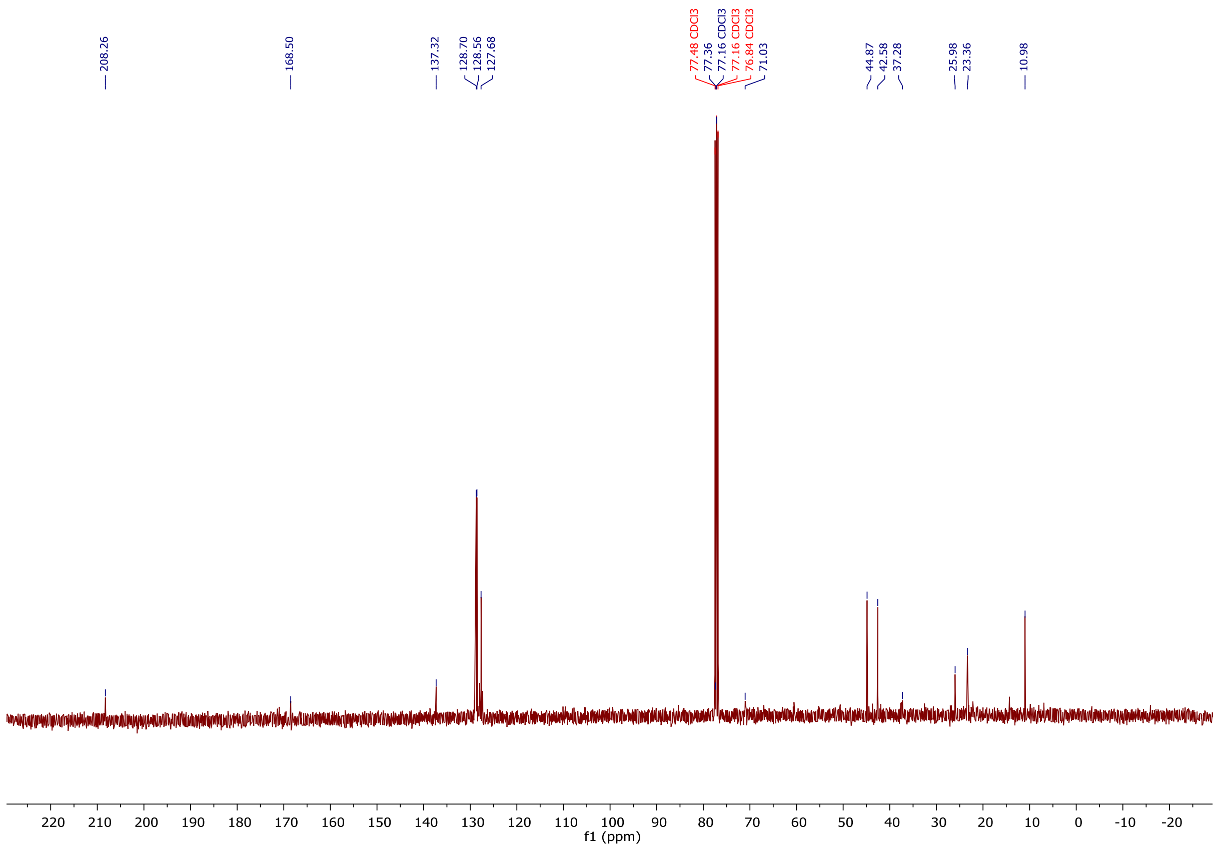

Compound 7a

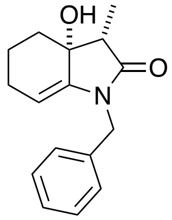
OH
O
N

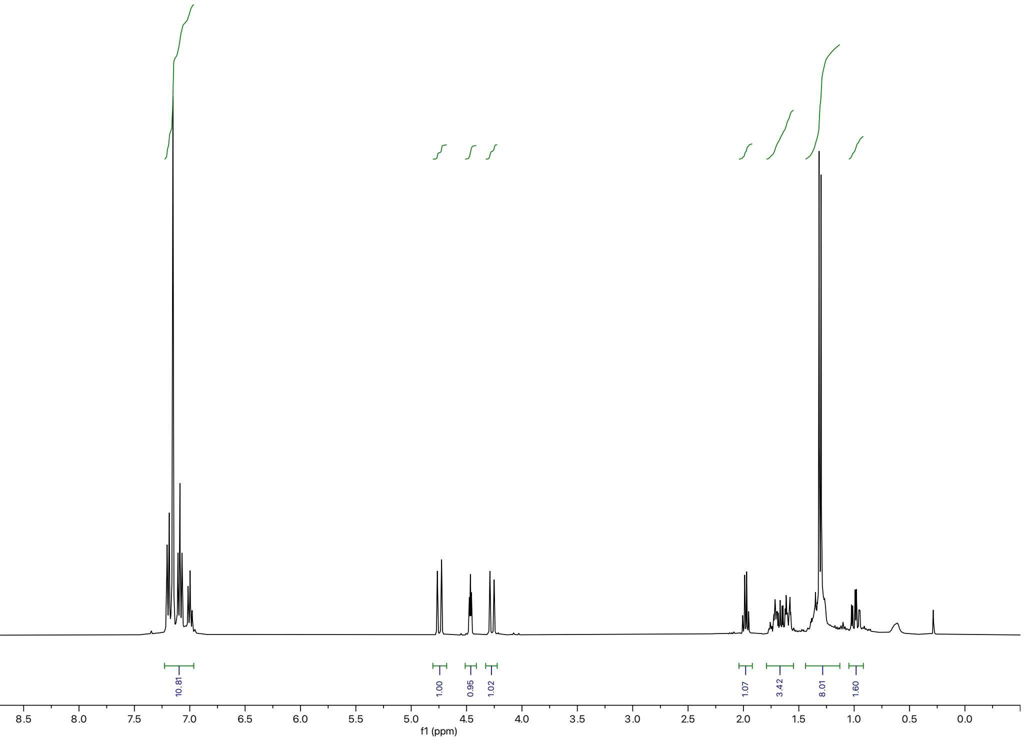
10.81
1.00
0.95
1.02
1.07
3.42
8.01
1.60
9.5
9.0
8.5
8.0
7.5
7.0
6.5
6.0
5.5
5.0
4.5
4.0
3.5
3.0
2.5
2.0
1.5
1.0
0.5
0.0
f1 (ppm)

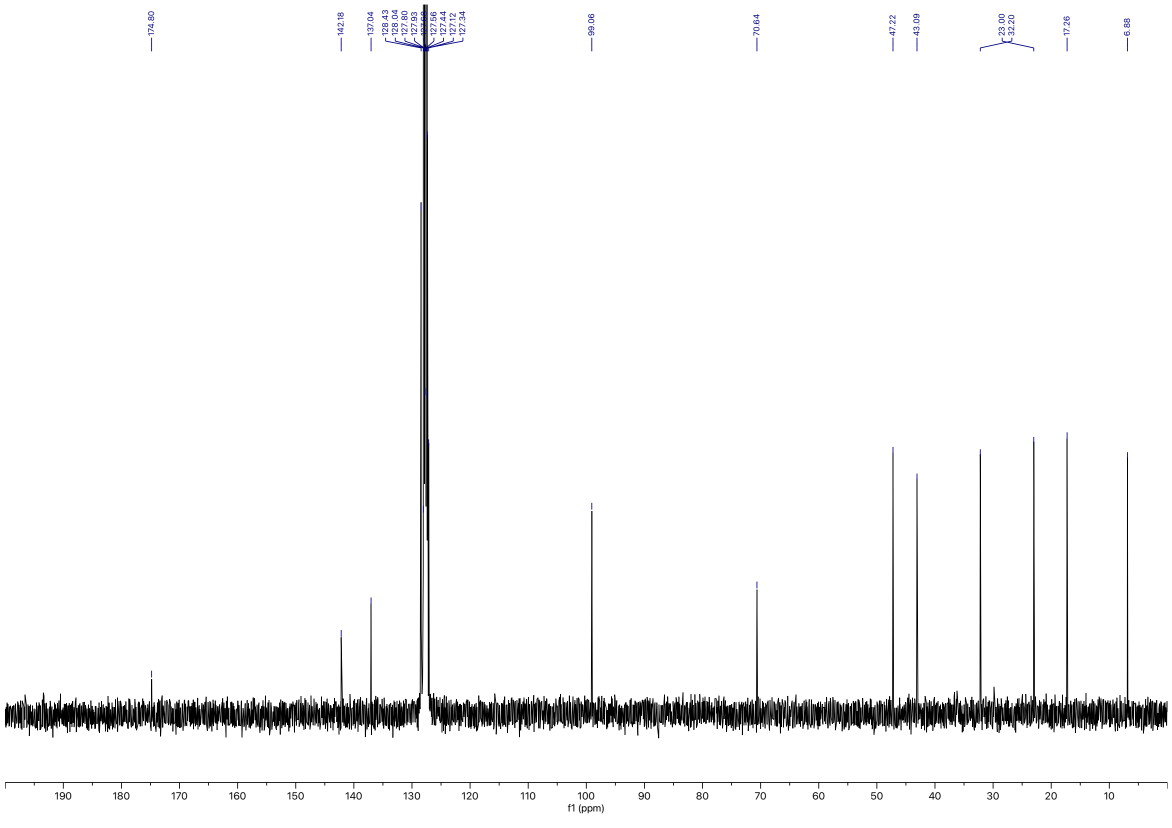
174.80
142.18
137.04
128.43
128.04
127.93
127.80
127.56
127.44
127.12
127.34
99.06
70.64
47.22
43.09
23.00
32.20
17.26
6.88
190
180
170
160
150
140
130
120
110
100
90
80
70
60
50
40
30
20
10
f1 (ppm)

Compound 11

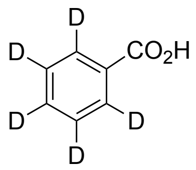

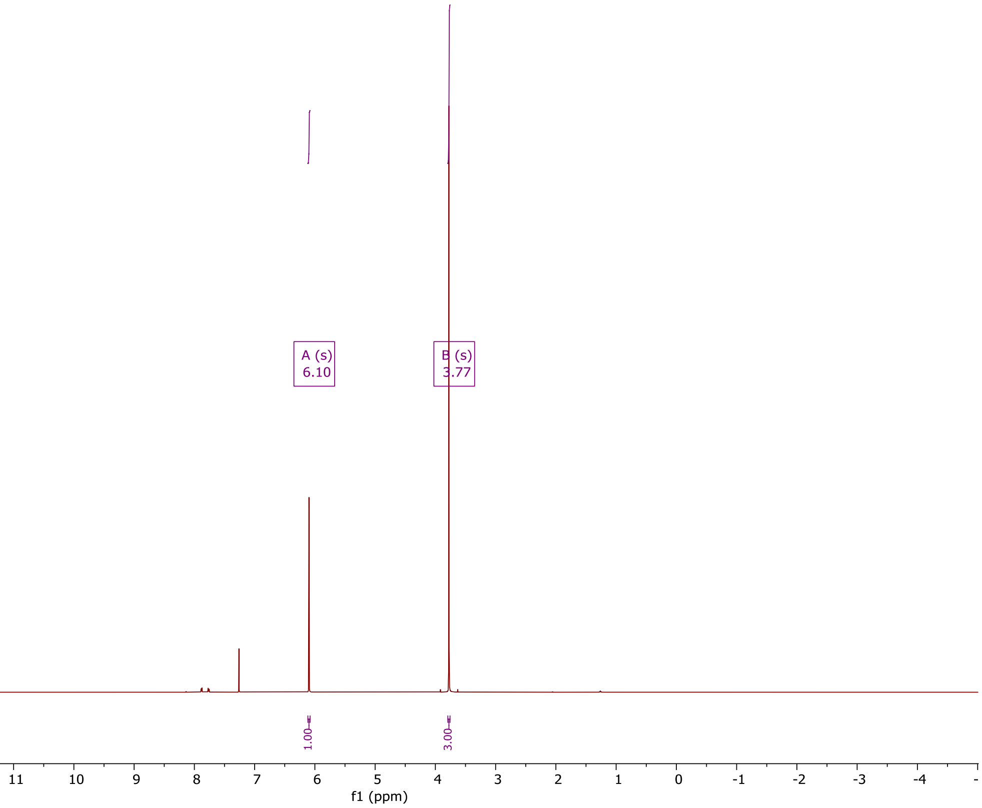
A (s)
6.10
B (s)
3.77
1.00
3.00
f1 (ppm)

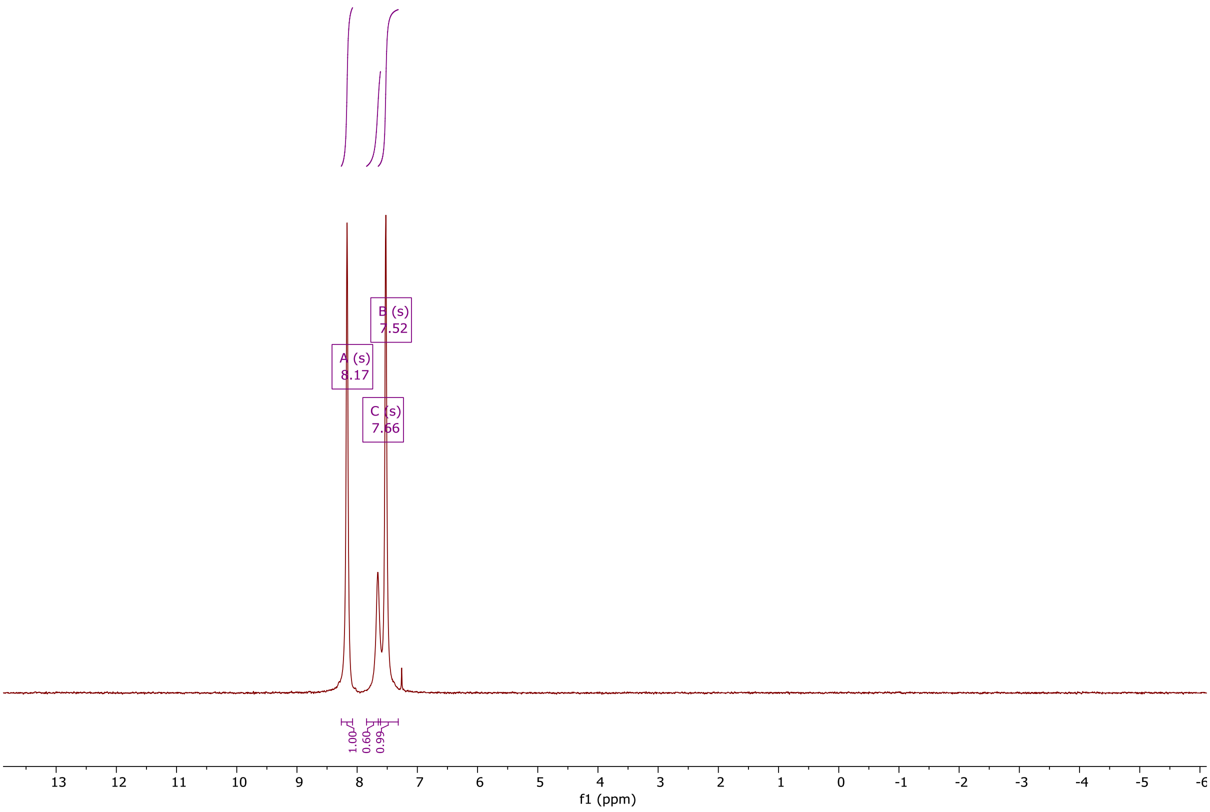
B (s)
7.52
A (s)
8.17
C (s)
7.66
1.00
0.60
0.99
f1 (ppm)

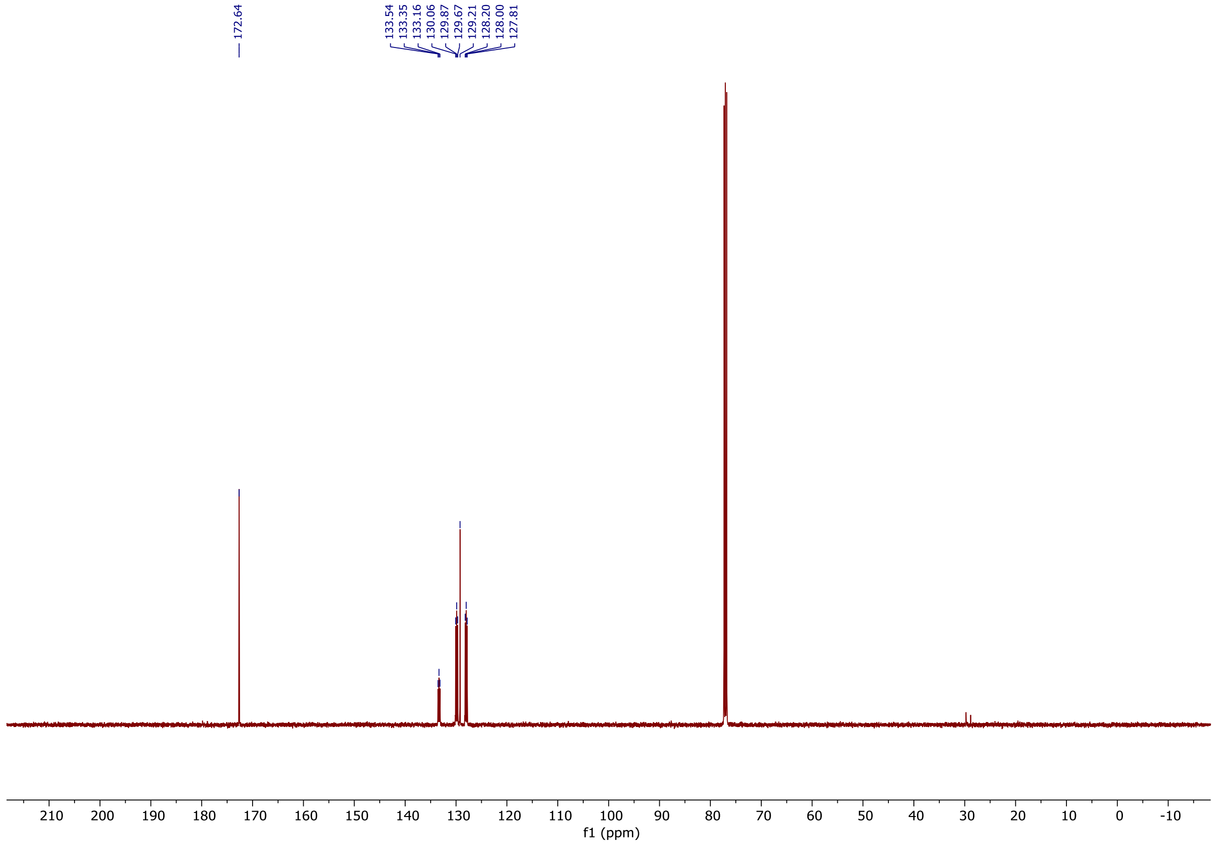
172.64
133.54
133.35
133.16
130.06
129.87
129.67
129.21
128.20
128.00
127.81
210 200 190 180 170 160 150 140 130 120 110 100 90 80 70 60 50 40 30 20 10 0 -10
f1 (ppm)

Compound 12

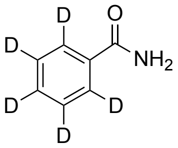
D
O
D
NH2
D
D
D

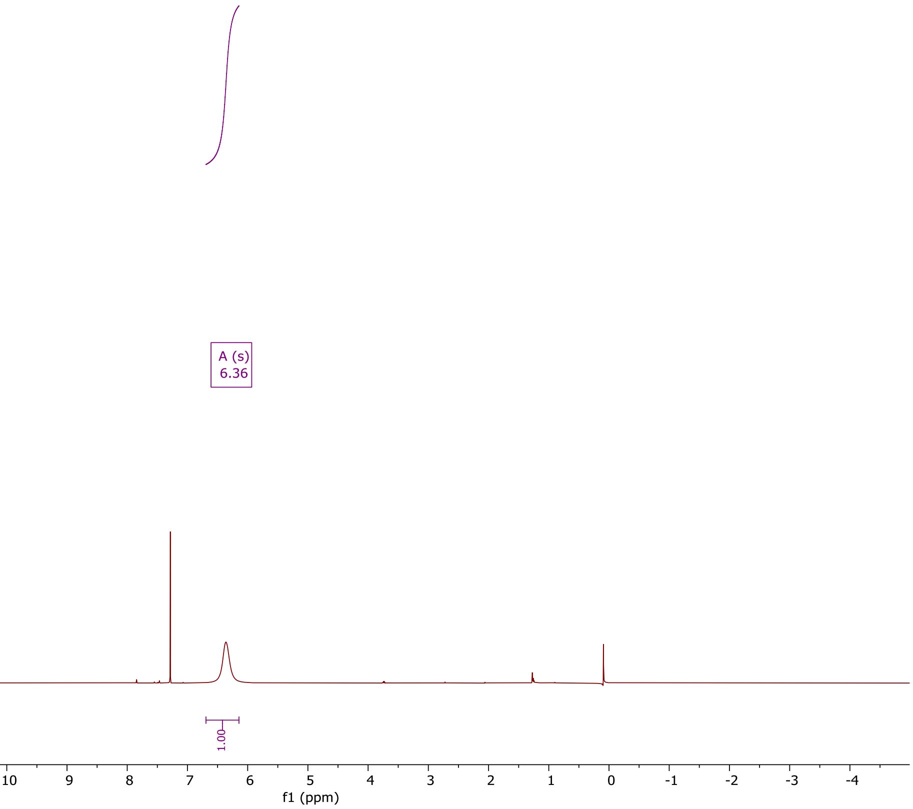
A (s)
6.36
1.00
14
13
12
11
10
9
8
7
6
5
4
3
2
1
0
-1
-2
-3
-4
f1 (ppm)

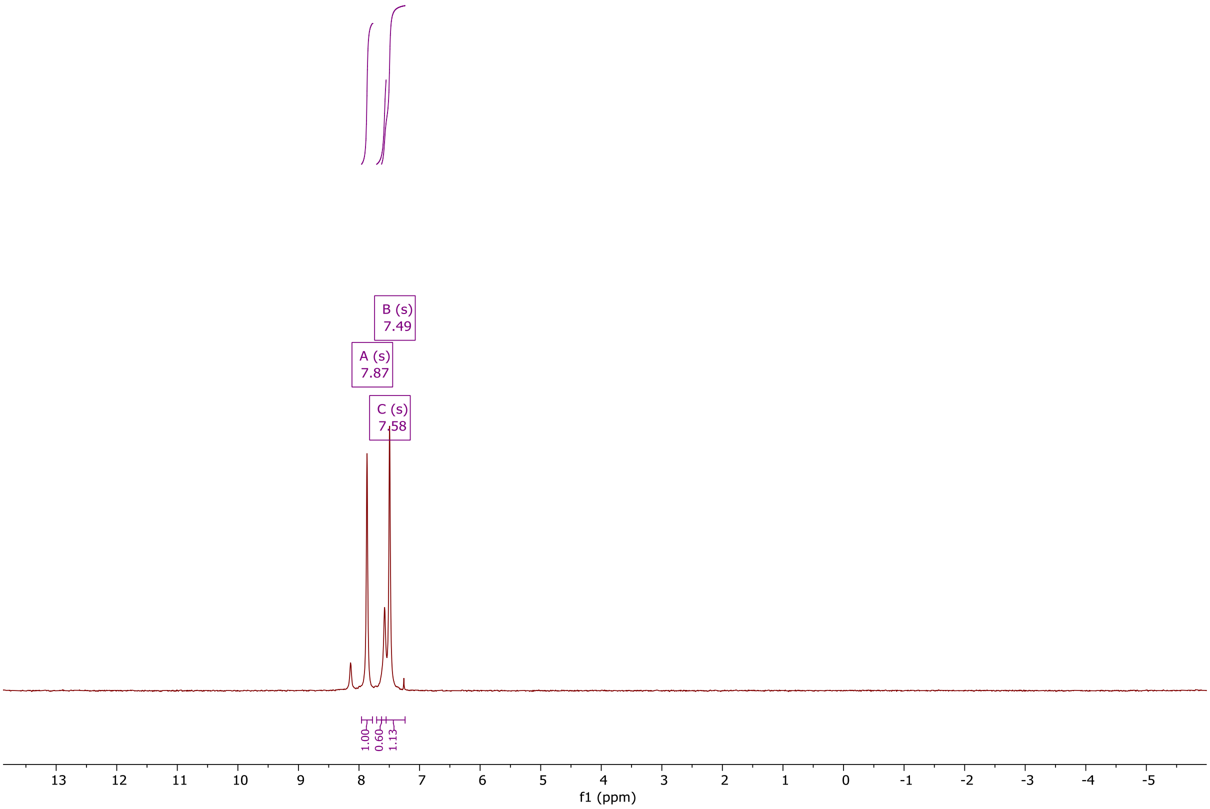
B (s)
7.49
A (s)
7.87
C (s)
7.58
1.00
0.60
1.13
13
12
11
10
9
8
7
6
5
4
3
2
1
0
-1
-2
-3
-4
-5
f1 (ppm)

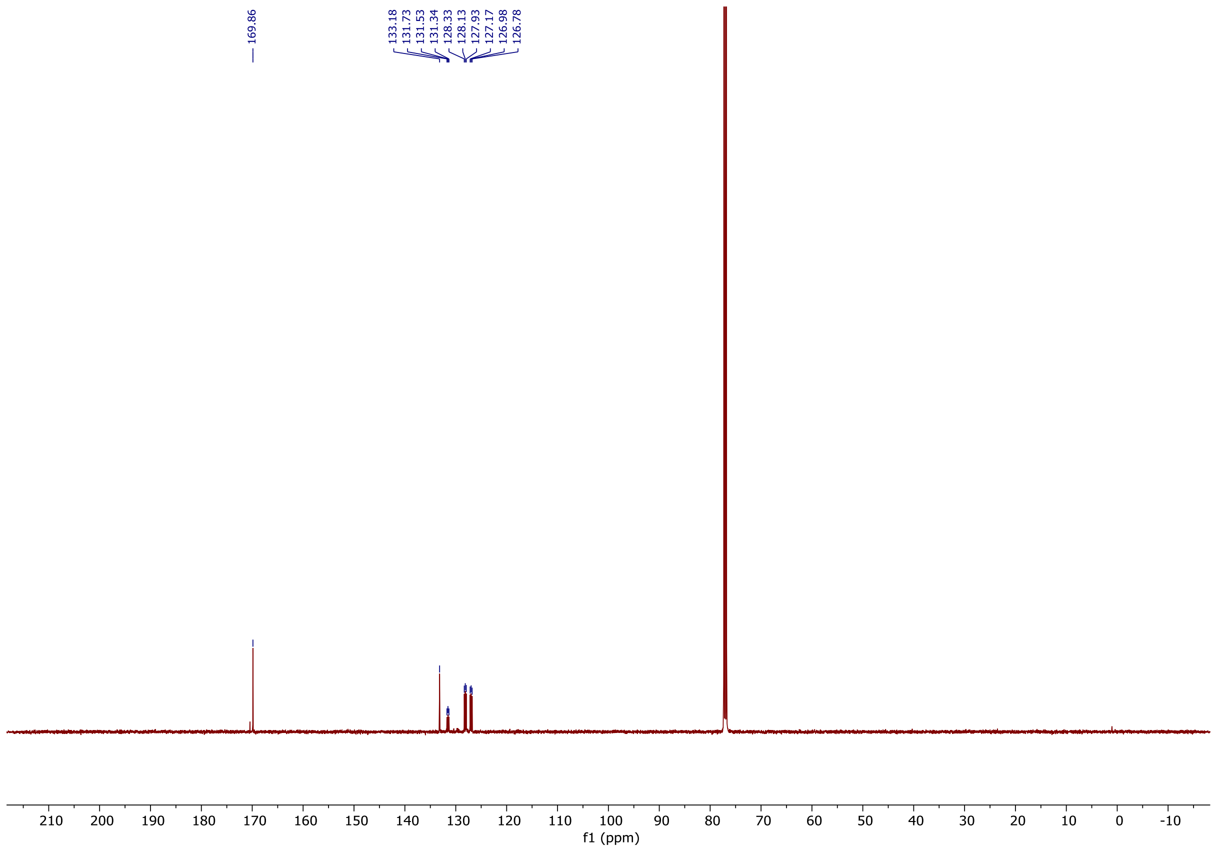

169.86
133.18
131.73
131.53
131.34
128.33
128.13
127.93
127.17
126.98
126.78
210
200
190
180
170
160
150
140
130
120
110
100
90
80
70
60
50
40
30
20
10
0
-10
f1 (ppm)

Compound 13

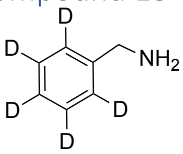

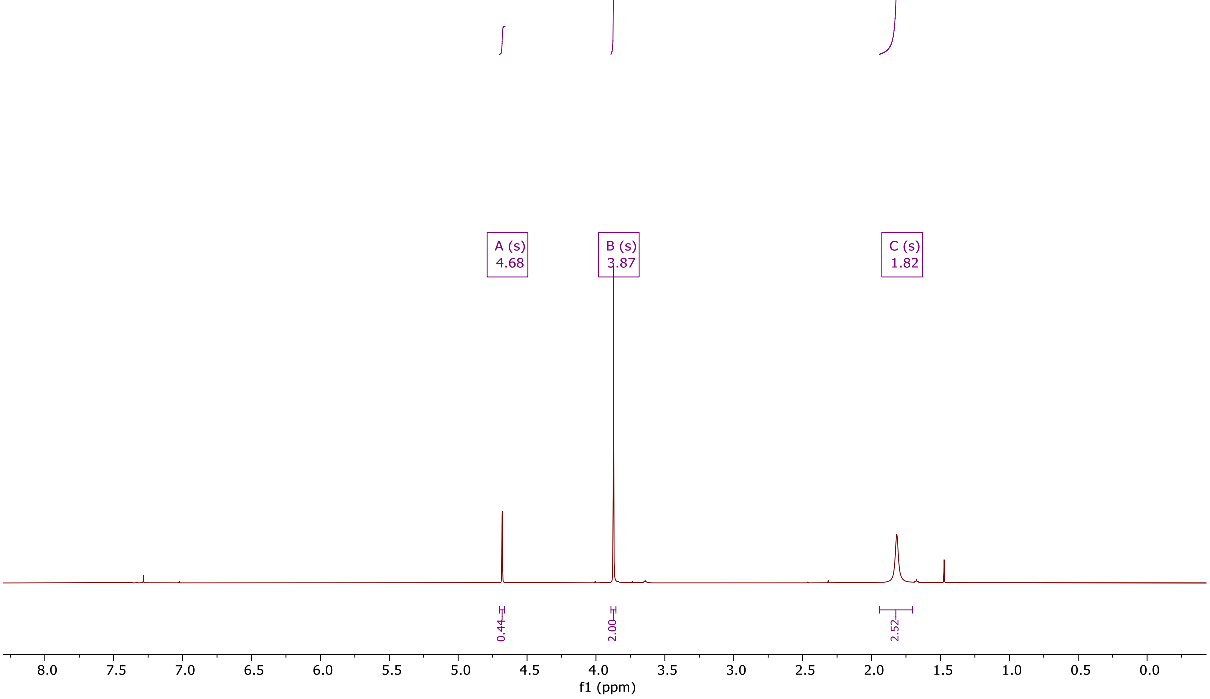

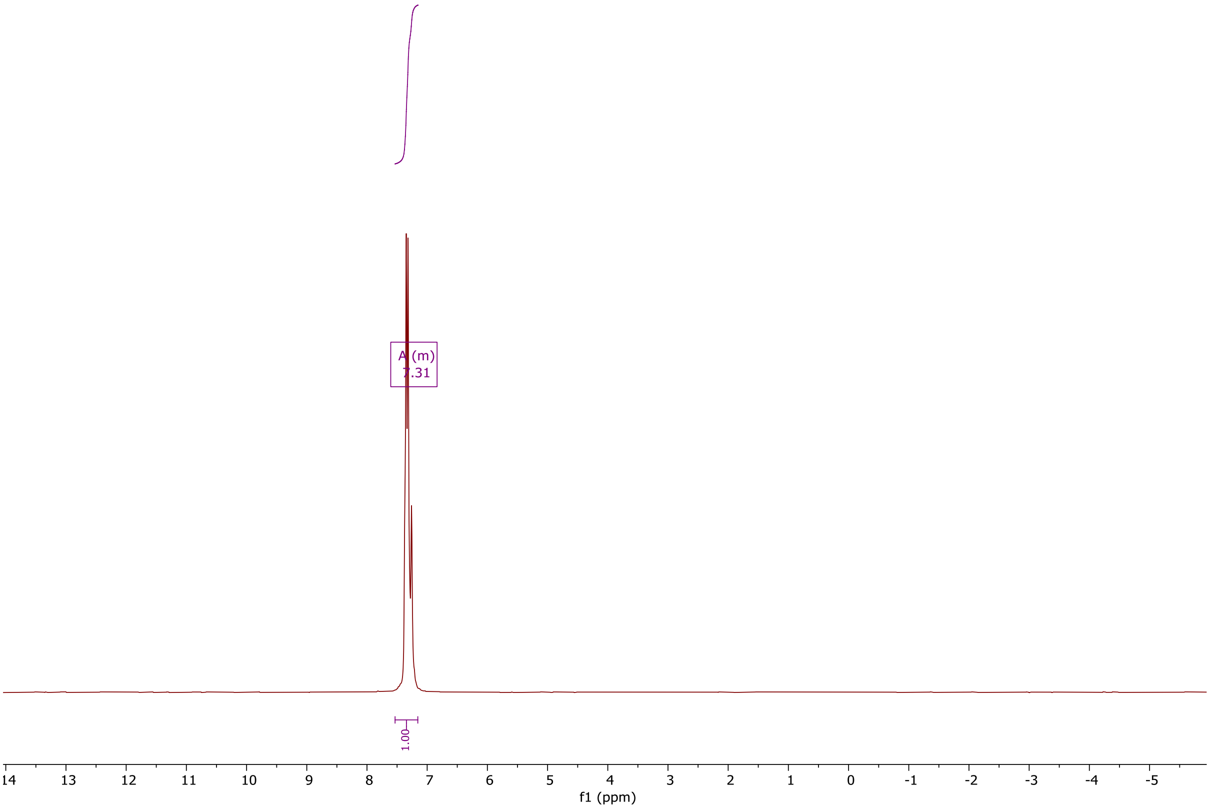

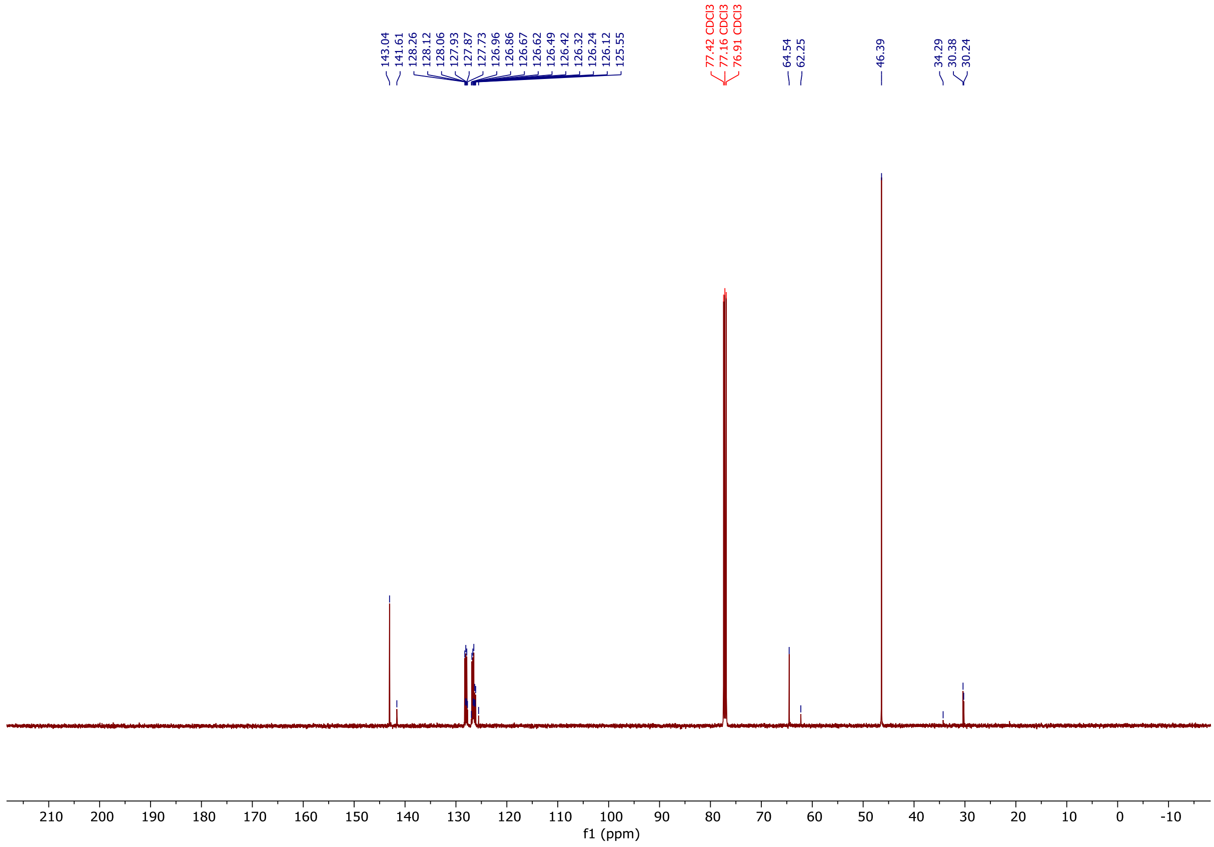
143.04
141.61
128.26
128.12
128.06
127.93
127.87
127.73
126.96
126.86
126.67
126.62
126.49
126.42
126.32
126.24
126.12
125.55
77.42 CDCl3
77.16 CDCl3
76.91 CDCl3
64.54
62.25
46.39
34.29
30.38
30.24
210 200 190 180 170 160 150 140 130 120 110 100 90 80 70 60 50 40 30 20 10 0 -10
f1 (ppm)

Compound 14

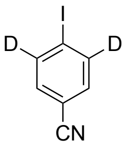

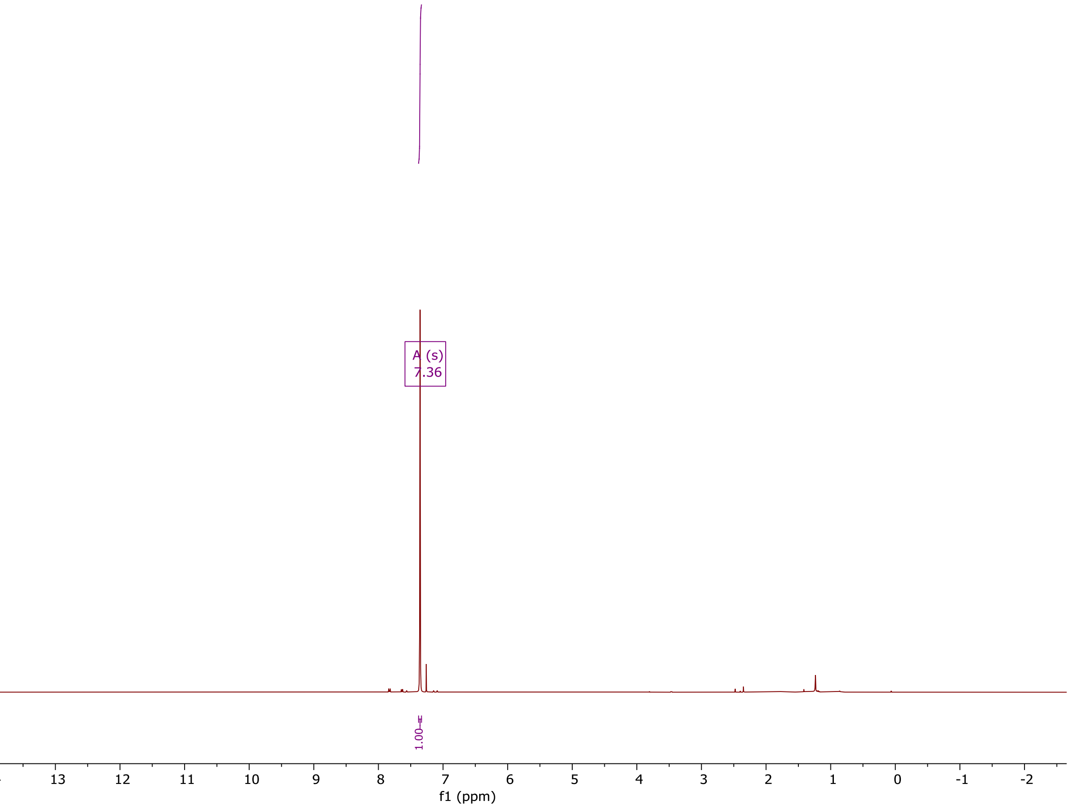

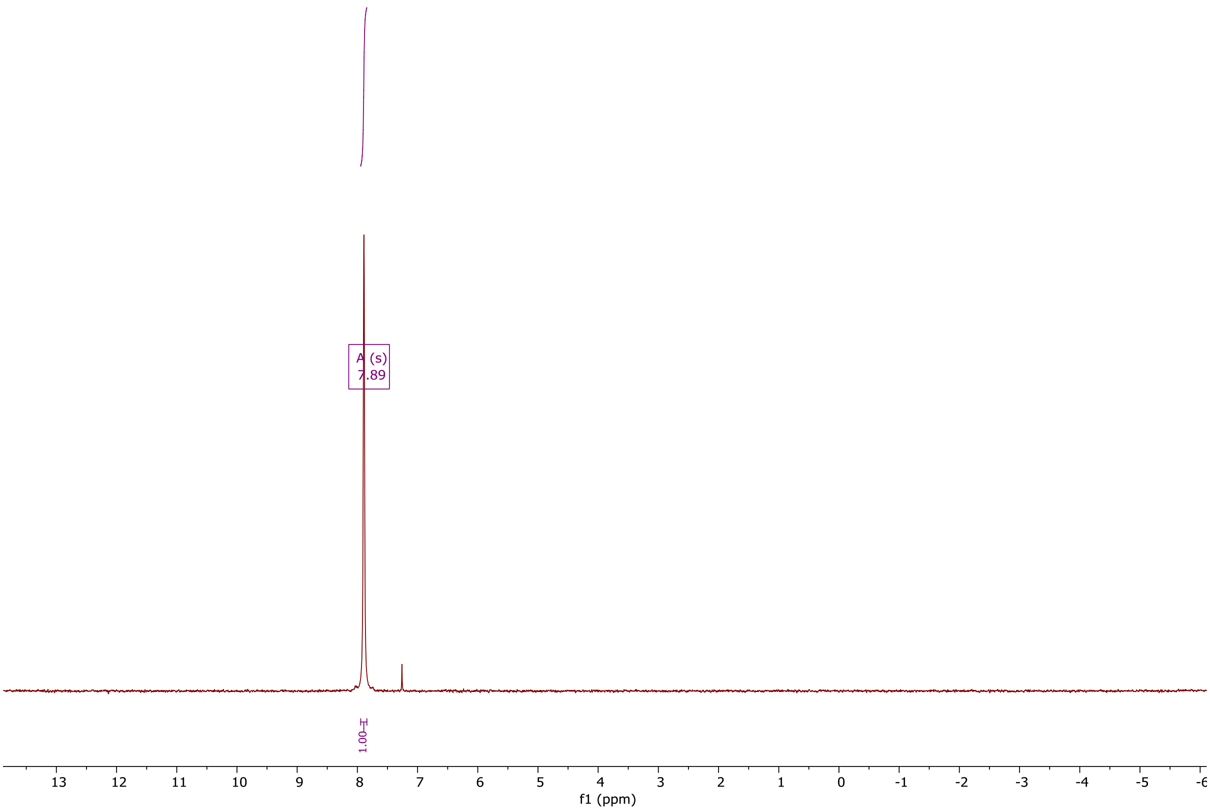

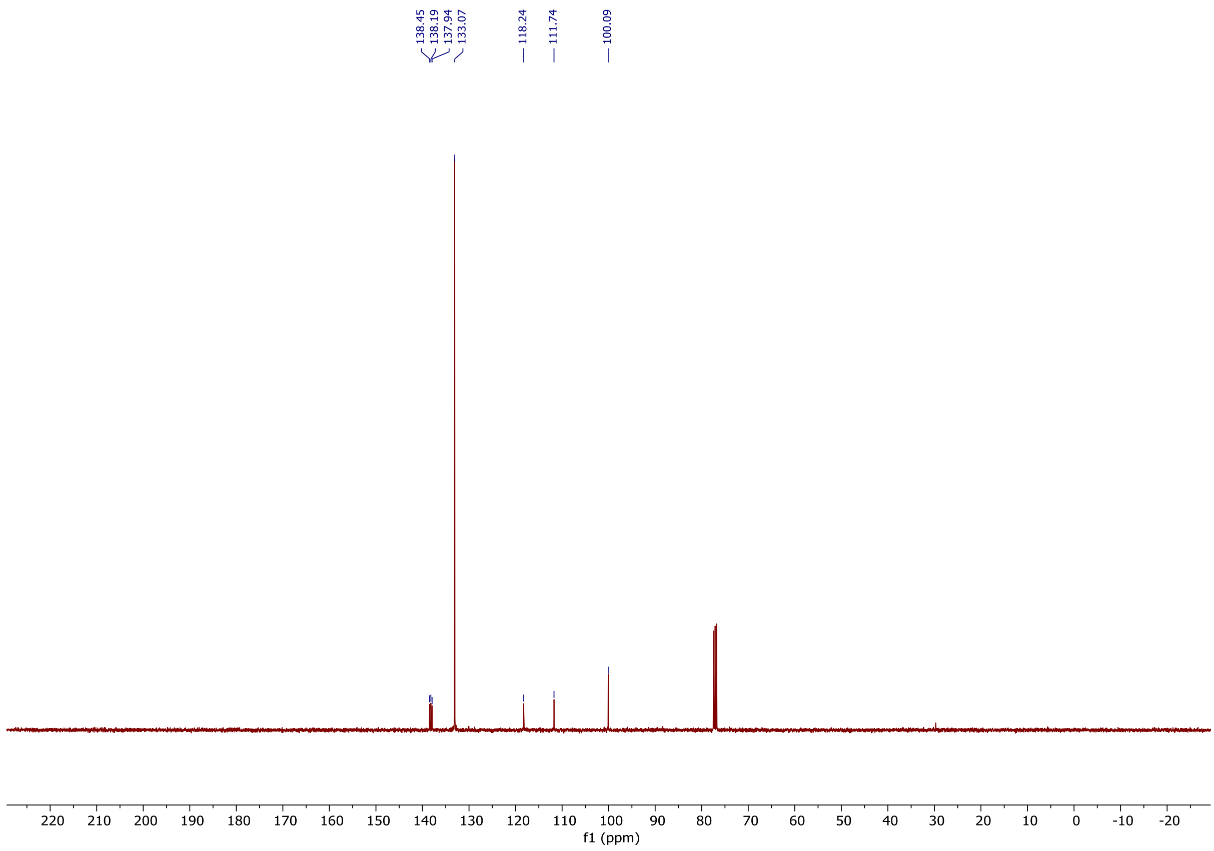
138.45
138.19
137.94
133.07
118.24
111.74
100.09
220 210 200 190 180 170 160 150 140 130 120 110 100 90 80 70 60 50 40 30 20 10 0 -10 -20
f1 (ppm)

Compound 15

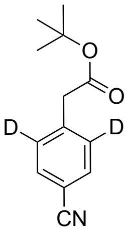

D
D
O
O
CN

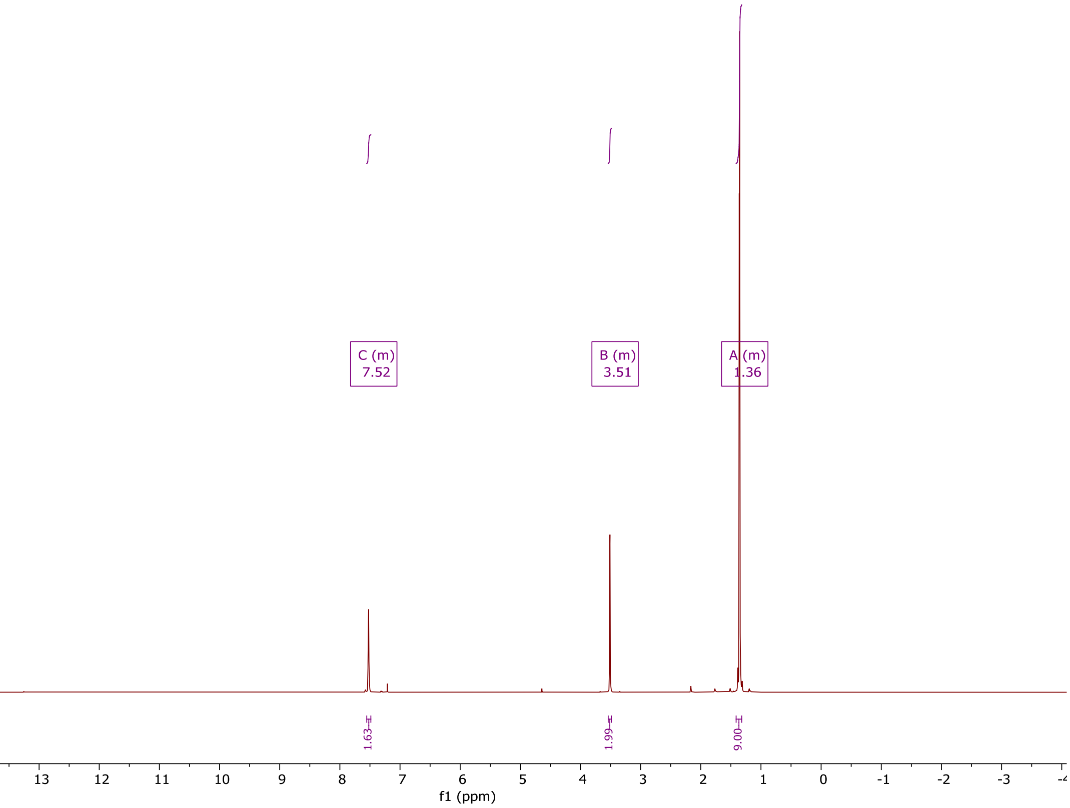

C (m)
7.52
B (m)
3.51
A (m)
1.36
1.63
1.99
9.00
15 14 13 12 11 10 9 8 7 6 5 4 3 2 1 0 -1 -2 -3
f1 (ppm)

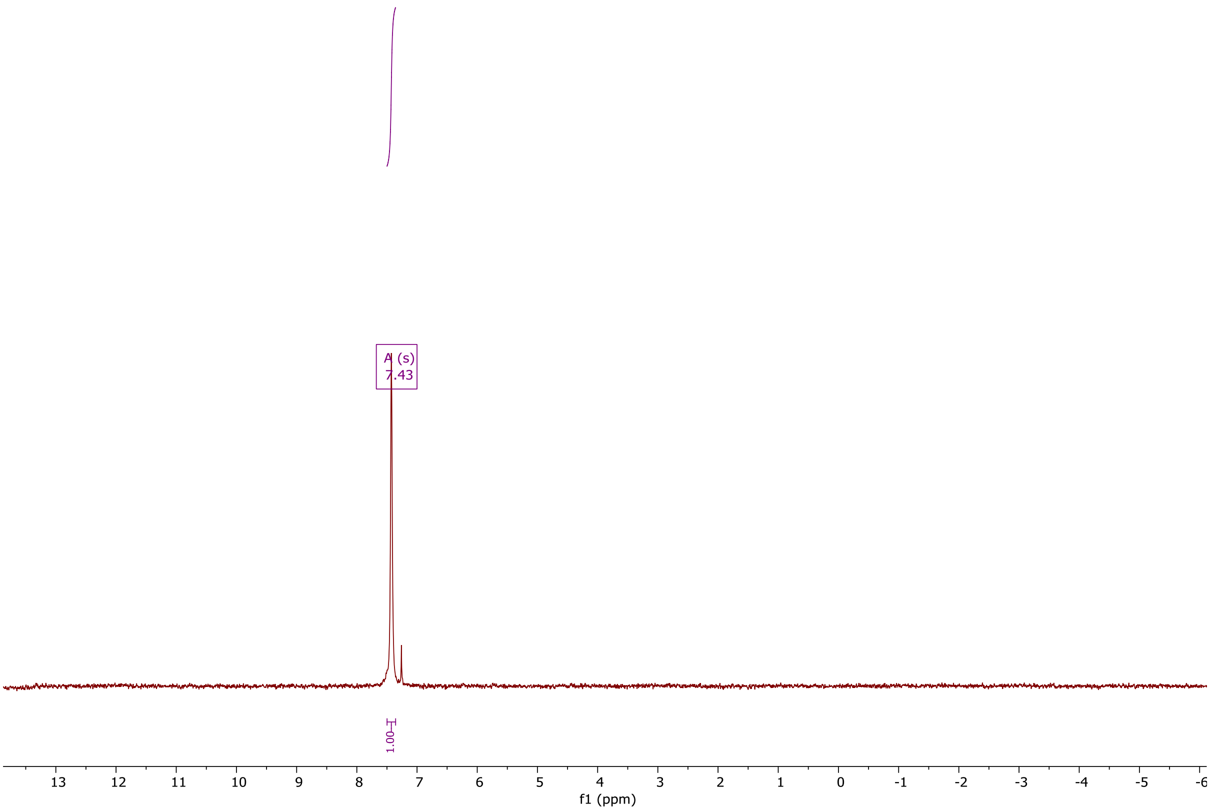

A (s)
7.43
1.00
13 12 11 10 9 8 7 6 5 4 3 2 1 0 -1 -2 -3 -4 -5 -6
f1 (ppm)

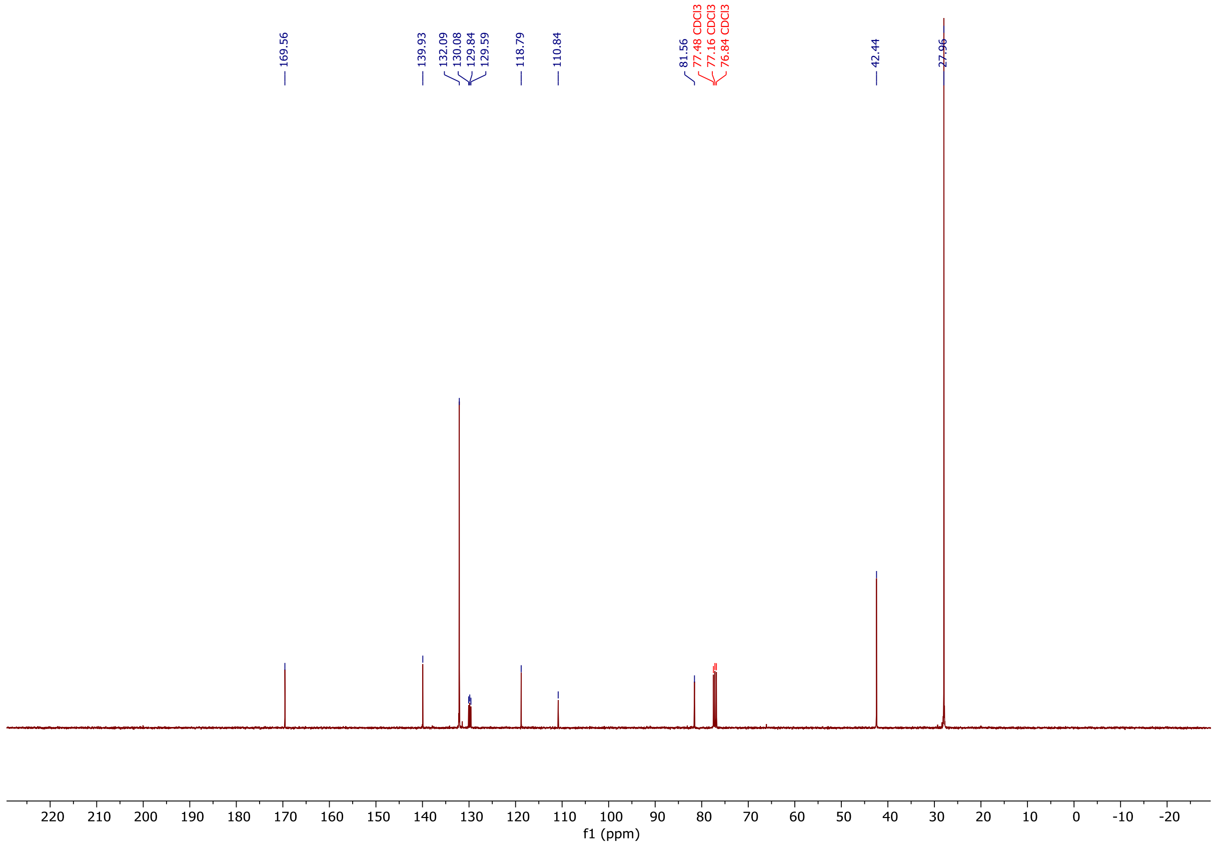
169.56
139.93
132.09
130.08
129.84
129.59
118.79
110.84
81.56
77.48 CDCl3
77.16 CDCl3
76.84 CDCl3
42.44
27.96
220 210 200 190 180 170 160 150 140 130 120 110 100 90 80 70 60 50 40 30 20 10 0 -10 -20
f1 (ppm)

Compound 16

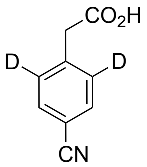

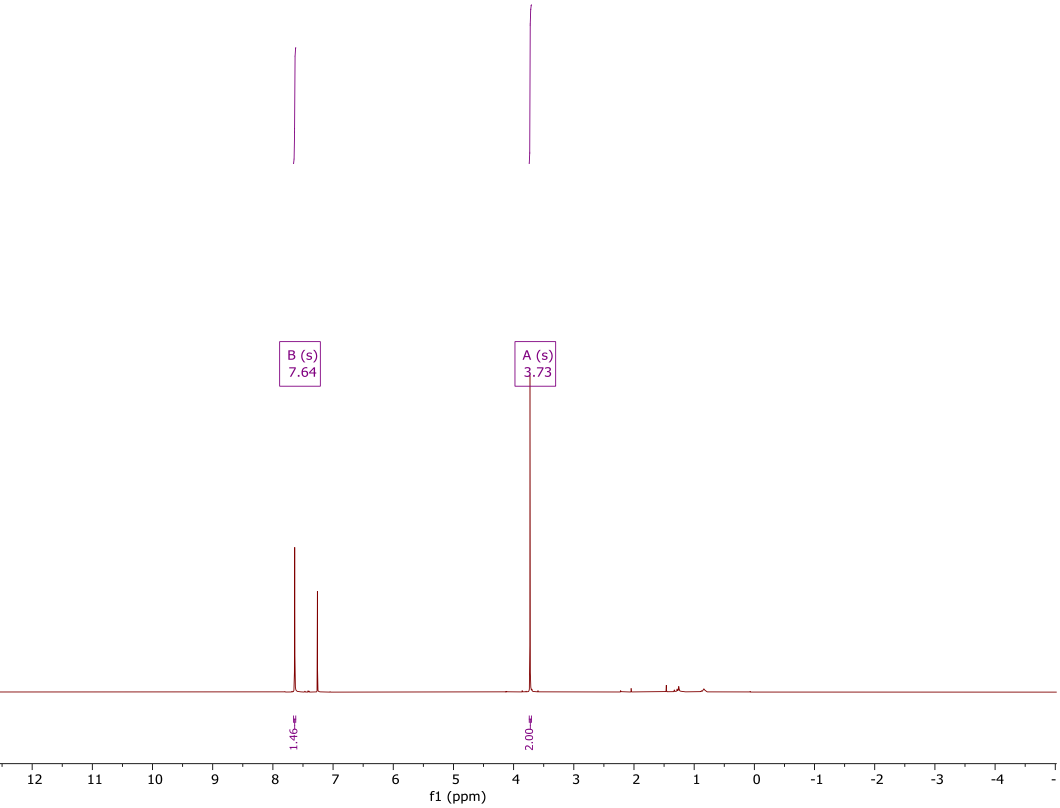

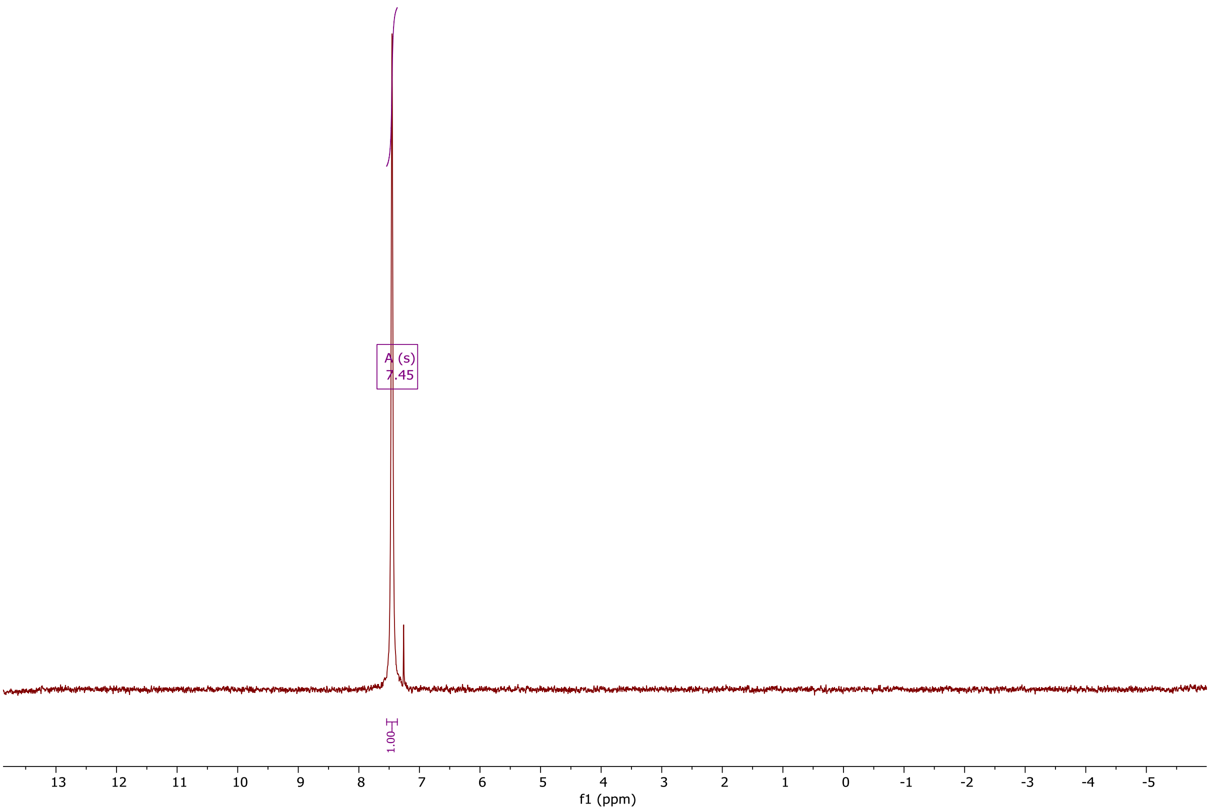

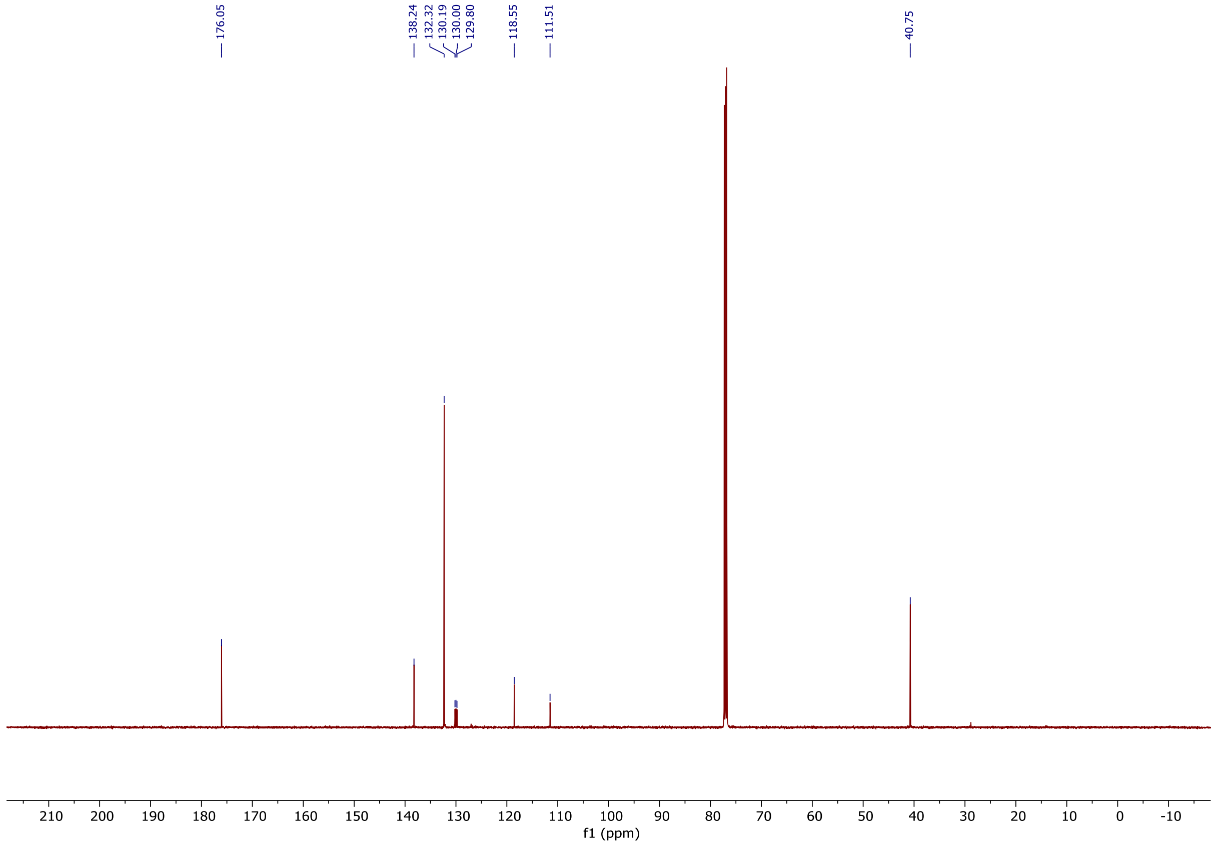
176.05
138.24
132.32
130.19
130.00
129.80
118.55
111.51
40.75
210 200 190 180 170 160 150 140 130 120 110 100 90 80 70 60 50 40 30 20 10 0 -10
f1 (ppm)

Compound 1i-d_7

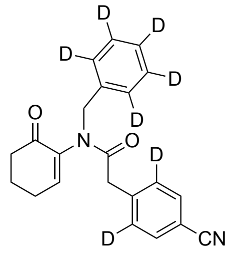

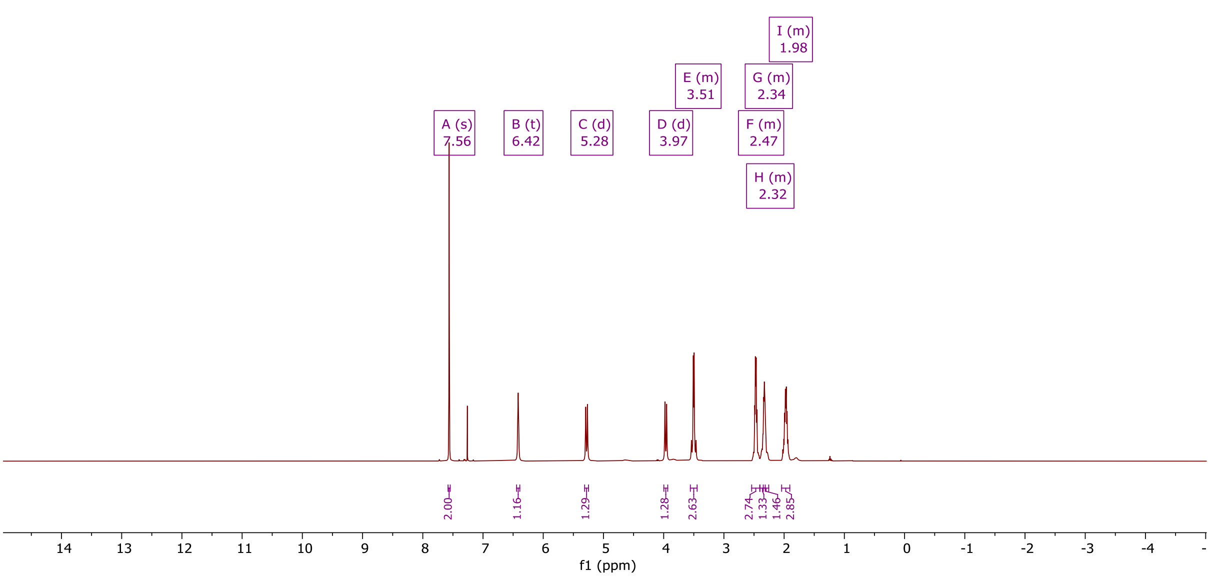

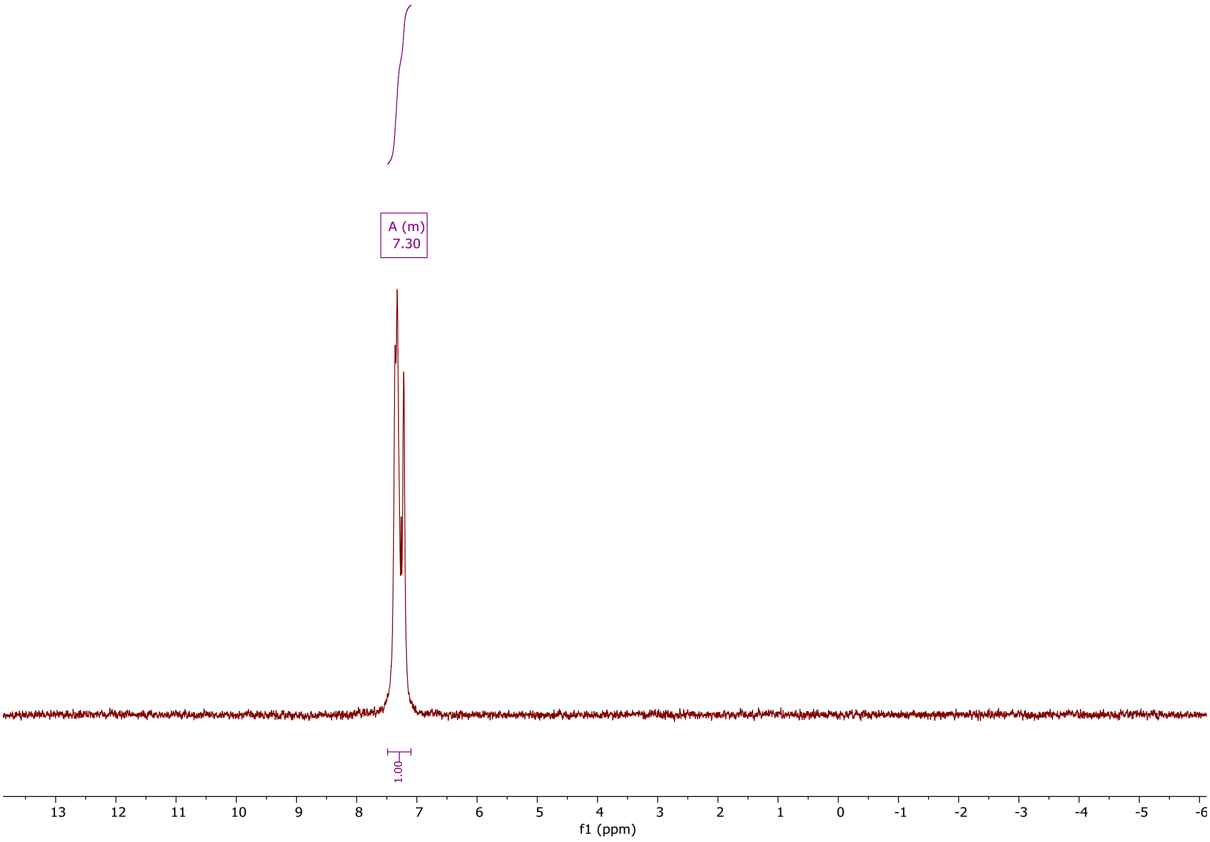

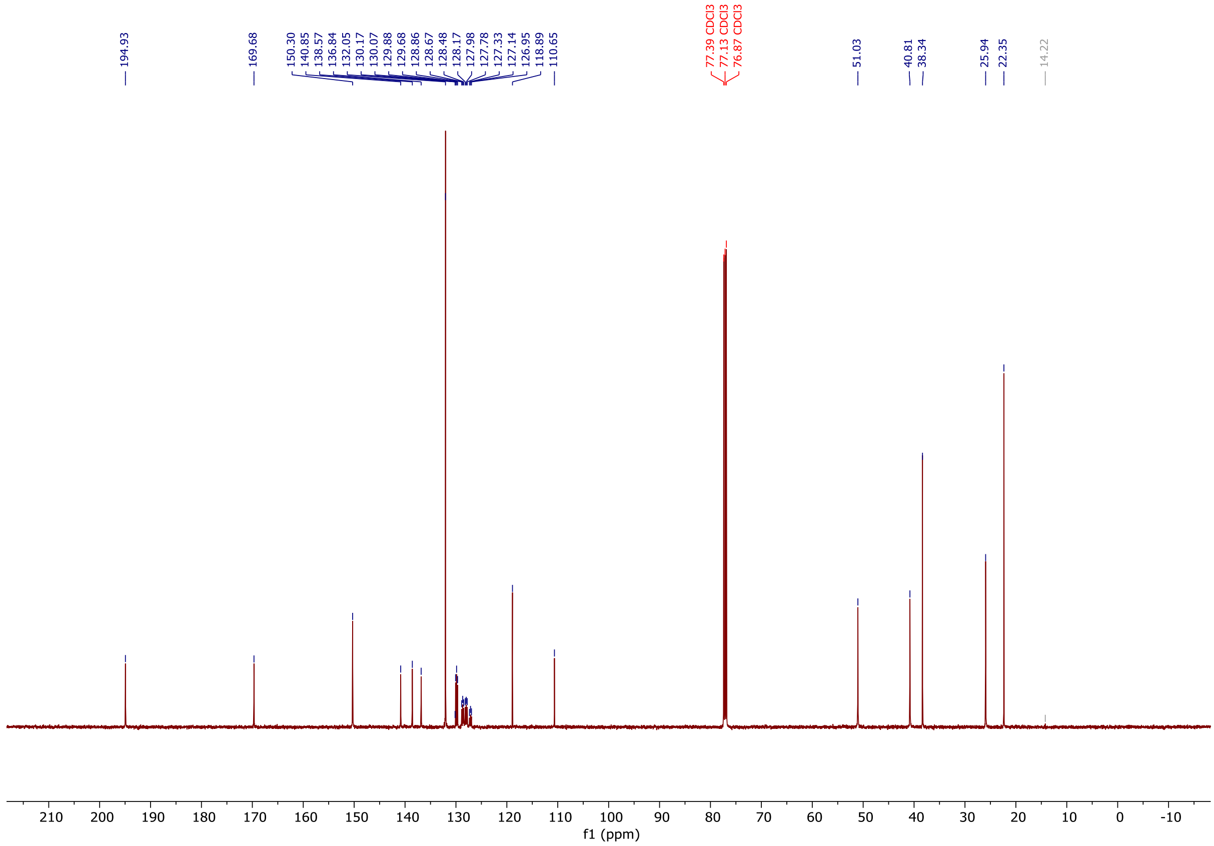
194.93
169.68
150.30
140.85
138.57
136.84
132.05
130.17
130.07
129.88
129.68
128.86
128.67
128.48
128.17
127.98
127.78
127.33
127.14
126.95
118.89
110.65
77.39 CDCl3
77.13 CDCl3
76.87 CDCl3
51.03
40.81
38.34
25.94
22.35
14.22
210
200
190
180
170
160
150
140
130
120
110
100
90
80
70
60
50
40
30
20
10
0
-10
f1 (ppm)
